# Childhood-onset dystonia-causing *KMT2B* variants result in a distinctive genomic hypermethylation profile

**DOI:** 10.1186/s13148-021-01145-y

**Published:** 2021-08-11

**Authors:** Andrea Ciolfi, Aidin Foroutan, Alessandro Capuano, Lucia Pedace, Lorena Travaglini, Simone Pizzi, Marco Andreani, Evelina Miele, Federica Invernizzi, Chiara Reale, Celeste Panteghini, Maria Iascone, Marcello Niceta, Ralitza H. Gavrilova, Laura Schultz-Rogers, Emanuele Agolini, Maria Francesca Bedeschi, Paolo Prontera, Matteo Garibaldi, Serena Galosi, Vincenzo Leuzzi, Paola Soliveri, Rory J. Olson, Giovanna S. Zorzi, Barbara M. Garavaglia, Marco Tartaglia, Bekim Sadikovic

**Affiliations:** 1 Genetics and Rare Diseases Research Division, Ospedale Pediatrico Bambino Gesù, IRCCS, 00146 Rome, Italy; 2 Department of Pathology and Laboratory Medicine, Western University, London, ON N6A 3K7 Canada; 3 Verspeeten Clinical Genome Centre, London Health Sciences Centre, London, Canada; 4 Department of Neuroscience, Ospedale Pediatrico Bambino Gesù, IRCCS, Rome, Italy; 5 Department of Pediatric Onco-Hematology and Cell and Gene Therapy, Ospedale Pediatrico Bambino Gesù, IRCCS, Rome, Italy; 6 Medical Genetics and Neurogenetics Unit, Fondazione IRCCS Istituto Neurologico C. Besta, Milano, Italy; 7 Medical Genetics Laboratory, ASST Papa Giovanni XXIII, Bergamo, Italy; 8 Center for Individualized Medicine, Mayo Clinic, Rochester, MN USA; 9 Translational Cytogenomics Research Unit, Bambino Gesù Children’s Hospital, IRCCS, Rome, Italy; 10 Medical Genetic Unit, Fondazione IRCCS Ca’ Granda Ospedale Maggiore Policlinico, Milan, Italy; 11 Maternal-Infantile Department, University Hospital of Perugia, Perugia, Italy; 12 Department of Neuroscience, NESMOS, Sapienza University, Sant’Andrea Hospital, Rome, Italy; 13 Department of Human Neuroscience, Child Neurology and Psychiatry, Sapienza University, Rome, Italy; 14 Department of Neurology, Fondazione IRCCS Istituto Neurologico C. Besta, Milano, Italy; 15 Department of Child Neurology, Fondazione IRCCS Istituto Neurologico C. Besta, Milano, Italy; 16 Molecular Diagnostics Division, London Health Sciences Centre, London, Canada

**Keywords:** DNA methylation, Episignature, KMT2B, Dystonia 28

## Abstract

**Background:**

Dystonia is a clinically and genetically heterogeneous movement disorder characterized by sustained or intermittent muscle contractions causing abnormal, often repetitive, movements and/or postures. Heterozygous variants in lysine methyltransferase 2B (*KMT2B*), encoding a histone H3 methyltransferase, have been associated with a childhood-onset, progressive and complex form of dystonia (dystonia 28, DYT28). Since 2016, more than one hundred rare *KMT2B* variants have been reported, including frameshift, nonsense, splice site, missense and other in-frame changes, many having an uncertain clinical impact.

**Results:**

We characterize the genome-wide peripheral blood DNA methylation profiles of a cohort of 18 patients with pathogenic and unclassified *KMT2B* variants. We resolve the “episignature” associated with *KMT2B* haploinsufficiency, proving that this approach is robust in diagnosing clinically unsolved cases, properly classifying them with respect to other partially overlapping dystonic phenotypes, other rare neurodevelopmental disorders and healthy controls. Notably, defective KMT2B function in DYT28 causes a non-random DNA hypermethylation across the genome, selectively involving promoters and other regulatory regions positively controlling gene expression.

**Conclusions:**

We demonstrate a distinctive DNA hypermethylation pattern associated with DYT28, provide an epigenetic signature for this disorder enabling accurate diagnosis and reclassification of ambiguous genetic findings and suggest potential therapeutic approaches.

**Supplementary Information:**

The online version contains supplementary material available at 10.1186/s13148-021-01145-y.

## Background

Dystonia is a neurological hyperkinetic movement condition characterized by sustained or intermittent muscle contractions causing abnormal movements and/or postures. Dystonia’s symptoms can be characterized based on body location into focal, segmental or generalized forms, as well as into isolated or combined forms, whether other movement disorders or neurological signs occur or not [1]. Childhood-onset dystonias, which are usually genetically determined and can be characterized by other additional neuropsychiatric and systemic features, pose a significant diagnostic challenge for clinicians [1].

The advent of high-throughput sequencing has revolutionized the landscape of dystonia’s genetics, enabling identification of several novel disease genes in the last decade, most of them causing complex forms [2]. Recently, heterozygous variants in lysine methyltransferase 2B (*KMT2B*; MIM *606834), encoding a histone H3 methyltransferase, have been associated with a childhood-onset, progressive and complex form of dystonia named dystonia 28 (DYT28; MIM #617284) [3–5]. Pathogenic *KMT2B* variants generally are de novo events and result in loss-of-function of the methyltransferase, indicating haploinsufficiency as the mechanism of disease [3–6]. Although the pathomechanistic consequences of *KMT2B* variants require further study, *KMT2B* haploinsufficiency is postulated to affect CNS development and function by perturbing the expression of key genes involved in neurodevelopment and motor control [7, 8]. Of note, while *KMT2B* was identified as a dystonia-associated gene only recently, more than one hundred rare or private heterozygous single nucleotide variants (SNVs) as well as insertions/deletions (indels) have been identified in this gene, the majority predicted to cause haploinsufficiency [4–6, 9]. A small proportion of *bona fide* pathogenic *KMT2B* variants are missense and have been reported to affect conserved functional domains of the protein (*i.e.*, the PHD-like and FYR-N domains). However, a number of clinically unclassified missense variants have been described and, in a significant proportion of cases, these variants are inherited from a healthy parent [3, 6]. Currently, no functional assay is available to classify these variants.

KMT2B belongs to the family of Set1-Trithorax-type methyltransferases, which are enzymes that specifically methylate histone 3 at lysine 4 (H3K4) and play a key role in chromatin remodeling and gene expression [10]. Mammals possess six SET-related H3K4 methyltransferases (*i.e.*, KMT2A (MLL1), KMT2B (MLL2), KMT2C (MLL3), KMT2D (MLL4), SETD1A and SETD1B), which are dynamically regulated during cell-type specification, in a spatially and temporally non-redundant way [7, 11]. H3K4 can be mono- (H3K4me1), di- (H3K4me2) and tri-methylated (H3K4me3), with each modification differentially distributed in promoters, enhancers and other regulatory regions of actively transcribed genes [12]. While SETD1A and SETD1B are major contributors of global histone H3K4 tri-methylation (H3K4me3), KMT2B and KMT2A H3K4me3 marks occur at the transcription start site (TSS) regions of a more restricted set of genes. Different from the other members of the KMT2 family, KMT2C and KMT2D catalyze H3K4me1/2, which are particularly enriched at enhancers [12].

There is a growing evidence that variants in genes encoding proteins involved in the maintenance of chromatin remodeling show unique DNA methylation (DNAm) patterns (known as “episignatures” or EpiSigns), and that these DNAm signatures can be used as highly specific and robust biomarkers for an increasing number of disorders caused by mutations in these genes [13–16]. These genome-wide DNAm signatures currently include over 40 rare neurodevelopmental disorders associated with more than 60 genes [13, 17]. Of note, these disease-specific episignatures are detectable in peripheral blood despite the variable nature and complexity of diseases and the high variance characterizing the DNAm of genomes from different cells and tissues [13, 17]. As such, DNA methylation testing has recently been implemented in clinical diagnosis of patients with rare disorders [17].

By characterizing the genome-wide DNA methylation profiles of a cohort of clinically and genetically confirmed DYT28 patients, here we define a disease-specific episignature for this disorder and show that the identified methylation pattern could be successfully used to classify *KMT2B* variants of uncertain significance (VUS) and help diagnose clinically unsolved cases. We also provide evidence that dysfunctional KMT2B in DYT28 causes DNA hypermethylation of promoters and other regulatory regions positively controlling gene expression, which collectively points out toward a general repression of transcriptional activity as pathogenic mechanism in DYT28.

## Methods

### Study cohort

The study included eight case individuals (five females and three males) with *bona fide* pathogenic *KMT2B* variants and clinically confirmed DYT28 (labelled as DYT28_Pathogenic), and 10 subjects (seven females and three males) showing variable clinical phenotypes and *KMT2B* VUS (labelled as DYT28_VUS), according to the American College of Medical Genetics and Genomics/Association for Molecular Pathology (ACMG/AMP) guidelines for interpretation of genomic sequence variants [18] (Additional file 9: Table S1). Additionally, nine control samples were also used (labelled as Control_Testing). The study was approved by the Ospedale Pediatrico Bambino Gesù Ethical Committee (1702_OPBG_2018) and the Western University Research Ethics Board (REB 106302). All DNA samples and clinical records were pseudonymized. DNA specimens were collected following procedures in accordance with the ethical standards of the declaration of Helsinki protocols, with signed informed consents from the participating subjects/families. Peripheral blood DNA was extracted using the salting out procedure [19] (patients 1, 2, 5–11, 14 and 18) or the Qiagen DNA extraction kit (patients 3, 4, 12, 13, 15–17). All variants had been confirmed by Sanger sequencing using the Big-Dye terminator reaction Kit v.1.1 on a 3100XL Genetic Analyzer Automated Sequencer (Applied BioSystems) (patients 1, 2, 5–11, 14 and 18) or using the Big-Dye terminator reaction Kit v.3.1 on a SeqStudio Genetic Analyzer (Applied Biosystems) (patients 3, 4, 12, 13, 15–17) (Additional file 1: Figure S1).

### DNA methylation profiling and data analysis

Following bisulfite conversion, samples were analyzed using Illumina Infinium MethylationEPIC BeadChips, according to the manufacturer’s protocol. Data analysis was carried out as previously reported [13, 14]. Briefly, IDAT files containing methylated and unmethylated signal intensity were imported into R v.4.0.2 for analysis following normalization with background correction using the minfi package [20]. Probes located on X/Y chromosomes or known to cross-react with chromosomal locations other than their target regions contain SNPs at or near the CpG sites, and suggested by Illumina to be cross-reactive were excluded, resulting in 776,314 probes remaining for the analysis. Arrays having more than 5% probe failure rate and those that were previously identified in our database to impose batch effect were excluded from the analysis. Sex of the two unknown samples was predicted using minfi package [20], whereas for the age estimation the wateRmelon package was used [21]. The eight samples with DYT28-causing *KMT2B* variants (DYT28_Pathogenic) were used to identify the episignature, while the 10 additional samples (DYT28_VUS) were used for validation and classification by blind testing (Additional file 9: Table S1).

Principal component analysis (PCA) was performed to inspect any batch effect and identify outlier samples. MatchIt package was used to select best-matching controls from EPIC arrays in the EpiSign Knowledge Database (EKD) at the London Health Sciences Center (LHSC) considering age, and sex, as matching variables, providing a control sample size seven times larger than that of tested cases (56 controls labelled as Control_Training) [13, 17]. The control cohort characterization is provided in Additional file 10: Table S2.

Methylation levels (beta values) were converted to M-values, which were used for linear regression modeling by means of empirical Bayes moderated t-statistic corrected for false discovery rate (FDR) using the Benjamini-Hochberg (BH) method (limma package [22]) to identify differentially methylated probes (DMPs). Estimated blood cell proportions for each sample were added to the model matrix to reduce the bias associated with those confounding variables [23]. The most informative 1,000 probes were identified considering the interaction between the effect size (absolute mean methylation difference between DYT28 samples and batch controls) and *p*-value [13, 14]. Receiver’s operating curve characteristic analysis was performed to identify the top 500 of these 1000 probes, then probes with a Pearson’s pairwise correlation > 0.9 were removed, resulting in identification of 196 independent probes.

Hierarchical clustering was performed using the gplots package. Multidimensional scaling (MDS) was performed by scaling of the pairwise Euclidean distances between samples. The e1071 R package was used to train a support vector machine (SVM) and for construction of a prediction model to calculate what we refer to as “methylation variant pathogenicity” (MVP) scores [13]. The eight samples with DYT28, and samples from the EKD databases split into two cohorts, 75% of control subjects (> 1000 individuals) and 75% of patients from 38 other neurodevelopmental disorders/rare diseases (NDDs/RDs) in the EKD (> 1000 individuals) were used as training set. The remaining 25% control subjects and patients with *KMT2B* VUS were considered as the testing set (Additional file 9: Table S1), in order to improve the specificity of the classifier. An MVP plot was generated to assess specificity of the classification model.

### Functional analysis of differentially methylated regions

To detect the differentially methylated regions (DMRs), the DMRcate package was used [24], and regions containing at least five different CpGs within 1 kb with a minimum methylation difference of 10% and a Fisher’s multiple comparison *P* < 0.01 were selected. Functional analysis of differentially methylated regions was performed by means of missMethyl R package [25] and WebGestalt [26]. Genomic region enrichment with respect to the EPIC annotation manifest for the episignature probes was calculated by means of Fisher’s exact test; for the region-level analysis of DMRs, we took advantage of bedTools (v.2.30) [27] Fisher command, considering DMRs showing overlapping fraction > 50% with the genomic regions annotated as 5’UTR, TSS1500, TSS200, 1stExon, Body, 3’UTR regions. DMRs comparison to the 127 reference epigenomes from the NIH Roadmap Epigenomics Consortium was carried out by means of GIGGLE, a fast and highly scalable genomic interval searching strategy, and evaluated using the GIGGLE score, that combines the estimation of the enrichment for observed versus expected (odds ratio), and Fisher's two tailed tests p-value [28].

## Results

### DYT28 is associated with hypermethylated DNA episignature in blood

**Fig. 1:**
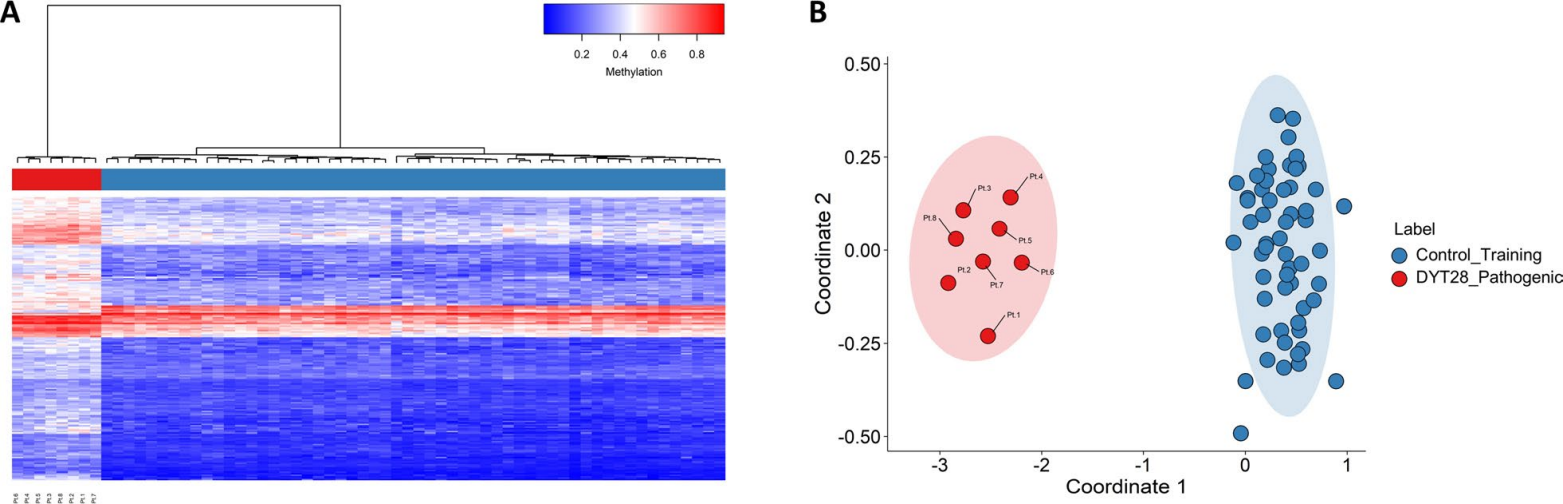
DYT28 episignature identification. **A** Hierarchical clustering with Ward’s method on Euclidean distance was performed. In the heatmap plot, each row illustrates a selected CpG site, and each column depicts a sample. The heatmap color scale indicates the range of methylation level; from blue (no methylation or 0) to red (full methylation or 1). The detected episignature clearly differentiates between samples with pathogenic *KMT2B* variants and controls. **B** The first two dimensions of a MDS plot using the selected probes separate the samples with pathogenic variants in *KMT2B* from control samples. Blue circles represent control subjects and red circles indicate subjects with pathogenic *KMT2B* variants and a confirmed diagnosis of DYT28. Ellipses indicate 95% confidence interval

Eight individuals carrying *bona fide* pathogenic variants in *KMT2B* (NM_014727.2), as *per* the ACMG/AMP guidelines [18], were included in the study. These variants can be considered as representative of the DYT28-causing *KMT2B* variants, as they included frameshift changes (Pt. 1–4 and 7), in-frame deletions (Pt. 6), and missense substitutions (Pt. 5 and 8) (Additional file 9: Table S1). Variants in Pt. 1, 2, 5–8 had been identified by WES in a cohort of 65 patients with genetically unclassified childhood-onset dystonia [3, 5, 6, 9]. The two frameshift variants in Pt. 3 and 4, both resulting in premature termination, were identified in the frame of diagnostic genetic testing. The clinical and molecular characterization of the DYT28 cohort is reported in Additional file 9: Table S1. Comparison of DNA methylation patterns between the peripheral blood DNA specimens of these 8 samples and 56 controls selected from our database based on matching for age and sex resulted in identification of 196 DMPs (methylation difference > 10%, FDR < 0.01, adjusted for blood cell-type compositions). Notably, more than 96% of probes (189 out of 196) exhibited relative hypermethylation (Additional file 11: Table S3). Of note, while the most robust and significant methylation change described in this episignature classifier involved hypermethylated regions in DYT28 (Additional file 2: Figure S2), the majority of the probes in these samples were found as slightly hypomethylated compared to the control group (Additional file 3: Figure S3). In order to assess the robustness of the episignature in differentiating between case and control samples, hierarchical clustering (Fig. 1A) and MDS analysis (Fig. 1B) were performed, resulting in clear separation between groups. Eight rounds of cross-validation on MDS plot were performed using different combinations of samples with pathogenic *KMT2B* variants (*n* = 7) as training set and single samples with pathogenic variants as testing set. In all steps, the testing samples were correctly clustered with the training samples further providing evidence of a robust common DNA methylation signature (Additional file 4: Figure S4). While the two DYT28-associated missense variants identified in Pt. 5 and Pt.8 satisfied the ACMG/AMP criteria as *bona fide* pathogenic variants [18], the same analysis was also performed excluding those patients (Additional file 5: Figure S5). Comparison of the DNA methylation patterns of these six samples and the same 56 Control-Training samples confirmed the previous results.

### The DYT28-specific episignature allows functional classification of *KMT2B* variants

The generated DYT28-specific episignature was used to test 10 samples with unclassified *KMT2B* variants that had been identified in diagnostic genetic testing (Additional file 9: Table S1). Among these variants, two missense substitutions, for which the inheritance pattern could not be established, had been found in two subjects with childhood-onset dystonia fitting DYT28 (Pt. 9 and 10). Two missense variants, both inherited from an apparently unaffected parent, had previously been reported in subjects with DOPA-responsive cervical and mandibular dystonia (Pt. 14) and slowly progressive dystonia and dyskinesias with caudo-rostral progression (Pt. 18) without decisive evidence for clinical relevance [3, 9]. Patient 13, who was characterized by progressive generalized dystonia with caudo-cranial progression, presented with an in-frame duplication previously reported as VUS in ClinVar (VCV000808518.4), and annotated in gnomAD v.2.1 (MAF = 1.18 × 10^−3^). The only other variant that had previously been reported in the general population was p.Ser2390Leu identified in patient 15 (MAF = 4.02 × 10^−5^, gnomAD), who showed gait instability and dystonia of the upper and lower limbs during paroxysmal attacks. Finally, we selected four additional private missense variants predicted as pathogenic by CADD [29] and MetaDome [30] algorithms (Additional file 9: Table S1) in patients showing late-onset dystonia (Pt. 11 and 12) and congenital movement disorders (Pt. 16 and 17). The de novo occurrence of these variants could be ascertained only in two cases (Pt. 16 and 17).

**Fig. 2:**
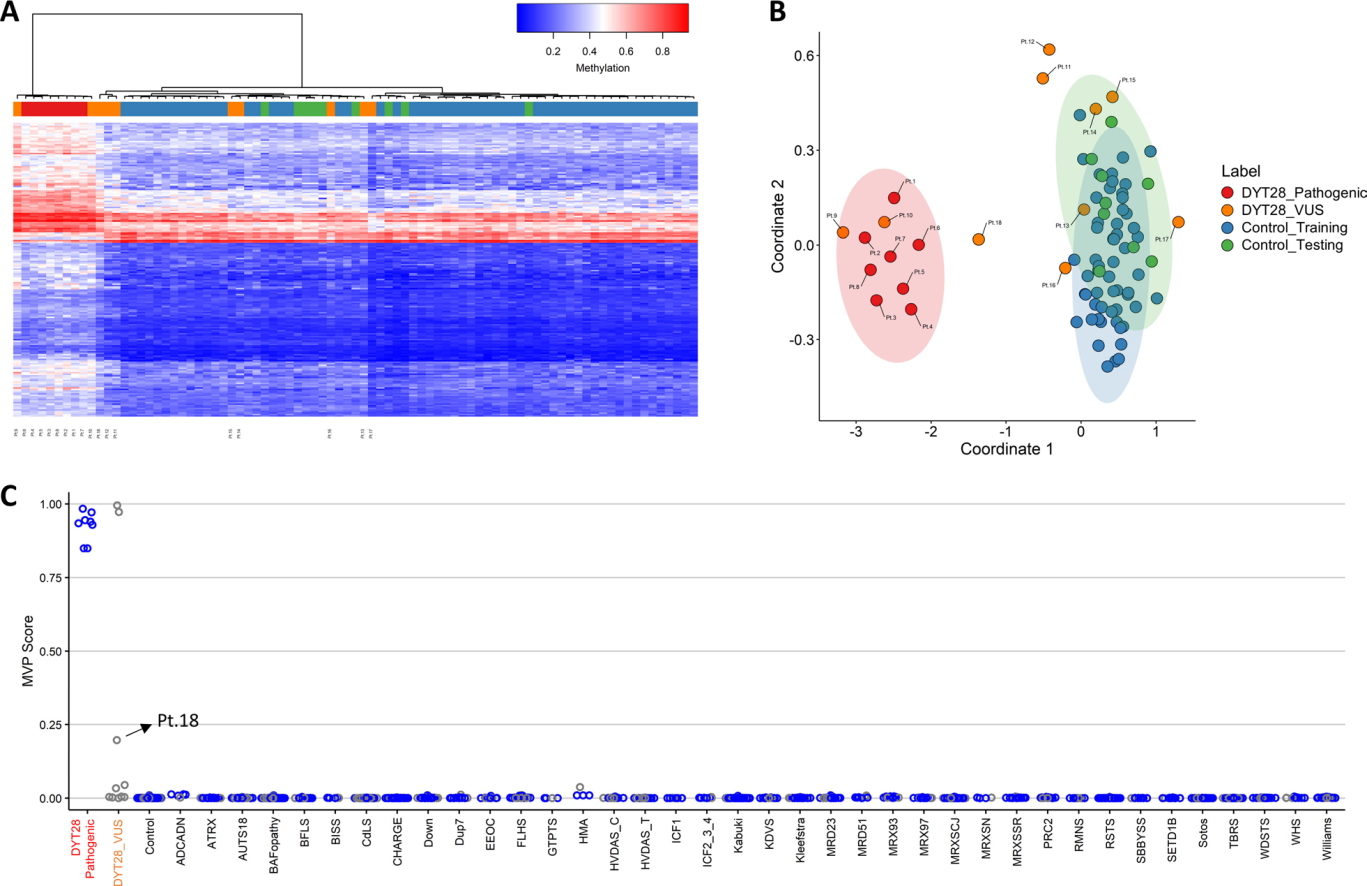
DNA methylation pattern analysis yields full sensitivity and specificity in classifying individuals affected by DYT28. Hierarchical clustering analysis (**A**) and MDS (**B**) plots are used to classify VUS (used as the testing set) with respect to pathogenic *KMT2B* variants and control samples (used as the training set). **C** A support vector machine (SVM) was used to classify samples and calculate probability scores reaches full sensitivity and specificity for *KMT2B* variants. The classifier was trained using pathogenic variants, controls and other NDDs/RDs. 75% of controls and NDD/RD samples used for training (blue), 25% for testing (grey). Ellipses indicate 95% confidence interval

The genome-wide methylation data obtained from blood DNA specimens of the 10 patients with heterozygous *KMT2B* VUS (Pt. 9 to 18) were analyzed by hierarchical clustering and MDS analysis using the 196 informative probes defining the DYT28-specific episignature (Fig. 2A, B). Two samples of the testing set (Pt. 9 and 10) clustered with the DYT28 cohort, seven grouped with controls and one sample (Pt. 18) showed an intermediate position.

**Fig. 3:**
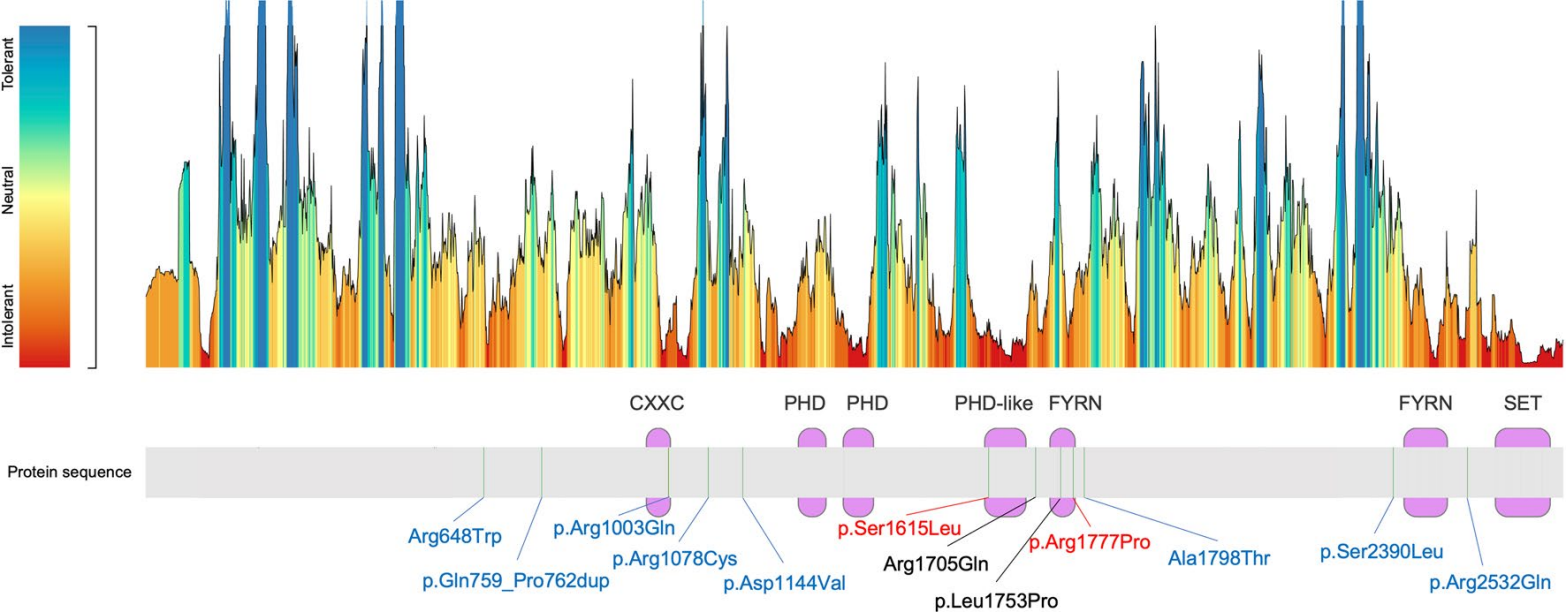
*KMT2B* missense variants distribution. The diagram on the top illustrates the KMT2B’s tolerance to missense changes landscape according to MetaDome web server. The protein structure is depicted on the bottom, along with the missense variants analyzed in the present work. Purple boxes indicate protein domains. Green bars depict mutated residues. Known pathogenic variants are written in black [9]; variants classified as disease-causing in this work (red) and those classified as benign (blue) by the identified episignature are also shown

To test the use of the episignature in a clinical setting, the recently developed SVM-based classifier was used [13, 17], trained by comparing the eight samples with *bona fide* pathogenic variants (Pt. 1–8) against the 10 VUS samples (Pt. 9–18), controls and a large set of individuals affected by various forms of NDDs or other RDs included in the EKD. All patients from other NDDs/RDs and controls were classified with low probability scores, indicating the high level of specificity of the DYT28 classifier (Fig. 2C). Consistent with the MDS and hierarchical clustering analyses, two missense *KMT2B* VUS, p.Ser1615Leu and p.Arg1777Pro (Pt. 9 and 10), were classified as disease-causing, while all other variants were scored with a significantly lower score, ruling out their clinical relevance in DYT28. Interestingly, the DYT28-related missense variants did not show a preferred localization in putative constrained regions and functional domains along the protein sequence. Nevertheless, we noted the existence of an apparent mutational hotspot for the four pathogenic missense variants between codons 1,615 and 1,777 (Fig. 3), similarly to what previously reported in the literature [3]. The clinical re-evaluation of patients 11 to 18 documented features or a natural history of disease that did not fit the classical presentation of DYT28, clinically validating the conclusions based on the episignature analysis (Additional file 9: Table S1).

Of note, the same validation analysis was performed considering the episignature generated excluding Pt.5 and Pt.8 (Additional file 6: Figure S6). As shown, hierarchical clustering and MVP classifier properly classified Pt.5, Pt.8, Pt.9 and Pt.10 within the DYT28 group, further documenting the robustness of the approach. An apparently lower specificity, however, was provided by MDS analysis, confirming the dependency of the signature classification efficacy/specificity on sample size.

### DYT28-causing *KMT2B* variants are associated with a non-random distribution of hypermethylation in the genome

H3K4 trimethylation is highly enriched at active promoters near TSS and is positively correlated with transcription [10, 11, 31, 32]. Since this non-random distribution of H3K4me3 throughout the genome, we assessed the genome-wide distribution of DMRs (defined as stretches harboring ≥ 5 consecutive CpGs) in DYT28. The analysis allowed us to identify significant methylation changes in 146 genomic regions (hg19 genome assembly), the vast majority represented by relative hypermethylation in Pt.1 to Pt.8 (144 out of 146; Additional file 12: Table S4, Additional file 7: Figure S7). Considering all probes contained in these 146 regions, we first assessed the overall fit of the methylation patterns characterizing the 10 samples with *KMT2B* VUS with those obtained for the DYT28 and control groups (Additional file 8: Figure S8). As shown, the methylation levels for Pt.9 and Pt.10 were more similar to those characterizing patients carrying pathogenic *KMT2B* variants (93 and 89% of probes, respectively), while Pt.11–18 clearly diverge from the DYT28-specific pattern for a significant proportion of probes (from 55 to 12% of probes with methylation levels similar to DYT28). These data further validate the ability of the identified episignature in functionally classifying the tested *KMT2B* VUS.

**Fig. 4:**
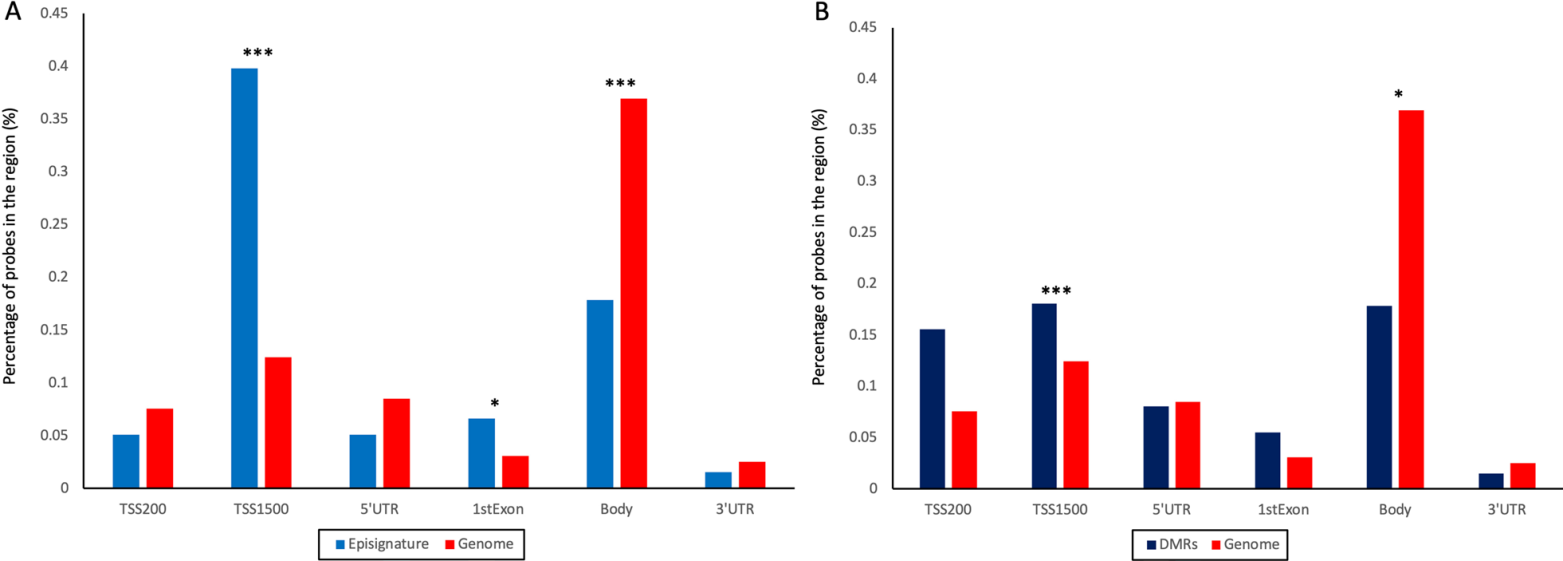
DYT28 is characterized by a hypermethylation pattern on specific gene regions. Histograms illustrate the non-random gene region distribution for episignature’s probes (**A**), and DMRs (**B**) in DYT28 patients. Fisher’s exact test was used to report the statistical significance of the enrichment/depletion with respect to genomic background (EPIC array). TSS200, transcription start sites 1–200; TSS, transcription start sites 201–1500; IGR, intergenic regions. **A** Percentage and statistical significance of feature enrichment for DYT28 episignature: *P < 0.01; **P < 10^−3^ ***P < 10^−6^; **B** Percentage and statistical significance of feature enrichment for DMRs in DYT28 patients: *P < 0.05; **P < 0.01 ***P < 0.001

Aiming to functionally characterize the genomic methylation differences in DYT28, we performed gene set enrichment analyses considering the genes mapping within DMRs by means of missMethyl [25] and WebGestalt [26] tools, failing to identify any enrichment for specific biological pathways (MSigDB’s hallmark, Gene Ontology, KEGG, Reactome, Panther pathways), including those functionally linked to neurodevelopment and neuronal physiology (data not shown). To further gain insights on the functional implications of the observed hypermethylated status associated with defective KMT2B function, we then explored the hypothesis of a non-random distribution of DNA hypermethylation in genomic regions typically enriched in H3K4me3, such as promoters. First, we noted that DMPs constituting the episignature were enriched for genomic regions that are generally poorly methylated in actively transcribed genes (*e.g.*, gene promoters [TSS1500] and first exon regions [1stExon]) (Fig. 4A). Consistently, gene body regions, which are known to be positively correlated with gene expression when methylated, where significantly underrepresented (Fig. 4A). To further delve into these results, all probes located within the annotated gene regions encompassing the identified DMRs were tested for enrichment analysis (Fig. 4B, Additional file 12: Table S4). Consistently, we observed a qualitatively similar pattern of enrichment involving promoter regions coupled with a depletion of gene body regions (Fig. 4B).

**Fig. 5:**
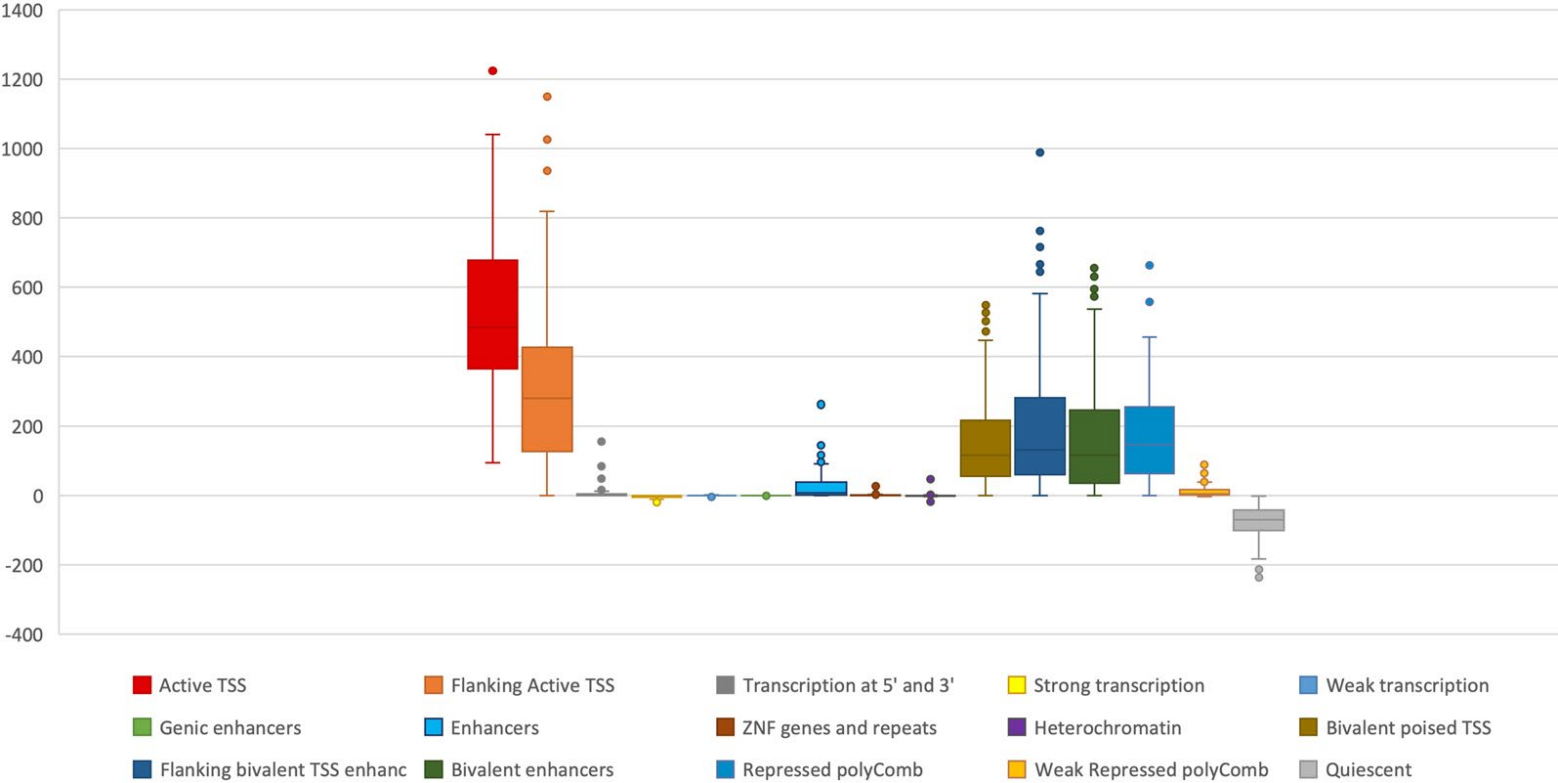
DMRs enrichment for 15 chromatin states in 127 reference epigenomes in DYT28. Boxplots display enrichment scores (GIGGLE combo scores) distribution for 15 chromatin states as defined by the NIH Roadmap Epigenomics project. The active states (associated with expressed genes) consist of active transcription start site (TSS) proximal promoter states (Active TSS and Flanking Active TSS), a transcribed state at the 5′ and 3′ end of genes showing both promoter and enhancer signatures (Transcription at 5' and 3'), actively transcribed states (Strong Transcription and Weak Transcription), enhancer states (Enhancers and Genic Enhancers) and a state associated with zinc finger protein genes (ZNF genes and repeats). The inactive states consist of constitutive heterochromatin, bivalent regulatory states (Bivalent poised TSS, Flanking bivalent TSS enhancers and Bivalent enhancers), repressed PolyComb states (Repressed PolyComb and Weak Repressed PolyComb) and a quiescent state

To further support the correlation between DNA methylation levels in DYT28 and the H3K4me3 epigenetic modification, we assessed the overlap of DMRs against the 127 reference epigenomes representing all major cell lineages in human body generated by the NIH Roadmap Epigenomics Consortium [12]. This analysis allowed us to inspect the enrichment of DMRs for regions specifically marked by H3K4me3 (*i.e.*, active TSSs) and the other 15 associated chromatin states as defined by the consortium (Fig. 5). The analysis documented a clear enrichment for the chromatin states associated to H3K4me3 marks (*i.e.*, active TSS and flanking active TSS) (Pairwise Wilcoxon Test’s adjusted p-value < 2.1 10^−7^), while regions in quiescent state were consistently underrepresented (Fig. 5).

## Discussion

Here we report that DYT28 is associated with a genome-wide hypermethylated profile, showing that the identified methylation episignature can be successfully used to diagnose this disorder. DYT28 is a recently identified form of dystonia caused by heterozygous inactivating *KMT2B* variants. The disorder is generally characterized by initial lower limb involvement during childhood followed by a more general involvement, usually including the bulbar and cranio-cervical muscles leading to dysarthria and dysphonia [3, 6]. Of note, the clinical phenotype associated with *KMT2B* variants is emerging as variable and has been reported to include conditions not manifesting dystonia at all [6]. Given the variable phenotypic presentation, lack of validated biomarkers and scarcity of experimental assays to promptly and straightforwardly assess KMT2B function, current interpreting of the clinical relevance of *KMT2B* variants can be challenging. In line with these considerations, bioinformatic strategies recently proposed showed lack of specificity [6, 33]. Although protein truncating variants and whole-gene deletions classification under a haploinsufficiency paradigm could be straightforward, caution is needed for missense changes and in-frame indels in a clinical setting. Indeed, even using combination of state-of-the-art in silico prediction algorithms (e.g., CADD [29]), regional constraint analyses (MetaDome [30]), as well as segregation and population frequency data, did not help to identify *bona fide* pathogenic variants among the subset of *KMT2B* missense variants included in this study.

The identified episignature in peripheral blood DNA of DYT28 patients is defined by a relatively small number of hypermethylated CpG sites (< 200) and proved to be robust and effective in classifying DYT28 patients, with respect to other partially overlapping dystonic phenotypes, other NDDs/RDs, as well as healthy controls. By using this episignature, we correctly classified missense changes not residing in constrained regions (Pt. 8) [30, 34], and excluded pathogenicity in cases with partially overlapping phenotype, or heterozygous for low-frequency alleles predicted as deleterious by in silico algorithms with unknown segregation (Pt. 15) [29, 30, 35], or documented to occur as de novo events (Pt. 16 and 17). Of note, our classification algorithm rejected a diagnosis of DYT28 in patient 18, who carried the p.Arg1003Gln variant in *KMT2B*, previously reported as VUS and predicted to have a destabilizing structural effect by homology modeling [9]. We would note, however, that while a confirmation of a related episignature in a patient with a genetic VUS is considered strong functional evidence for pathogenicity under the PS3/BS3 criterion [13, 17], using the ACMG/AMP sequence variant interpretation framework [18, 36], a negative result in a patient with or without a known genetic variant, while indicative, does not rule out pathogenicity. Although majority of the genes with episignatures currently have a single common episignature mapped, genes with multiple episignatures have been described, and assessment of pathogenicity of variants outside the established reference range warrants caution [17].

The present epigenomic analysis also provides relevant insights on the molecular effects of DYT28-causing *KMTB2* variants. A significant genome-wide shift in DNA methylation was documented: this hypermethylated status was not associated with a specific enrichment of biological pathways but was characterized by a significant overrepresentation of regulatory regions known to be inversely correlated with gene expression (*i.e.*, gene promoters, and first exons) [11, 37, 38], and a depletion of gene-body regions, where a positive correlation between DNA methylation and active transcription has been described [11, 39]. This non-random hypermethylation pattern is opposite of the relative enrichment of nucleosomes with H3K4me3, which is prominent in TSS of actively transcribed loci [10–12, 40]. Based on the key role of KMT2B in H3K4me3, our findings provide new evidence that dysfunctional KMT2B in DYT28 causes specific DNA hypermethylation of promoters and other regulatory regions positively controlling gene expression, which collectively points out toward a general repression of transcriptional activity in DYT28. This finding has potentially relevant implications in terms of therapy, as it points to the use of nonspecific DNA methyltransferase inhibitors (*e.g.*, 5-aza-2’-deoxycytidine) or small molecules specifically targeting KDM5 demethylases (*e.g.*, CPI-455), as a potential approach to restore proper DNA methylation levels in DYT28 [41].

## Conclusions

In summary, we demonstrate evidence of a distinct DNA methylation episignature associated with *KMT2B*-related DYT28, enabling accurate diagnosis and reclassification of ambiguous genetic findings. We also provide insights into the molecular pathophysiology of this disorder, documenting that *KMT2B* haploinsufficiency causes specific DNA hypermethylation of promoters and other regulatory regions positively controlling gene expression, pointing to the use of DNA methyltransferase inhibitors or molecules targeting KDM5 demethylases as potential therapeutic approaches in DYT28.

## Supplementary Information

**Additional file 1: Figure S1**. Chromatograms showing the *KMT2B* variants identified in the 18 patients included in the study.

**Additional file 2: Figure S2**. Volcano plot of differences in the methylation status of individual probes between patients carrying pathogenic *KMT2B* variants and controls versus statistical significance (-log p-value) of individual probes. Red dots represent selected, significant differentially methylated probes (DMPs) in Pt. 1-8. Positive and negative mean methylation difference show hypermethylation and hypomethylation, respectively.

**Additional file 3: Figure S3**. Mean methylation difference between patients carrying pathogenic *KMT2B* variants and control samples versus individual probes.

**Additional file 4: Figure S4**. Leave-1-out cross validation carried out by means of MDS plots based on the episignature analysis. For each round of validation, seven of the eight samples with bona fide pathogenic *KMT2B* variants were used for probe selection along with control samples and the one remaining was saved for testing. MDS was used to cluster the samples. Each time, the testing sample clustered with the other *KMT2B* mutated samples.

**Additional file 5: Figure S5**. DYT28 episignature identification excluding samples with missense *KMT2B* variants. (A) Hierarchical clustering with Ward’s method on Euclidean distance was performed. In the heatmap plot, each row illustrates a selected CpG site, and each column depicts a sample. The heatmap color scale indicates the range of methylation level; from blue (no methylation or 0) to red (full methylation or 1). The detected episignature clearly differentiates between samples with pathogenic *KMT2B* variants and controls. (B) The first two dimensions of a MDS plot using the selected probes separate the samples with pathogenic variants in *KMT2B* from control samples. Blue circles represent control subjects and red circles indicate subjects with pathogenic *KMT2B* variants and a confirmed diagnosis of DYT28. Ellipses indicate 95% confidence interval.

**Additional file 6: Figure S6**. DNA methylation pattern analysis excluding samples with missense *KMT2B* variants yields full sensitivity and specificity in classifying individuals affected by DYT28. Hierarchical clustering analysis (A) and MDS (B) plots are used to classify VUS/and likely pathogenic missense variants (used as the testing set) with respect to pathogenic *KMT2B* variants in Pt.1-4,6,7 and control samples (used as the training set). (C) A support vector machine (SVM) was used to classify samples and calculate probability scores reaching full sensitivity and specificity for identifying pathogenic *KMT2B* variants. The classifier was trained using bona fide pathogenic *KMT2B* variants, controls and other NDDs/RDs. 75% of controls and NDD/RD samples used for training (blue), 25% for testing (grey). Ellipses indicate 95% confidence intervals.

**Additional file 7: Figure S7**. Differentially methylated regions (DMRs) in DYT28. For each significant differentially methylated genomic region, the plot displays methylation levels calculated for 8 pathogenic variants (Pt. 1-8), used to define the episignature, versus 56 control samples. Mean difference, along with statistically significance according to Fisher’s and Stouffer’s methods, are reported for each region.

**Additional file 8: Figure S8**. DMRs methylation levels distribution throughout different genomic regions. Histograms show the DNA methylation levels (as beta values) for different genomic regions (TSS1500, TSS200, 5’UTR, 1stExon, Body, 3’UTR, IGR) in all the probes contained in DYT28’s DMRs. Blue track displays median beta values in DYT28 (Pt.1-8); red track, median values for controls; black tracks, subjects with pathogenic *KMT2B* variants; grey tracks, individuals with *KMT2B* VUS not related to DYT28. The numbers on the right show the percentage of CpG probes having a beta value more similar to the median in DYT28 patients compared to controls, calculated for each patient *i* as *ABS*(*B*_*i*_ – *control median*) – *ABS*(*B*_*i*_ –* DYT28 median*), where *ABS* is the absolute value of the beta difference for the inspected probe. “TSS” indicates transcription start sites, “IGR” indicates intergenic regions, “Body” indicates genomic regions encompassing gene bodies.

**Additional file 9: Table S1**. Clinical characterization of the study cohort.

**Additional file 10: Table S2**. Sex and age of the patient and control groups used in the episignature discovery analysis.

**Additional file 11: Table S3**. Probes defining the methylation episignature associated with DYT28-causing KMT2B variants.

**Additional file 12: Table S4**. Regions showing differential methylation in DYT28.

## Data Availability

Clinical data of the study cohort, probes defining the methylation episignature associated with DYT28-causing KMT2B variants and list of regions differentially methylated in DYT28 are reported in additional files 8–11. Additional data are available from the corresponding authors upon request.

## Abbreviations

ACMG/AMP: American College of Medical Genetics and Genomics/Association for Molecular Pathology
CNS: Central nervous system
DOPA: 3,4-Dihydroxyphenylalanine
DMP: Differentially methylated probe
DMR: Differentially methylated region
DNAm: DNA methylation
DYT28: Dystonia 28
EKD: EpiSign knowledge database
FDR: False discovery rate
MDS: Multidimensional scaling
NDD: Neurodevelopmental disorders
PCA: Principal component analysis
RD: Rare diseases
VUS: Variant(s) of uncertain significance
