# Supplementary figures and images for: Childhood-onset dystonia-causing KMT2B variants result in a distinctive genomic hypermethylation profile
Source: Clin Epigenetics. 2021 Aug 11;13:157. doi: 10.1186/s13148-021-01145-y (PMC8359374; doi:10.1186/s13148-021-01145-y)

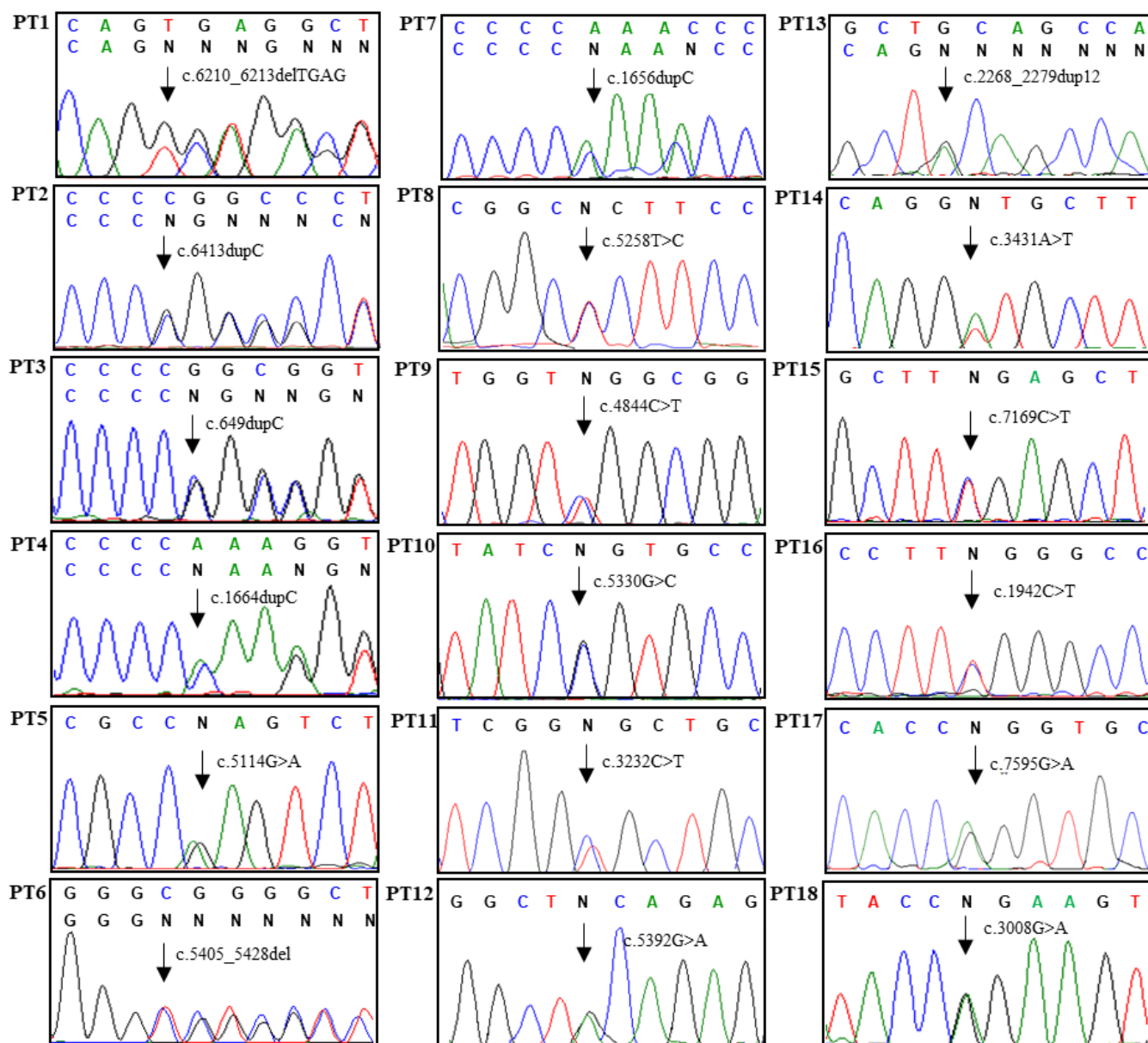

Supplement: Supplementary file 1 — Additional file 1: Figure S1. Chromatograms showing the KMT2B variants identified in the 18 patients included in the study. [file 13148_2021_1145_MOESM1_ESM.pdf]

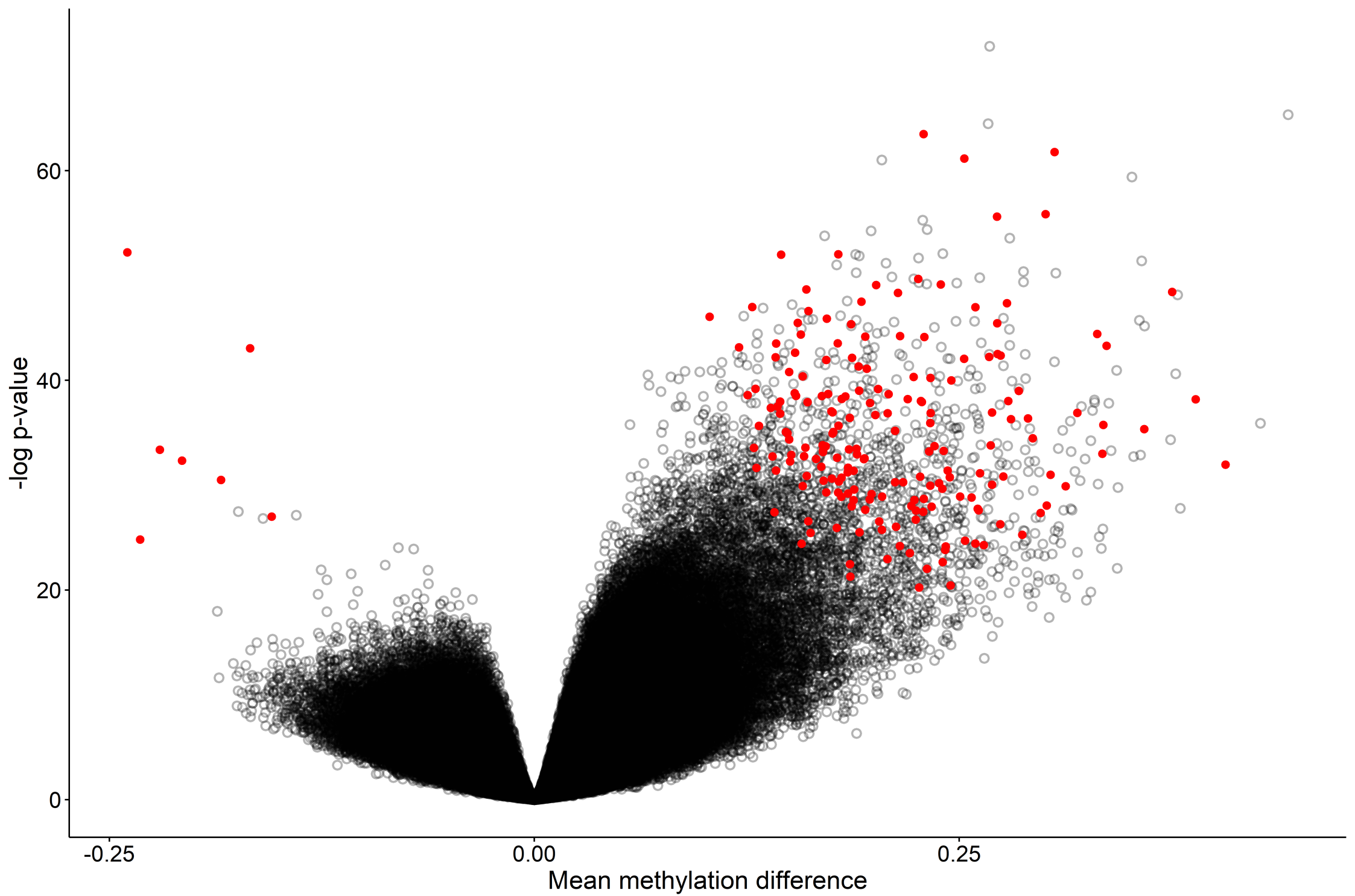

Supplement: Supplementary file 2 — Additional file 2: Figure S2. Volcano plot of differences in the methylation status of individual probes between patients carrying pathogenic KMT2B variants and controls versus statistical significance (-log p-value) of individual probes. Red dots represent selected, significant differentially methylated probes (DMPs) in Pt. 1-8. Positive and negative mean methylation difference show hypermethylation and hypomethylation, respectively. [file 13148_2021_1145_MOESM2_ESM.pdf]

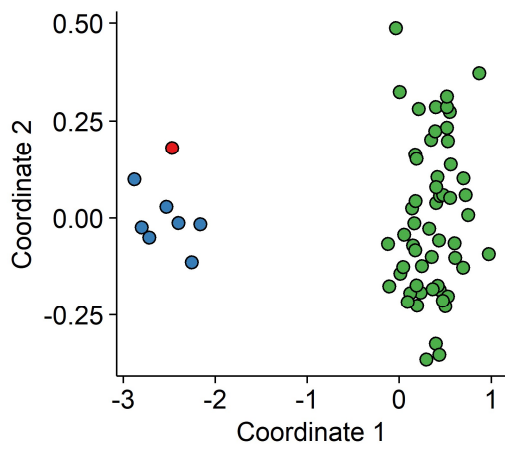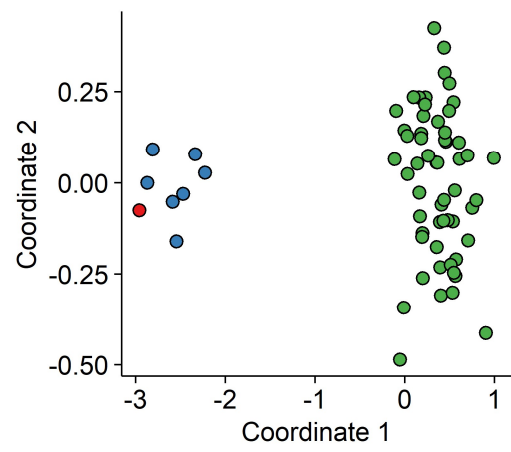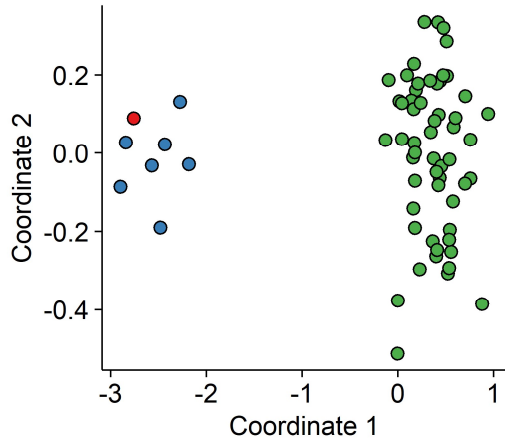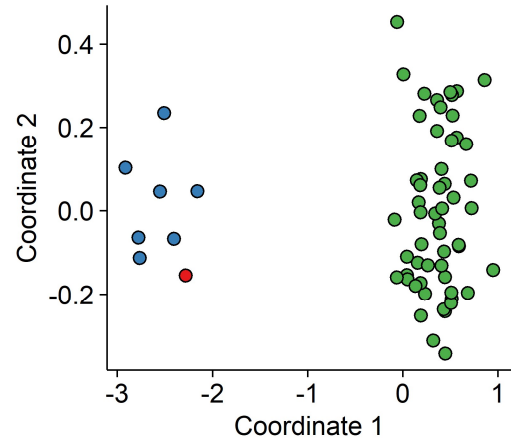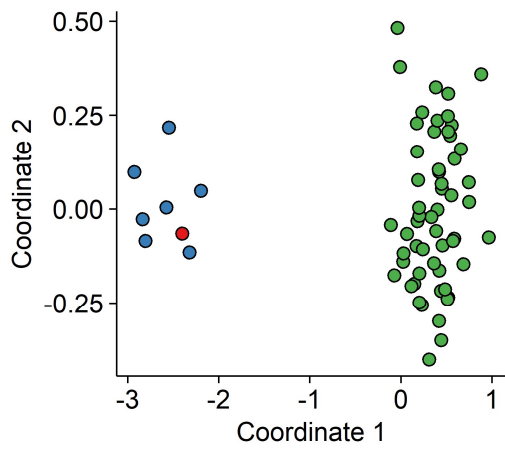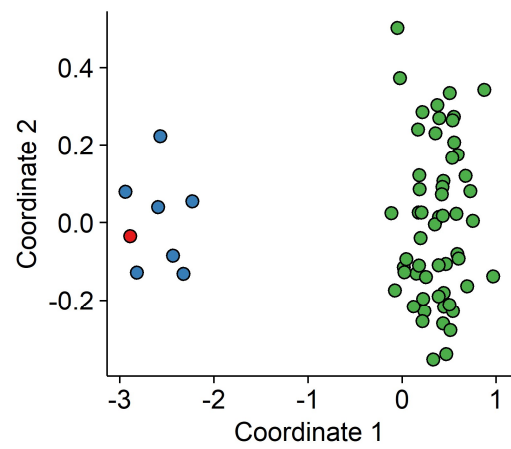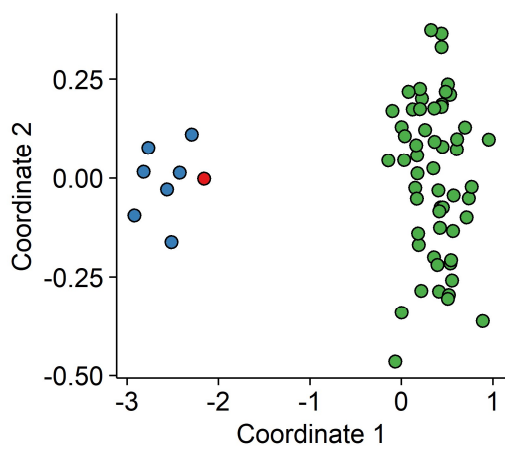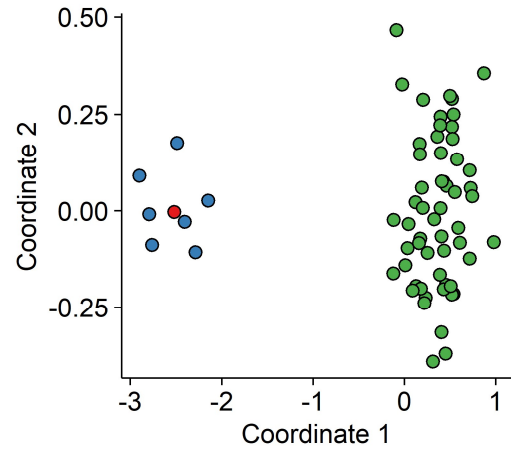

Supplement: Supplementary file 4 — Additional file 4: Figure S4. Leave-1-out cross validation carried out by means of MDS plots based on the episignature analysis. For each round of validation, seven of the eight samples with bona fide pathogenic KMT2B variants were used for probe selection along with control samples and the one remaining was saved for testing. MDS was used to cluster the samples. Each time, the testing sample clustered with the other KMT2B mutated samples. [file 13148_2021_1145_MOESM4_ESM.pdf]

**A**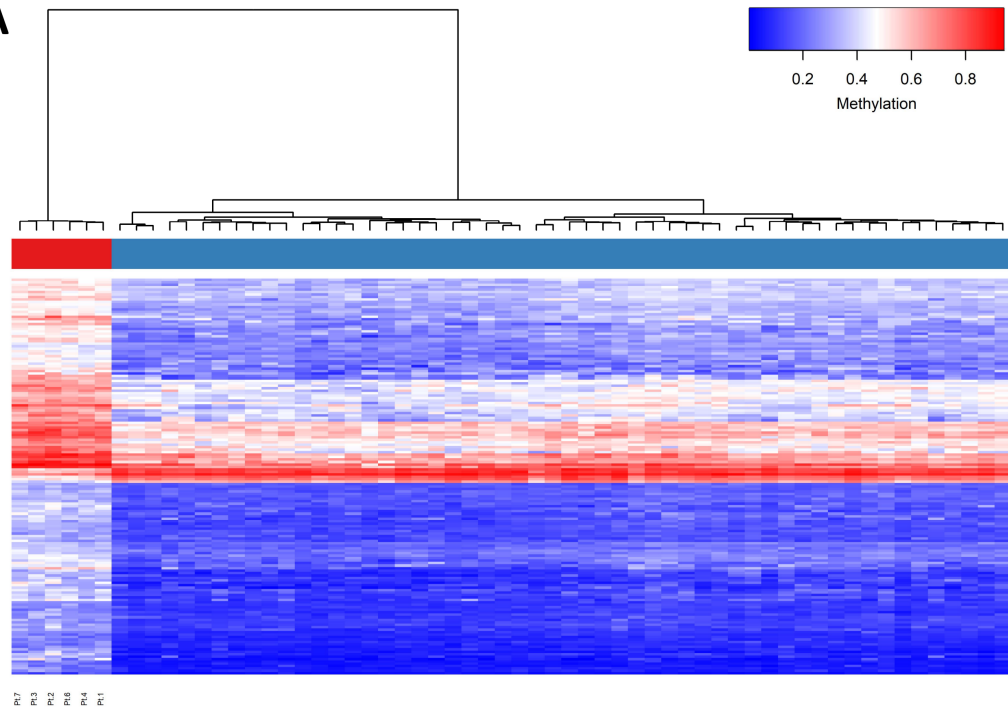**B**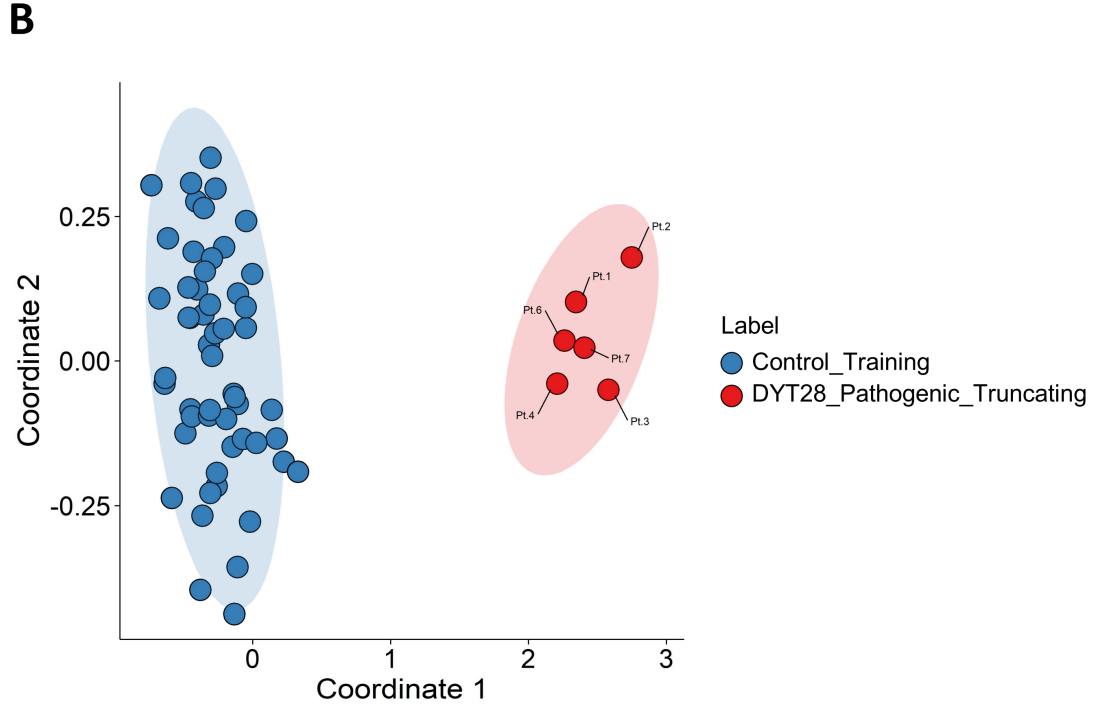

Supplement: Supplementary file 5 — Additional file 5: Figure S5. DYT28 episignature identification excluding samples with missense KMT2B variants. (A) Hierarchical clustering with Ward’s method on Euclidean distance was performed. In the heatmap plot, each row illustrates a selected CpG site, and each column depicts a sample. The heatmap color scale indicates the range of methylation level; from blue (no methylation or 0) to red (full methylation or 1). The detected episignature clearly differentiates between samples with pathogenic KMT2B variants and controls. (B) The first two dimensions of a MDS plot using the selected probes separate the samples with pathogenic variants in KMT2B from control samples. Blue circles represent control subjects and red circles indicate subjects with pathogenic KMT2B variants and a confirmed diagnosis of DYT28. Ellipses indicate 95% confidence interval. [file 13148_2021_1145_MOESM5_ESM.pdf]

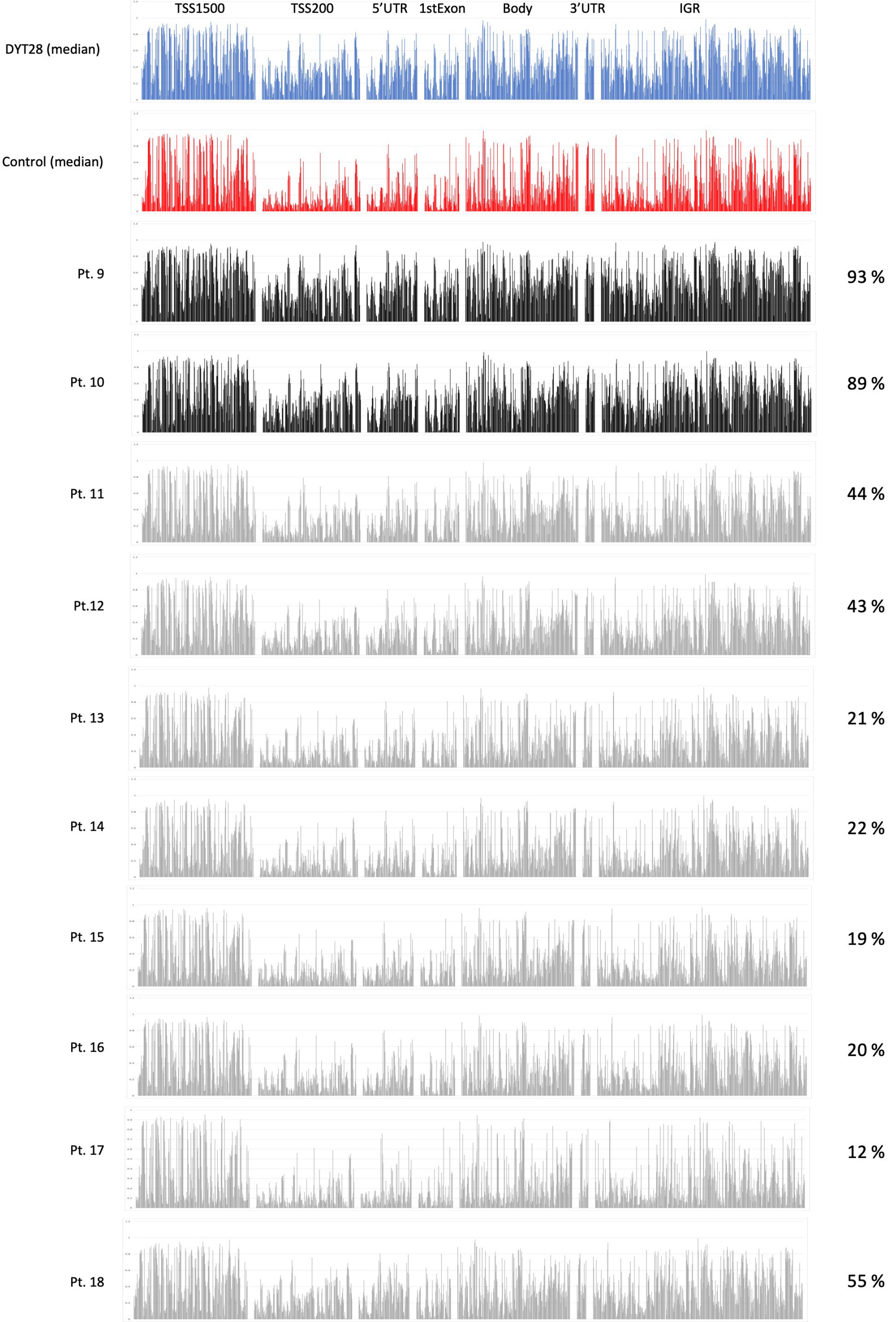

Supplement: Supplementary file 8 — Additional file 8: Figure S8. DMRs methylation levels distribution throughout different genomic regions. Histograms show the DNA methylation levels (as beta values) for different genomic regions (TSS1500, TSS200, 5’UTR, 1stExon, Body, 3’UTR, IGR) in all the probes contained in DYT28’s DMRs. Blue track displays median beta values in DYT28 (Pt.1-8); red track, median values for controls; black tracks, subjects with pathogenic KMT2B variants; grey tracks, individuals with KMT2B VUS not related to DYT28. The numbers on the right show the percentage of CpG probes having a beta value more similar to the median in DYT28 patients compared to controls, calculated for each patient i as ABS(Bi – control median) – ABS(Bi – DYT28 median), where ABS is the absolute value of the beta difference for the inspected probe. “TSS” indicates transcription start sites, “IGR” indicates intergenic regions, “Body” indicates genomic regions encompassing gene bodies. [file 13148_2021_1145_MOESM8_ESM.pdf]
