# Supplementary material for: Childhood-onset dystonia-causing KMT2B variants result in a distinctive genomic hypermethylation profile
Source: Clin Epigenetics. 2021 Aug 11;13:157. doi: 10.1186/s13148-021-01145-y (PMC8359374; doi:10.1186/s13148-021-01145-y)
Supplement: Supplementary file 3 — Additional file 3: Figure S3. Mean methylation difference between patients carrying pathogenic KMT2B variants and control samples versus individual probes. [file 13148_2021_1145_MOESM3_ESM.pdf]

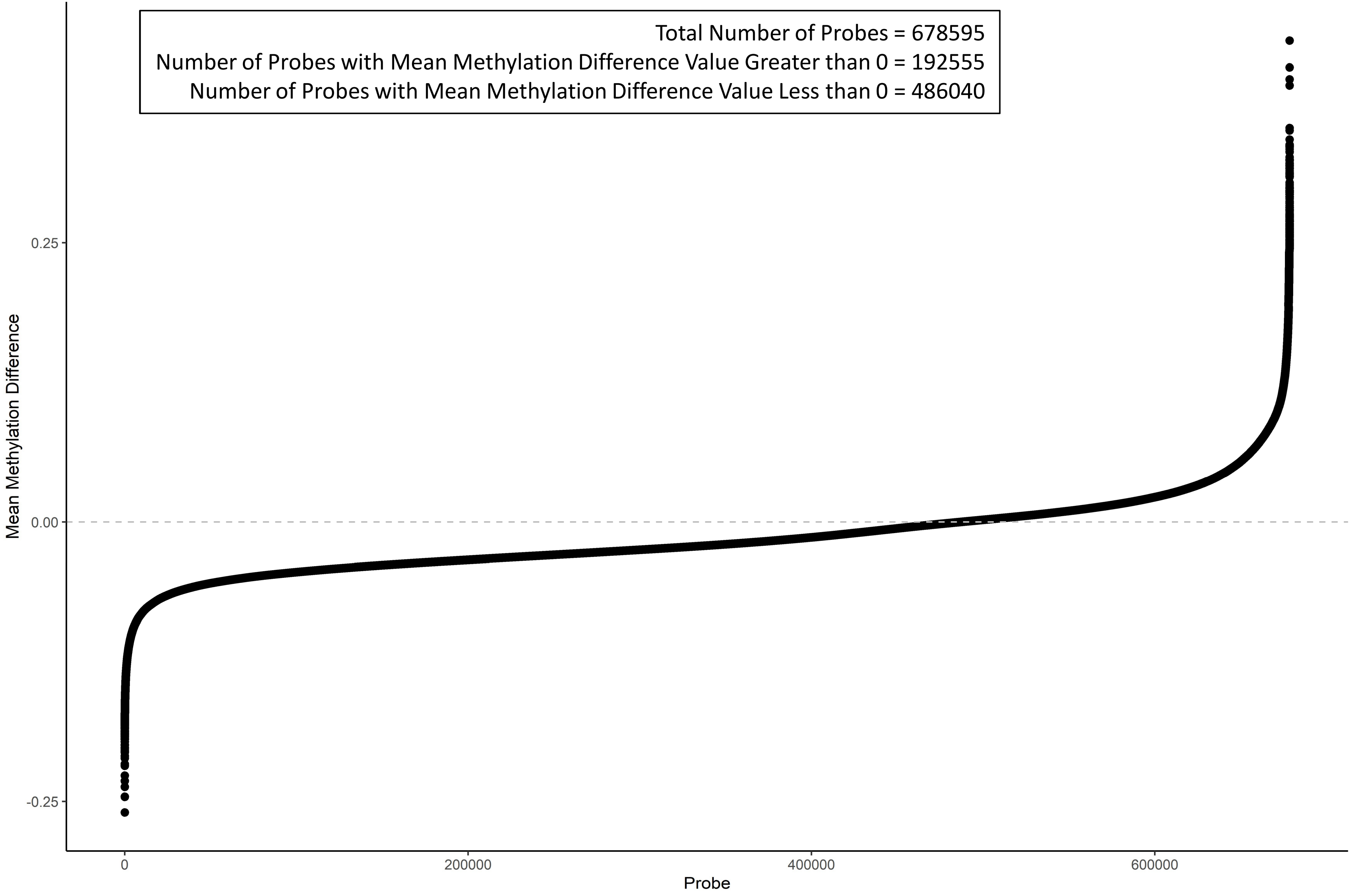
Total Number of Probes = 678595
Number of Probes with Mean Methylation Difference Value Greater than 0 = 192555
Number of Probes with Mean Methylation Difference Value Less than 0 = 486040
Mean Methylation Difference
0.25
0.00
-0.25
0
200000
400000
600000
Probe
