# Supplementary material for: Childhood-onset dystonia-causing KMT2B variants result in a distinctive genomic hypermethylation profile
Source: Clin Epigenetics. 2021 Aug 11;13:157. doi: 10.1186/s13148-021-01145-y (PMC8359374; doi:10.1186/s13148-021-01145-y)
Supplement: Supplementary file 6 — Additional file 6: Figure S6. DNA methylation pattern analysis excluding samples with missense KMT2B variants yields full sensitivity and specificity in classifying individuals affected by DYT28. Hierarchical clustering analysis (A) and MDS (B) plots are used to classify VUS/and likely pathogenic missense variants (used as the testing set) with respect to pathogenic KMT2B variants in Pt.1-4,6,7 and control samples (used as the training set). (C) A support vector machine (SVM) was used to classify samples and calculate probability scores reaching full sensitivity and specificity for identifying pathogenic KMT2B variants. The classifier was trained using bona fide pathogenic KMT2B variants, controls and other NDDs/RDs. 75% of controls and NDD/RD samples used for training (blue), 25% for testing (grey). Ellipses indicate 95% confidence intervals. [file 13148_2021_1145_MOESM6_ESM.pdf]

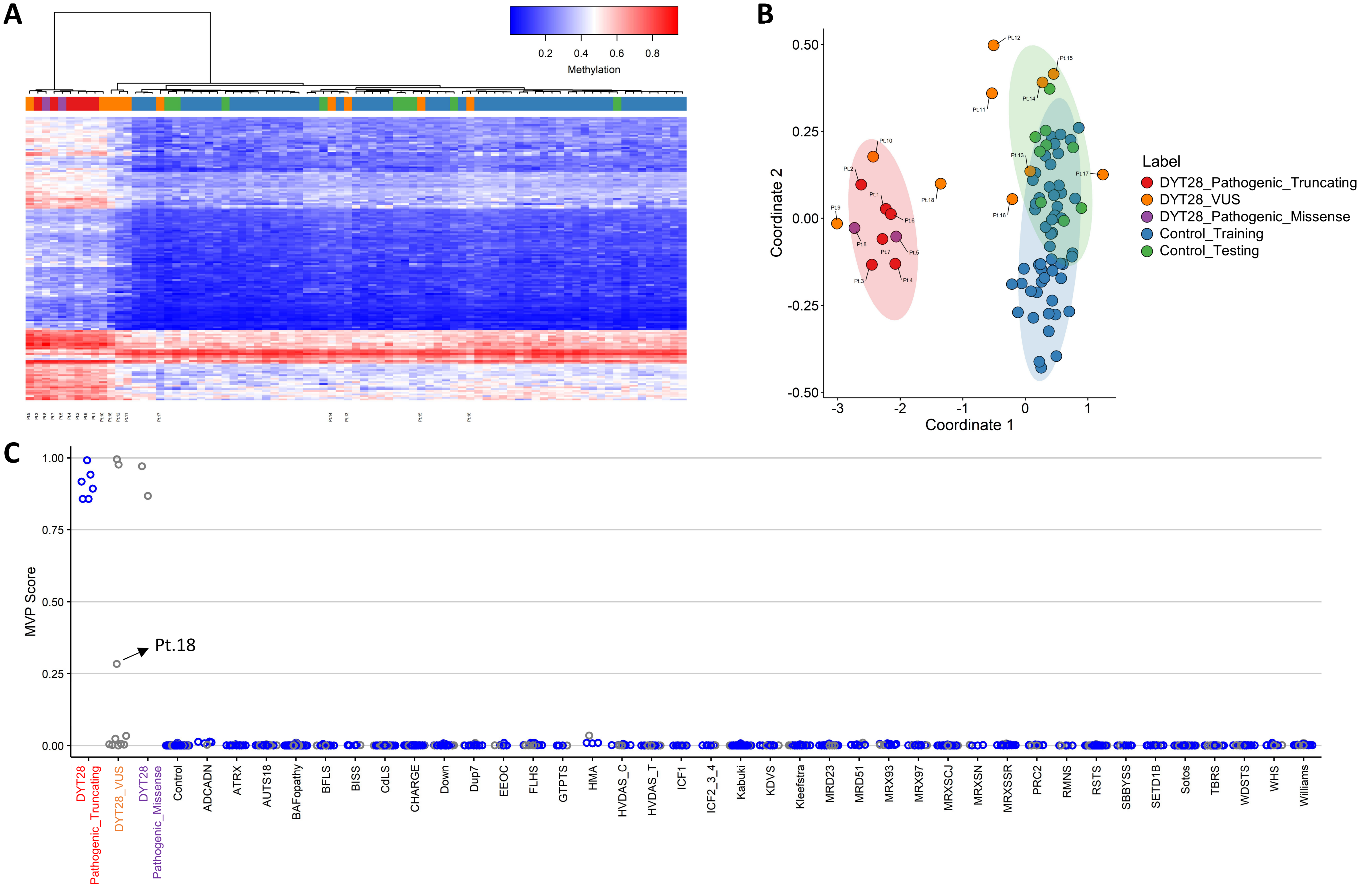
A
0.2 0.4 0.6 0.8
Methylation
B
Label
DYT28_Pathogenic_Truncating
DYT28_VUS
DYT28_Pathogenic_Missense
Control_Training
Control_Testing
Coordinate 1
Coordinate 2
0.50 0.25 0.00 -0.25 -0.50
-3 -2 -1 0 1
Pt.1 Pt.2 Pt.3 Pt.4 Pt.5 Pt.6 Pt.7 Pt.8 Pt.9 Pt.10 Pt.11 Pt.12 Pt.13 Pt.14 Pt.15 Pt.16 Pt.17 Pt.18
C
MVP Score
1.00 0.75 0.50 0.25 0.00
Pt.18
DYT28 Pathogenic_Truncating
DYT28_VUS
DYT28 Pathogenic_Missense
Control
ADCADN
ATRX
AUTS18
BAFopathy
BFLS
BISS
CdLS
CHARGE
Down
Dup7
EEOC
FLHS
GTPTS
HMA
HVDAS_C
HVDAS_T
ICF1
ICF2_3_4
Kabuki
KDVS
Kleefstra
MRD23
MRD51
MRX93
MRX97
MRXSCJ
MRXSN
MRXSSR
PRC2
RMNS
RSTS
SBBYSS
SETD1B
Sotos
TBRS
WDSTS
WHS
Williams
