# Supplementary material for: Childhood-onset dystonia-causing KMT2B variants result in a distinctive genomic hypermethylation profile
Source: Clin Epigenetics. 2021 Aug 11;13:157. doi: 10.1186/s13148-021-01145-y (PMC8359374; doi:10.1186/s13148-021-01145-y)
Supplement: Supplementary file 7 — Additional file 7: Figure S7. Differentially methylated regions (DMRs) in DYT28. For each significant differentially methylated genomic region, the plot displays methylation levels calculated for 8 pathogenic variants (Pt. 1-8), used to define the episignature, versus 56 control samples. Mean difference, along with statistically significance according to Fisher’s and Stouffer’s methods, are reported for each region. [file 13148_2021_1145_MOESM7_ESM.pdf]

Region 1: chr7:95025194−95027751
Fisher: 3.69232183739702e−171
Stouffer: 2.3285624328954e−160
Mean difference: 0.143821508375814

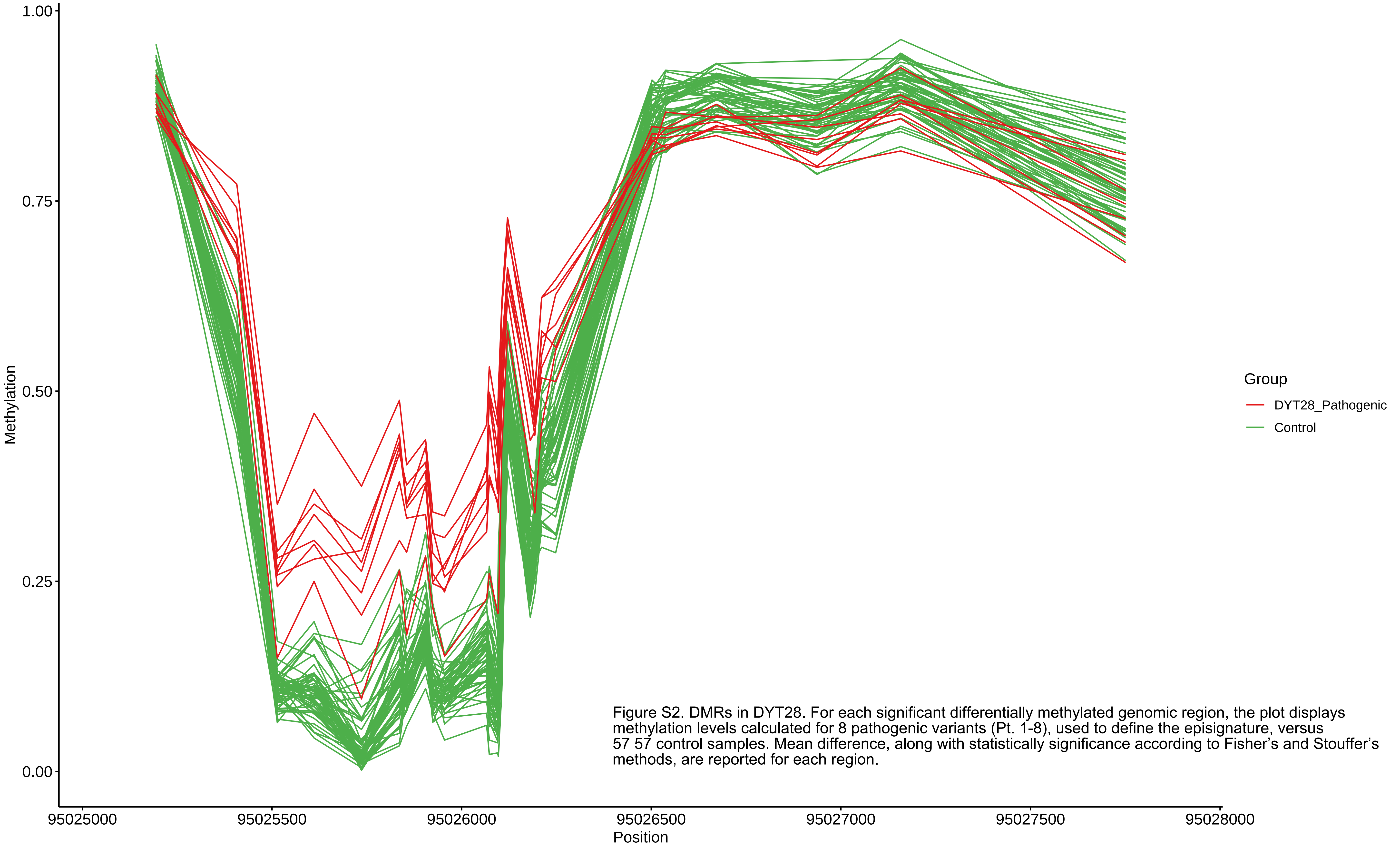

Figure S2. DMRs in DYT28. For each significant differentially methylated genomic region, the plot displays methylation levels calculated for 8 pathogenic variants (Pt. 1-8), used to define the episignature, versus 57 57 control samples. Mean difference, along with statistically significance according to Fisher's and Stouffer's methods, are reported for each region.

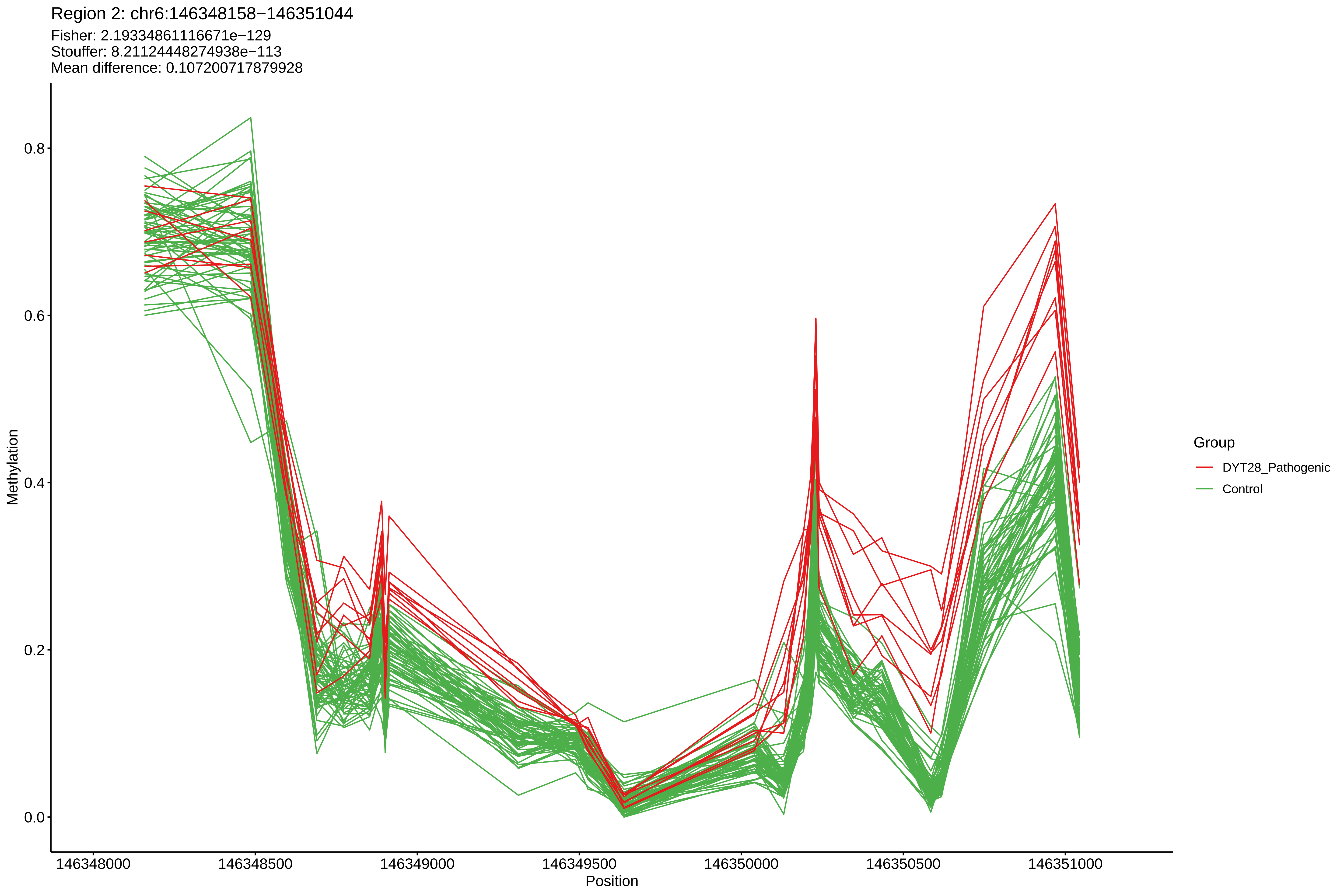

Region 2: chr6:146348158−146351044
Fisher: 2.19334861116671e−129
Stouffer: 8.21124448274938e−113
Mean difference: 0.107200717879928
Methylation
0.0
0.2
0.4
0.6
0.8
Position
146348000
146348500
146349000
146349500
146350000
146350500
146351000
Group
DYT28_Pathogenic
Control

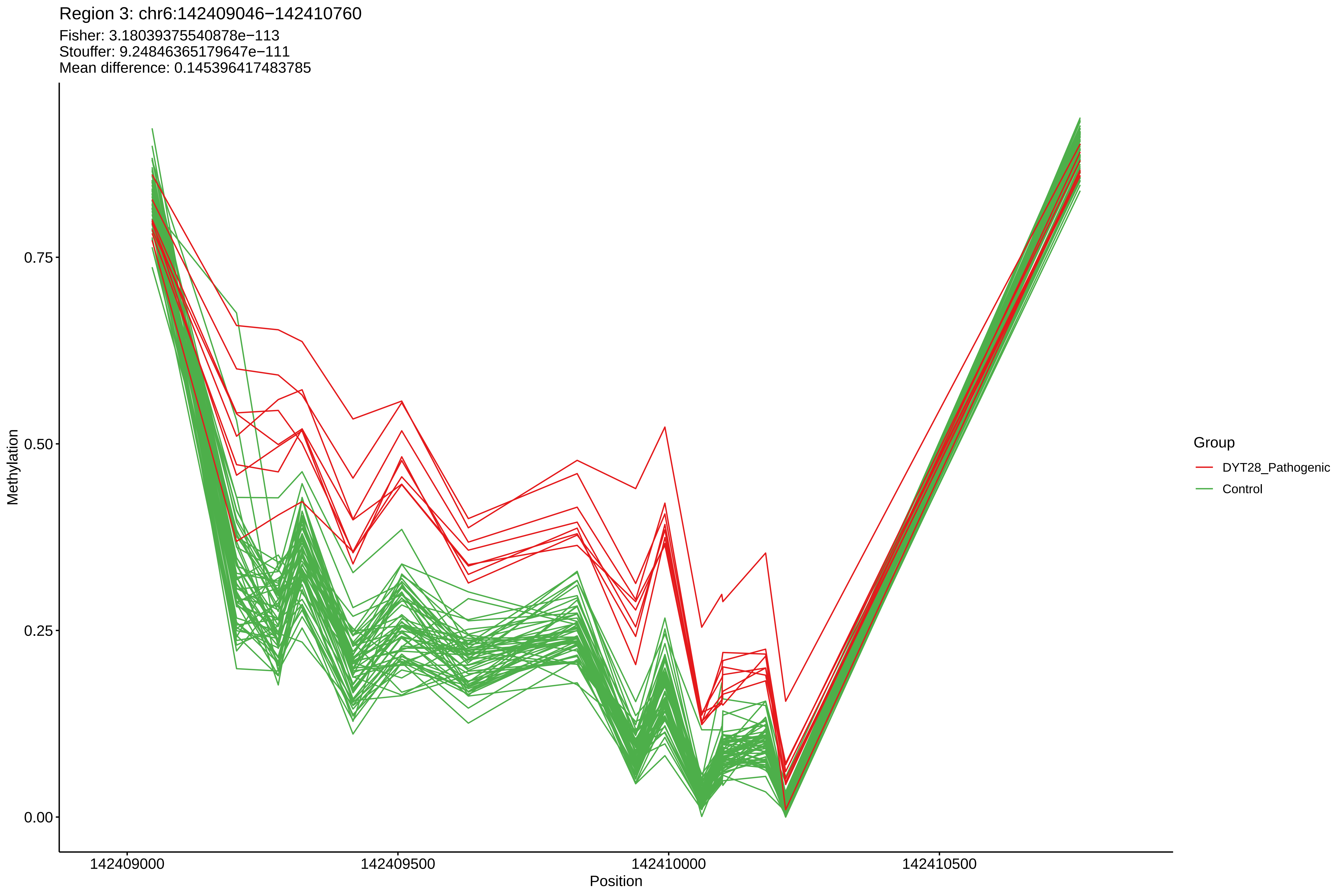

Region 3: chr6:142409046−142410760
Fisher: 3.18039375540878e−113
Stouffer: 9.24846365179647e−111
Mean difference: 0.145396417483785
Methylation
0.75
0.50
0.25
0.00
142409000
142409500
142410000
142410500
Position
Group
DYT28_Pathogenic
Control

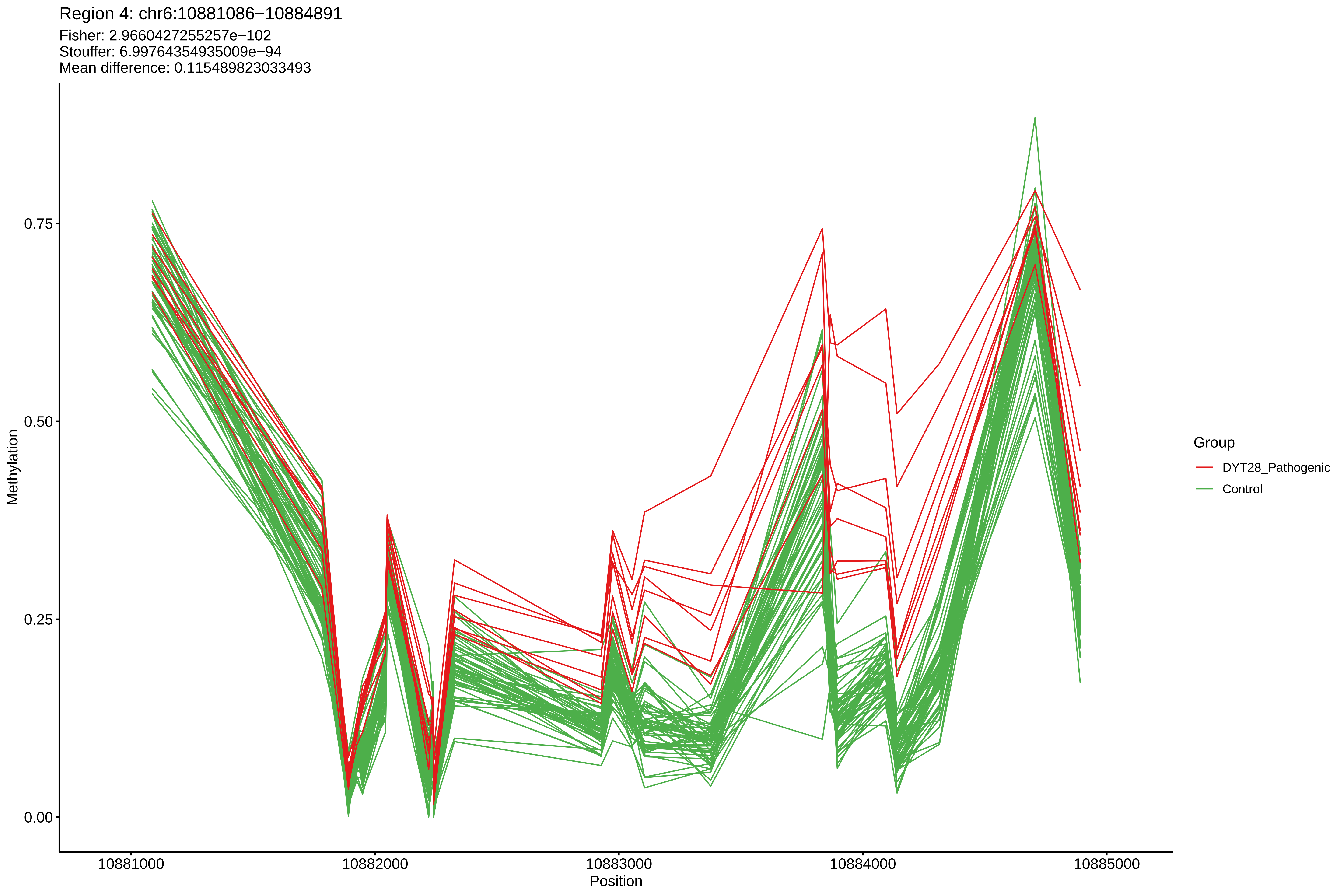
Region 4: chr6:10881086−10884891
Fisher: 2.9660427255257e−102
Stouffer: 6.99764354935009e−94
Mean difference: 0.115489823033493
Methylation
0.75
0.50
0.25
0.00
10881000
10882000
10883000
10884000
10885000
Position
Group
DYT28_Pathogenic
Control

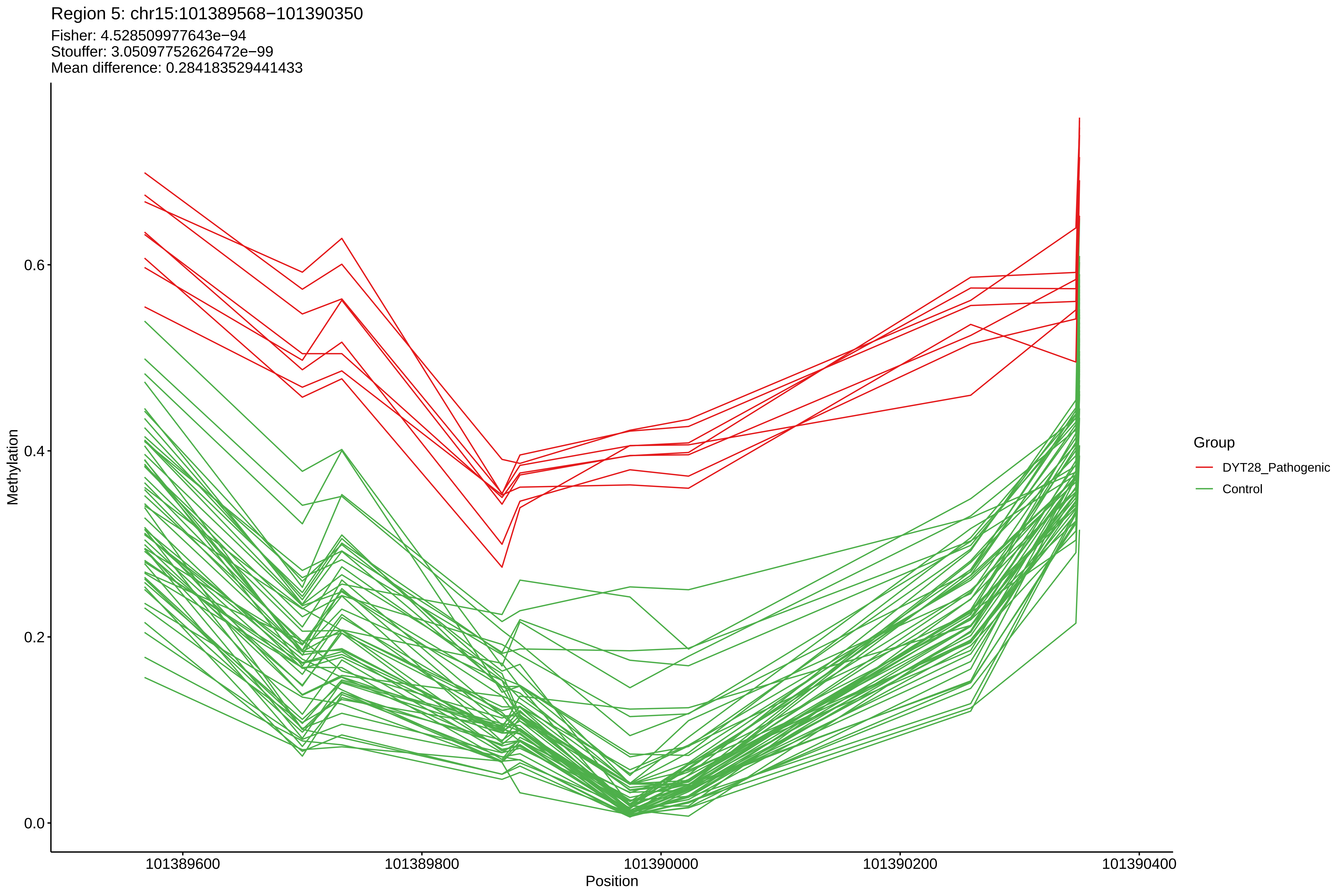

Region 5: chr15:101389568−101390350
Fisher: 4.528509977643e−94
Stouffer: 3.05097752626472e−99
Mean difference: 0.284183529441433
Methylation
0.0
0.2
0.4
0.6
Position
101389600
101389800
101390000
101390200
101390400
Group
DYT28_Pathogenic
Control

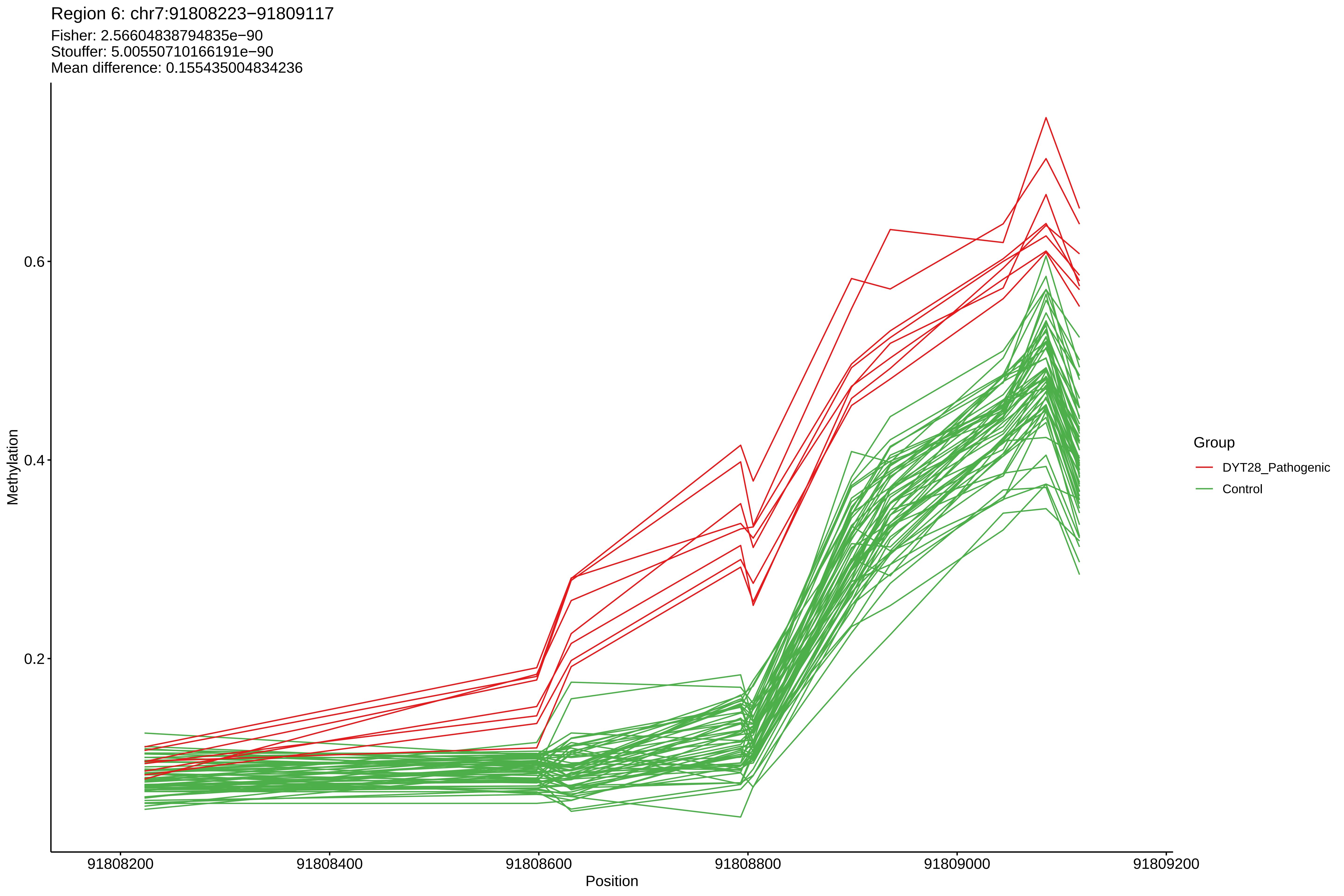
Region 6: chr7:91808223−91809117
Fisher: 2.56604838794835e−90
Stouffer: 5.00550710166191e−90
Mean difference: 0.155435004834236
Methylation
0.2
0.4
0.6
Position
91808200
91808400
91808600
91808800
91809000
91809200
Group
DYT28_Pathogenic
Control

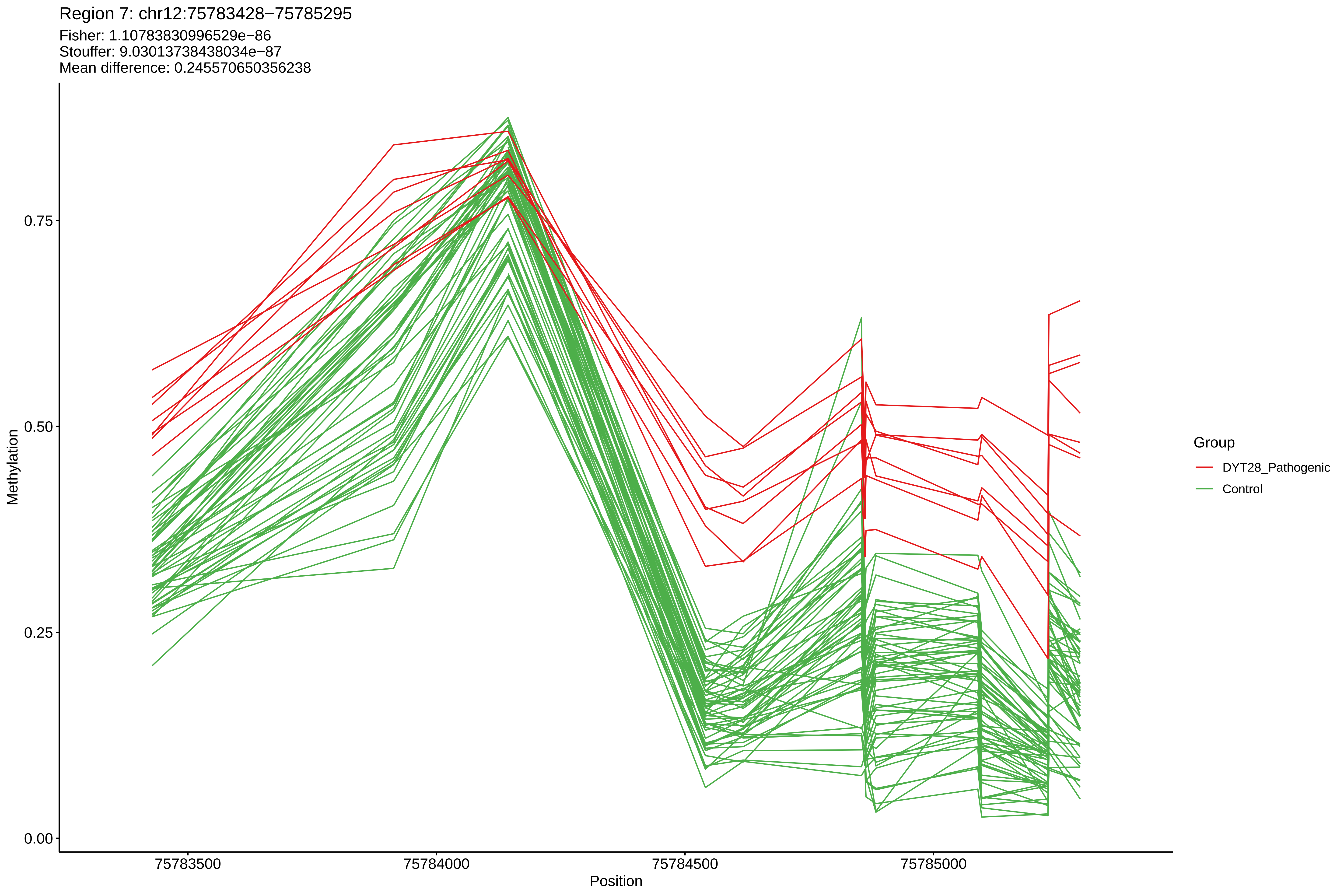
Region 7: chr12:75783428−75785295
Fisher: 1.10783830996529e−86
Stouffer: 9.03013738438034e−87
Mean difference: 0.245570650356238
Methylation
0.00
0.25
0.50
0.75
75783500
75784000
75784500
75785000
Position
Group
DYT28_Pathogenic
Control

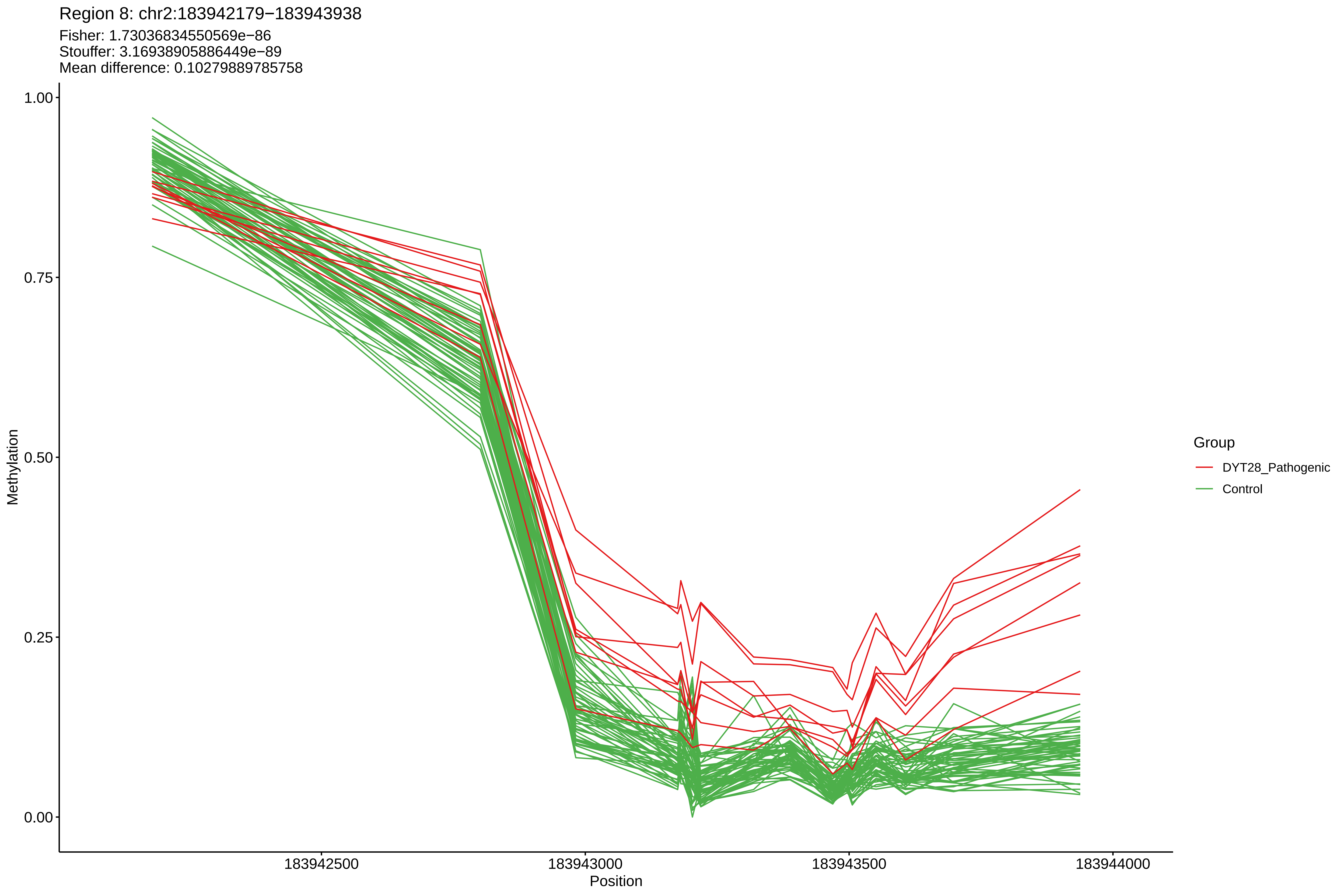
Region 8: chr2:183942179−183943938
Fisher: 1.73036834550569e−86
Stouffer: 3.16938905886449e−89
Mean difference: 0.10279889785758
Methylation
1.00
0.75
0.50
0.25
0.00
183942500
183943000
183943500
183944000
Position
Group
DYT28_Pathogenic
Control

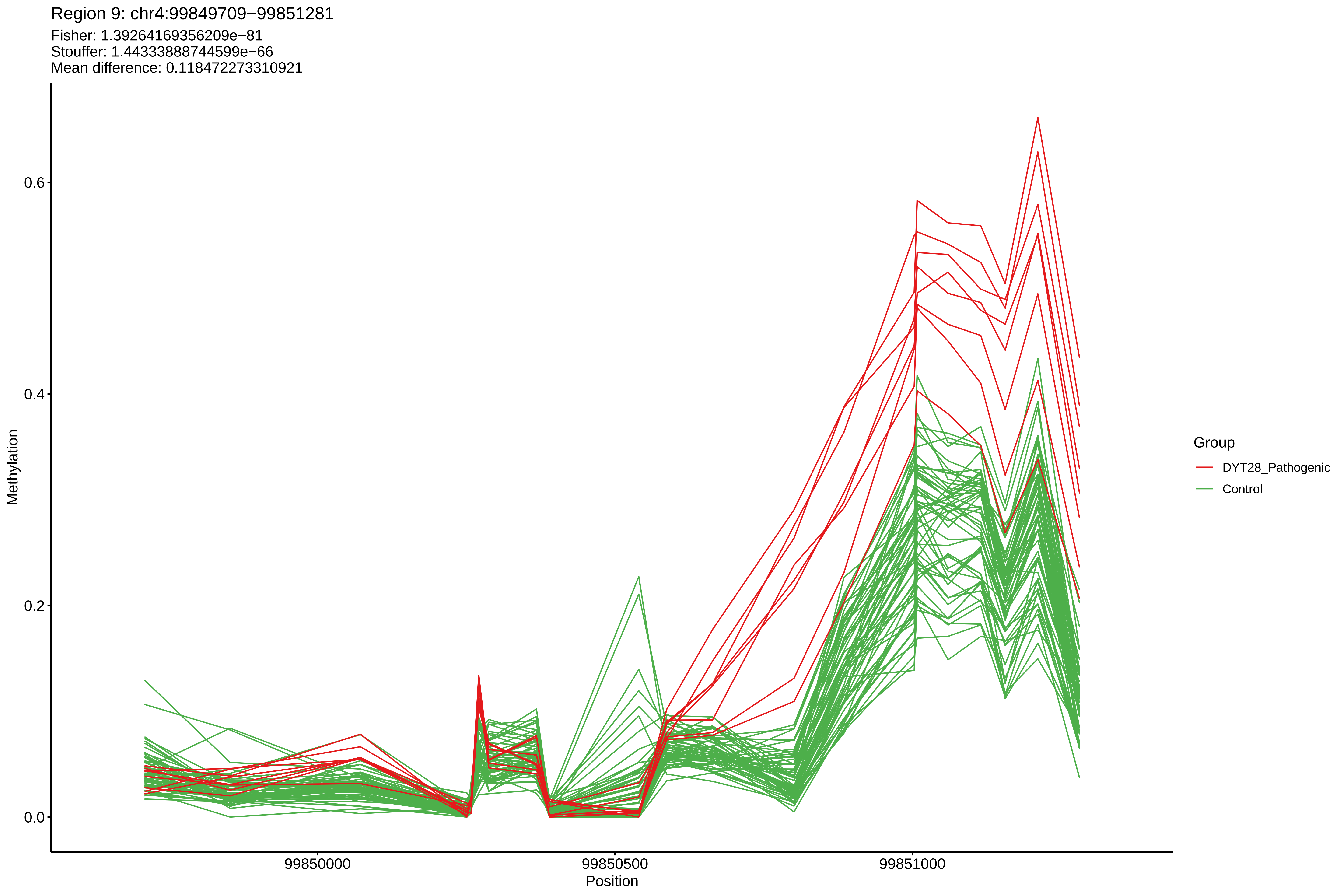
Region 9: chr4:99849709−99851281
Fisher: 1.39264169356209e−81
Stouffer: 1.44333888744599e−66
Mean difference: 0.118472273310921
Methylation
0.0
0.2
0.4
0.6
99850000
99850500
99851000
Position
Group
DYT28_Pathogenic
Control

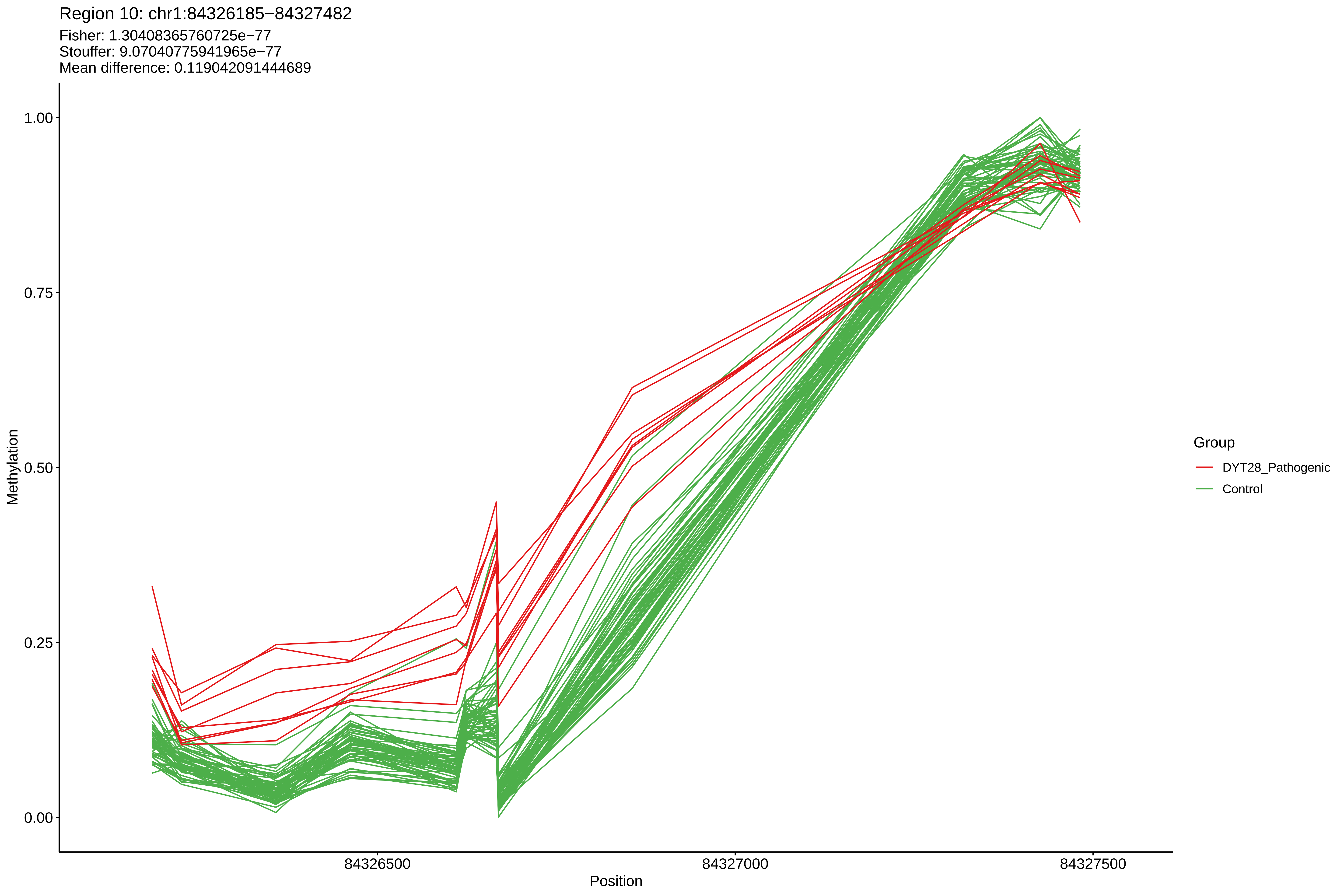

Region 10: chr1:84326185−84327482
Fisher: 1.30408365760725e−77
Stouffer: 9.07040775941965e−77
Mean difference: 0.119042091444689
1.00
0.75
0.50
0.25
0.00
Methylation
84326500
84327000
84327500
Position
Group
DYT28_Pathogenic
Control

Region 11: chr6:28583971−28584464

Fisher: 2.94735809897005e−74
Stouffer: 9.53012645423371e−78
Mean difference: 0.167356205248983

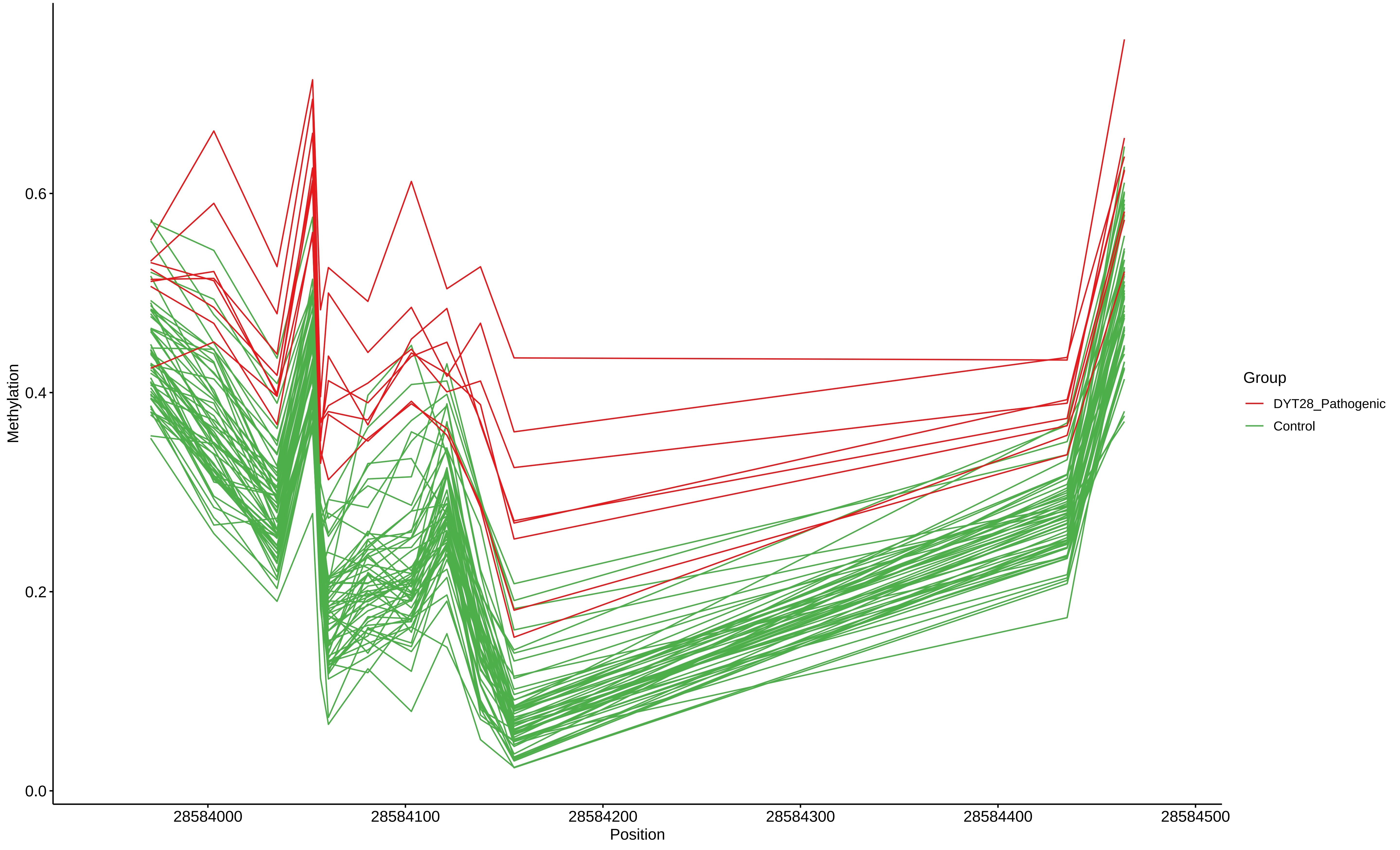

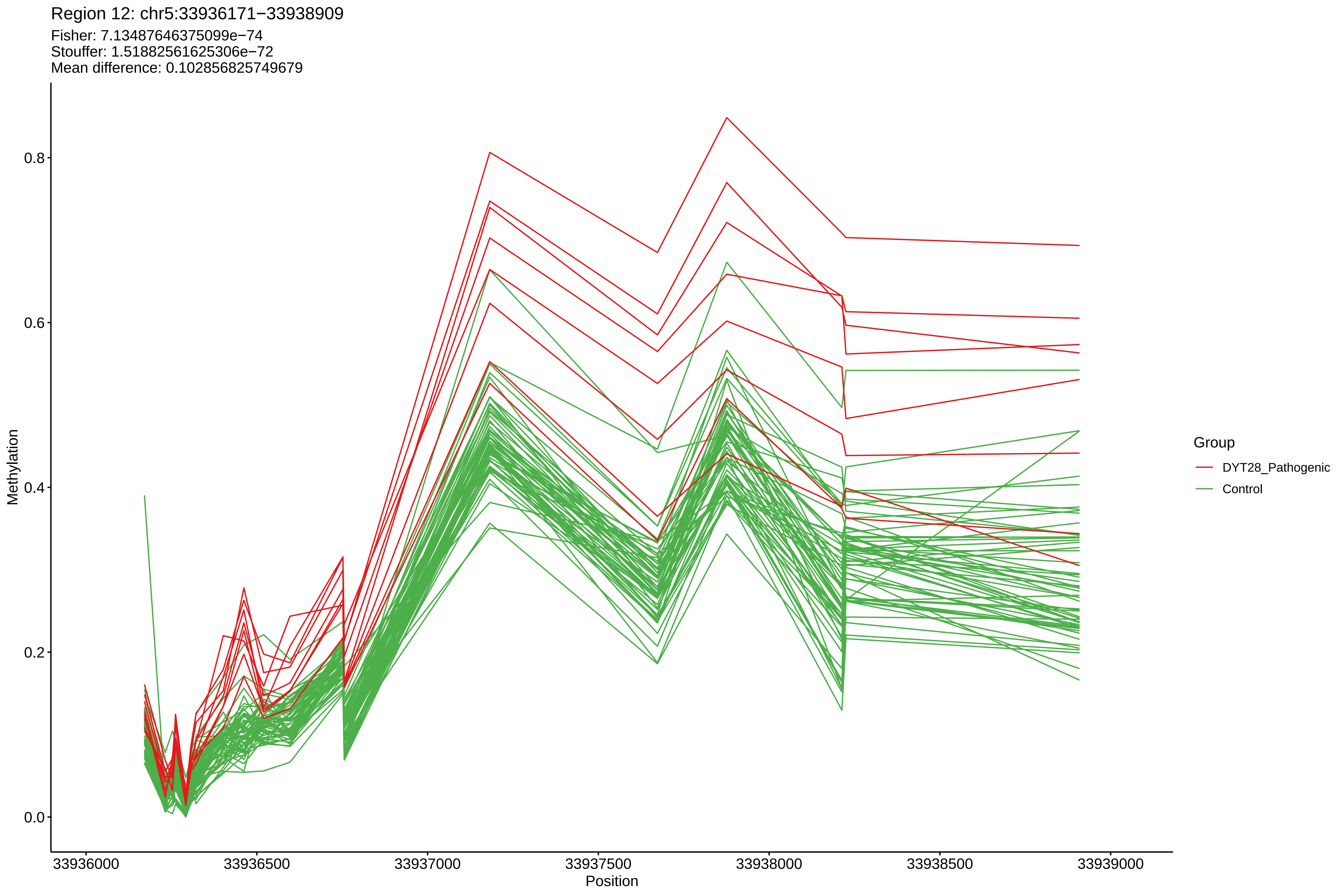

Region 12: chr5:33936171−33938909
Fisher: 7.13487646375099e−74
Stouffer: 1.51882561625306e−72
Mean difference: 0.102856825749679
Methylation
0.0
0.2
0.4
0.6
0.8
33936000
33936500
33937000
33937500
33938000
33938500
33939000
Position
Group
DYT28_Pathogenic
Control

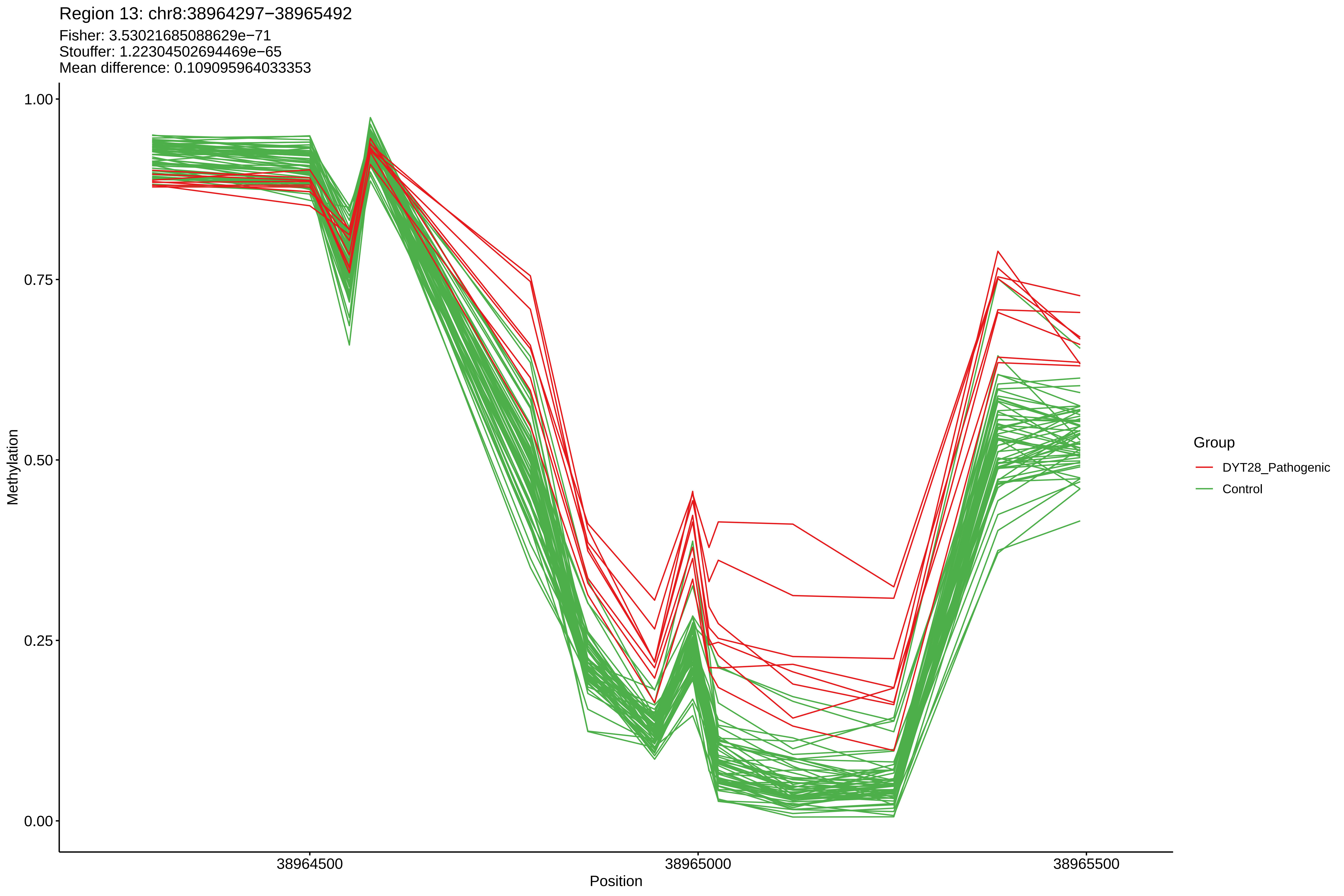
Region 13: chr8:38964297−38965492
Fisher: 3.53021685088629e−71
Stouffer: 1.22304502694469e−65
Mean difference: 0.109095964033353
Methylation
1.00
0.75
0.50
0.25
0.00
38964500
38965000
38965500
Position
Group
DYT28_Pathogenic
Control

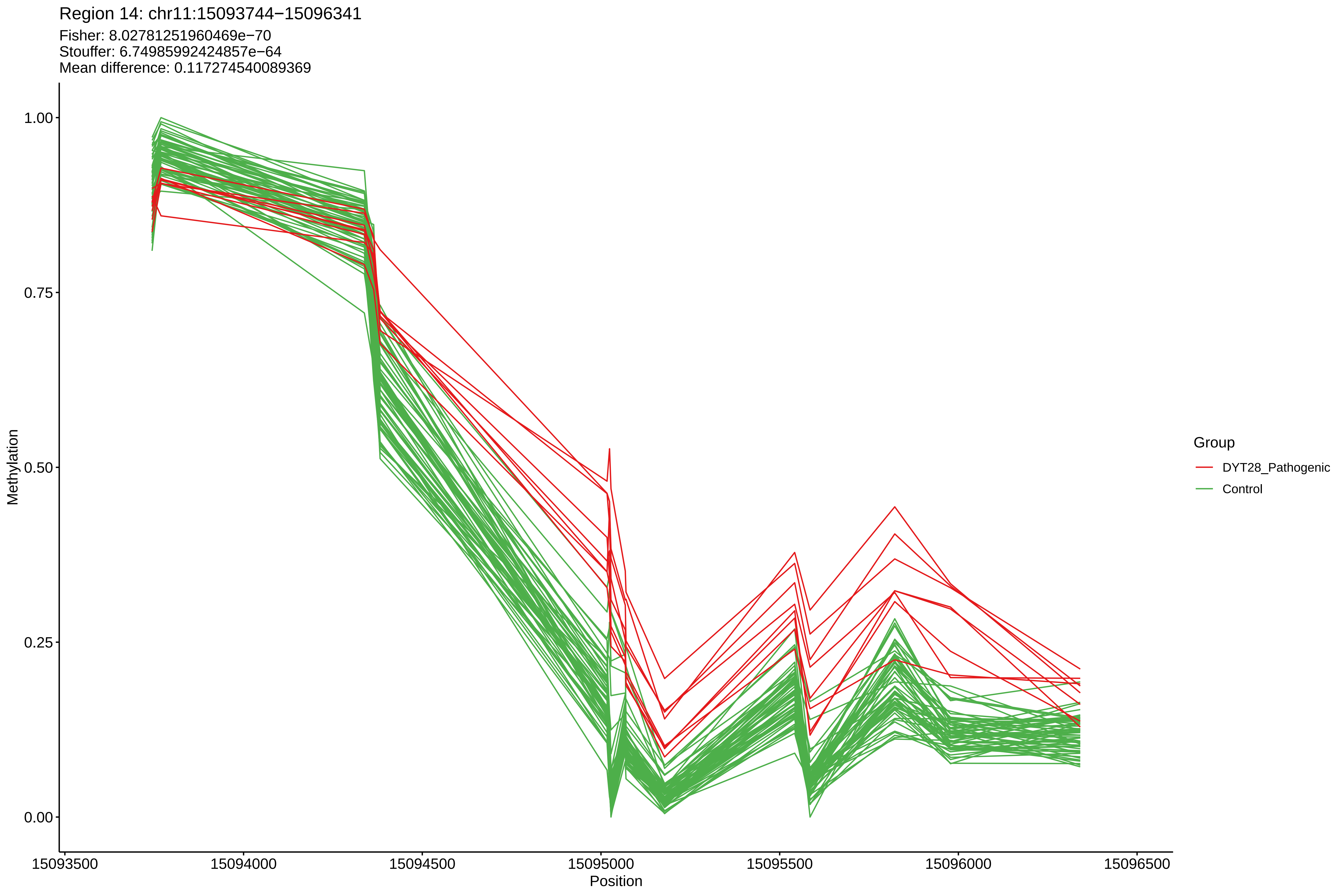
Region 14: chr11:15093744−15096341
Fisher: 8.02781251960469e−70
Stouffer: 6.74985992424857e−64
Mean difference: 0.117274540089369
1.00
0.75
0.50
0.25
0.00
Methylation
15093500
15094000
15094500
15095000
15095500
15096000
15096500
Position
Group
DYT28_Pathogenic
Control

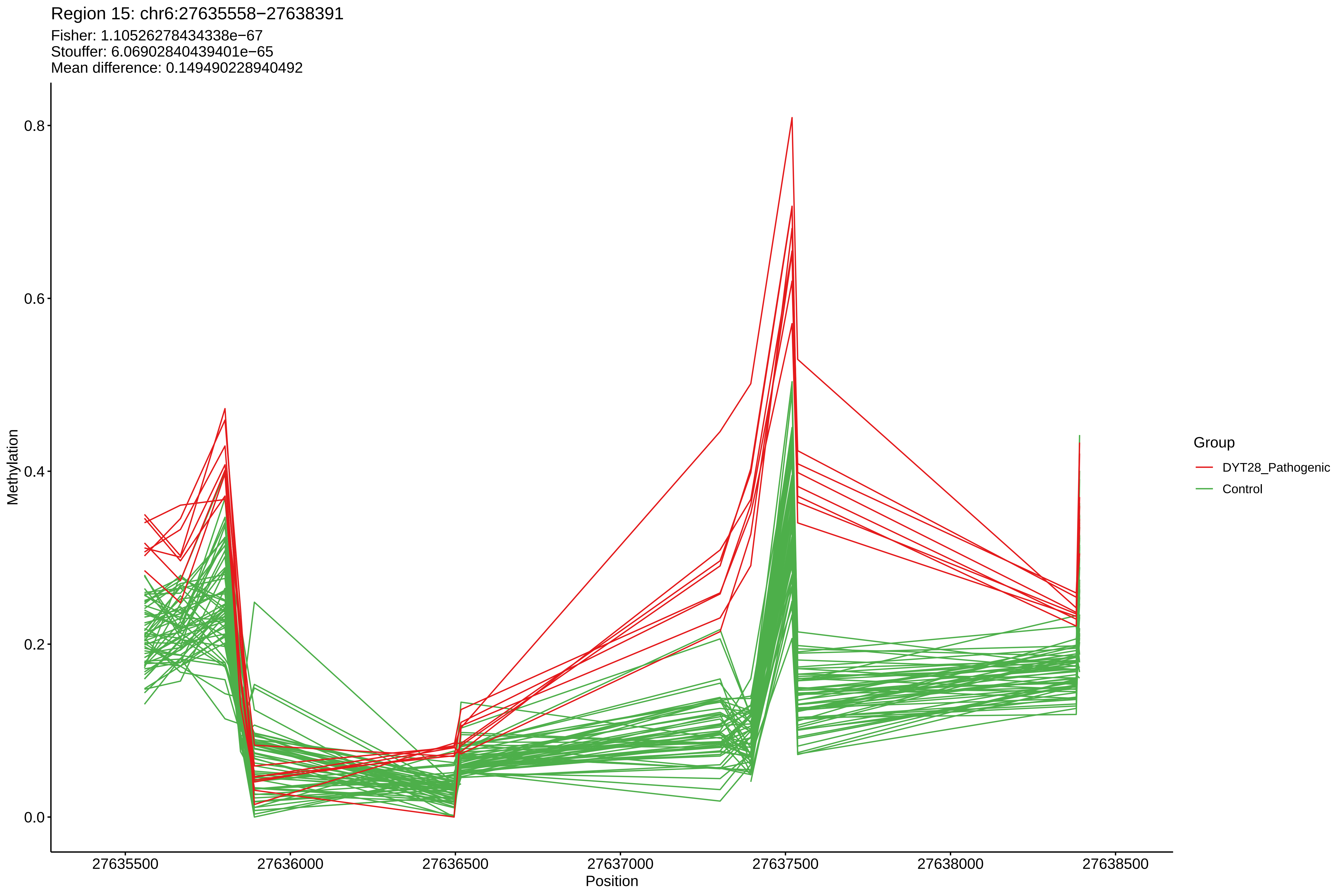
Region 15: chr6:27635558−27638391
Fisher: 1.10526278434338e−67
Stouffer: 6.06902840439401e−65
Mean difference: 0.149490228940492
Methylation
0.0
0.2
0.4
0.6
0.8
27635500
27636000
27636500
27637000
27637500
27638000
27638500
Position
Group
DYT28_Pathogenic
Control

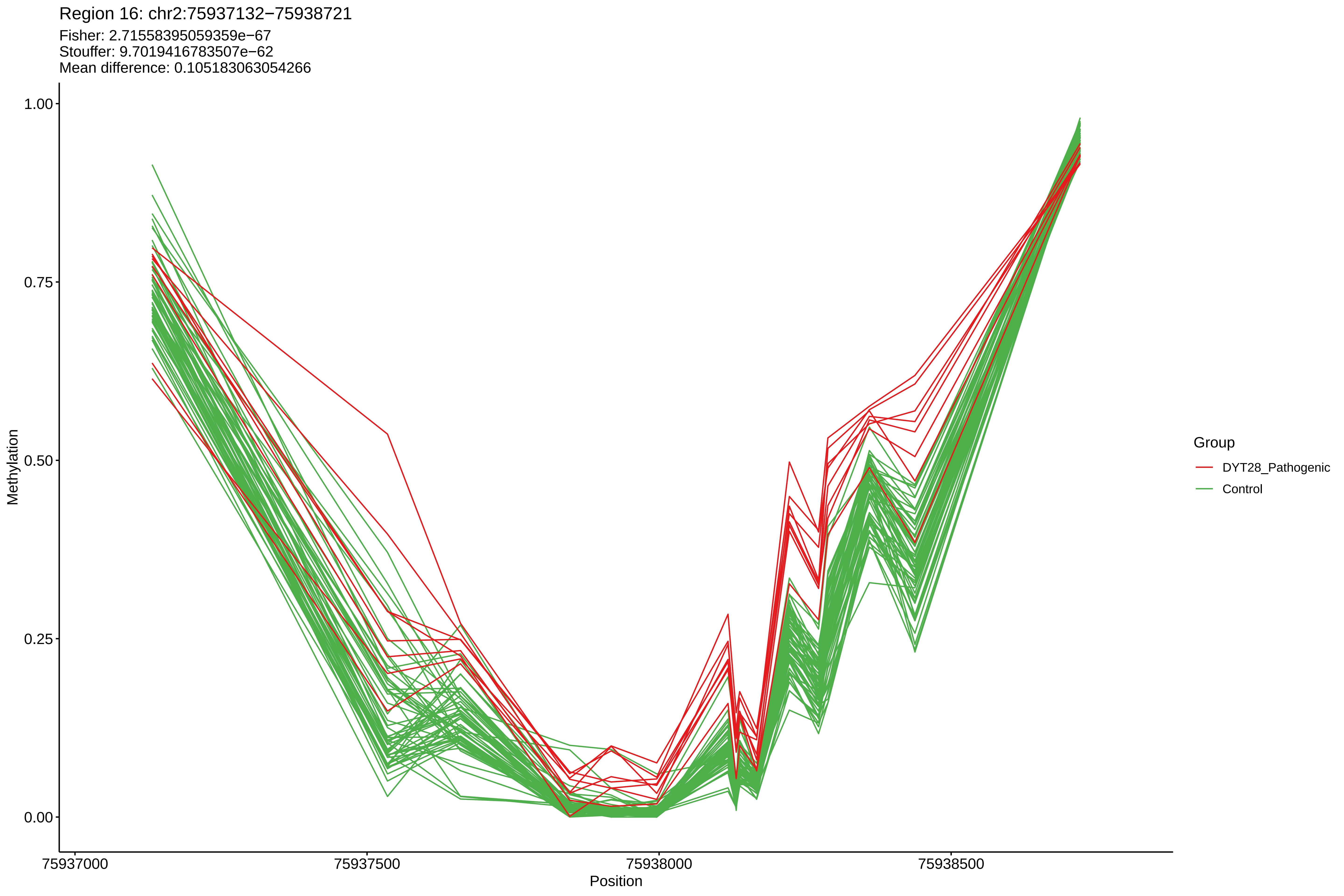
Region 16: chr2:75937132−75938721
Fisher: 2.71558395059359e−67
Stouffer: 9.7019416783507e−62
Mean difference: 0.105183063054266
Methylation
1.00
0.75
0.50
0.25
0.00
75937000
75937500
75938000
75938500
Position
Group
DYT28_Pathogenic
Control

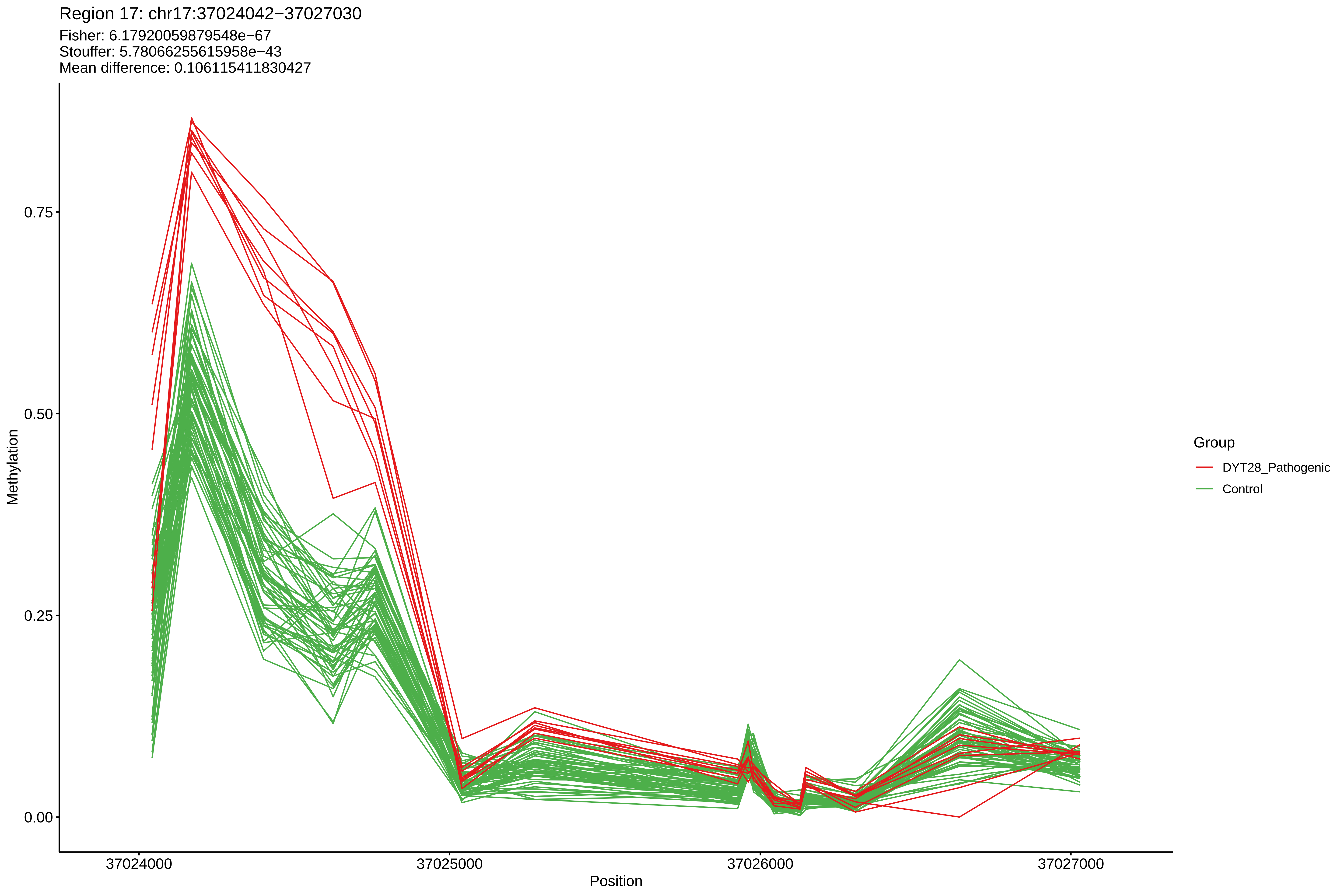
Region 17: chr17:37024042−37027030
Fisher: 6.17920059879548e−67
Stouffer: 5.78066255615958e−43
Mean difference: 0.106115411830427
Methylation
0.75
0.50
0.25
0.00
37024000
37025000
37026000
37027000
Position
Group
DYT28_Pathogenic
Control

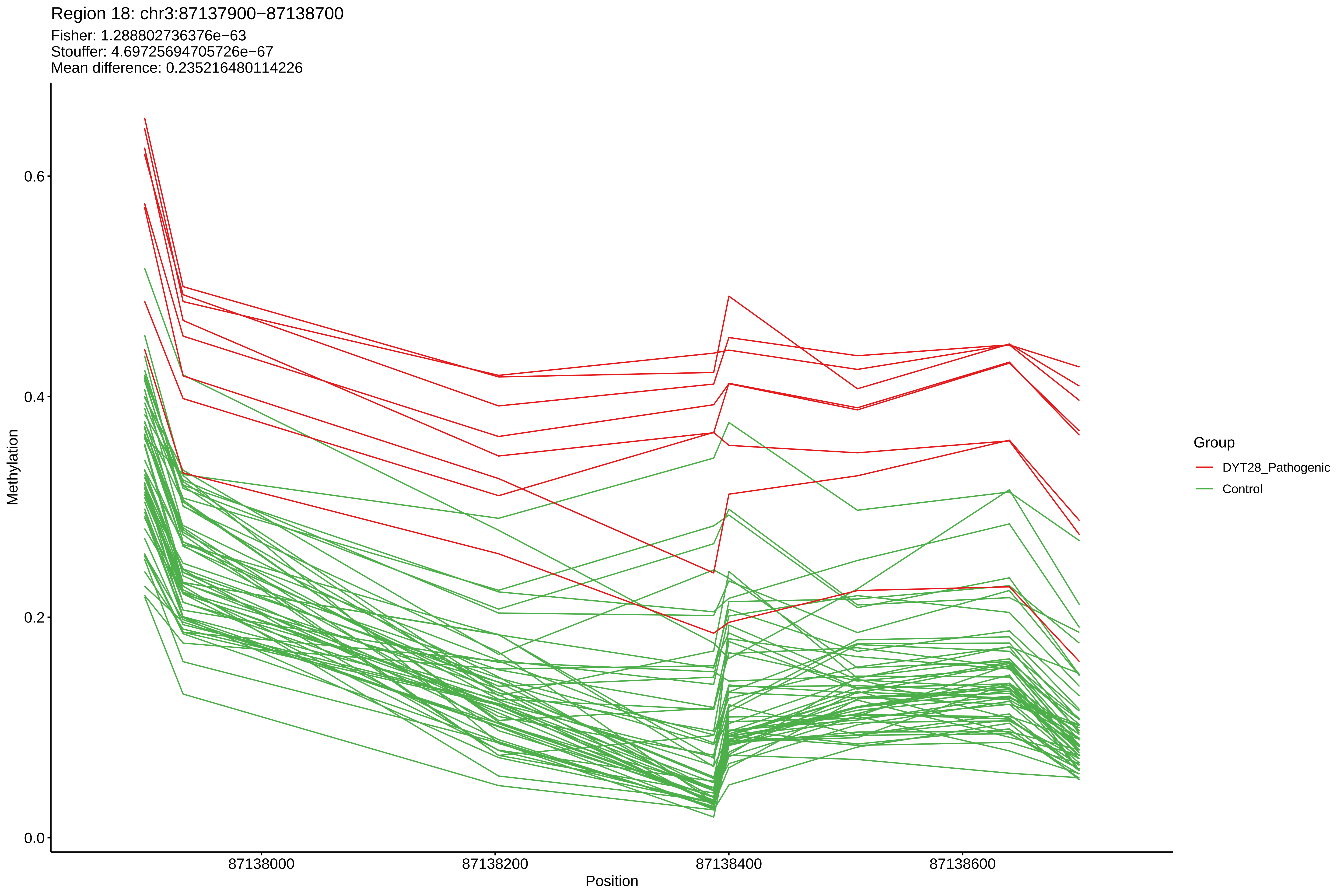
Region 18: chr3:87137900−87138700
Fisher: 1.288802736376e−63
Stouffer: 4.69725694705726e−67
Mean difference: 0.235216480114226
Methylation
0.0
0.2
0.4
0.6
Position
87138000
87138200
87138400
87138600
Group
DYT28_Pathogenic
Control

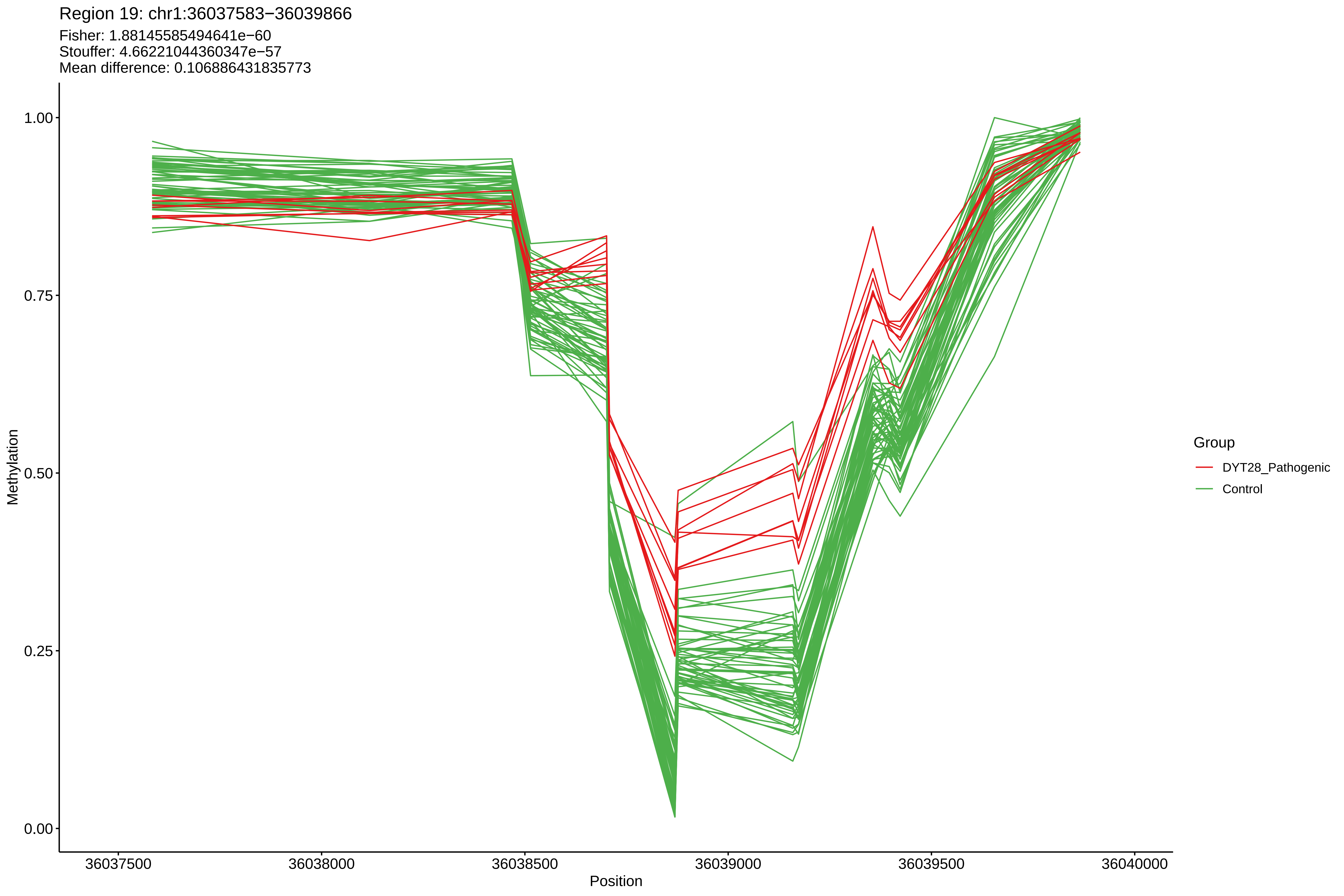

Region 19: chr1:36037583−36039866
Fisher: 1.88145585494641e−60
Stouffer: 4.66221044360347e−57
Mean difference: 0.106886431835773
1.00
0.75
0.50
0.25
0.00
Methylation
36037500
36038000
36038500
36039000
36039500
36040000
Position
Group
DYT28_Pathogenic
Control

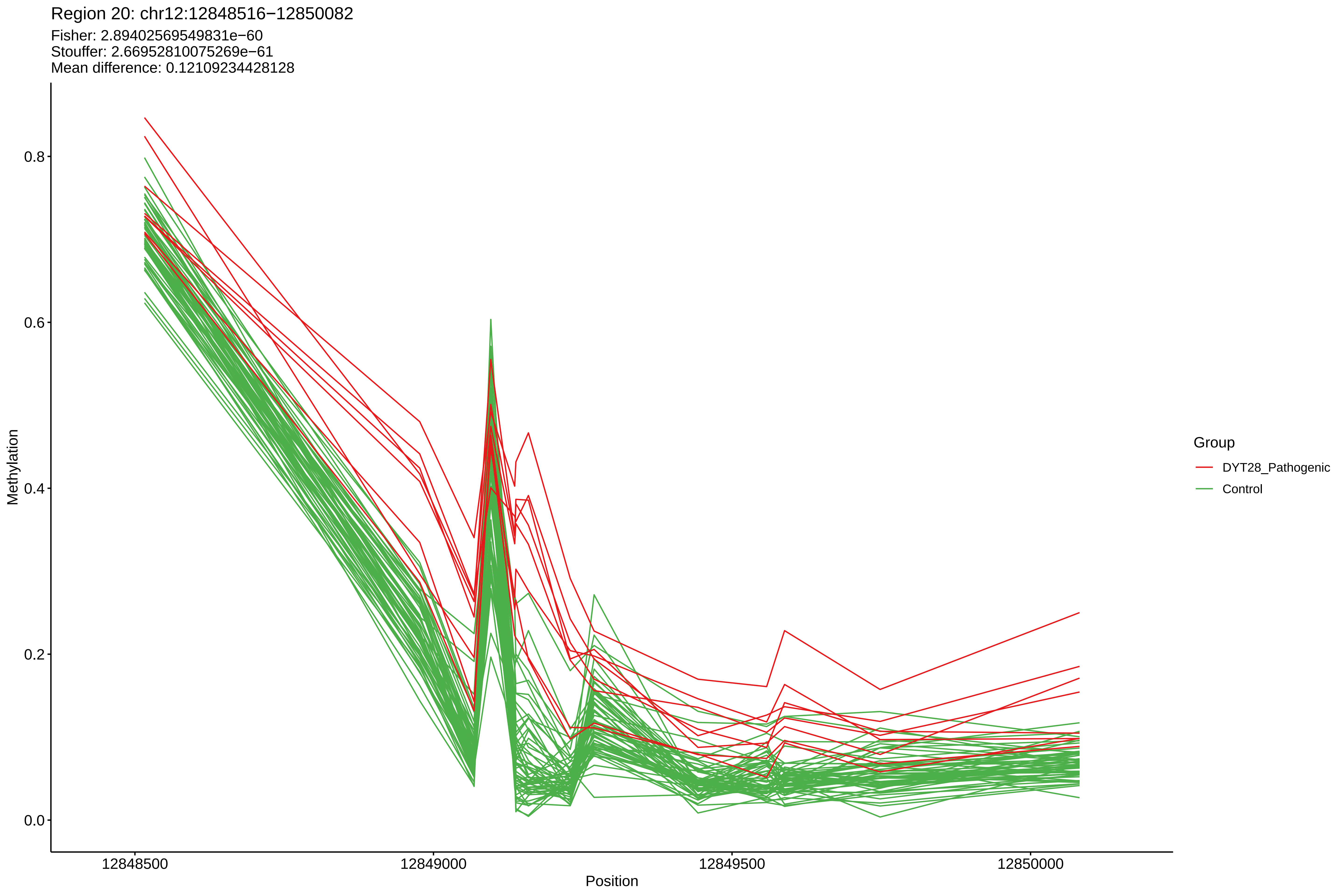
Region 20: chr12:12848516−12850082
Fisher: 2.89402569549831e−60
Stouffer: 2.66952810075269e−61
Mean difference: 0.12109234428128
Methylation
0.0
0.2
0.4
0.6
0.8
Position
12848500
12849000
12849500
12850000
Group
DYT28_Pathogenic
Control

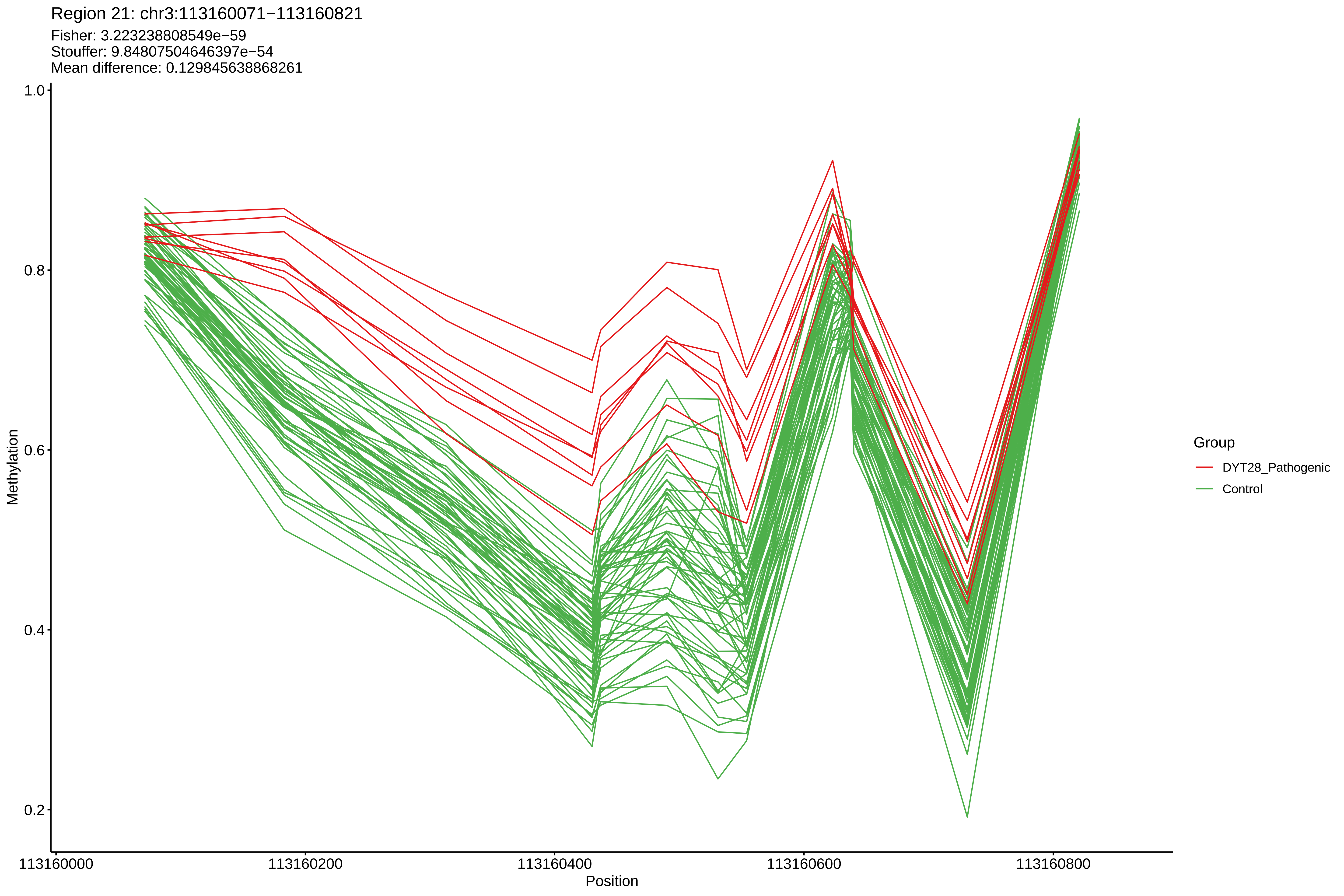
Region 21: chr3:113160071−113160821
Fisher: 3.223238808549e−59
Stouffer: 9.84807504646397e−54
Mean difference: 0.129845638868261
1.0
0.8
0.6
0.4
0.2
Methylation
113160000
113160200
113160400
113160600
113160800
Position
Group
DYT28_Pathogenic
Control

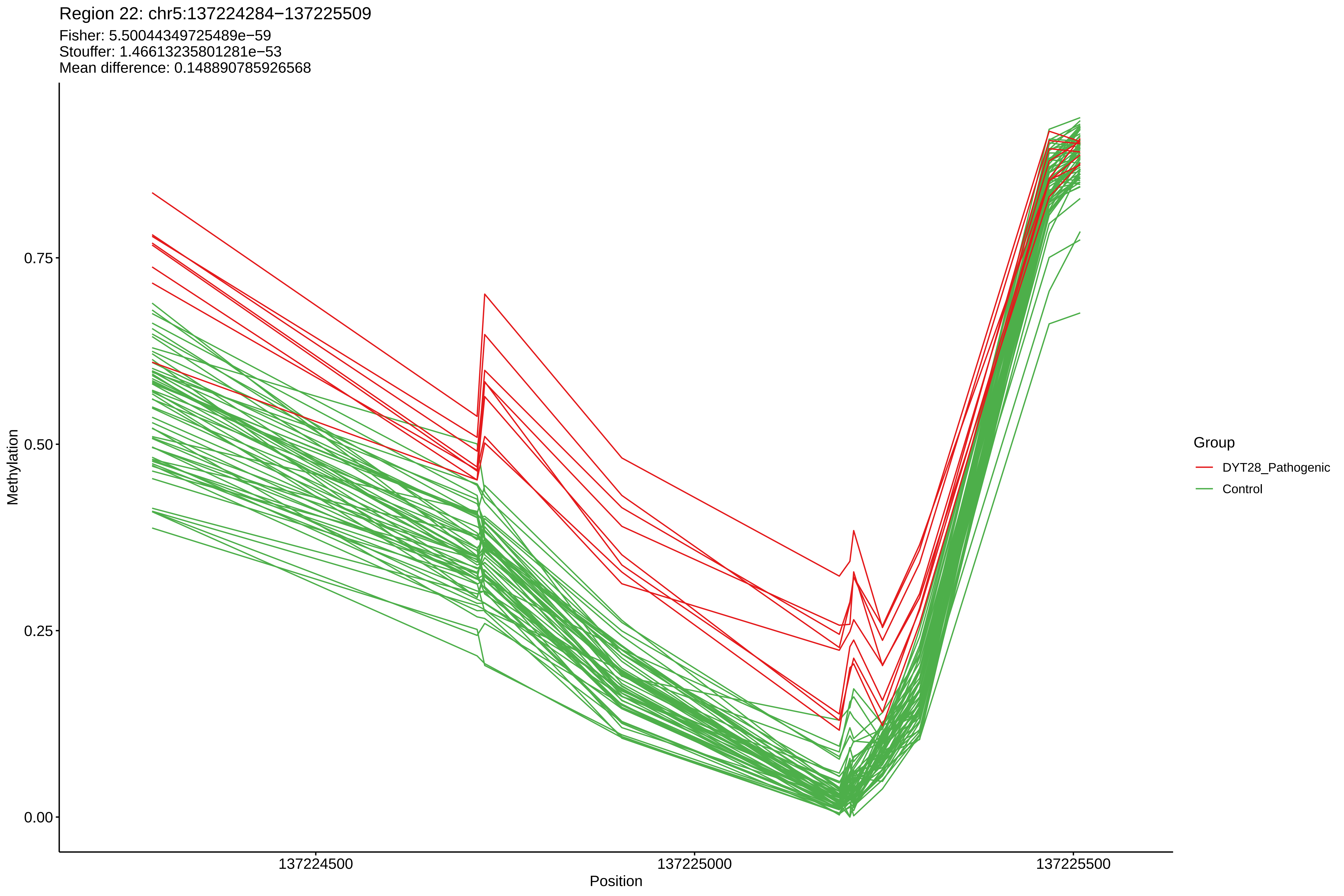
Region 22: chr5:137224284−137225509
Fisher: 5.50044349725489e−59
Stouffer: 1.46613235801281e−53
Mean difference: 0.148890785926568
Methylation
0.75
0.50
0.25
0.00
137224500
137225000
137225500
Position
Group
DYT28_Pathogenic
Control

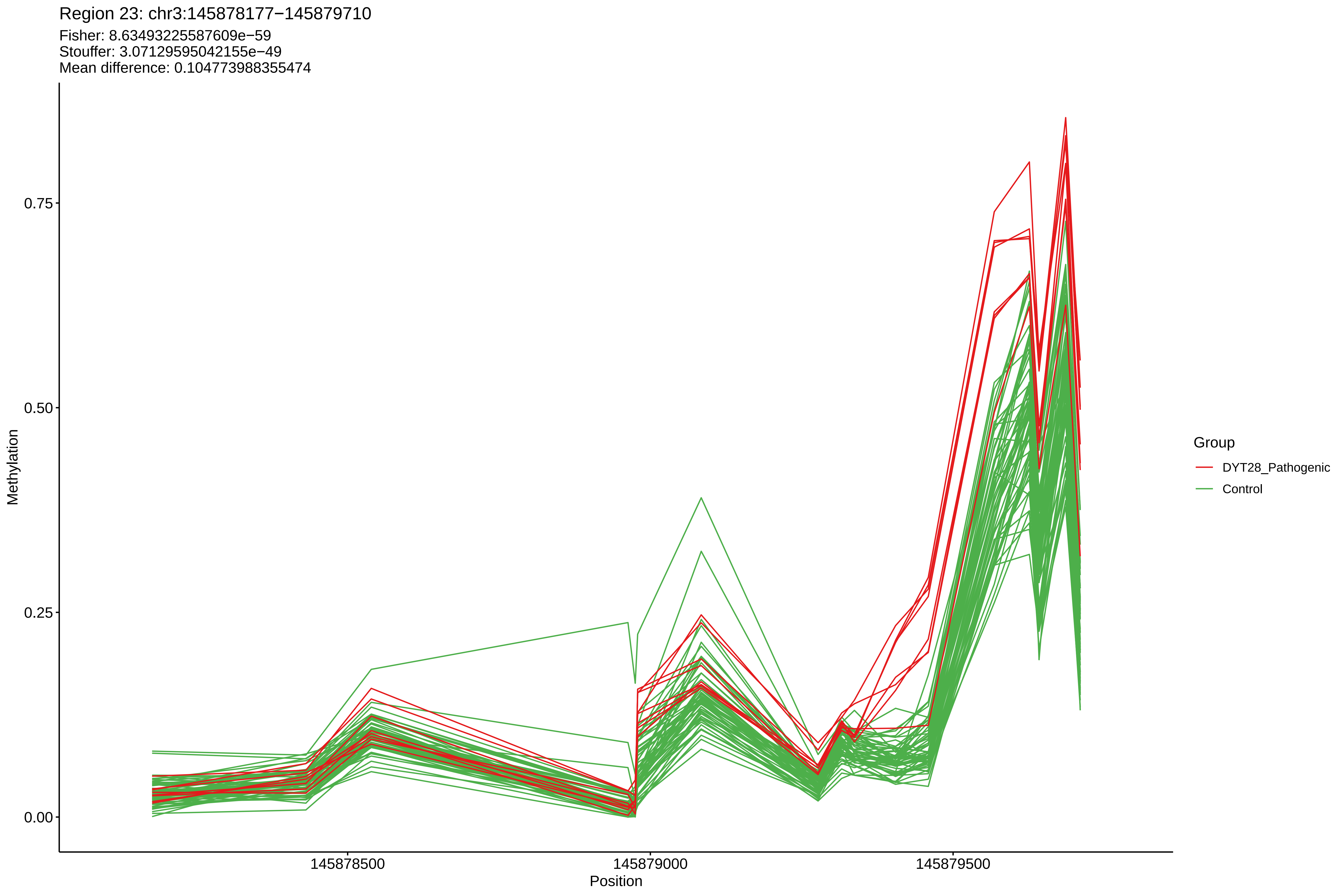
Region 23: chr3:145878177−145879710
Fisher: 8.63493225587609e−59
Stouffer: 3.07129595042155e−49
Mean difference: 0.104773988355474
0.75
0.50
0.25
0.00
Methylation
145878500
145879000
145879500
Position
Group
DYT28_Pathogenic
Control

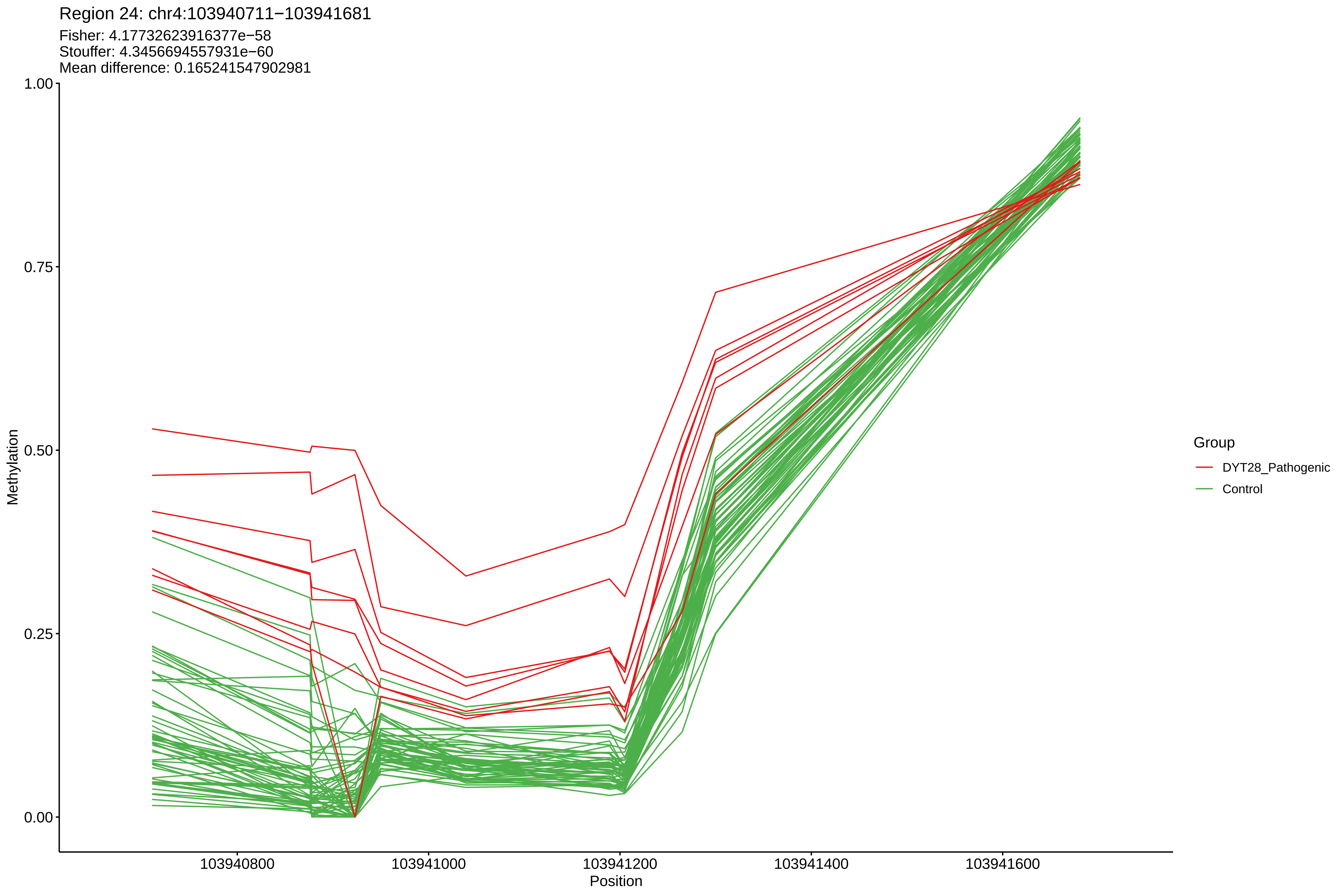

Region 24: chr4:103940711−103941681
Fisher: 4.17732623916377e−58
Stouffer: 4.3456694557931e−60
Mean difference: 0.165241547902981
1.00
0.75
0.50
0.25
0.00
Methylation
103940800
103941000
103941200
103941400
103941600
Position
Group
DYT28_Pathogenic
Control

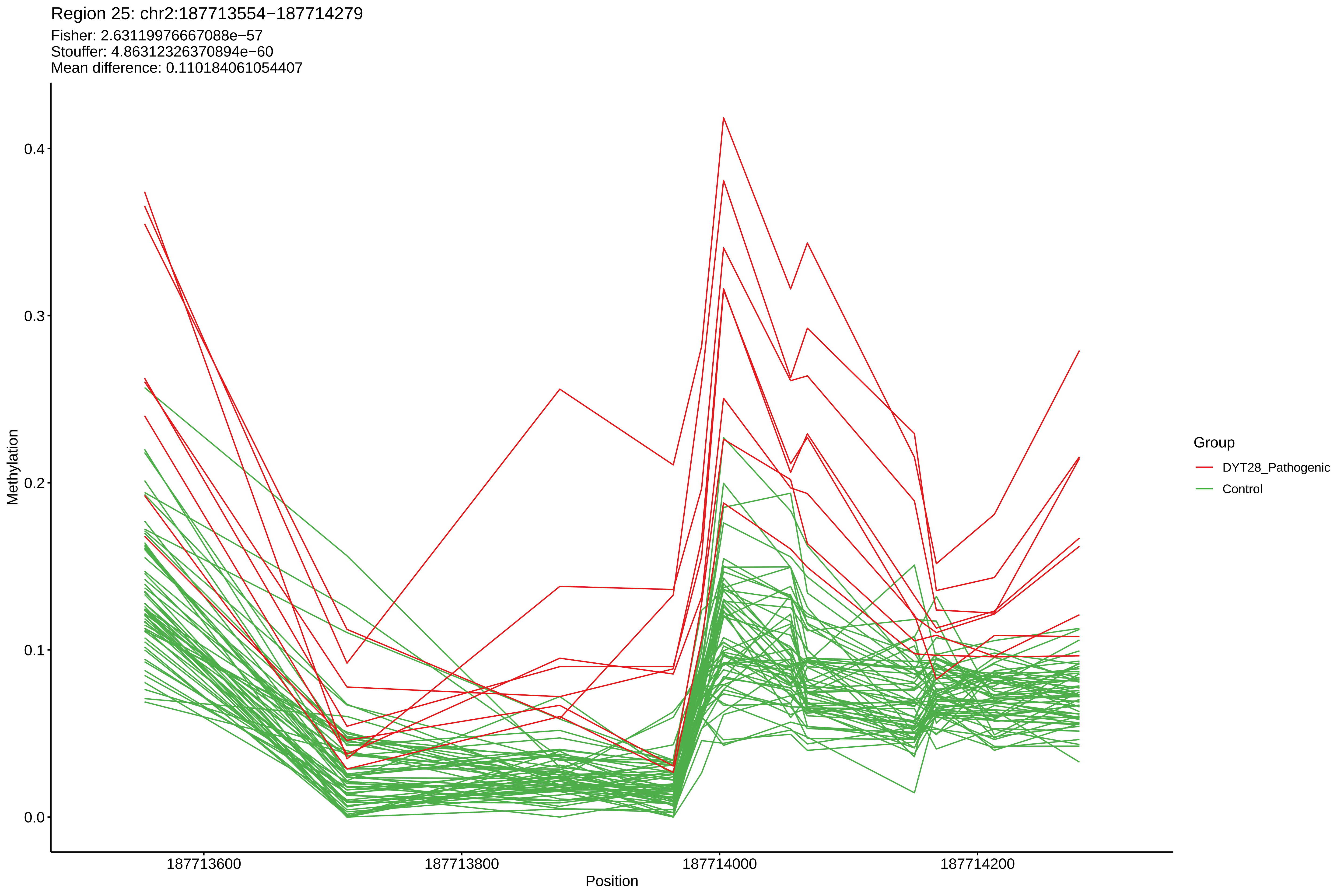

Region 25: chr2:187713554−187714279
Fisher: 2.63119976667088e−57
Stouffer: 4.86312326370894e−60
Mean difference: 0.110184061054407
Methylation
0.0
0.1
0.2
0.3
0.4
187713600
187713800
187714000
187714200
Position
Group
DYT28_Pathogenic
Control

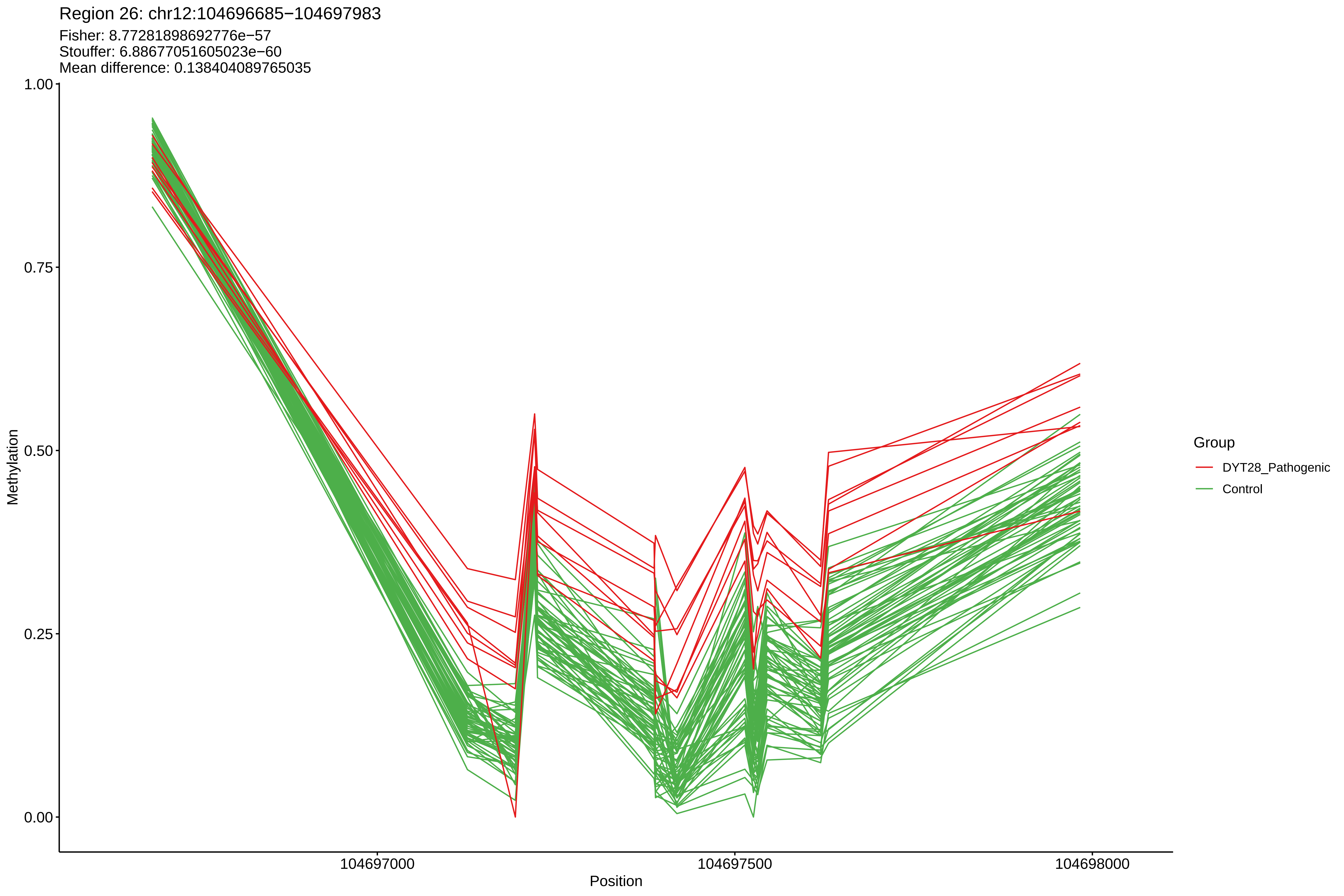
Region 26: chr12:104696685−104697983
Fisher: 8.77281898692776e−57
Stouffer: 6.88677051605023e−60
Mean difference: 0.138404089765035
1.00
0.75
0.50
0.25
0.00
Methylation
104697000
104697500
104698000
Position
Group
DYT28_Pathogenic
Control

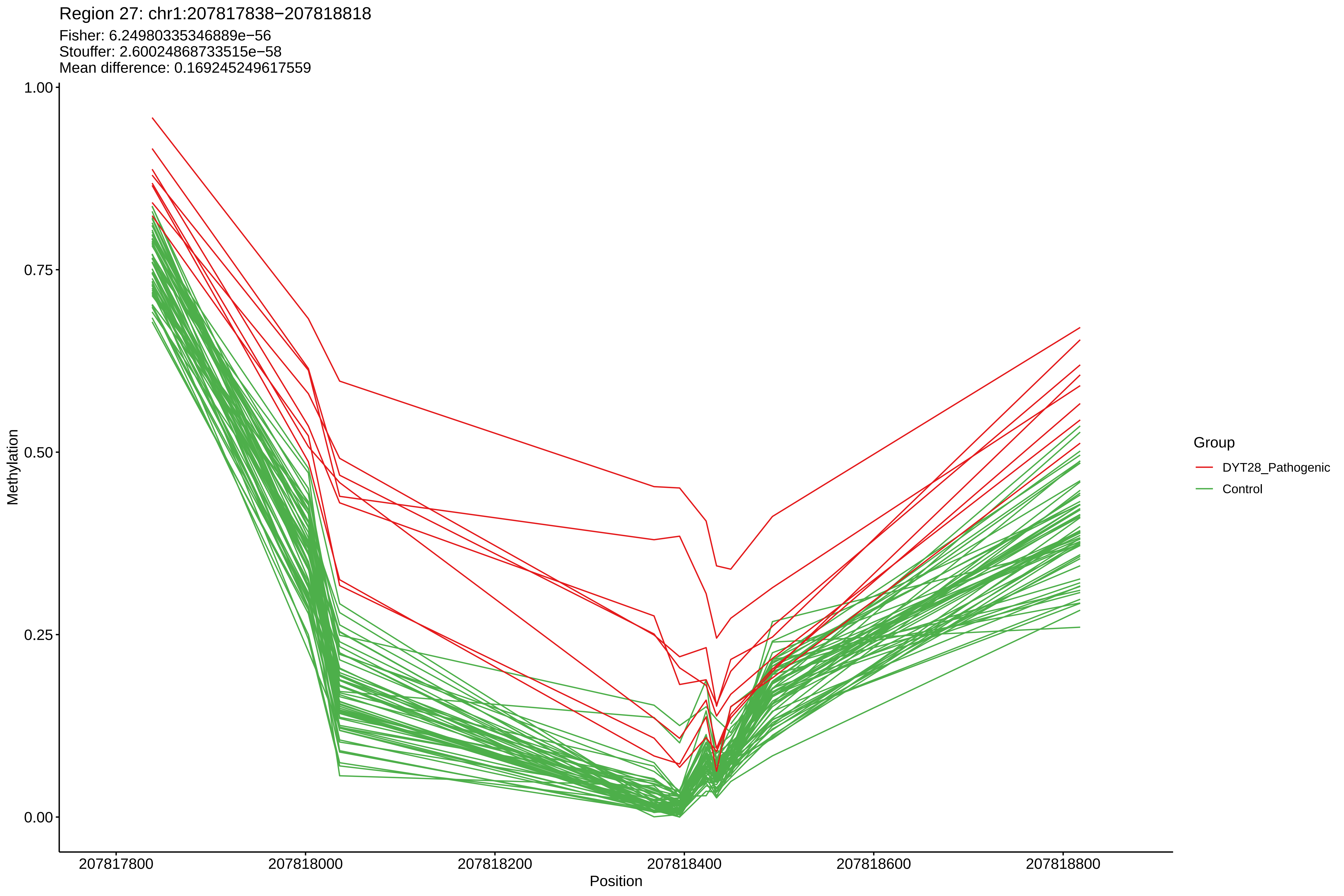
Region 27: chr1:207817838−207818818
Fisher: 6.24980335346889e−56
Stouffer: 2.60024868733515e−58
Mean difference: 0.169245249617559
Methylation
1.00
0.75
0.50
0.25
0.00
207817800
207818000
207818200
207818400
207818600
207818800
Position
Group
DYT28_Pathogenic
Control

Region 28: chr14:96890120−96891077

Fisher: 3.70859934958739e−55
Stouffer: 3.4035349272261e−44
Mean difference: 0.188359990268931

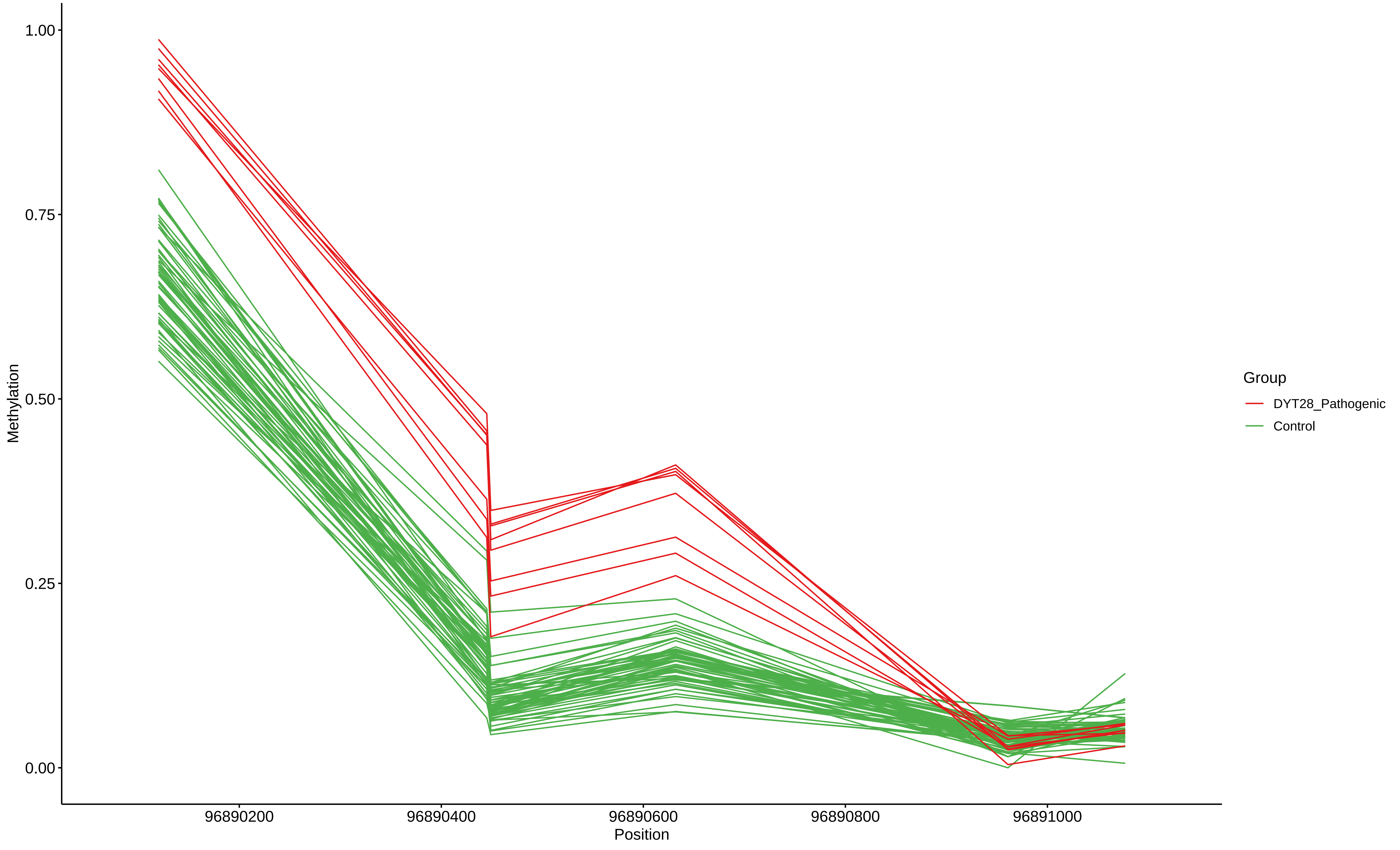

Region 29: chr2:165811765−165812478

Fisher: 1.39117011915112e−54

Stouffer: 3.10303253993836e−51

Mean difference: 0.101044444159004

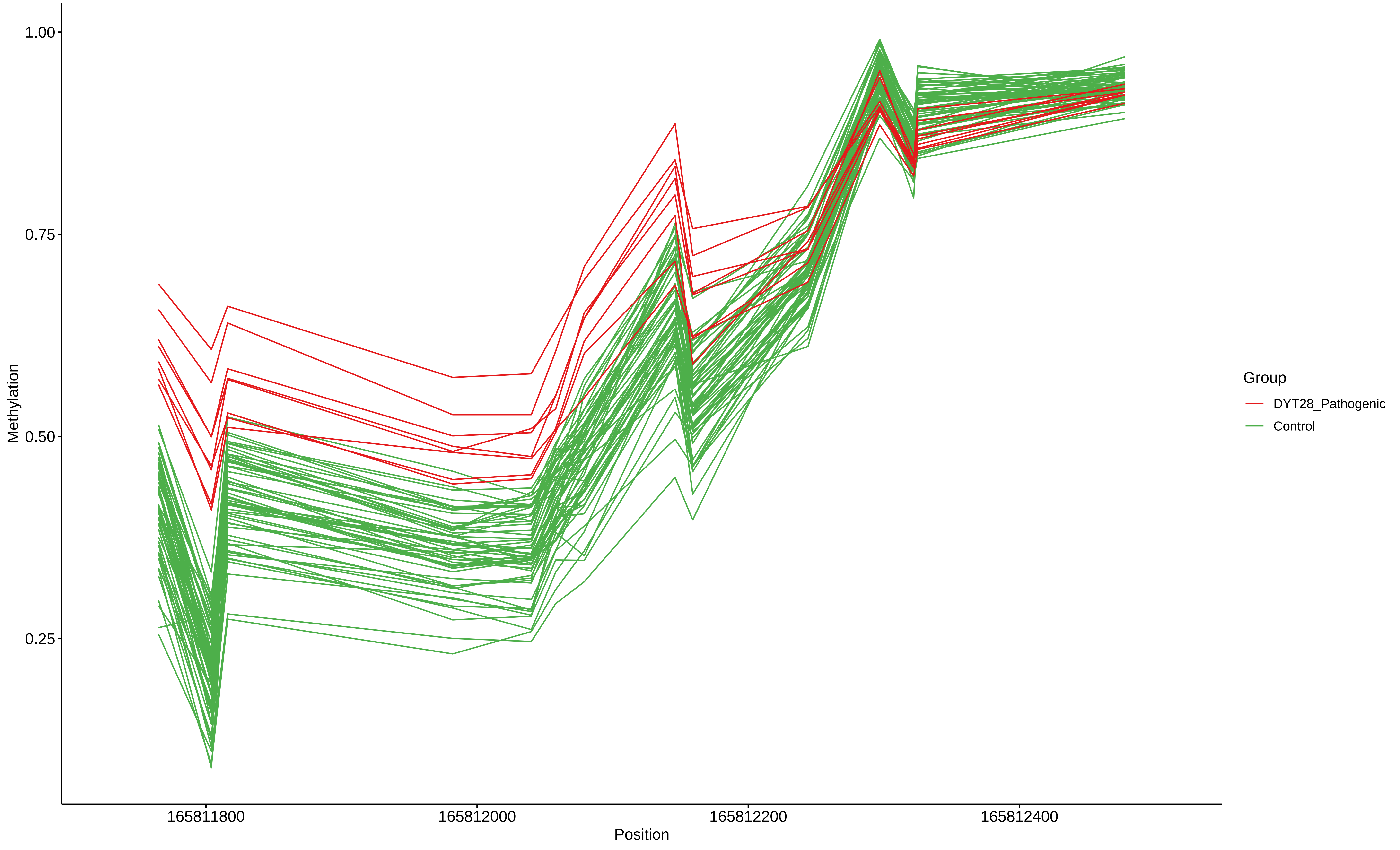

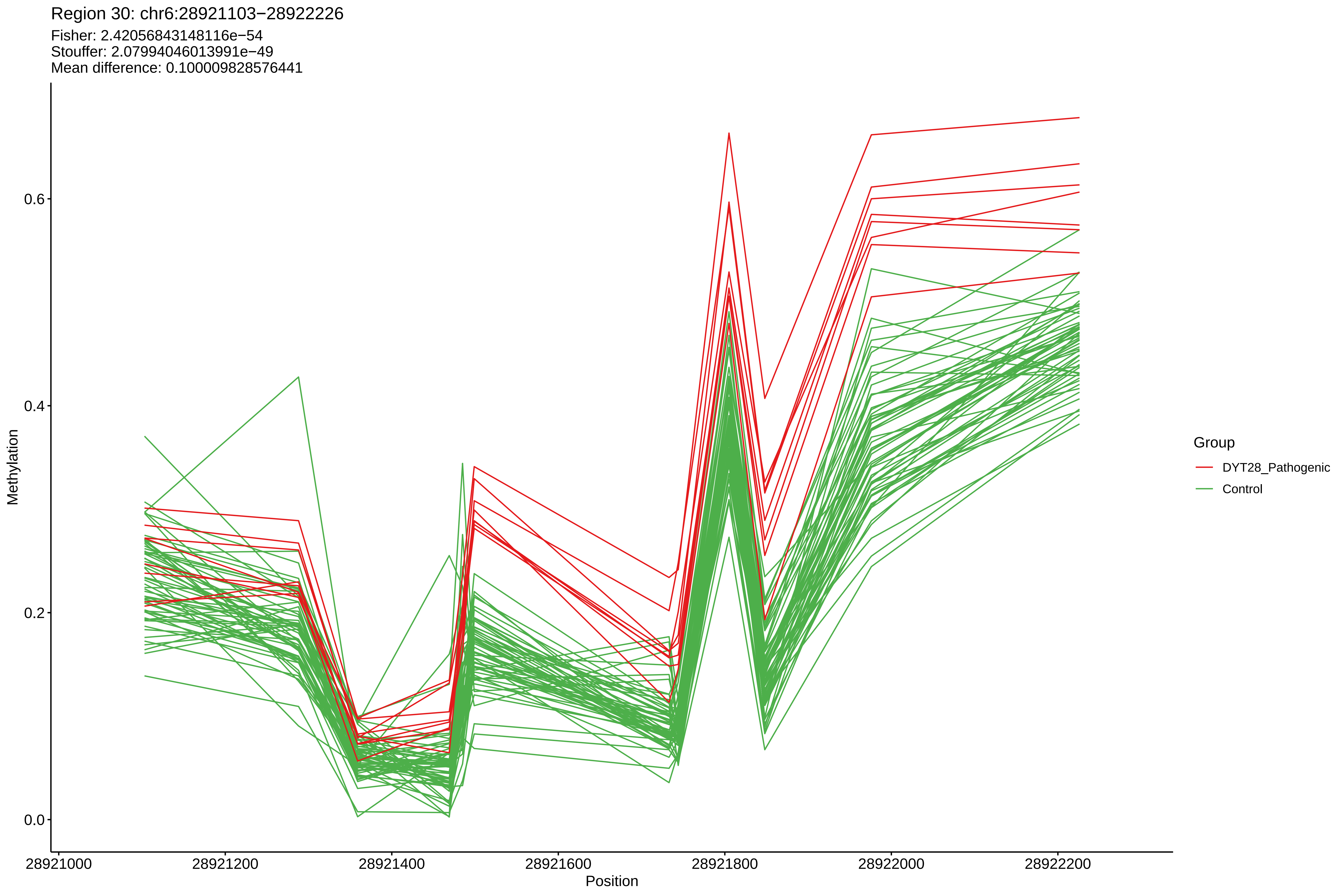

Region 30: chr6:28921103−28922226
Fisher: 2.42056843148116e−54
Stouffer: 2.07994046013991e−49
Mean difference: 0.100009828576441
Methylation
0.0
0.2
0.4
0.6
28921000
28921200
28921400
28921600
28921800
28922000
28922200
Position
Group
DYT28_Pathogenic
Control

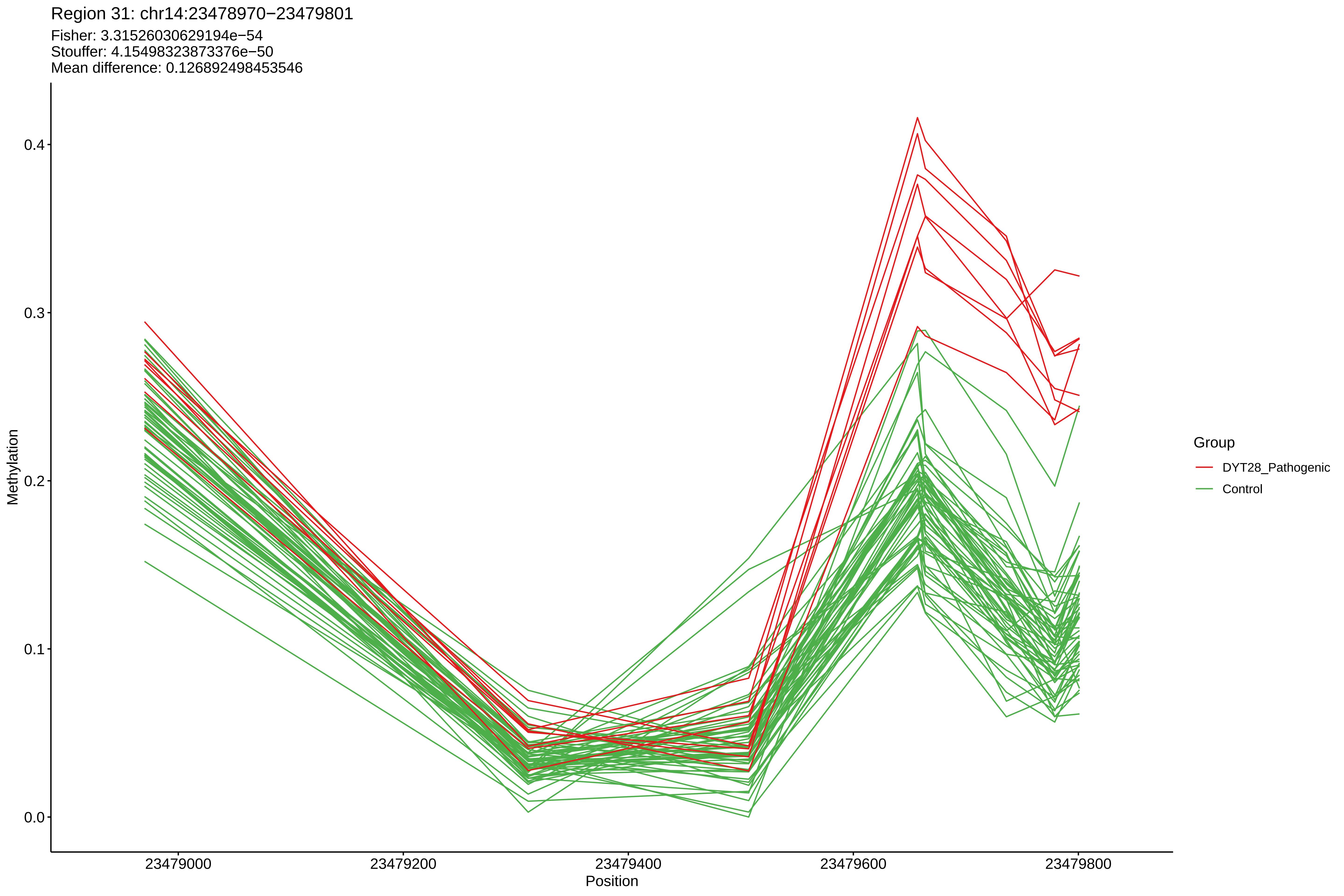
Region 31: chr14:23478970−23479801
Fisher: 3.31526030629194e−54
Stouffer: 4.15498323873376e−50
Mean difference: 0.126892498453546
Methylation
0.0
0.1
0.2
0.3
0.4
23479000
23479200
23479400
23479600
23479800
Position
Group
DYT28_Pathogenic
Control

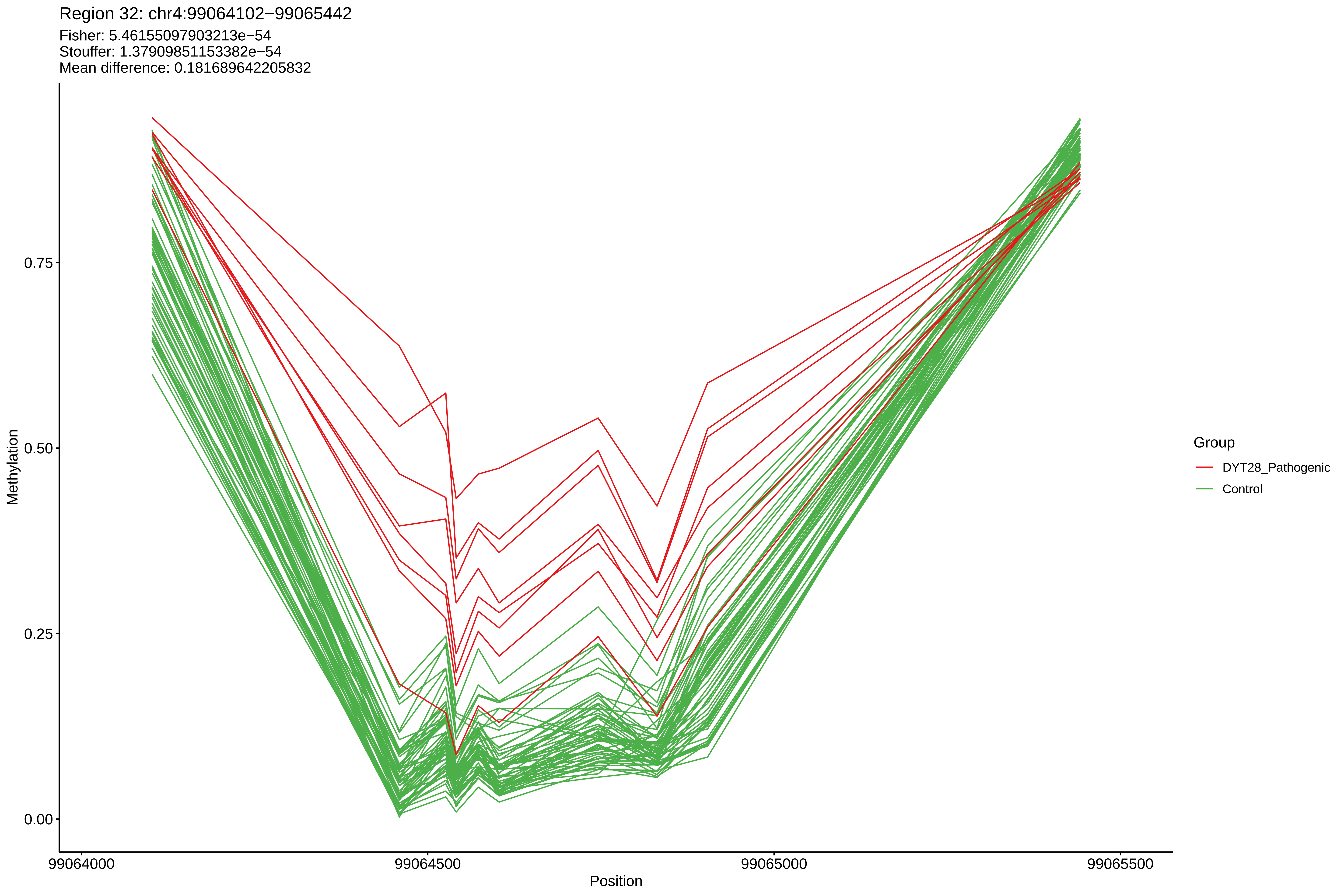

Region 32: chr4:99064102−99065442
Fisher: 5.46155097903213e−54
Stouffer: 1.37909851153382e−54
Mean difference: 0.181689642205832
Methylation
0.00
0.25
0.50
0.75
99064000
99064500
99065000
99065500
Position
Group
DYT28_Pathogenic
Control

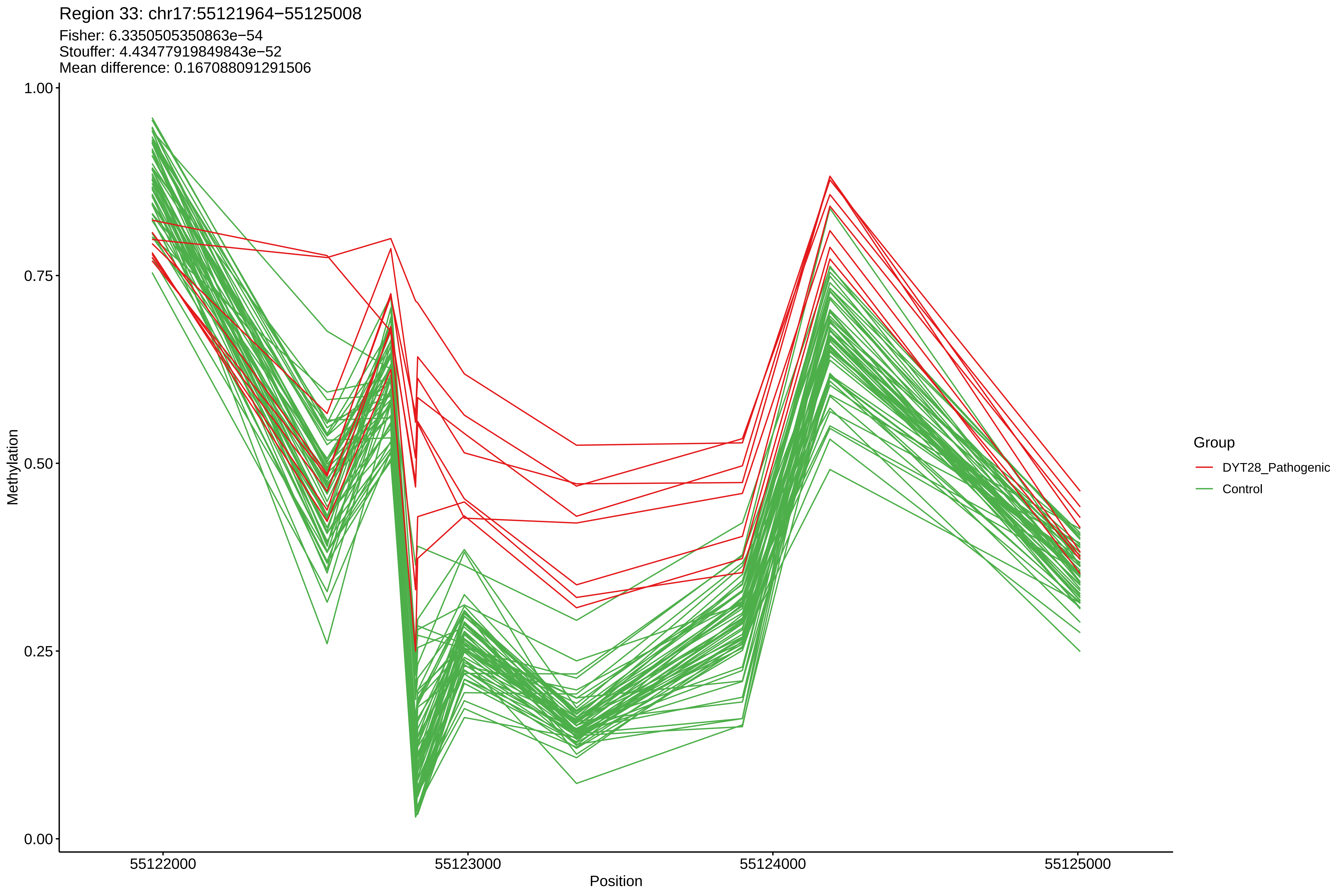
Region 33: chr17:55121964−55125008
Fisher: 6.3350505350863e−54
Stouffer: 4.43477919849843e−52
Mean difference: 0.167088091291506
1.00
0.75
0.50
0.25
0.00
Methylation
55122000
55123000
55124000
55125000
Position
Group
DYT28_Pathogenic
Control

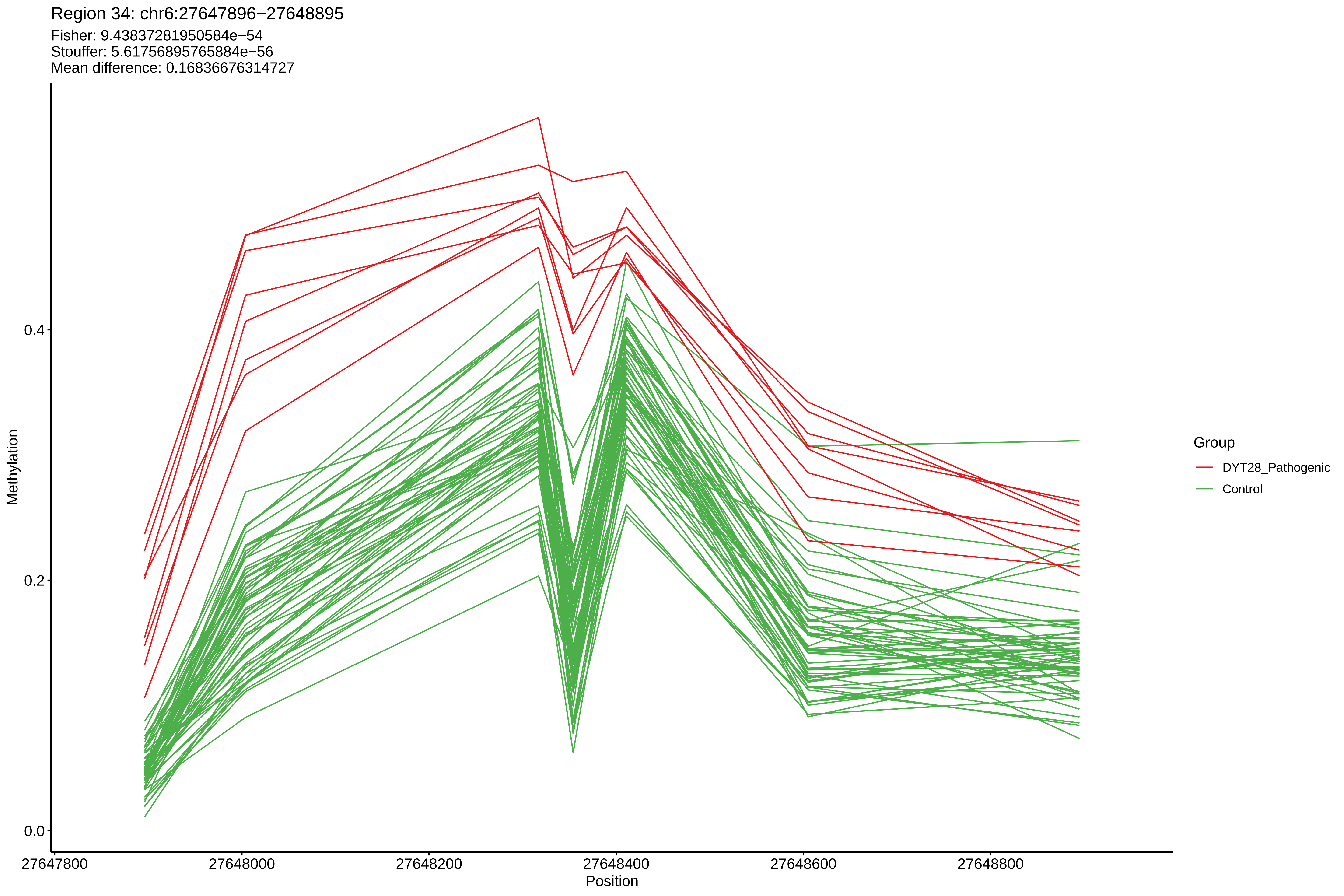
Region 34: chr6:27647896−27648895
Fisher: 9.43837281950584e−54
Stouffer: 5.61756895765884e−56
Mean difference: 0.16836676314727
Methylation
0.0
0.2
0.4
Position
27647800
27648000
27648200
27648400
27648600
27648800
Group
DYT28_Pathogenic
Control

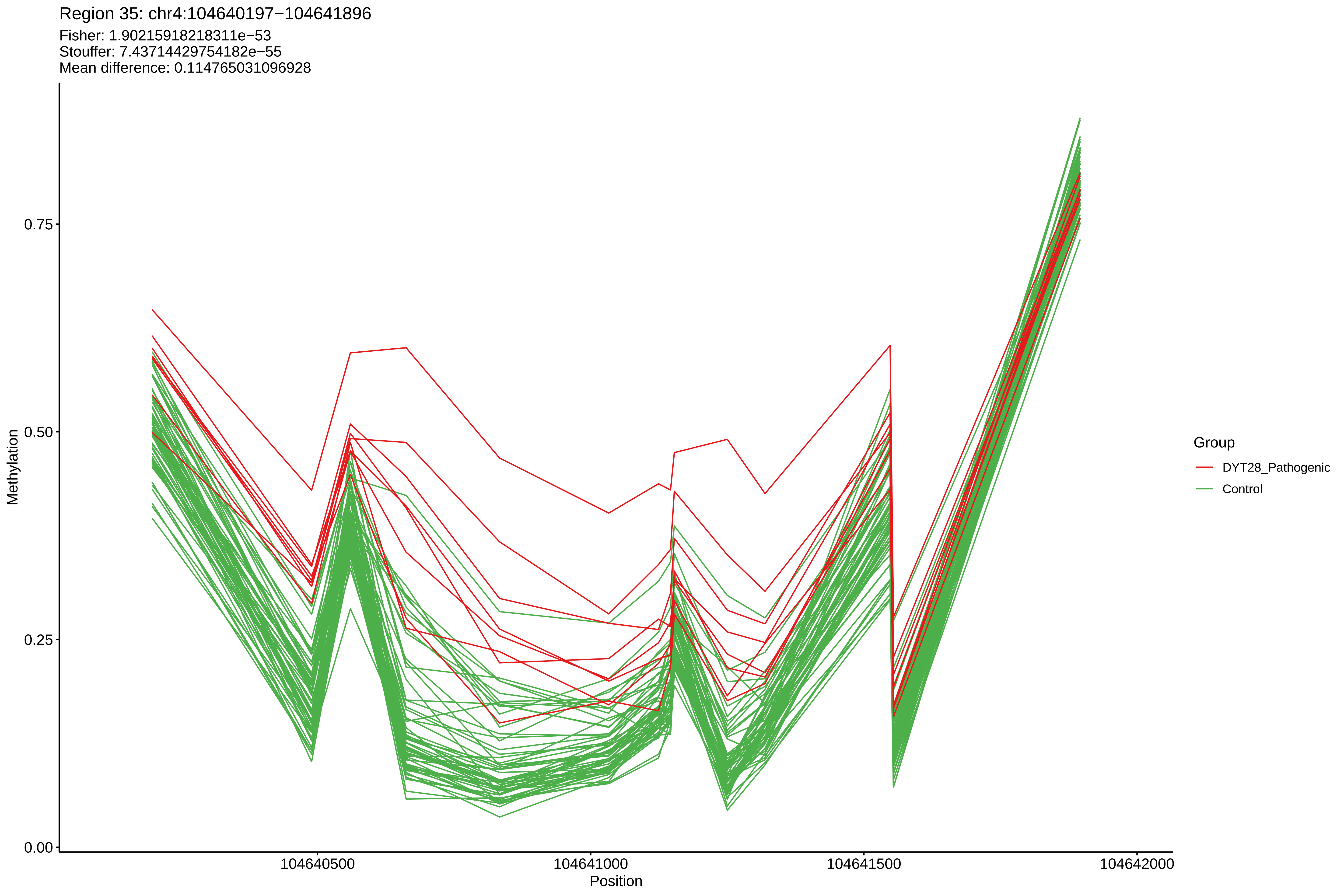
Region 35: chr4:104640197−104641896
Fisher: 1.90215918218311e−53
Stouffer: 7.43714429754182e−55
Mean difference: 0.114765031096928
Methylation
0.00
0.25
0.50
0.75
104640500
104641000
104641500
104642000
Position
Group
DYT28_Pathogenic
Control

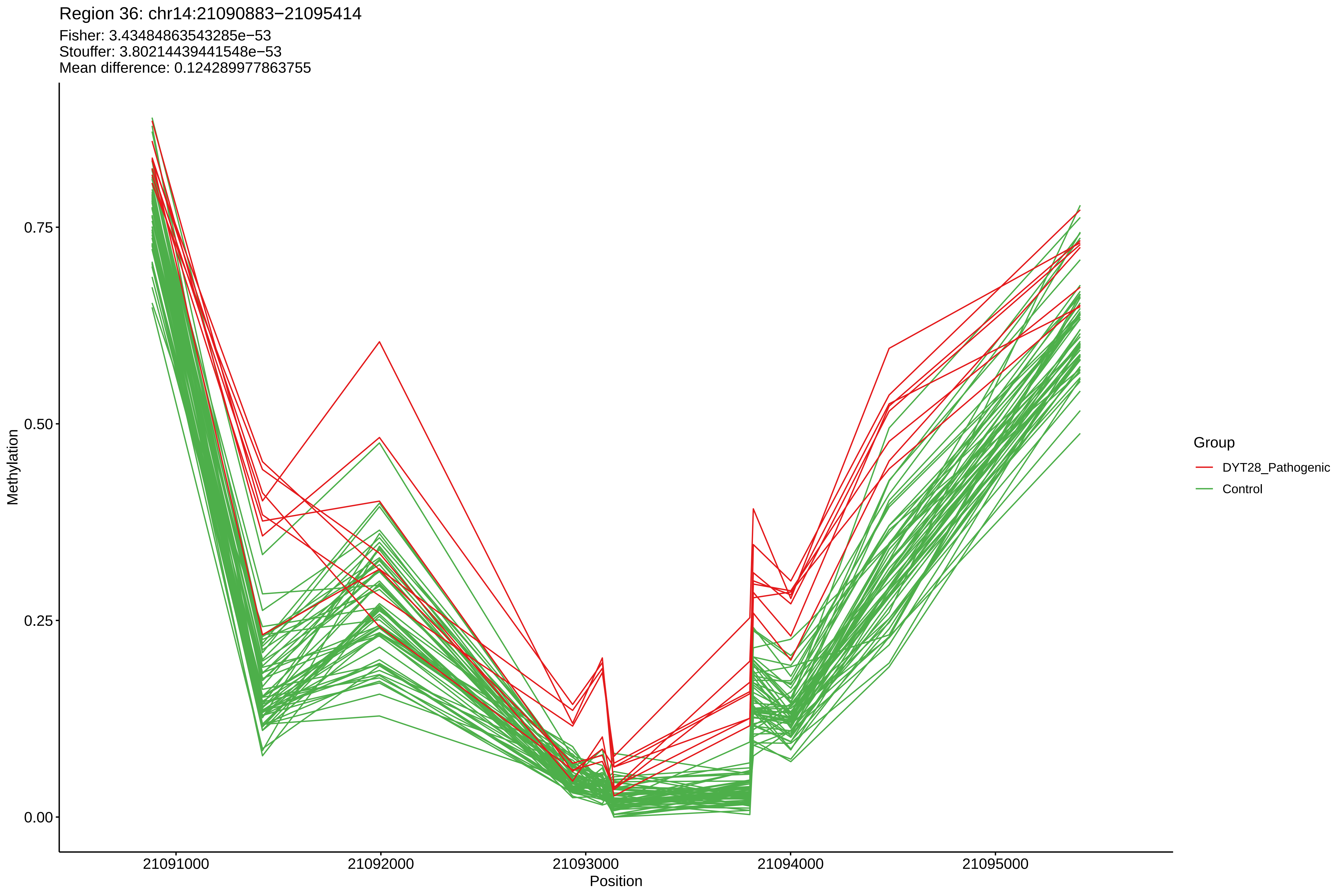
Region 36: chr14:21090883−21095414
Fisher: 3.43484863543285e−53
Stouffer: 3.80214439441548e−53
Mean difference: 0.124289977863755
Methylation
0.75
0.50
0.25
0.00
21091000
21092000
21093000
21094000
21095000
Position
Group
DYT28_Pathogenic
Control

Region 37: chr2:71205052−71206528

Fisher: 2.58646502578219e−52

Stouffer: 4.45648251595386e−52

Mean difference: 0.109374718497371

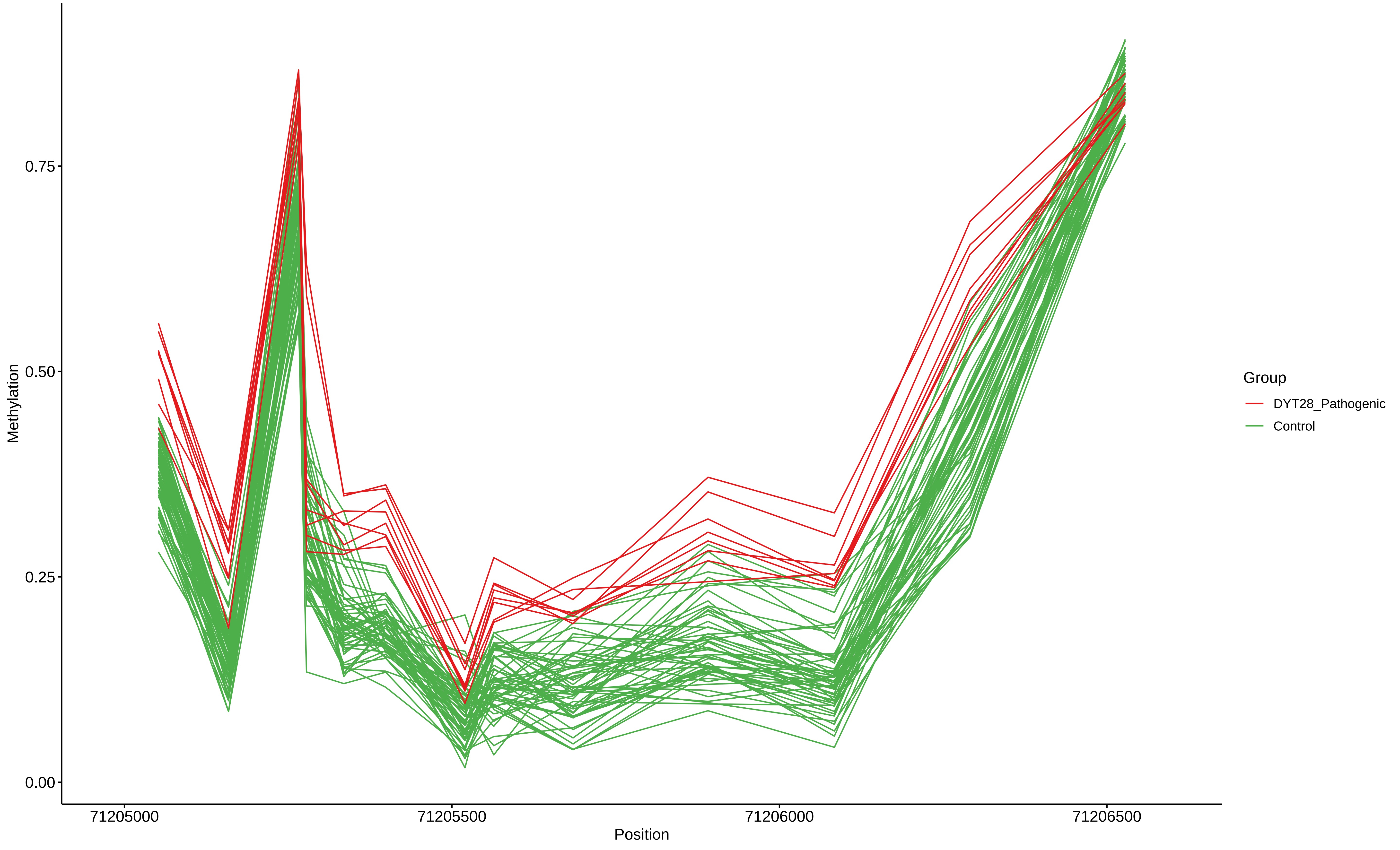

Region 38: chr3:52351355−52351963

Fisher: 7.31916098973452e−52
Stouffer: 6.63955108000881e−54
Mean difference: 0.205720262178768

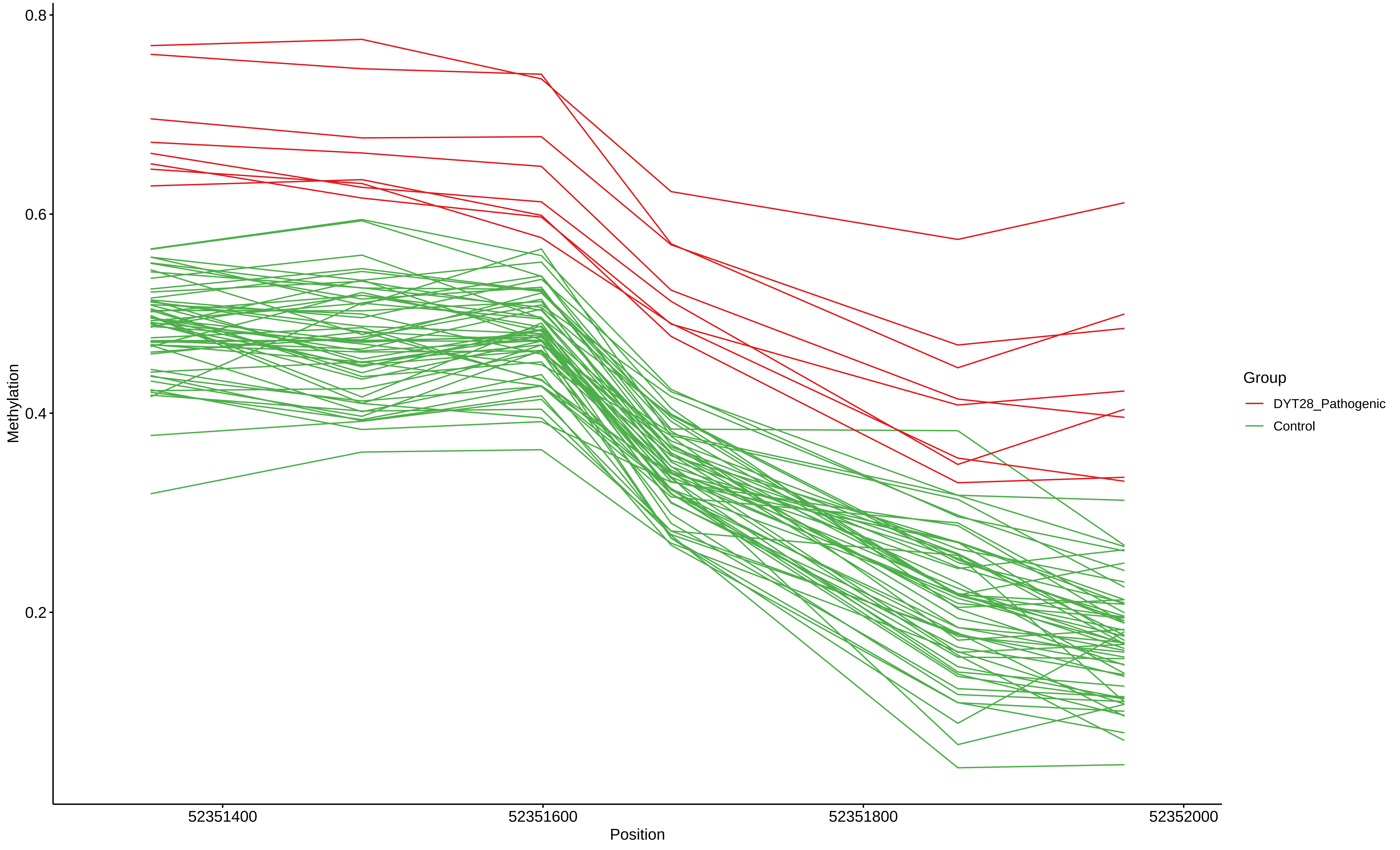

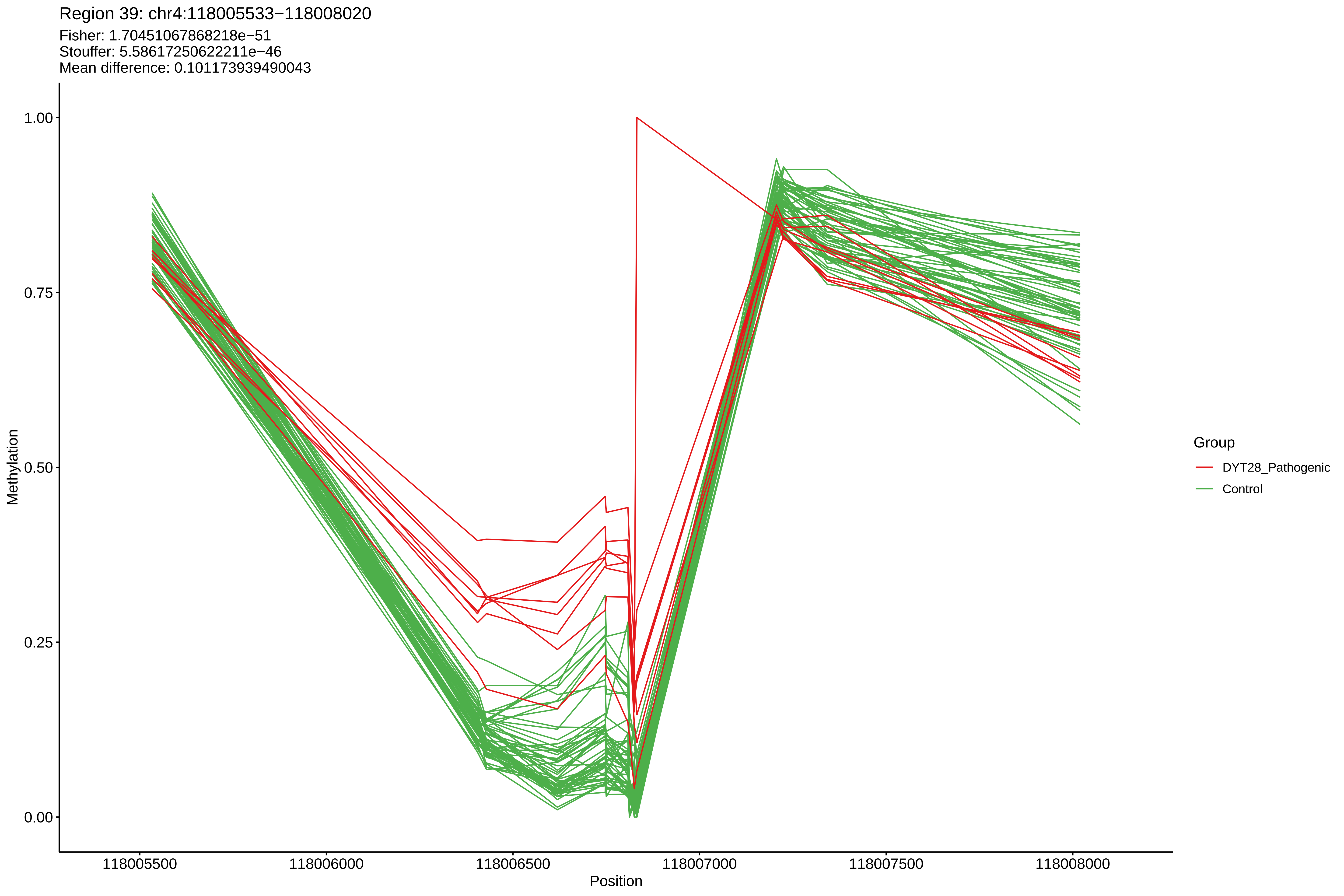

Region 39: chr4:118005533−118008020
Fisher: 1.70451067868218e−51
Stouffer: 5.58617250622211e−46
Mean difference: 0.101173939490043
Methylation
1.00
0.75
0.50
0.25
0.00
118005500
118006000
118006500
118007000
118007500
118008000
Position
Group
DYT28_Pathogenic
Control

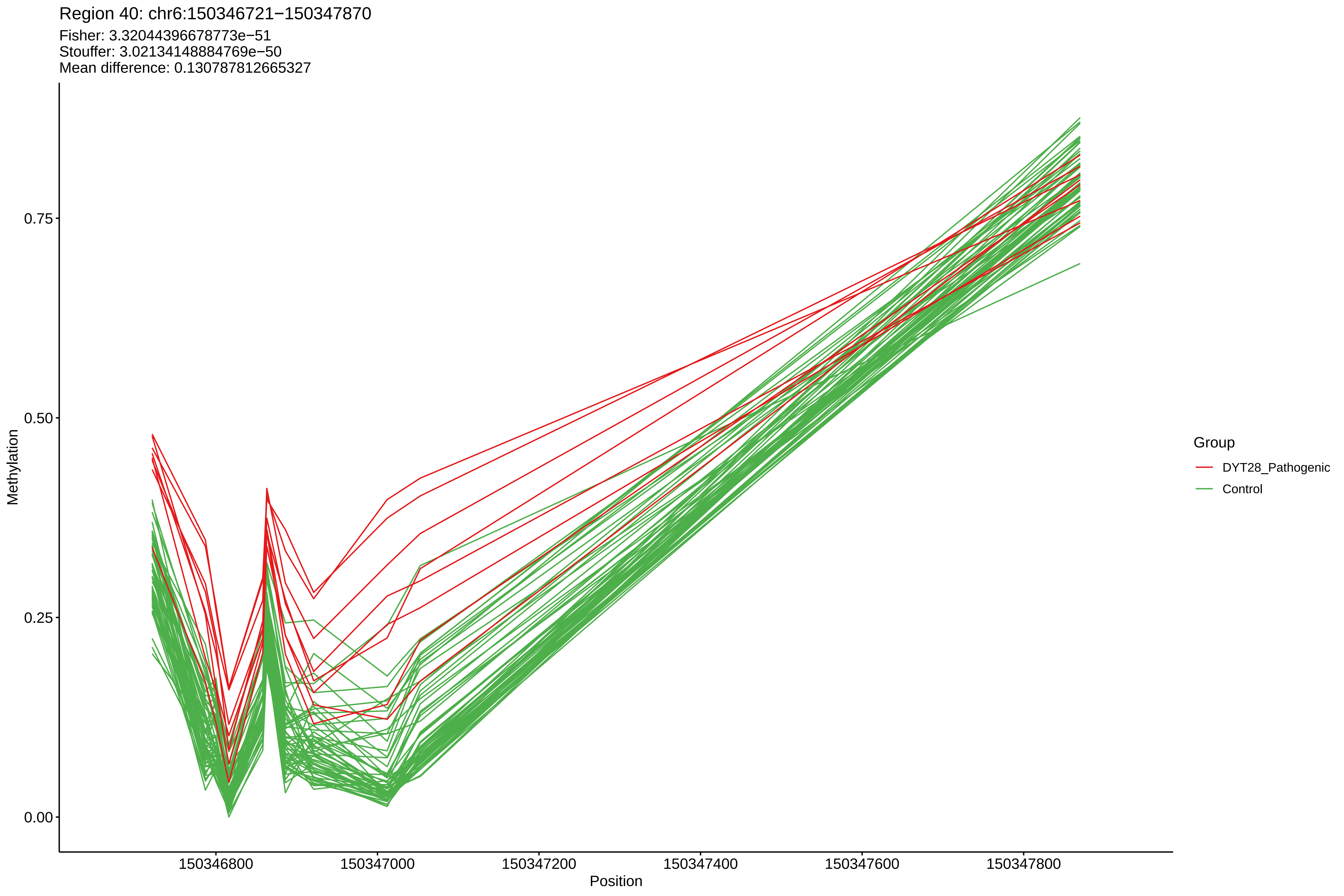

Region 40: chr6:150346721−150347870
Fisher: 3.32044396678773e−51
Stouffer: 3.02134148884769e−50
Mean difference: 0.130787812665327
Methylation
0.75
0.50
0.25
0.00
150346800
150347000
150347200
150347400
150347600
150347800
Position
Group
DYT28_Pathogenic
Control

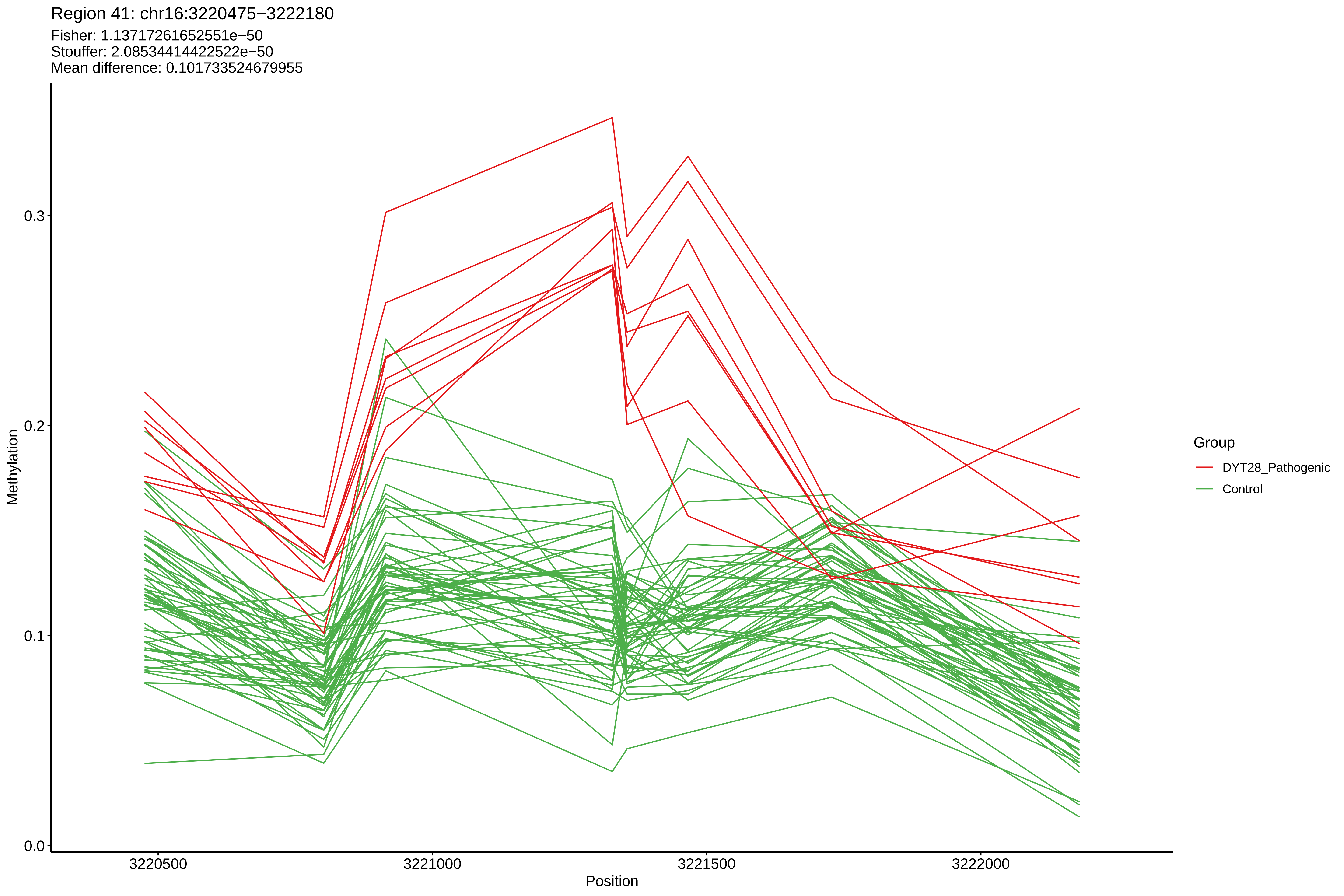

Region 41: chr16:3220475−3222180
Fisher: 1.13717261652551e−50
Stouffer: 2.08534414422522e−50
Mean difference: 0.101733524679955
Methylation
0.0
0.1
0.2
0.3
Position
3220500
3221000
3221500
3222000
Group
DYT28_Pathogenic
Control

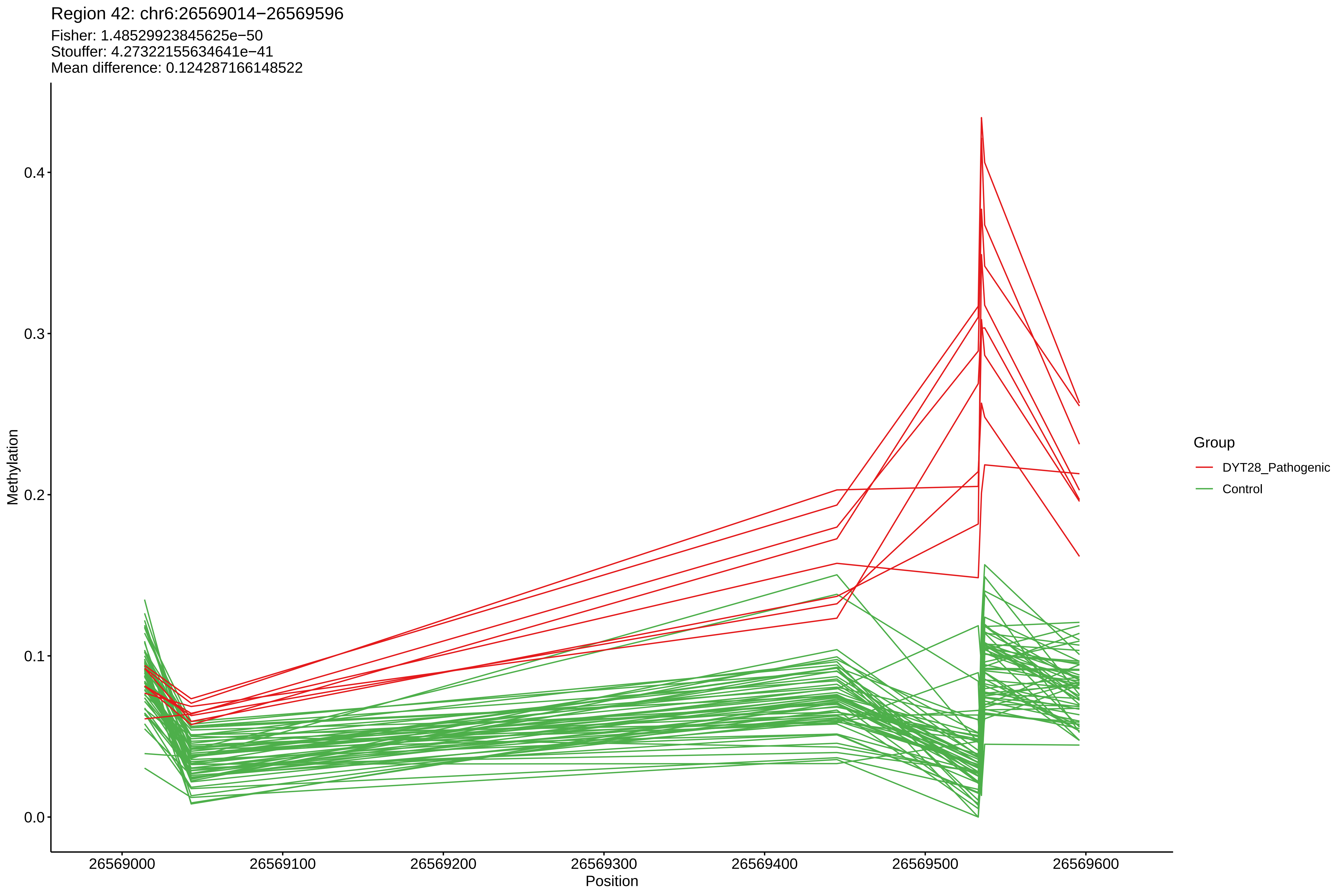

Region 42: chr6:26569014−26569596
Fisher: 1.48529923845625e−50
Stouffer: 4.27322155634641e−41
Mean difference: 0.124287166148522
Methylation
0.0
0.1
0.2
0.3
0.4
26569000
26569100
26569200
26569300
26569400
26569500
26569600
Position
Group
DYT28_Pathogenic
Control

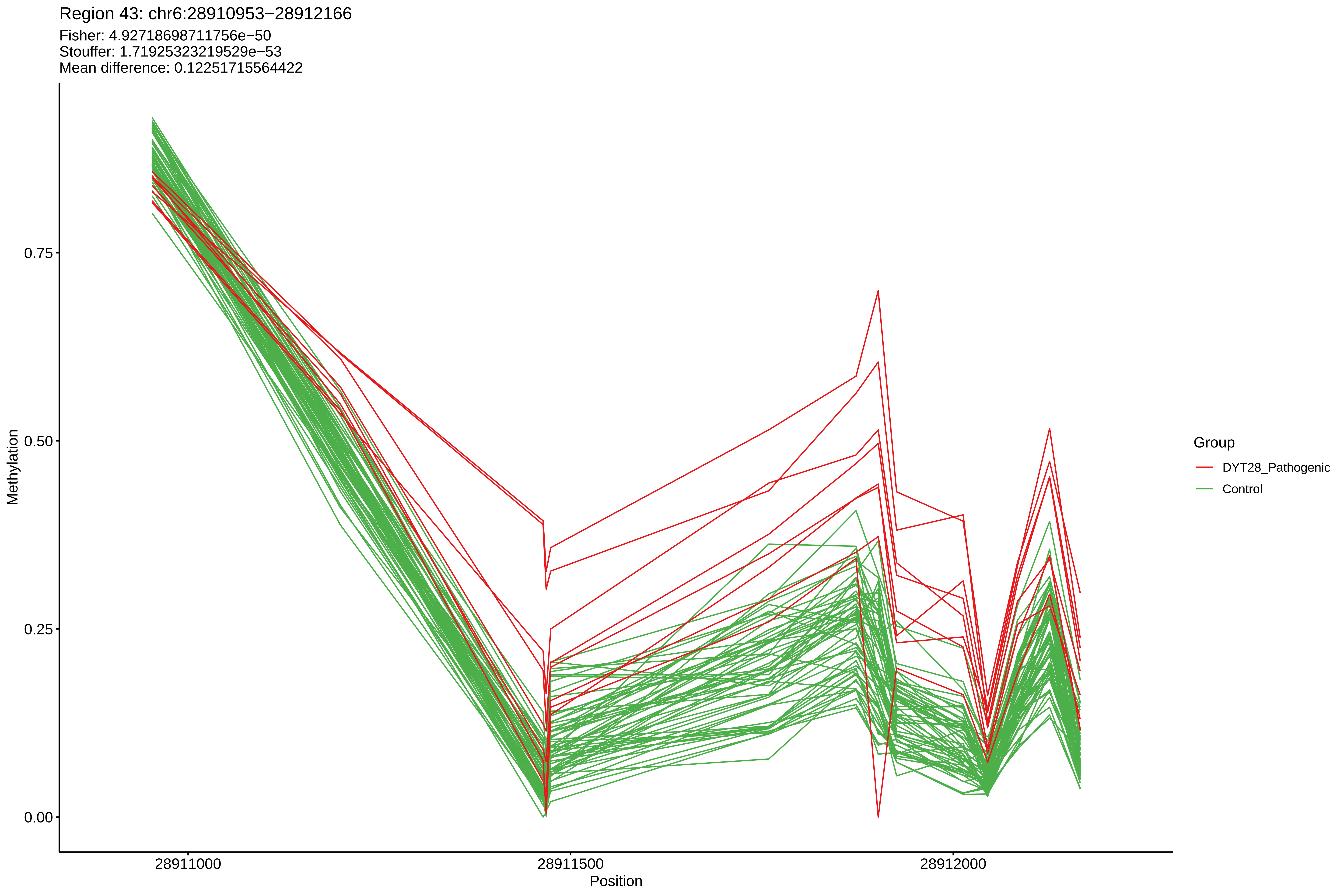

Region 43: chr6:28910953−28912166
Fisher: 4.92718698711756e−50
Stouffer: 1.71925323219529e−53
Mean difference: 0.12251715564422
Methylation
0.75
0.50
0.25
0.00
28911000
28911500
28912000
Position
Group
DYT28_Pathogenic
Control

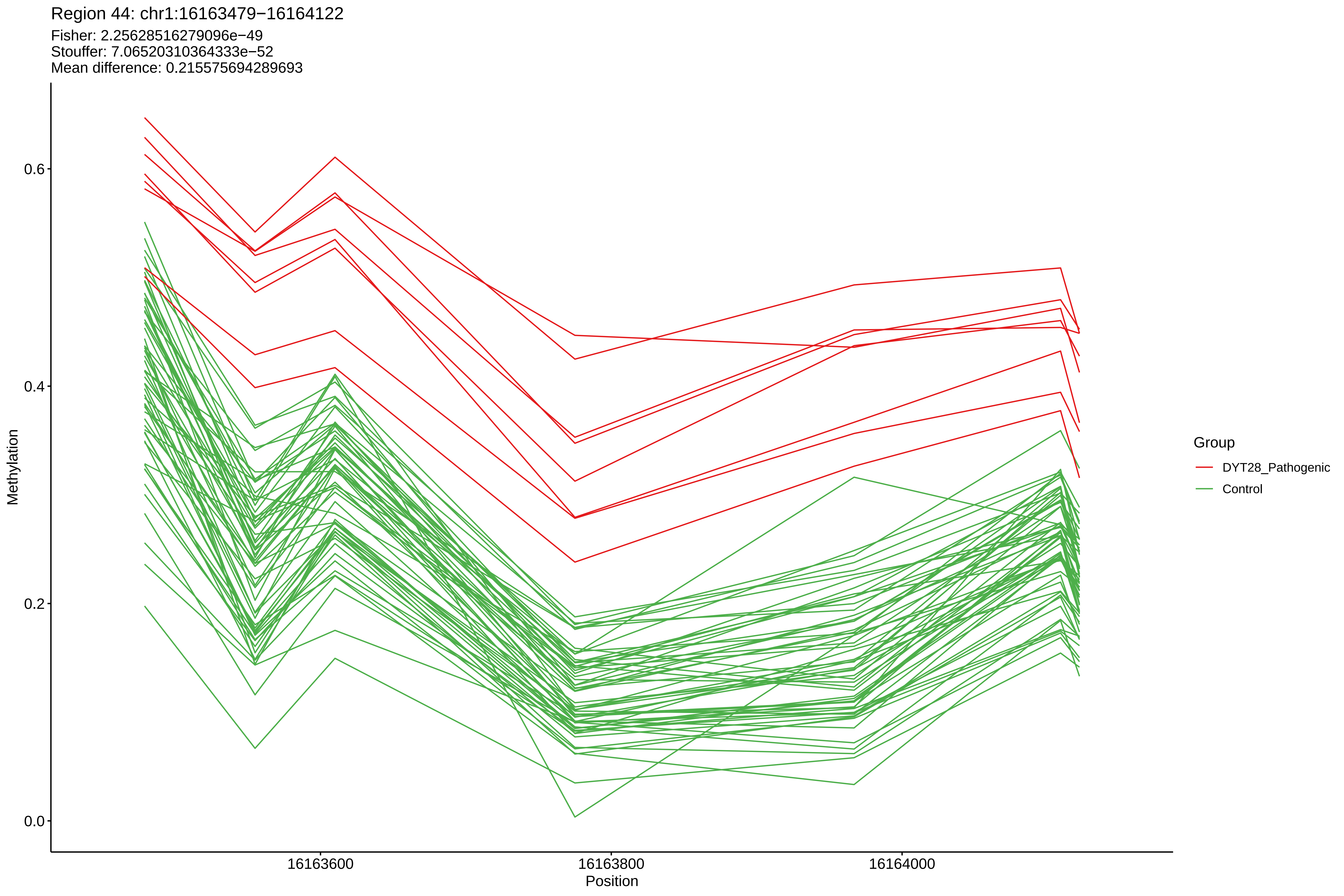
Region 44: chr1:16163479−16164122
Fisher: 2.25628516279096e−49
Stouffer: 7.06520310364333e−52
Mean difference: 0.215575694289693
Methylation
0.0
0.2
0.4
0.6
Position
16163600
16163800
16164000
Group
DYT28_Pathogenic
Control

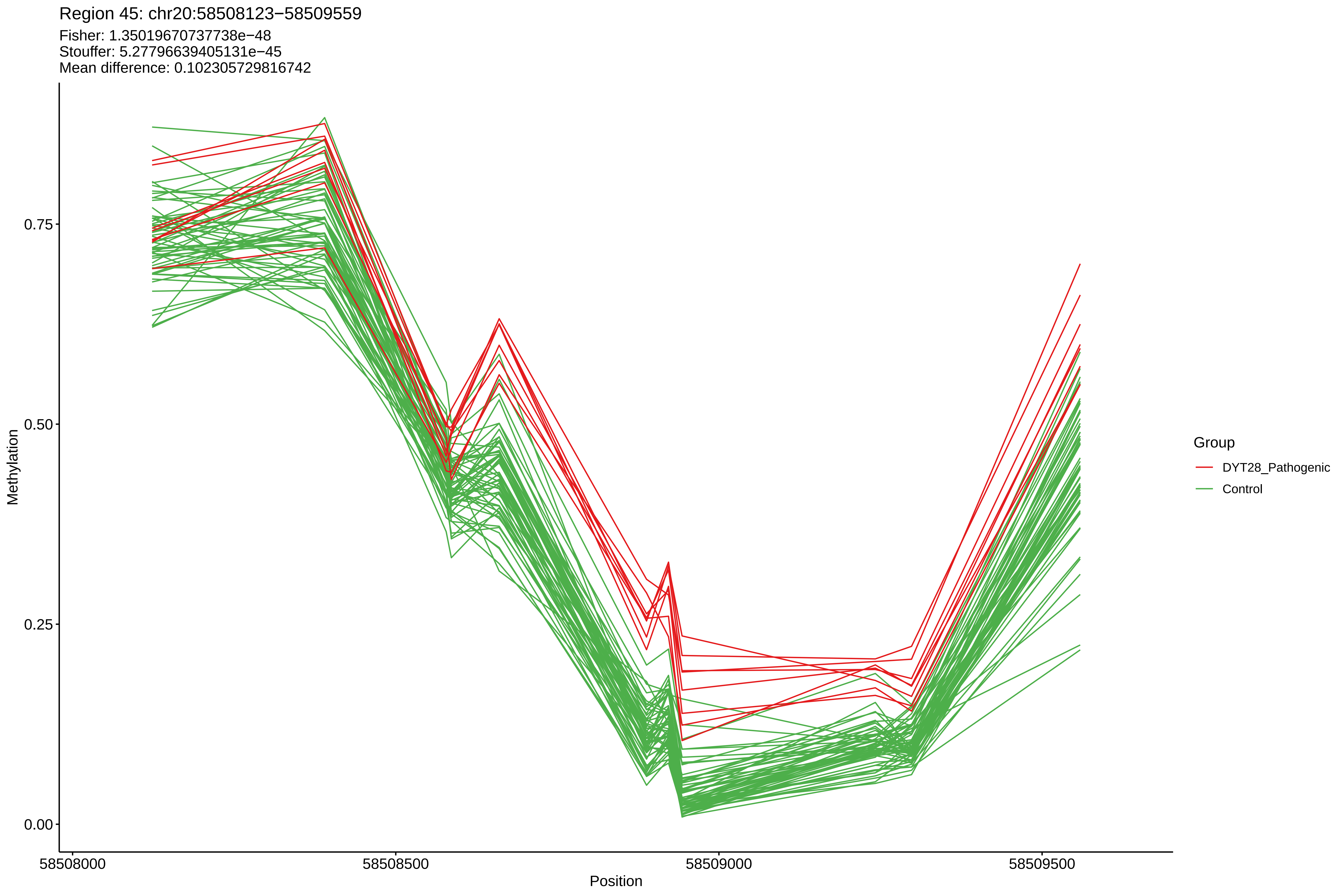
Region 45: chr20:58508123−58509559
Fisher: 1.35019670737738e−48
Stouffer: 5.27796639405131e−45
Mean difference: 0.102305729816742
Methylation
0.00
0.25
0.50
0.75
Position
58508000
58508500
58509000
58509500
Group
DYT28_Pathogenic
Control

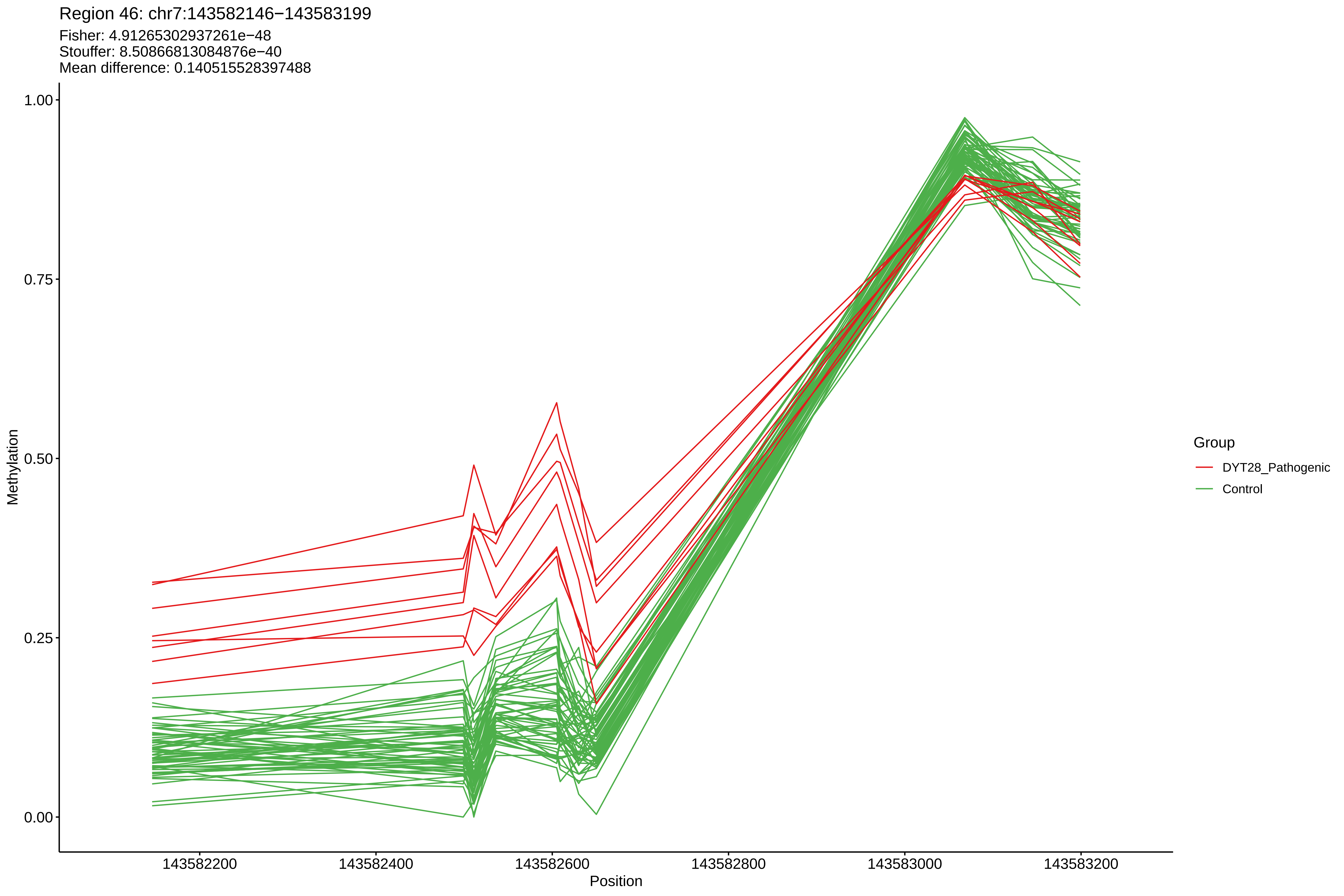

Region 46: chr7:143582146−143583199
Fisher: 4.91265302937261e−48
Stouffer: 8.50866813084876e−40
Mean difference: 0.140515528397488
Methylation
1.00
0.75
0.50
0.25
0.00
143582200
143582400
143582600
143582800
143583000
143583200
Position
Group
DYT28_Pathogenic
Control

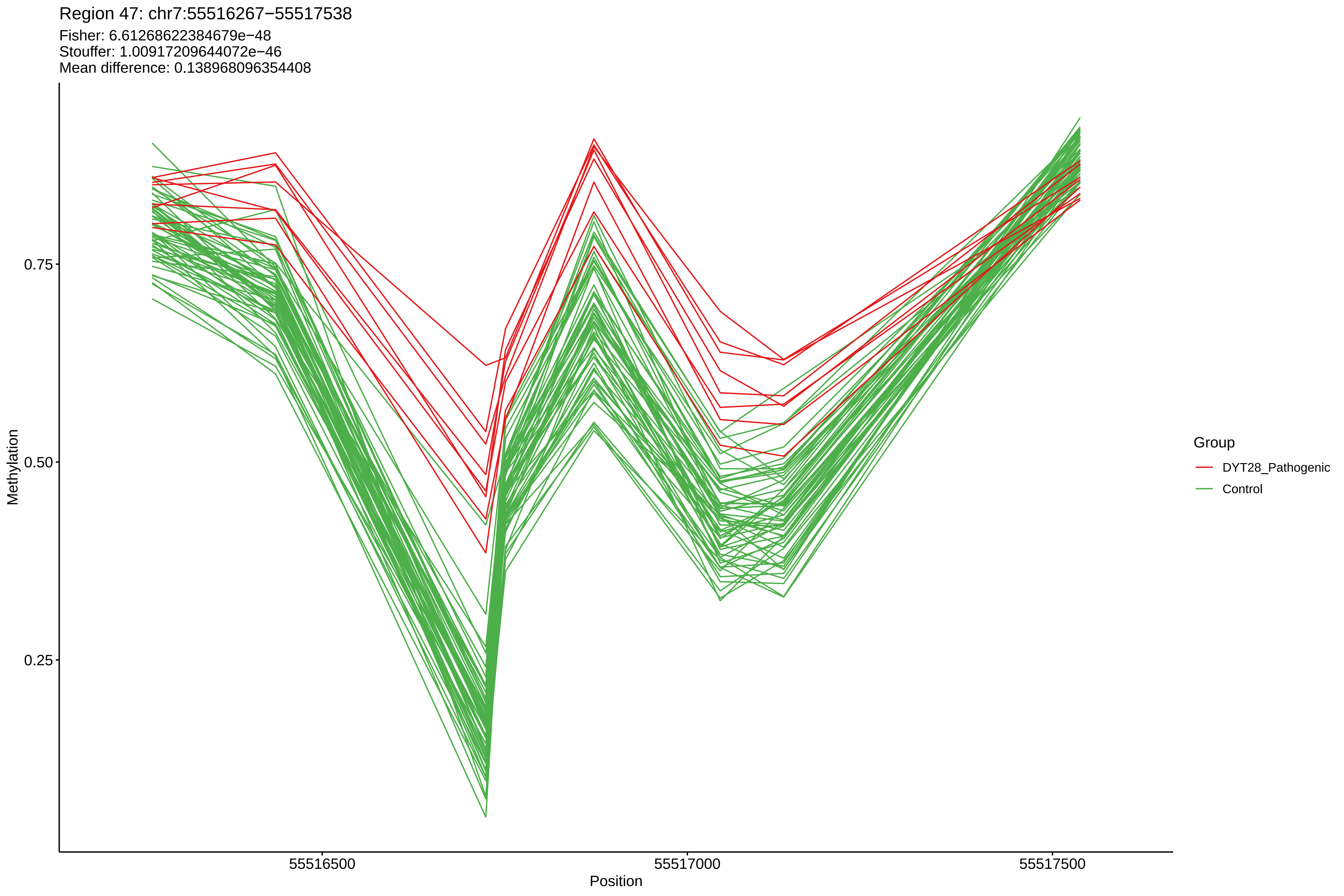
Region 47: chr7:55516267−55517538
Fisher: 6.61268622384679e−48
Stouffer: 1.00917209644072e−46
Mean difference: 0.138968096354408
Methylation
0.75
0.50
0.25
55516500
55517000
55517500
Position
Group
DYT28_Pathogenic
Control

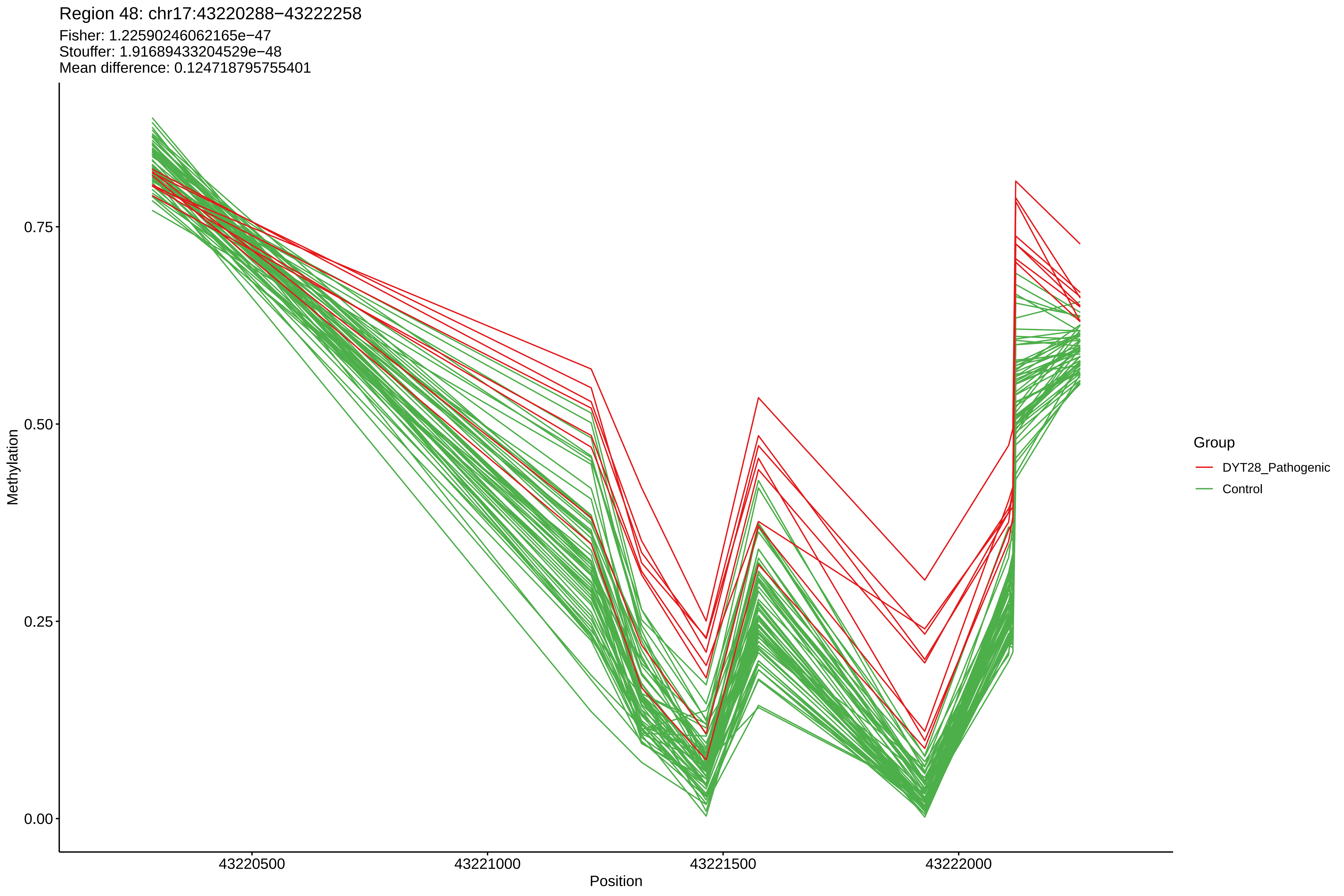
Region 48: chr17:43220288−43222258
Fisher: 1.22590246062165e−47
Stouffer: 1.91689433204529e−48
Mean difference: 0.124718795755401
Methylation
0.75
0.50
0.25
0.00
43220500
43221000
43221500
43222000
Position
Group
DYT28_Pathogenic
Control

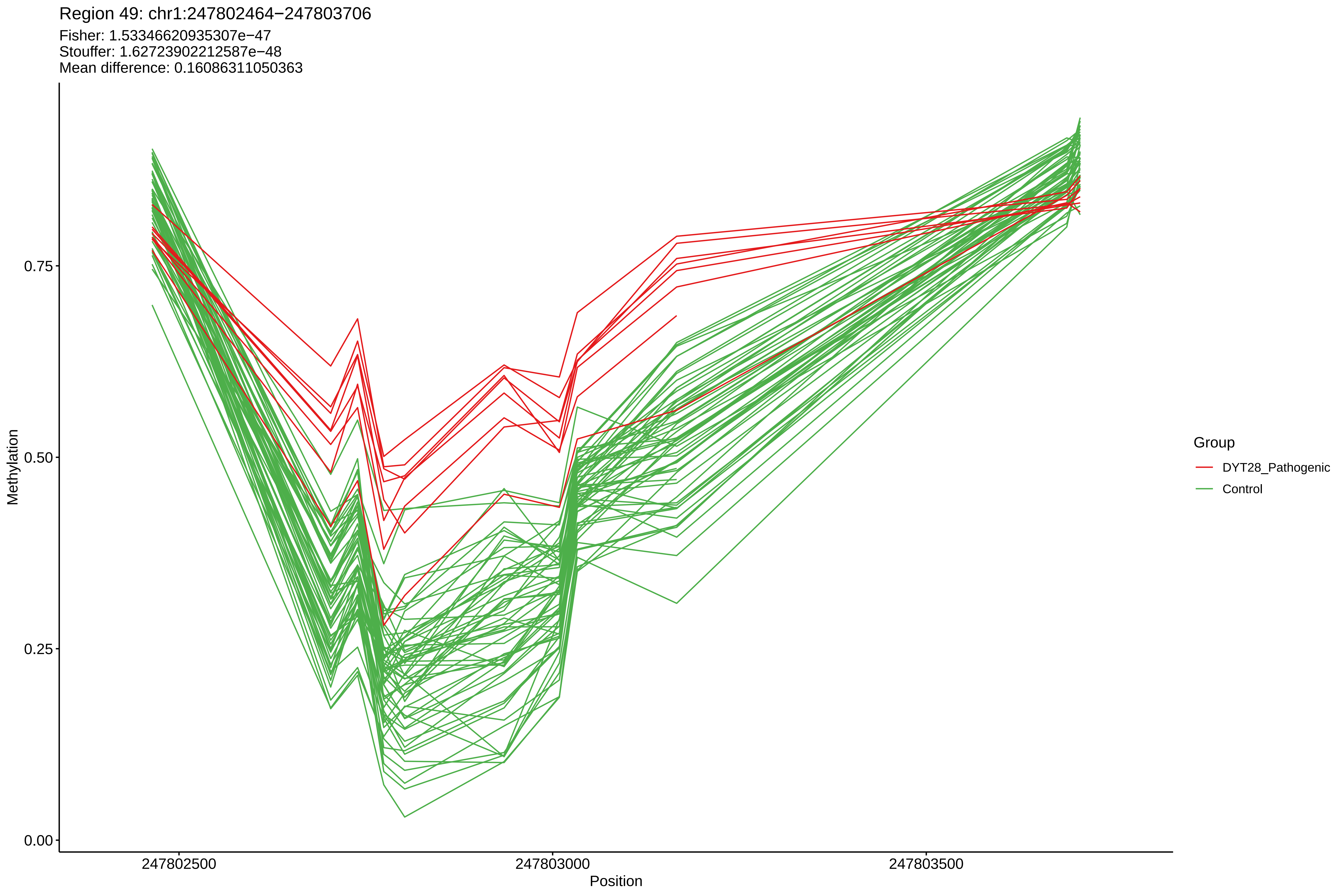
Region 49: chr1:247802464−247803706
Fisher: 1.53346620935307e−47
Stouffer: 1.62723902212587e−48
Mean difference: 0.16086311050363
Methylation
0.75
0.50
0.25
0.00
247802500
247803000
247803500
Position
Group
DYT28_Pathogenic
Control

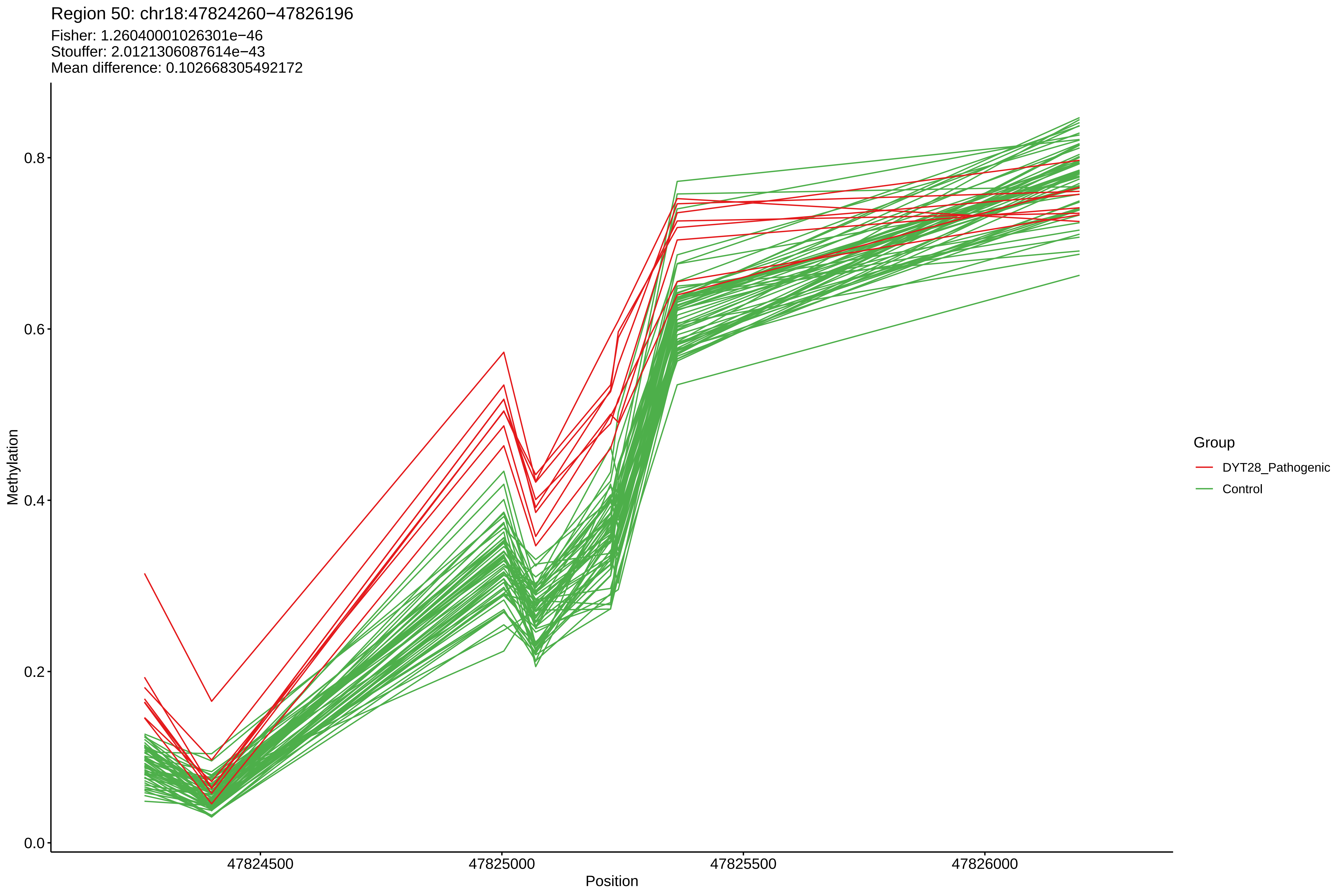

Region 50: chr18:47824260−47826196
Fisher: 1.26040001026301e−46
Stouffer: 2.0121306087614e−43
Mean difference: 0.102668305492172
Methylation
0.0
0.2
0.4
0.6
0.8
Position
47824500
47825000
47825500
47826000
Group
DYT28_Pathogenic
Control

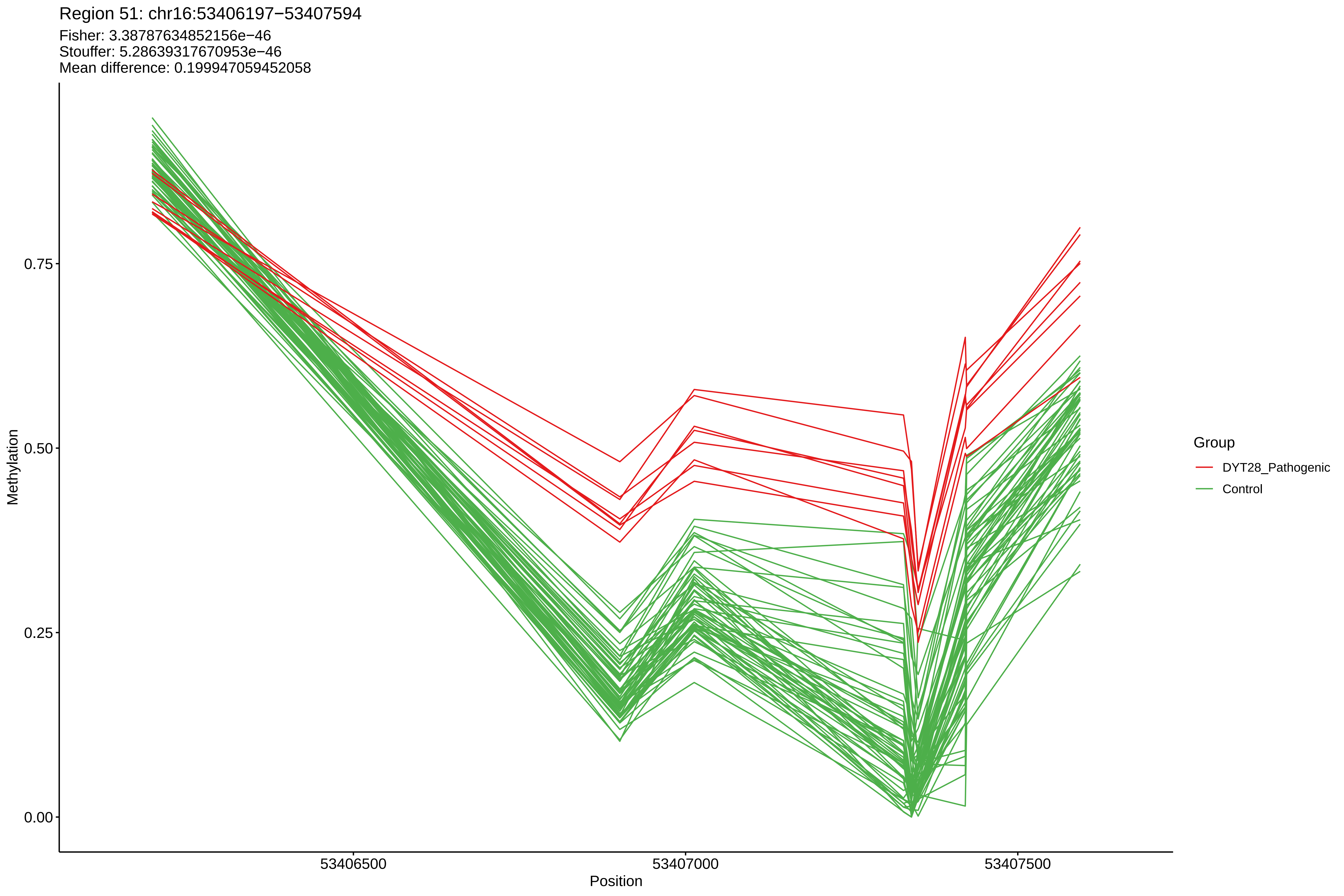
Region 51: chr16:53406197−53407594
Fisher: 3.38787634852156e−46
Stouffer: 5.28639317670953e−46
Mean difference: 0.199947059452058
Methylation
0.75
0.50
0.25
0.00
53406500
53407000
53407500
Position
Group
DYT28_Pathogenic
Control

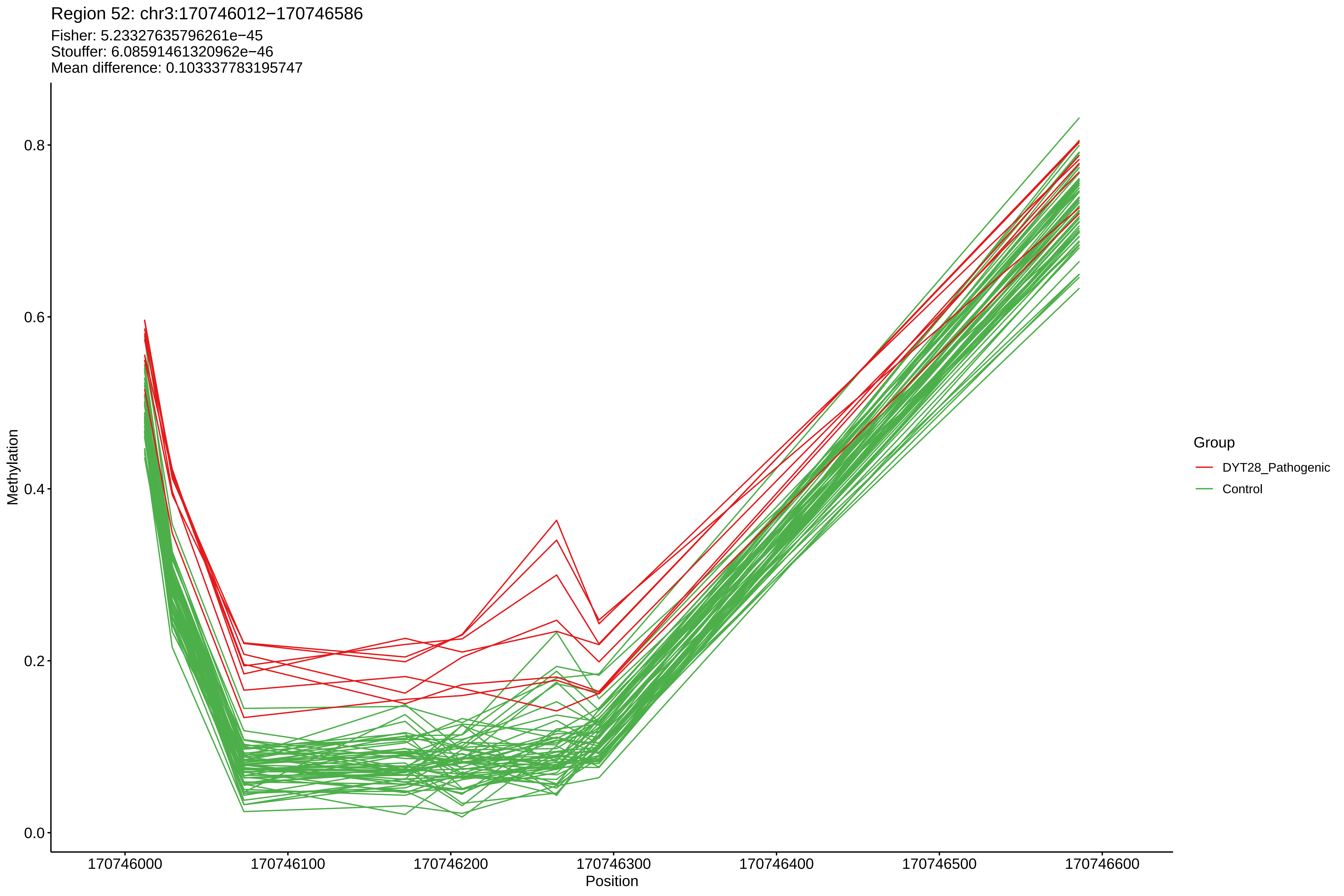
Region 52: chr3:170746012−170746586
Fisher: 5.23327635796261e−45
Stouffer: 6.08591461320962e−46
Mean difference: 0.103337783195747
Methylation
0.0
0.2
0.4
0.6
0.8
170746000
170746100
170746200
170746300
170746400
170746500
170746600
Position
Group
DYT28_Pathogenic
Control

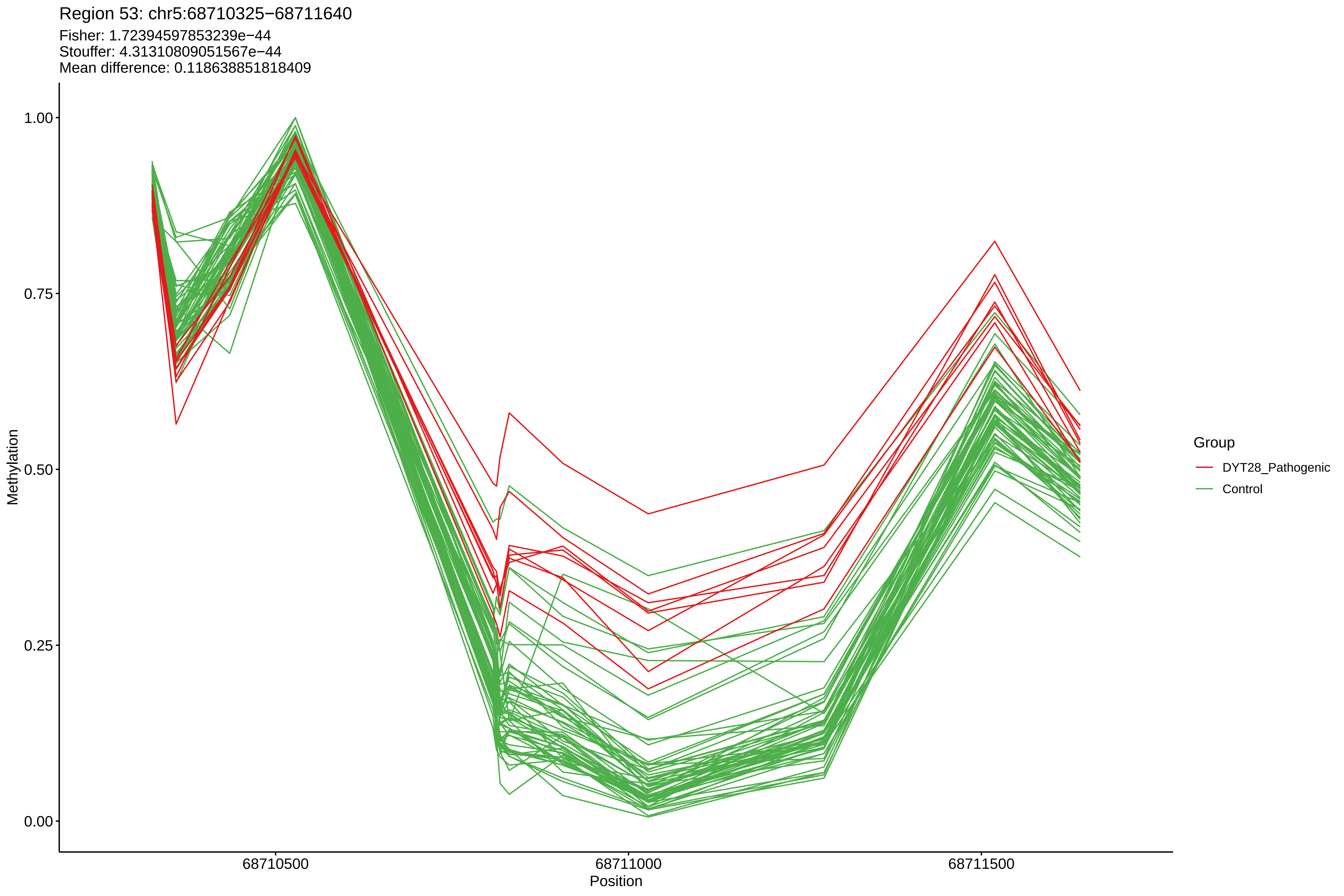
Region 53: chr5:68710325−68711640
Fisher: 1.72394597853239e−44
Stouffer: 4.31310809051567e−44
Mean difference: 0.118638851818409
Methylation
1.00
0.75
0.50
0.25
0.00
68710500
68711000
68711500
Position
Group
DYT28_Pathogenic
Control

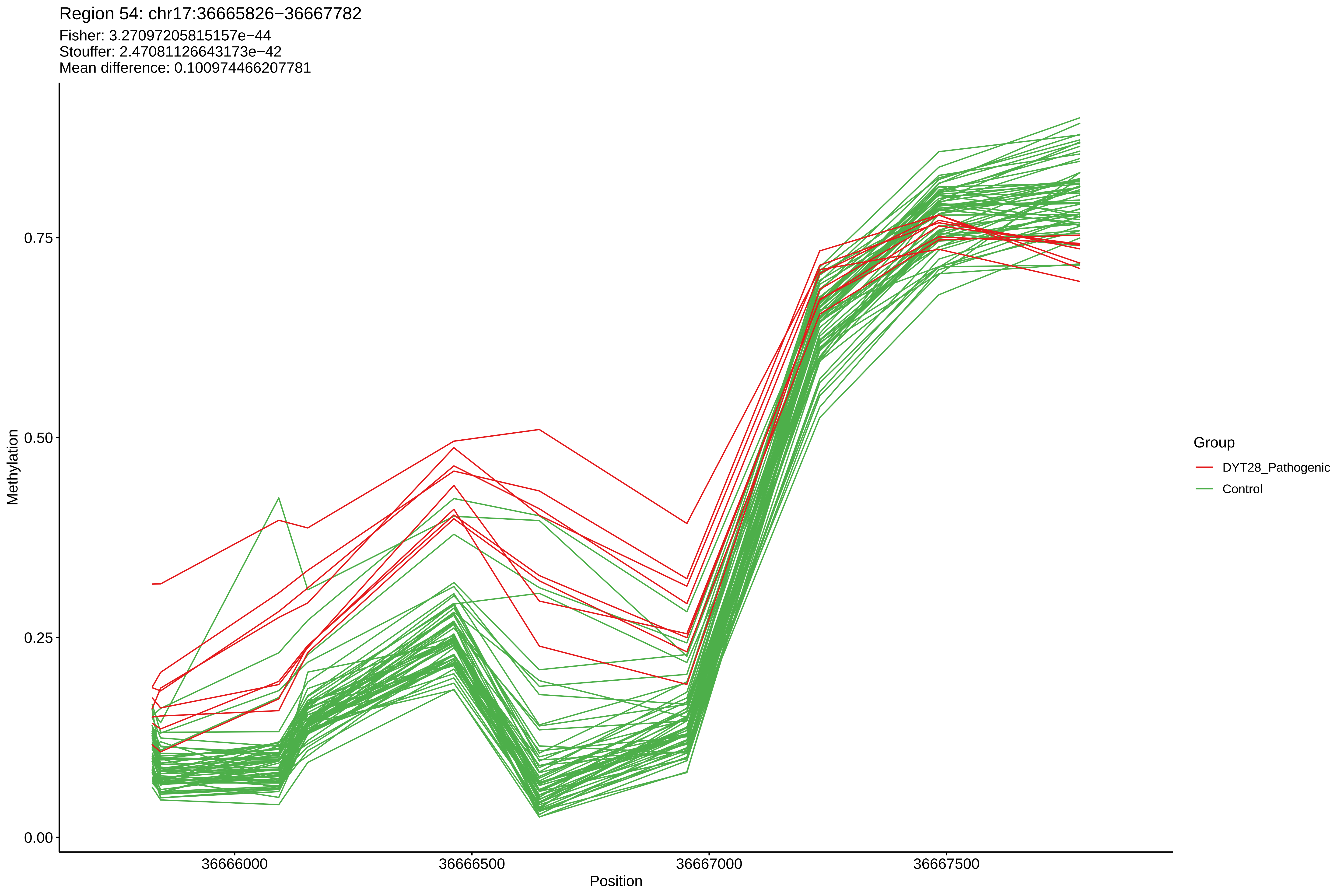
Region 54: chr17:36665826−36667782
Fisher: 3.27097205815157e−44
Stouffer: 2.47081126643173e−42
Mean difference: 0.100974466207781
Methylation
0.00
0.25
0.50
0.75
36666000
36666500
36667000
36667500
Position
Group
DYT28_Pathogenic
Control

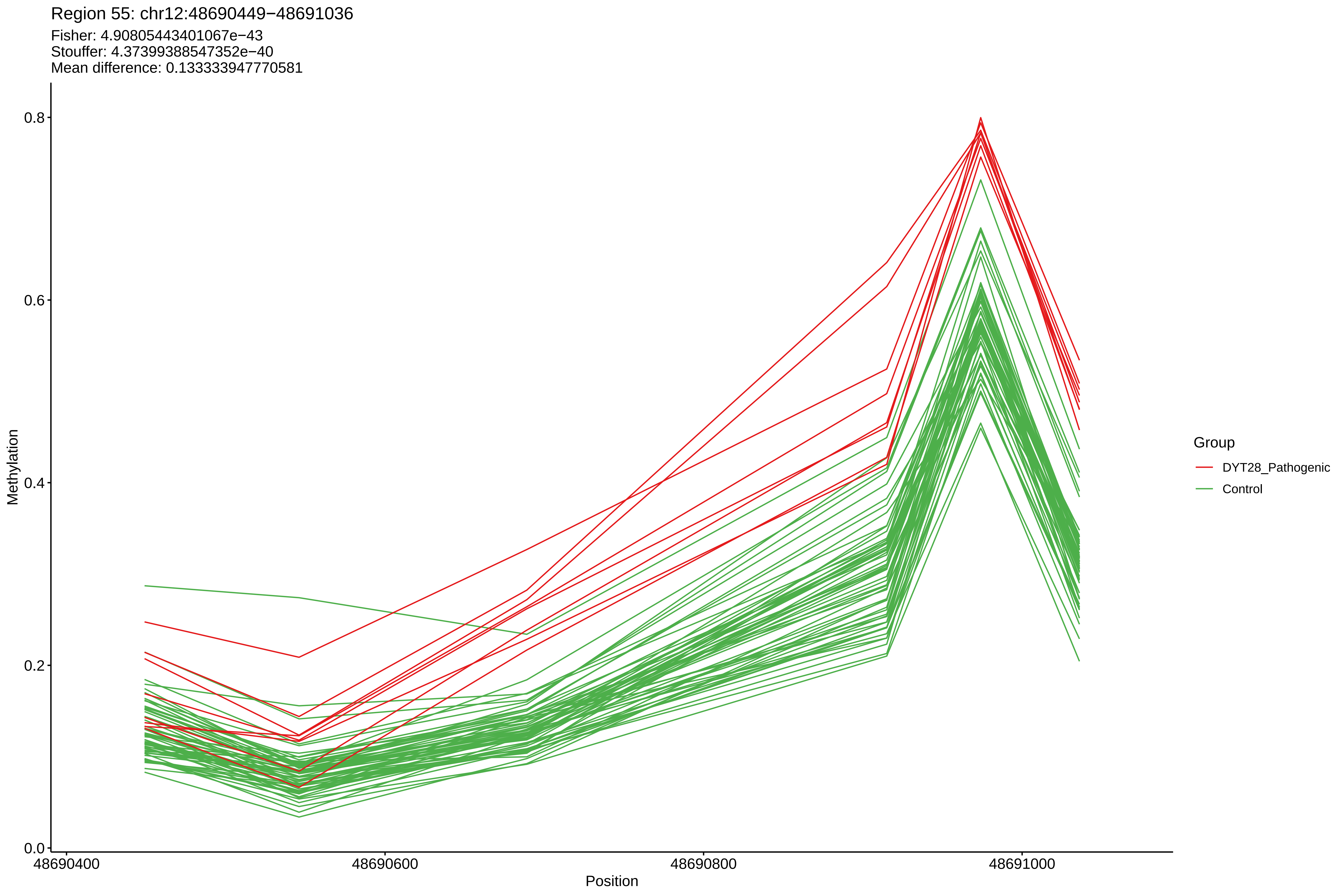

Region 55: chr12:48690449−48691036
Fisher: 4.90805443401067e−43
Stouffer: 4.37399388547352e−40
Mean difference: 0.133333947770581
Methylation
0.0
0.2
0.4
0.6
0.8
48690400
48690600
48690800
48691000
Position
Group
DYT28_Pathogenic
Control

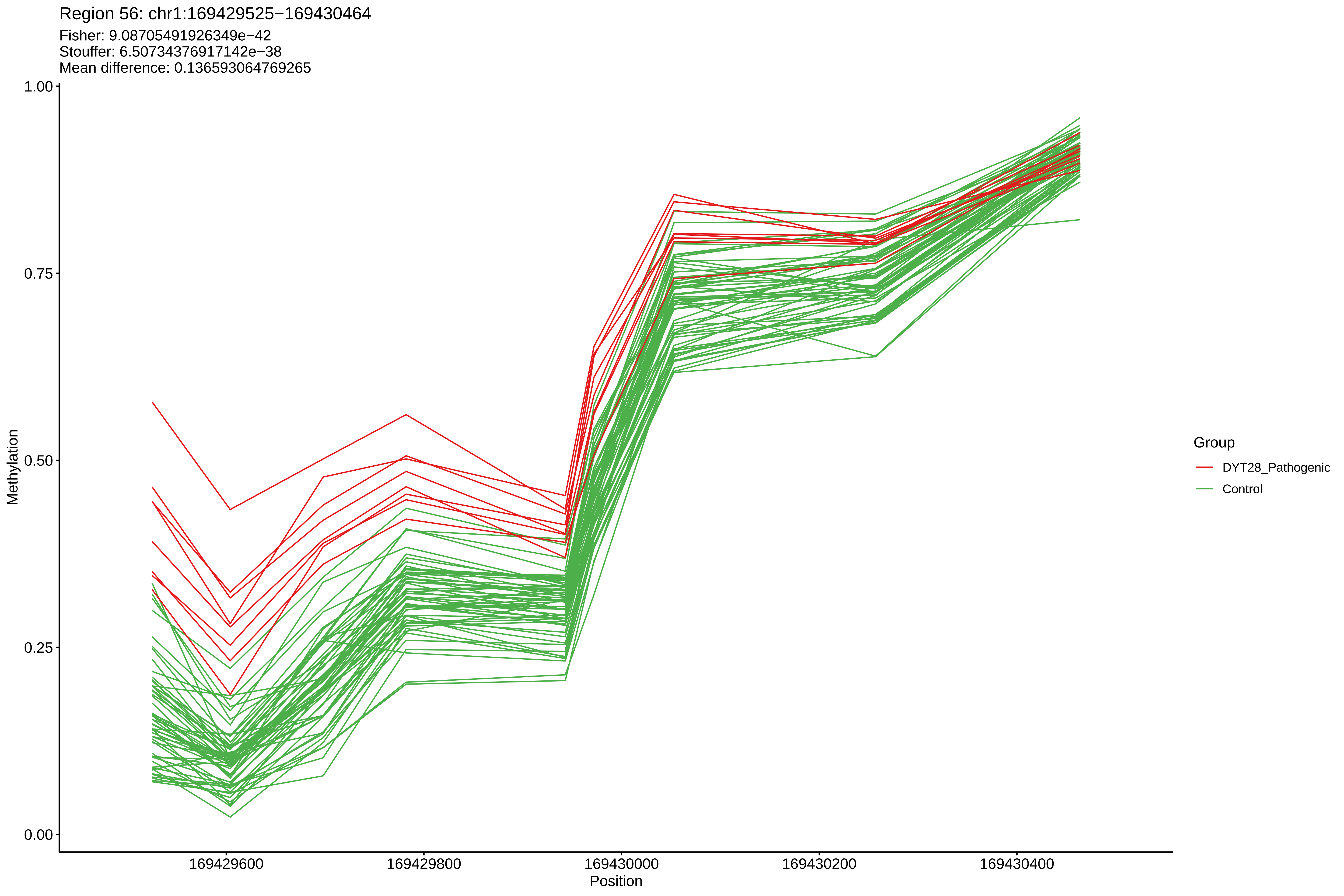

Region 56: chr1:169429525−169430464
Fisher: 9.08705491926349e−42
Stouffer: 6.50734376917142e−38
Mean difference: 0.136593064769265
Methylation
1.00
0.75
0.50
0.25
0.00
169429600
169429800
169430000
169430200
169430400
Position
Group
DYT28_Pathogenic
Control

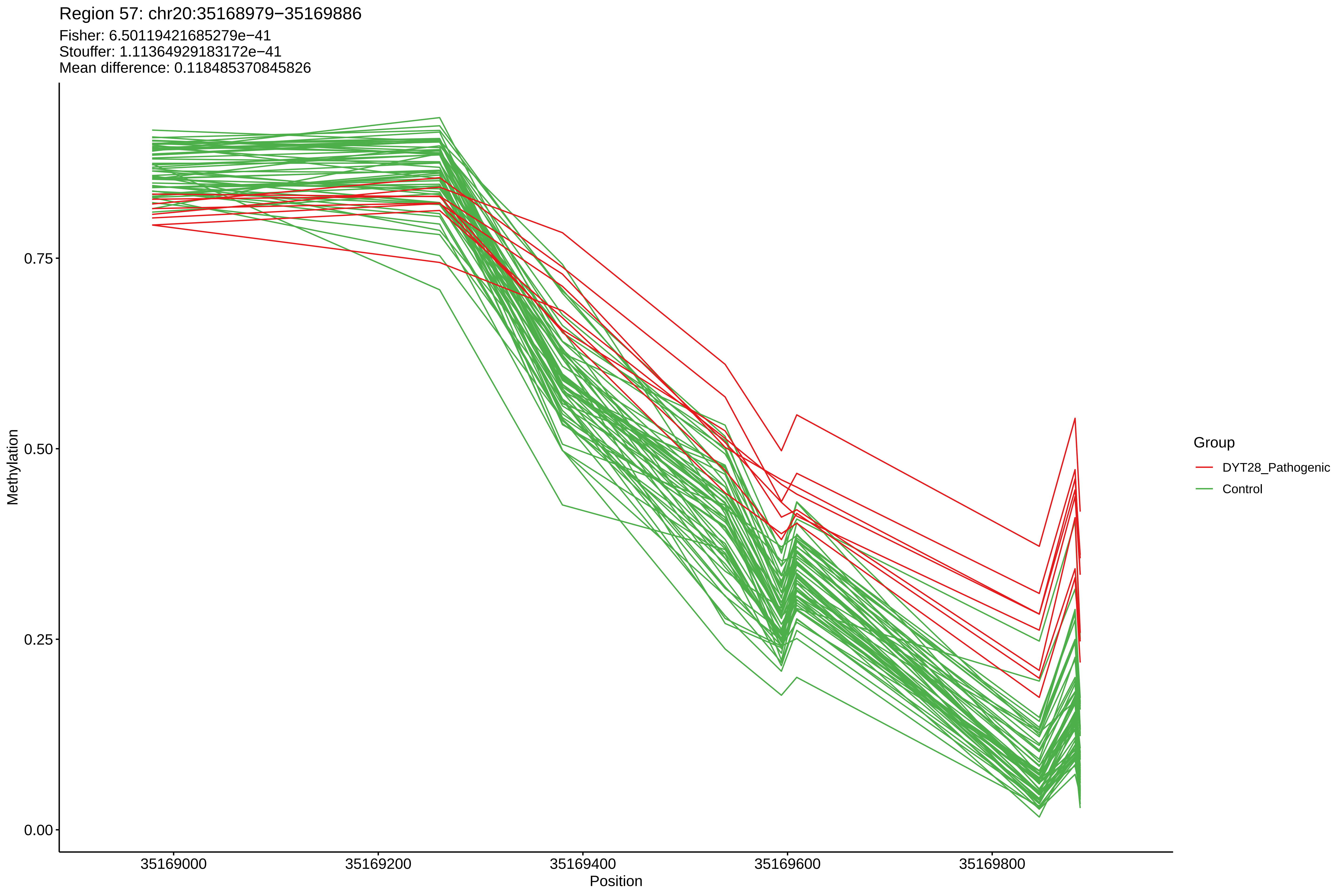

Region 57: chr20:35168979−35169886
Fisher: 6.50119421685279e−41
Stouffer: 1.11364929183172e−41
Mean difference: 0.118485370845826
Methylation
0.00
0.25
0.50
0.75
Position
35169000
35169200
35169400
35169600
35169800
Group
DYT28_Pathogenic
Control

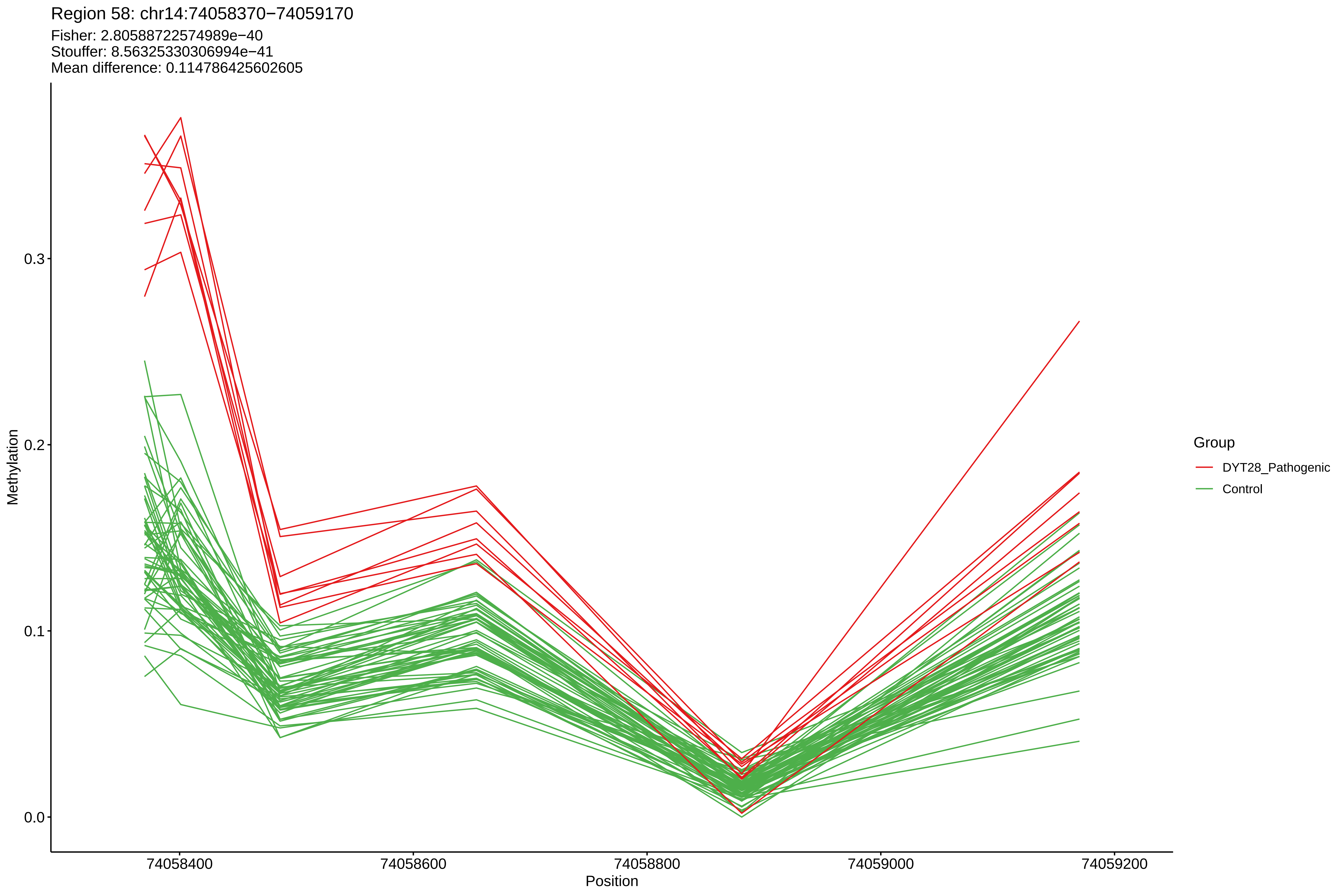
Region 58: chr14:74058370−74059170
Fisher: 2.80588722574989e−40
Stouffer: 8.56325330306994e−41
Mean difference: 0.114786425602605
Methylation
0.0
0.1
0.2
0.3
74058400
74058600
74058800
74059000
74059200
Position
Group
DYT28_Pathogenic
Control

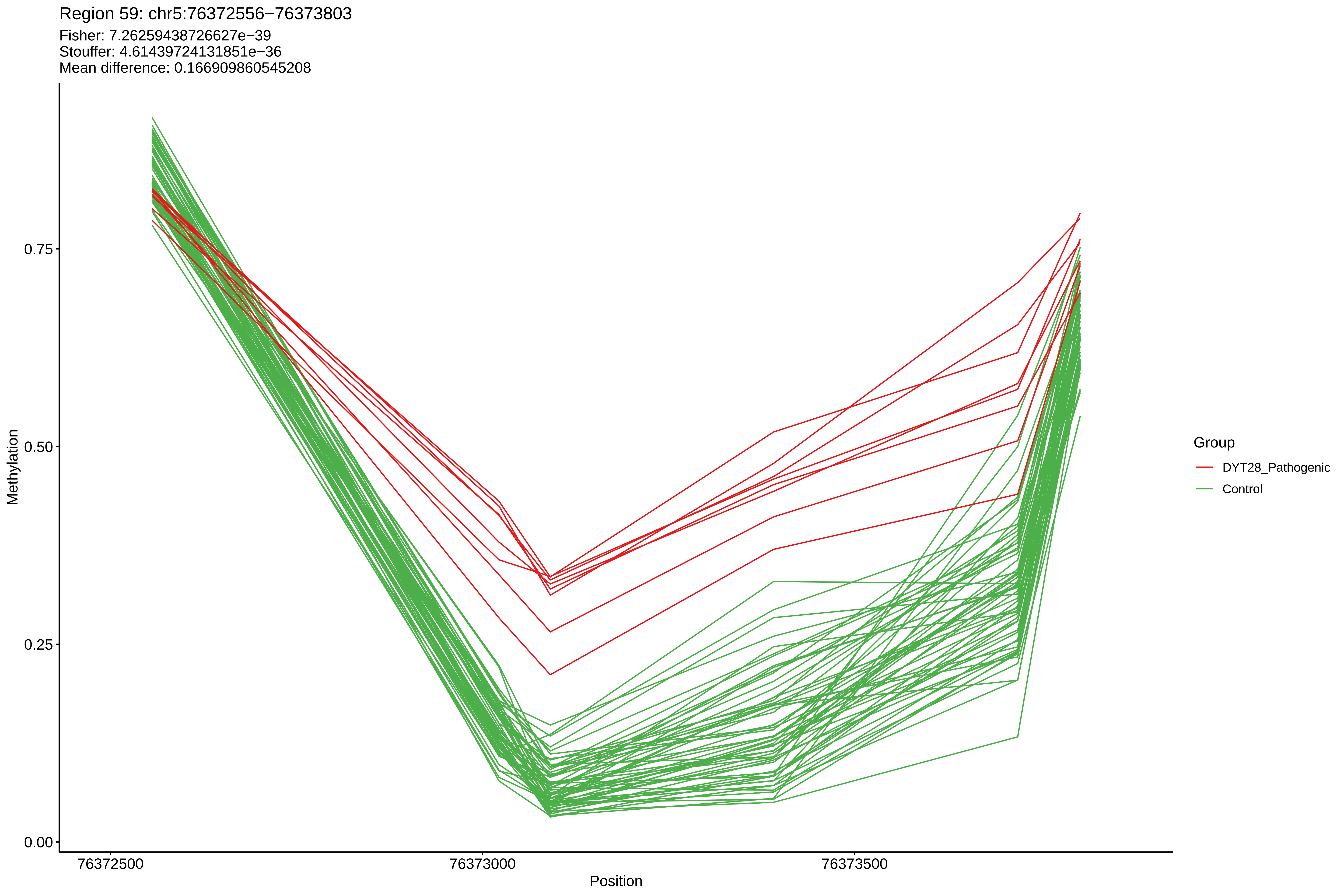
Region 59: chr5:76372556−76373803
Fisher: 7.26259438726627e−39
Stouffer: 4.61439724131851e−36
Mean difference: 0.166909860545208
Methylation
0.75
0.50
0.25
0.00
76372500
76373000
76373500
Position
Group
DYT28_Pathogenic
Control

Region 60: chr6:144416220−144418218

Fisher: 1.20686257185167e−38

Stouffer: 5.50474809588906e−18

Mean difference: 0.108126350420021

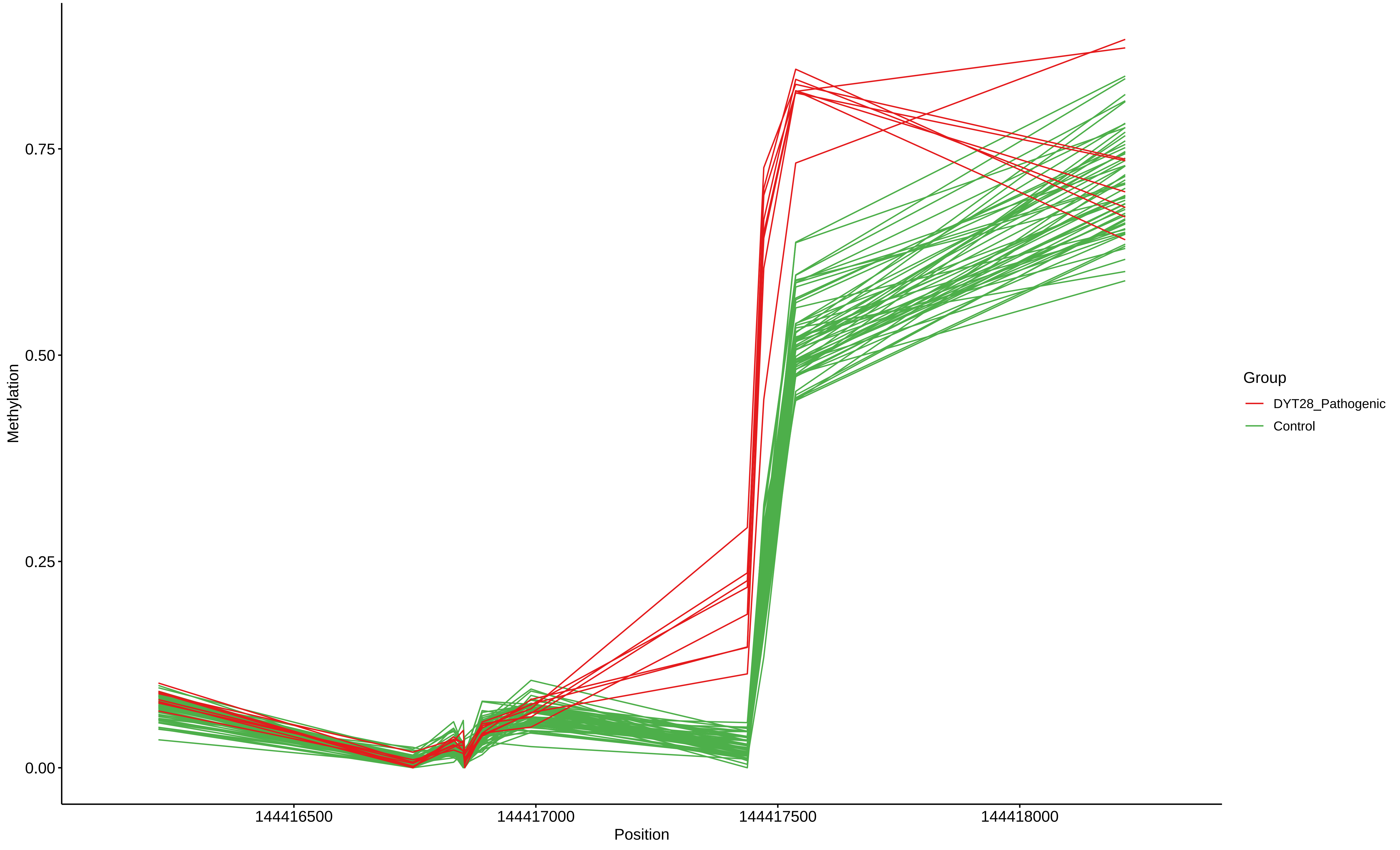

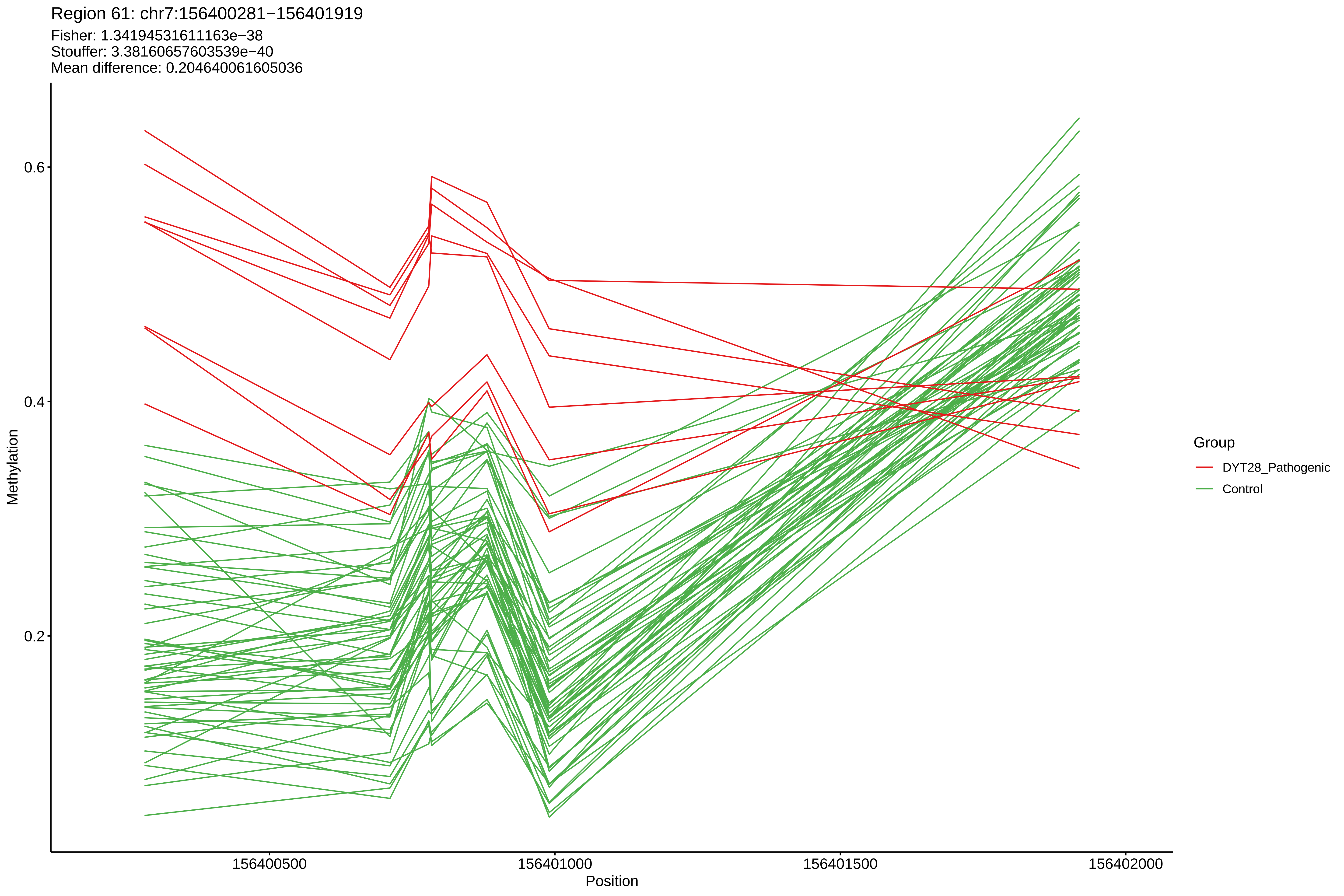
Region 61: chr7:156400281−156401919
Fisher: 1.34194531611163e−38
Stouffer: 3.38160657603539e−40
Mean difference: 0.204640061605036
0.6
0.4
0.2
Methylation
156400500
156401000
156401500
156402000
Position
Group
DYT28_Pathogenic
Control

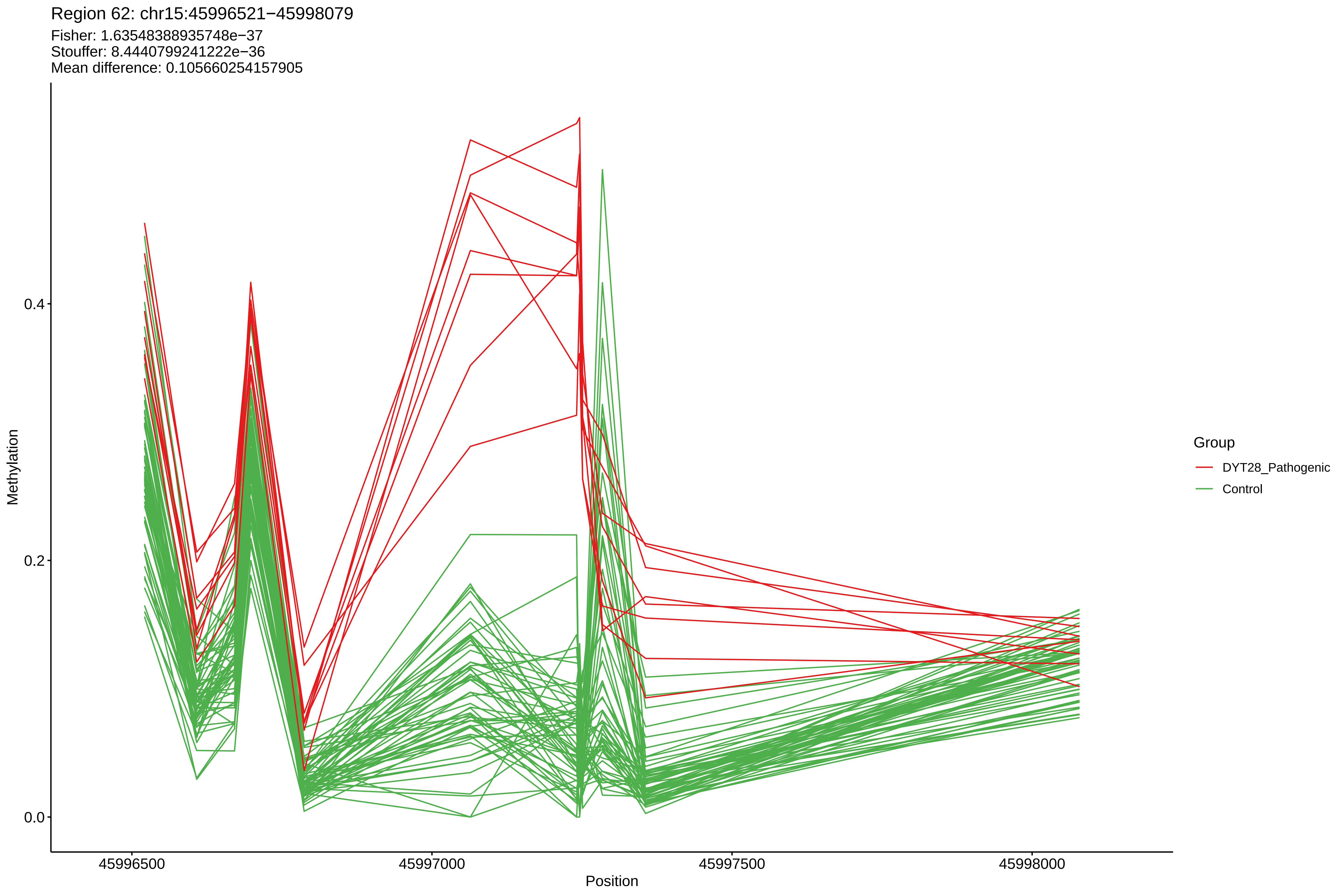

Region 62: chr15:45996521−45998079
Fisher: 1.63548388935748e−37
Stouffer: 8.4440799241222e−36
Mean difference: 0.105660254157905
Methylation
0.4
0.2
0.0
45996500
45997000
45997500
45998000
Position
Group
DYT28_Pathogenic
Control

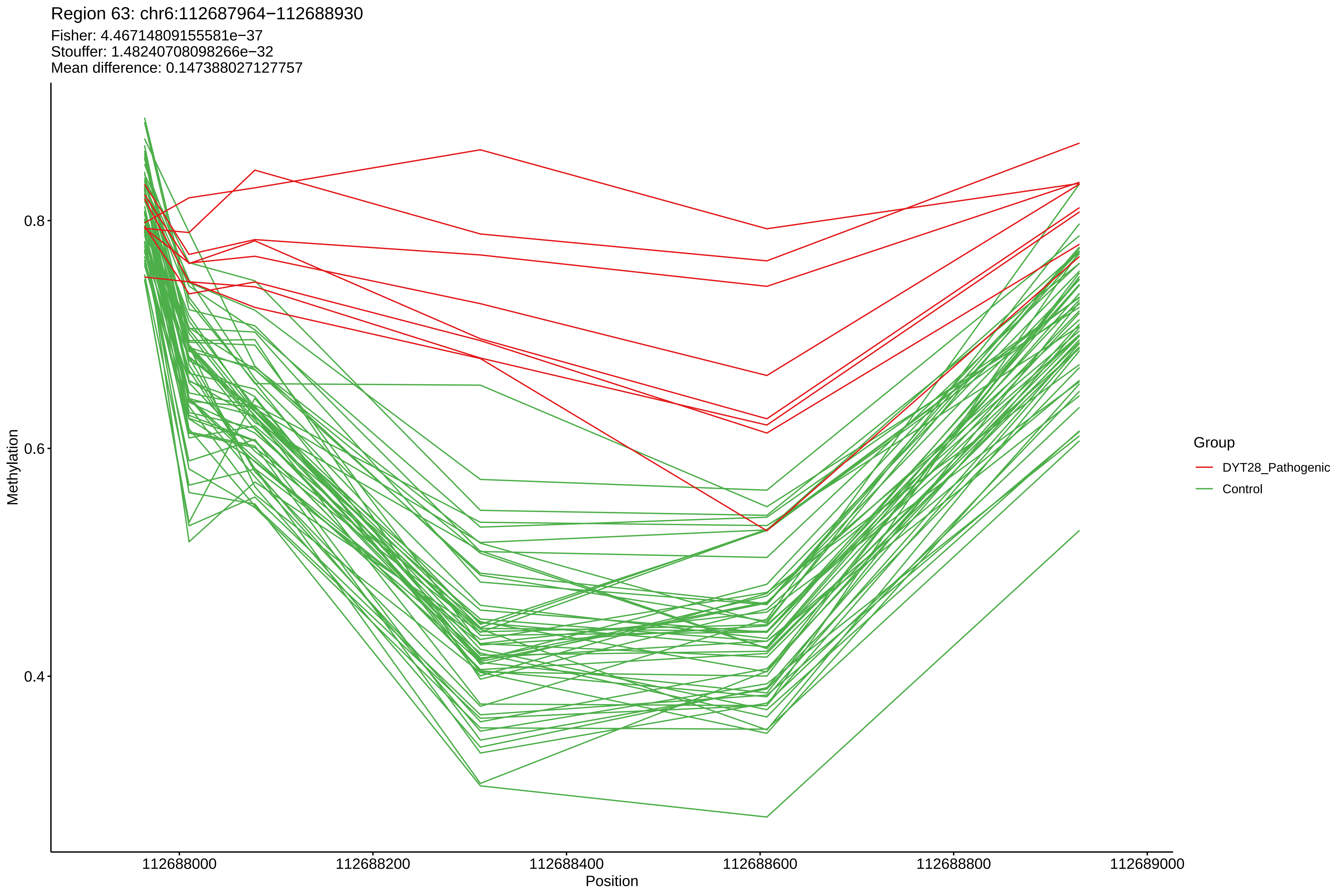
Region 63: chr6:112687964−112688930
Fisher: 4.46714809155581e−37
Stouffer: 1.48240708098266e−32
Mean difference: 0.147388027127757
Methylation
0.8
0.6
0.4
112688000
112688200
112688400
112688600
112688800
112689000
Position
Group
DYT28_Pathogenic
Control

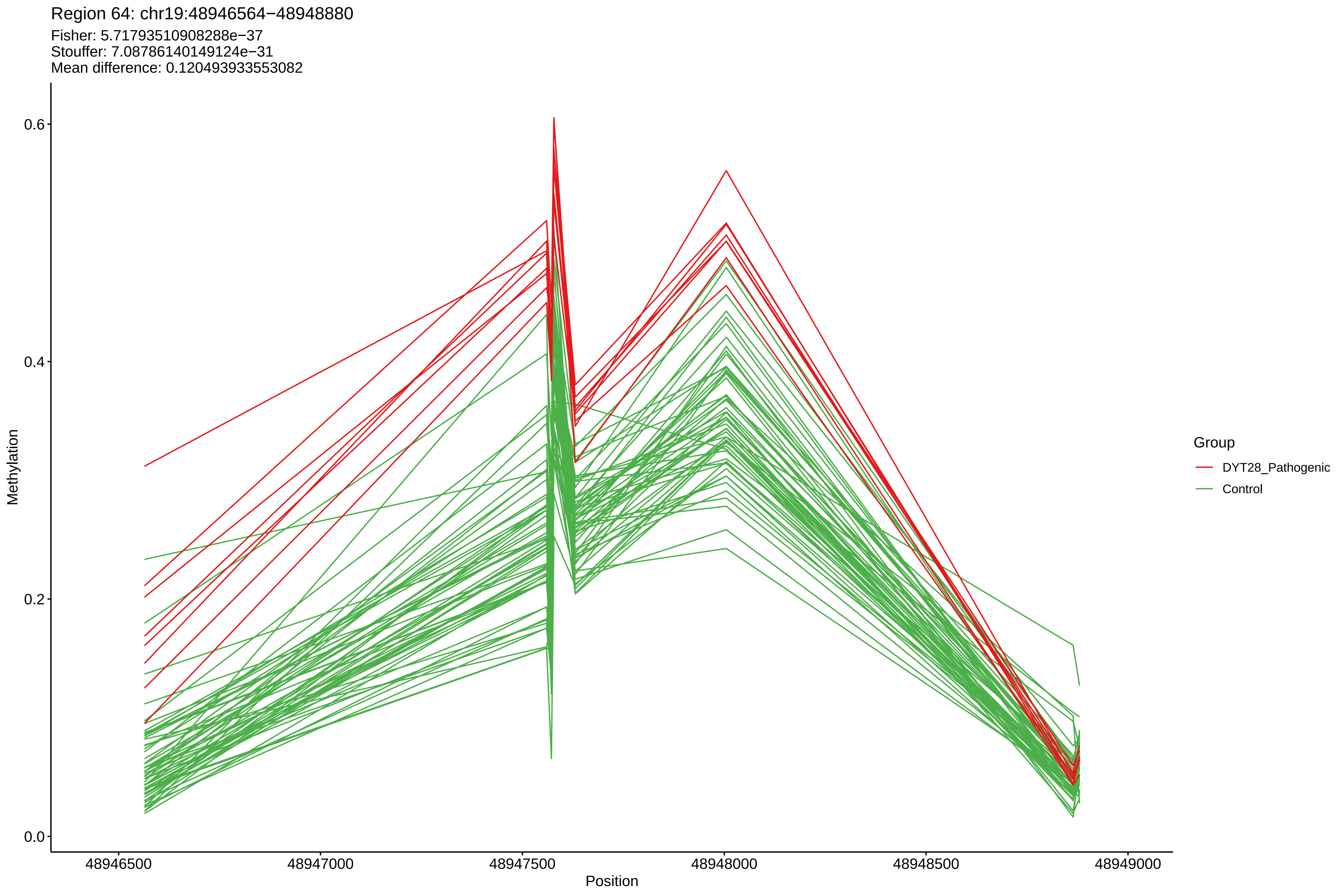
Region 64: chr19:48946564−48948880
Fisher: 5.71793510908288e−37
Stouffer: 7.08786140149124e−31
Mean difference: 0.120493933553082
Methylation
0.0
0.2
0.4
0.6
Position
48946500
48947000
48947500
48948000
48948500
48949000
Group
DYT28_Pathogenic
Control

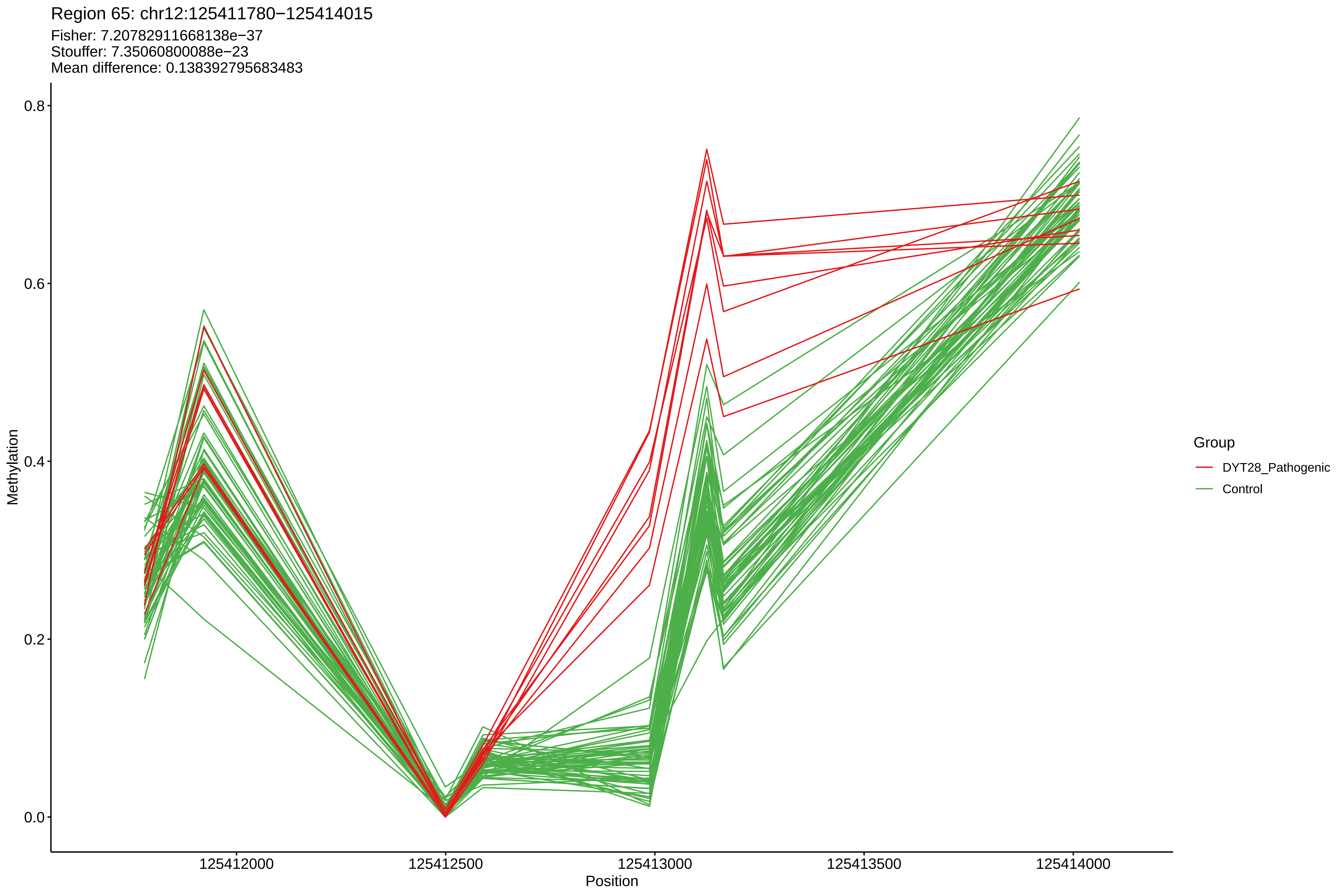
Region 65: chr12:125411780−125414015
Fisher: 7.20782911668138e−37
Stouffer: 7.35060800088e−23
Mean difference: 0.138392795683483
Methylation
0.0
0.2
0.4
0.6
0.8
Position
125412000
125412500
125413000
125413500
125414000
Group
DYT28_Pathogenic
Control

Region 66: chr18:14747082−14748439
Fisher: 1.32330652907332e−36
Stouffer: 4.748155688846e−38
Mean difference: 0.122065412924441

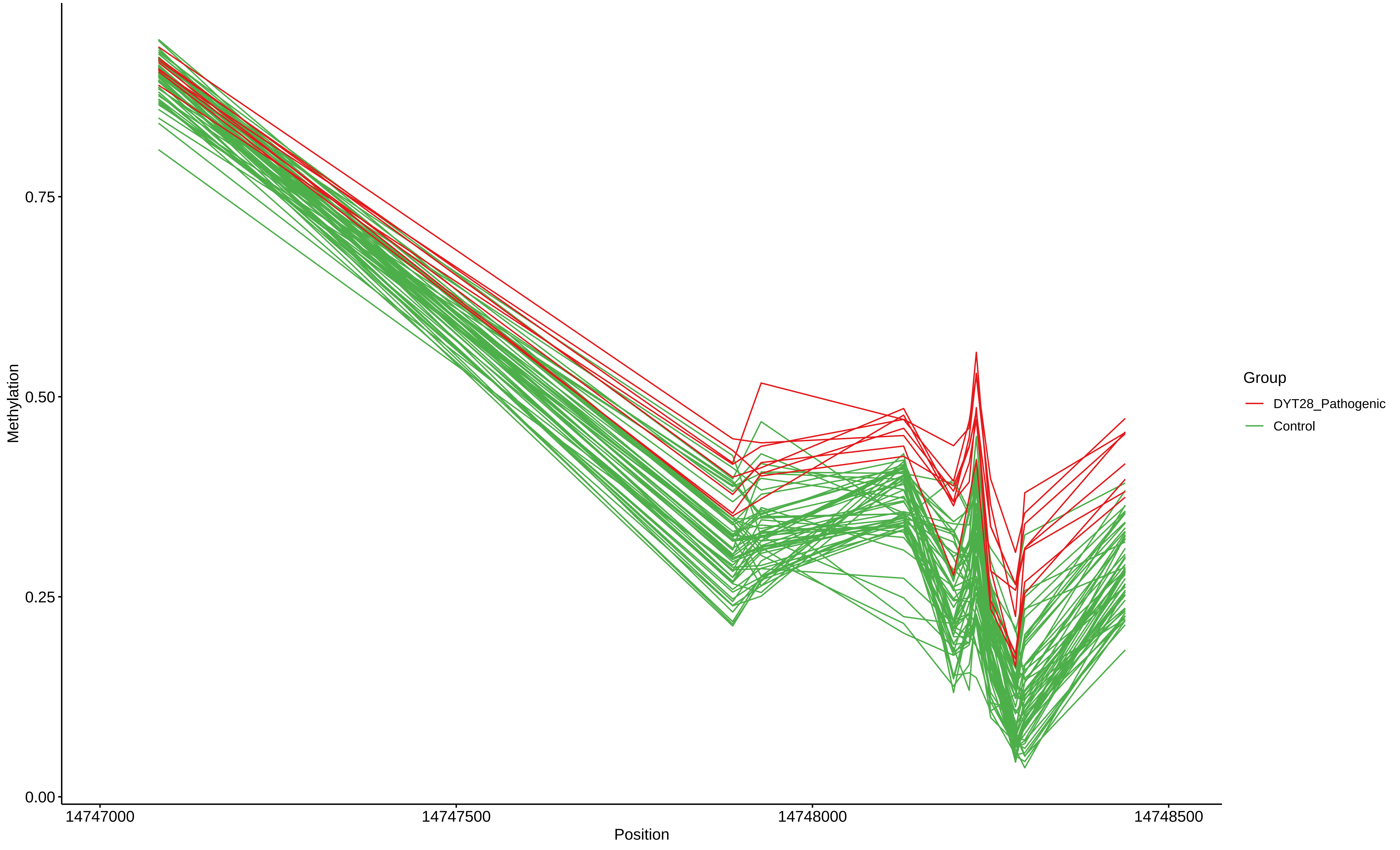

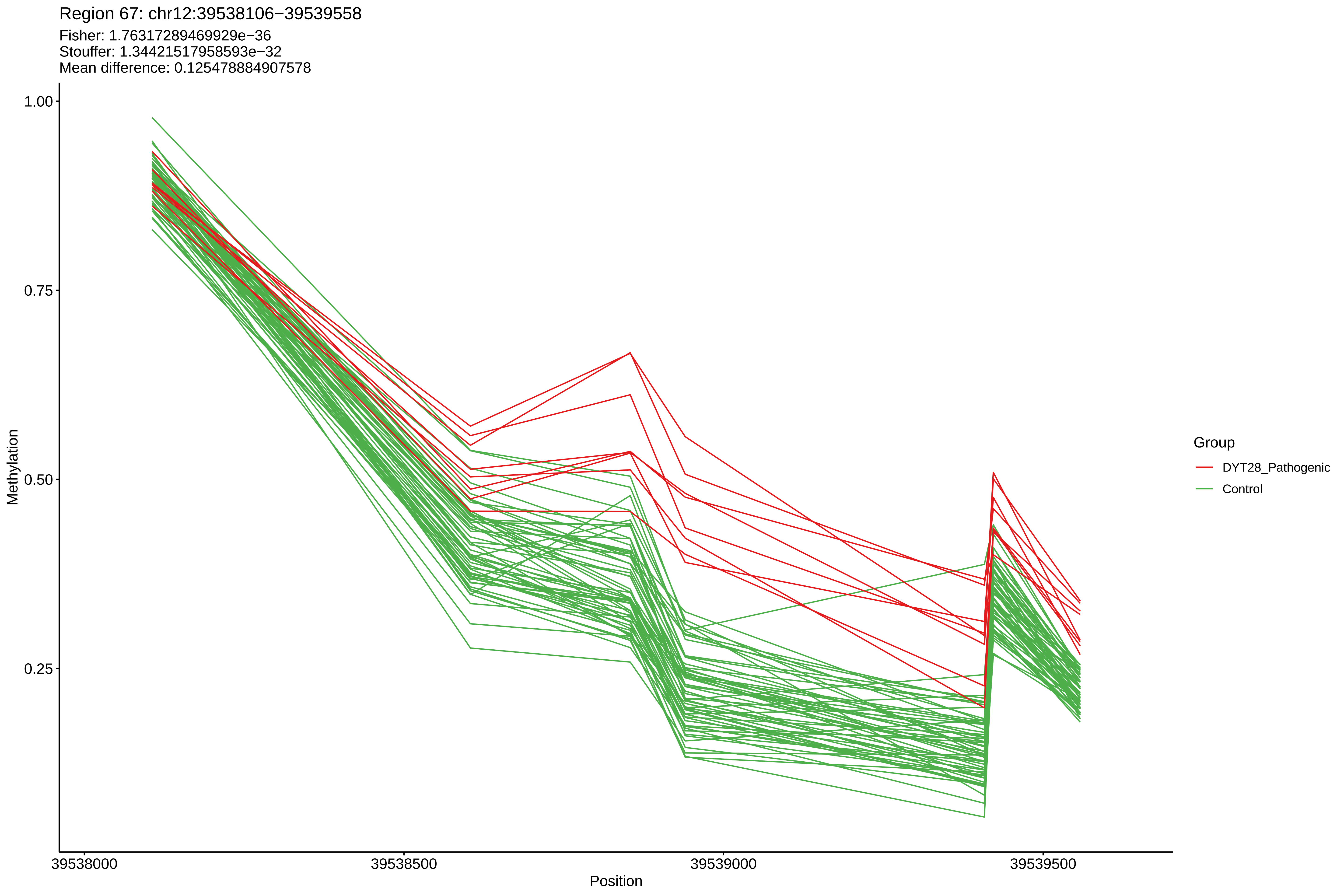

Region 67: chr12:39538106−39539558
Fisher: 1.76317289469929e−36
Stouffer: 1.34421517958593e−32
Mean difference: 0.125478884907578
Methylation
1.00
0.75
0.50
0.25
39538000
39538500
39539000
39539500
Position
Group
DYT28_Pathogenic
Control

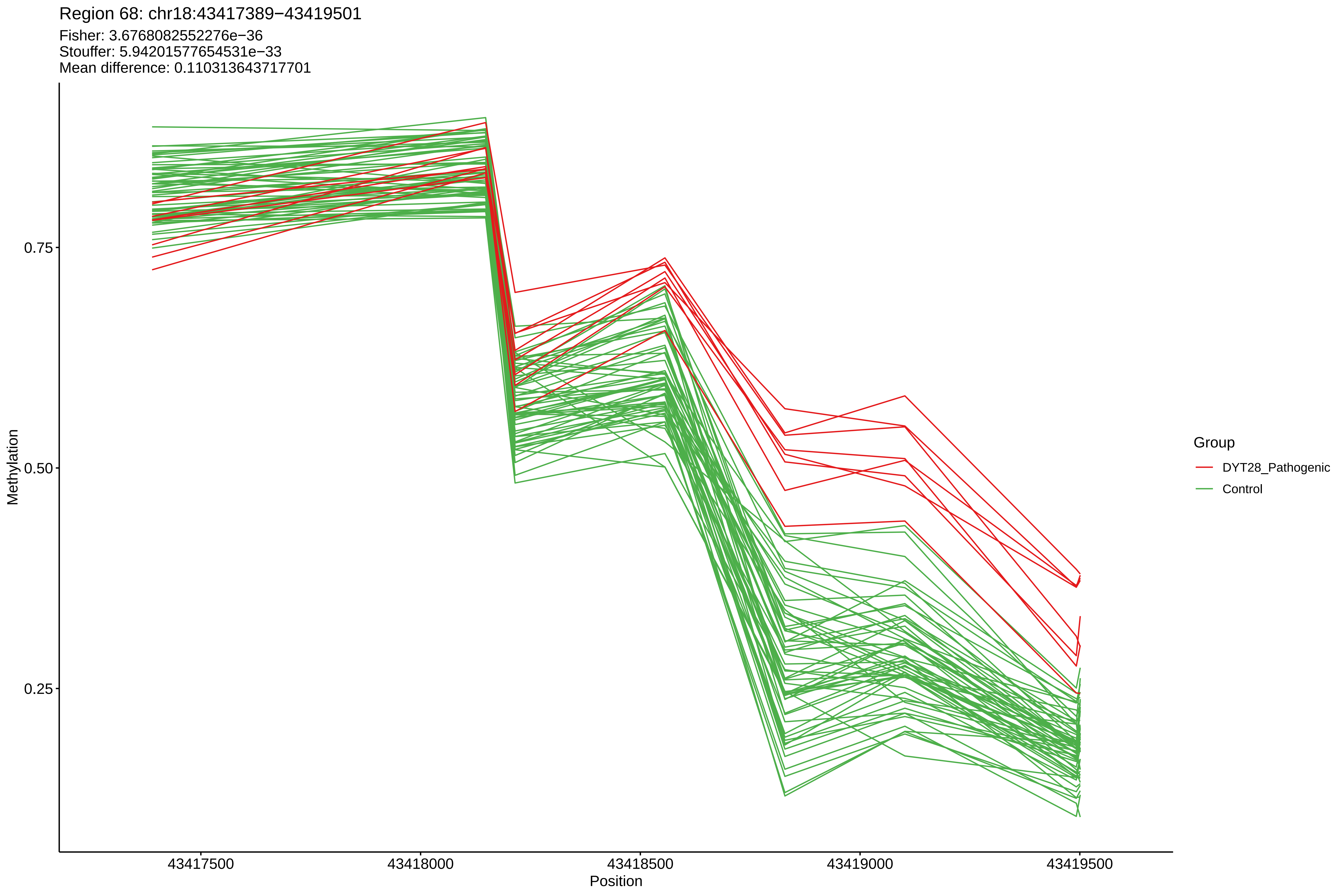

Region 68: chr18:43417389−43419501
Fisher: 3.6768082552276e−36
Stouffer: 5.94201577654531e−33
Mean difference: 0.110313643717701
Methylation
0.75
0.50
0.25
43417500
43418000
43418500
43419000
43419500
Position
Group
DYT28_Pathogenic
Control

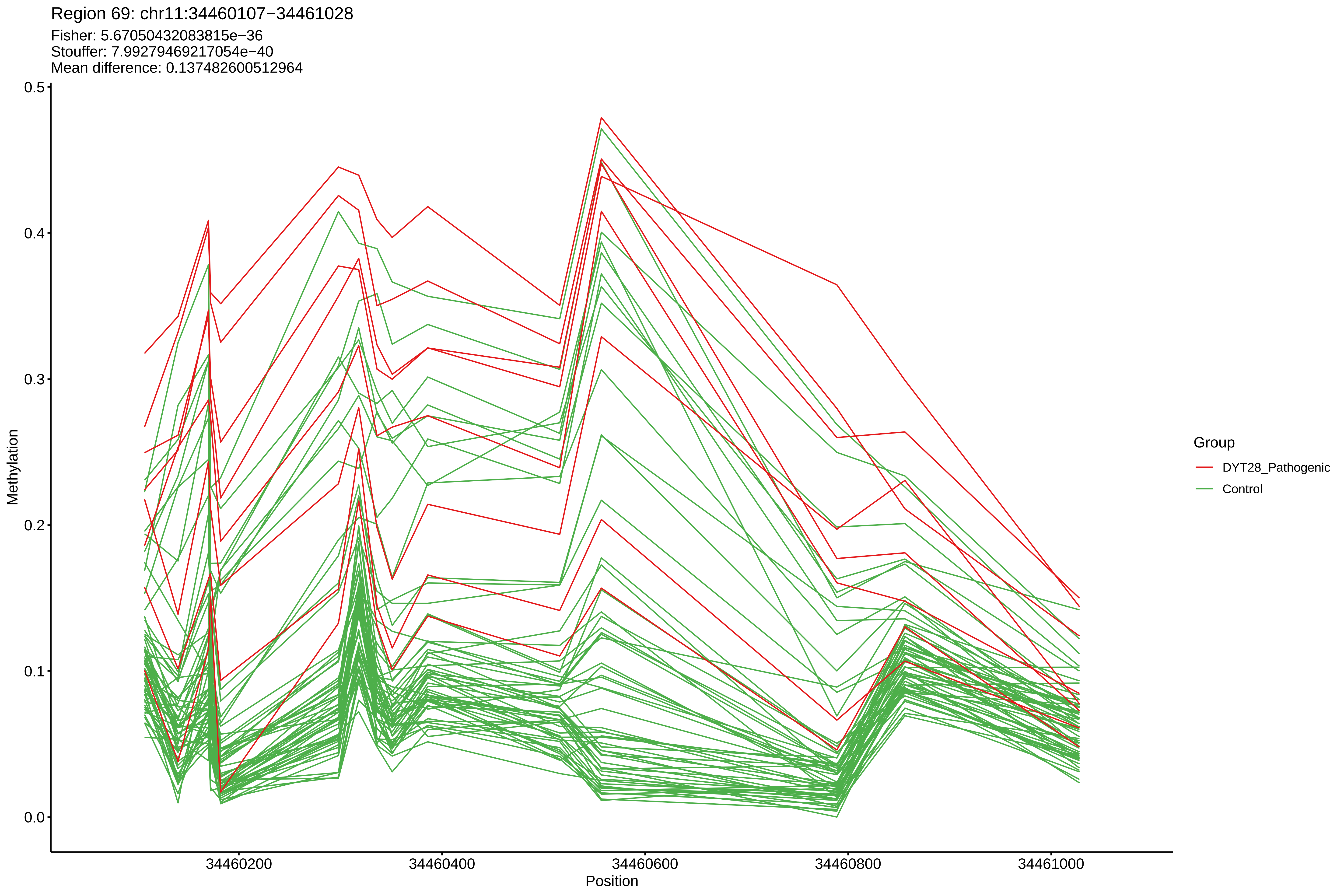
Region 69: chr11:34460107−34461028
Fisher: 5.67050432083815e−36
Stouffer: 7.99279469217054e−40
Mean difference: 0.137482600512964
Methylation
0.5
0.4
0.3
0.2
0.1
0.0
34460200
34460400
34460600
34460800
34461000
Position
Group
DYT28_Pathogenic
Control

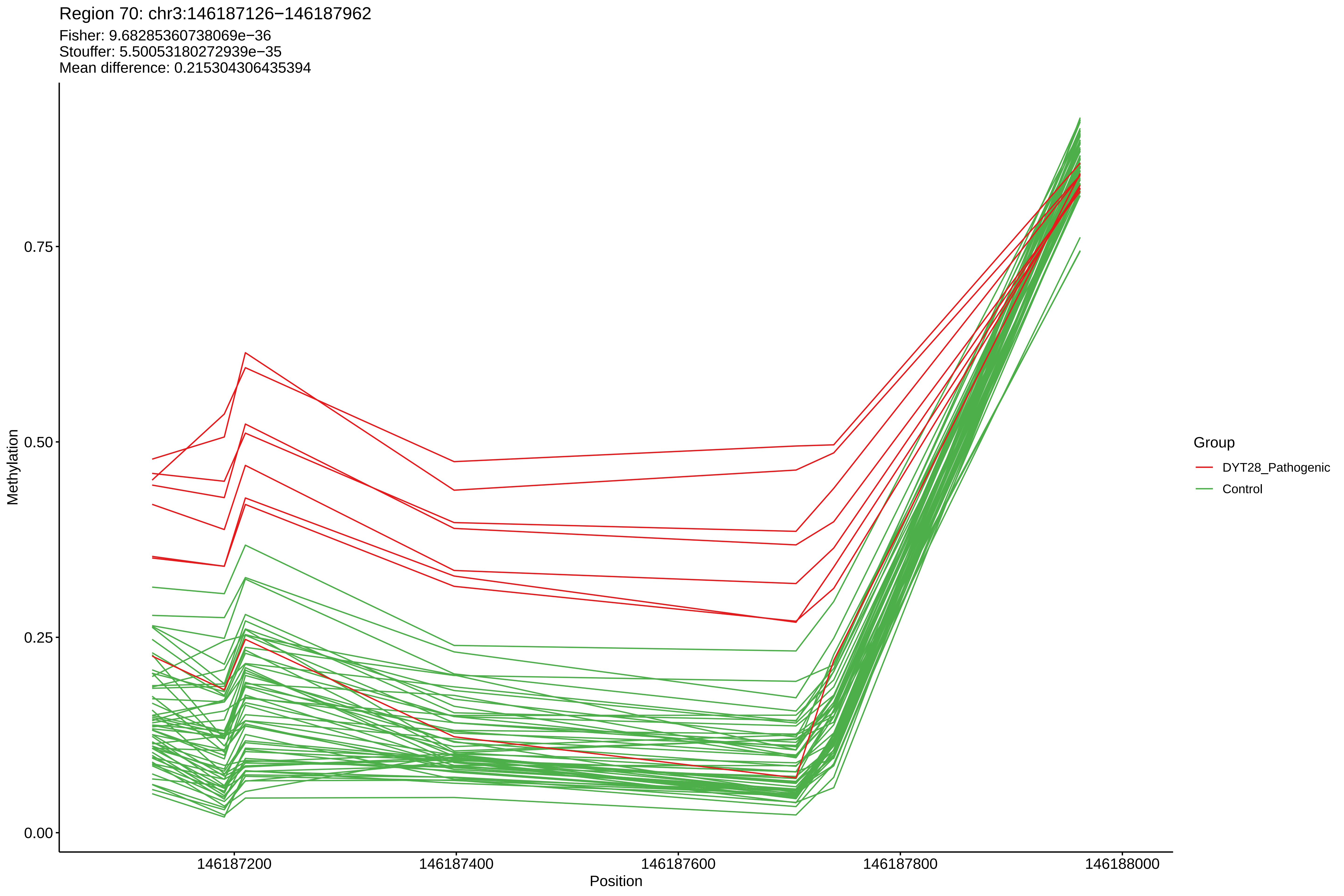

Region 70: chr3:146187126−146187962
Fisher: 9.68285360738069e−36
Stouffer: 5.50053180272939e−35
Mean difference: 0.215304306435394
Methylation
0.00
0.25
0.50
0.75
146187200
146187400
146187600
146187800
146188000
Position
Group
DYT28_Pathogenic
Control

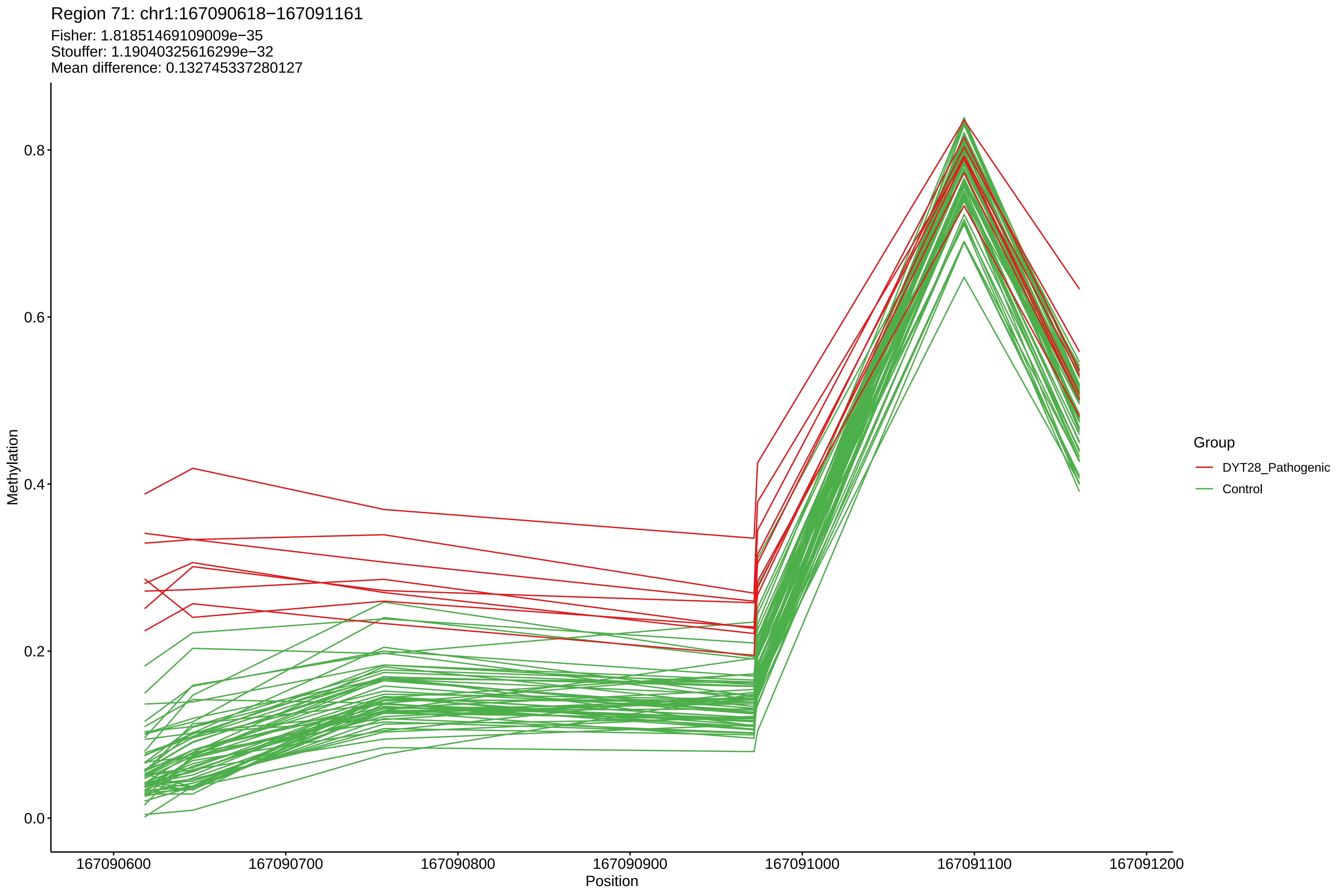

Region 71: chr1:167090618−167091161
Fisher: 1.81851469109009e−35
Stouffer: 1.19040325616299e−32
Mean difference: 0.132745337280127
Methylation
0.0
0.2
0.4
0.6
0.8
167090600
167090700
167090800
167090900
167091000
167091100
167091200
Position
Group
DYT28_Pathogenic
Control

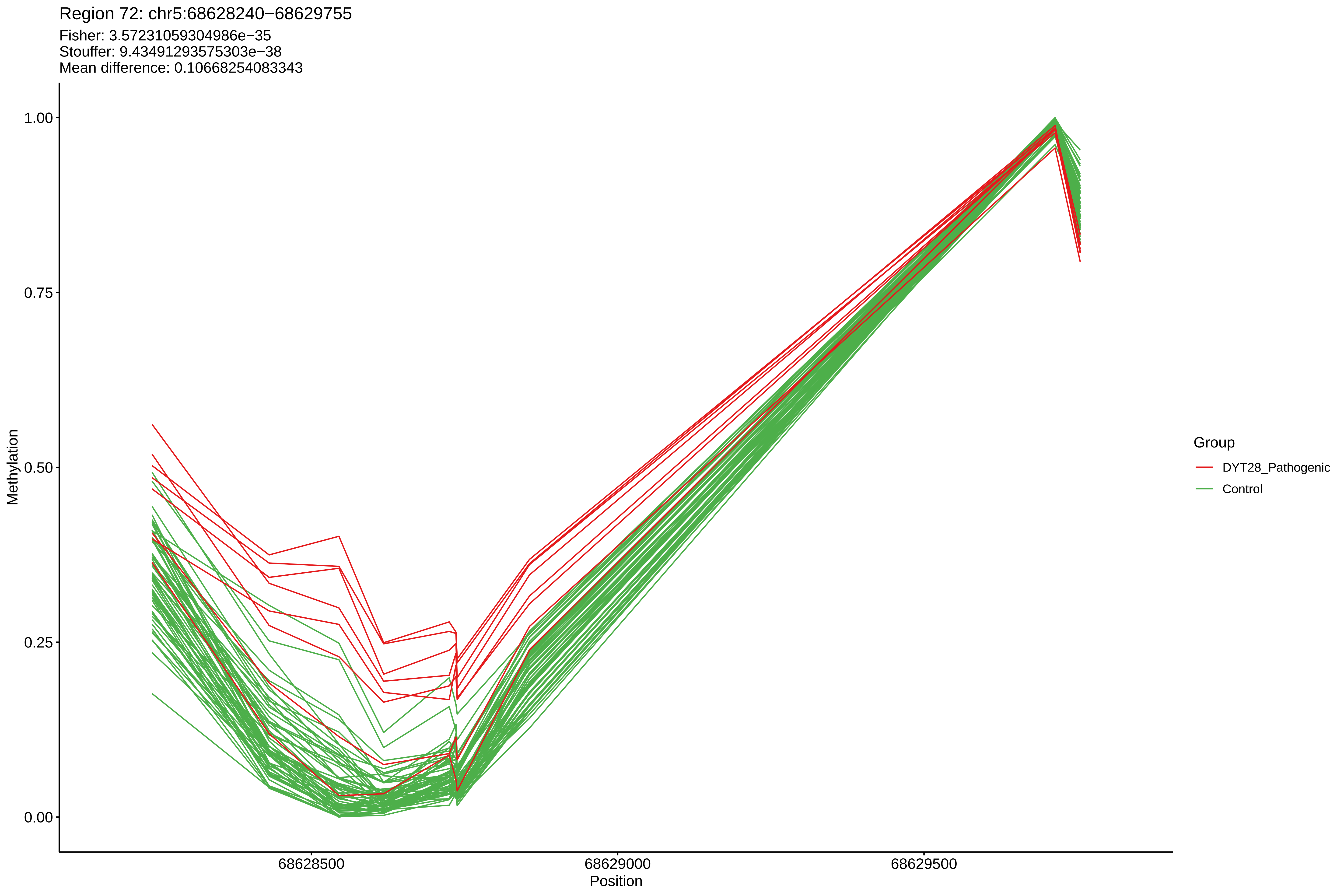

Region 72: chr5:68628240−68629755
Fisher: 3.57231059304986e−35
Stouffer: 9.43491293575303e−38
Mean difference: 0.10668254083343
Methylation
1.00
0.75
0.50
0.25
0.00
68628500
68629000
68629500
Position
Group
DYT28_Pathogenic
Control

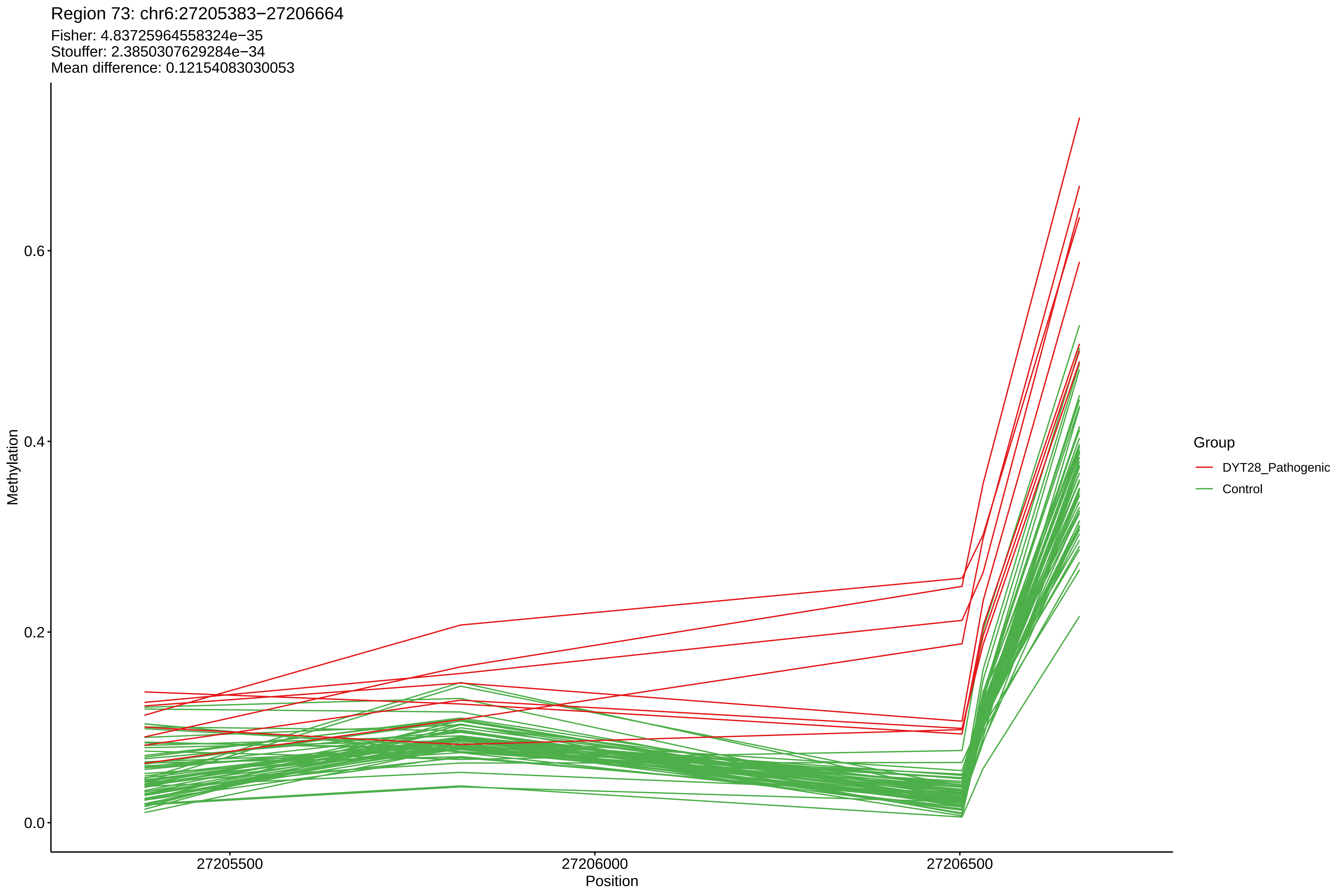
Region 73: chr6:27205383−27206664
Fisher: 4.83725964558324e−35
Stouffer: 2.3850307629284e−34
Mean difference: 0.12154083030053
Methylation
0.0
0.2
0.4
0.6
27205500
27206000
27206500
Position
Group
DYT28_Pathogenic
Control

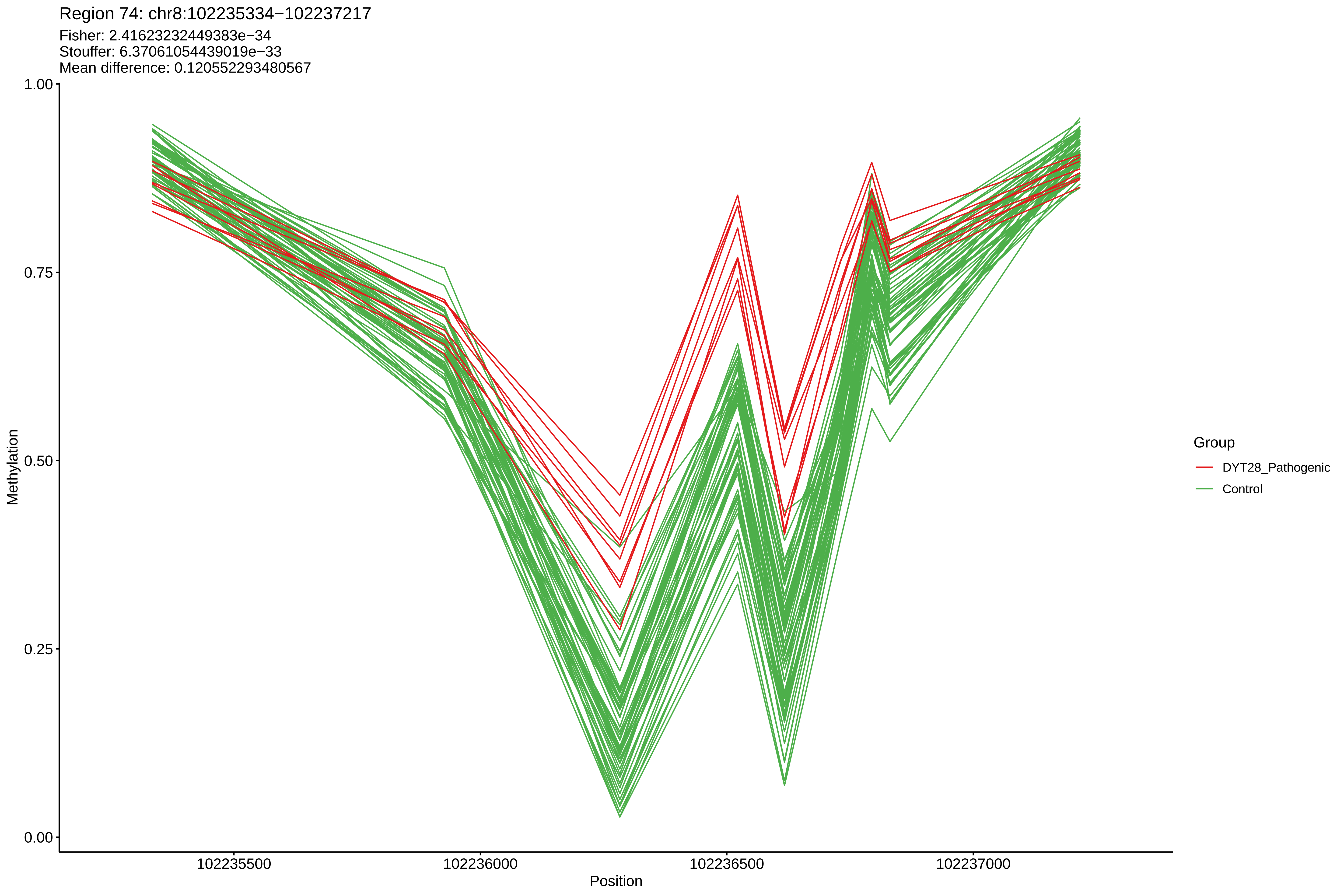
Region 74: chr8:102235334−102237217
Fisher: 2.41623232449383e−34
Stouffer: 6.37061054439019e−33
Mean difference: 0.120552293480567
1.00
0.75
0.50
0.25
0.00
Methylation
102235500
102236000
102236500
102237000
Position
Group
DYT28_Pathogenic
Control

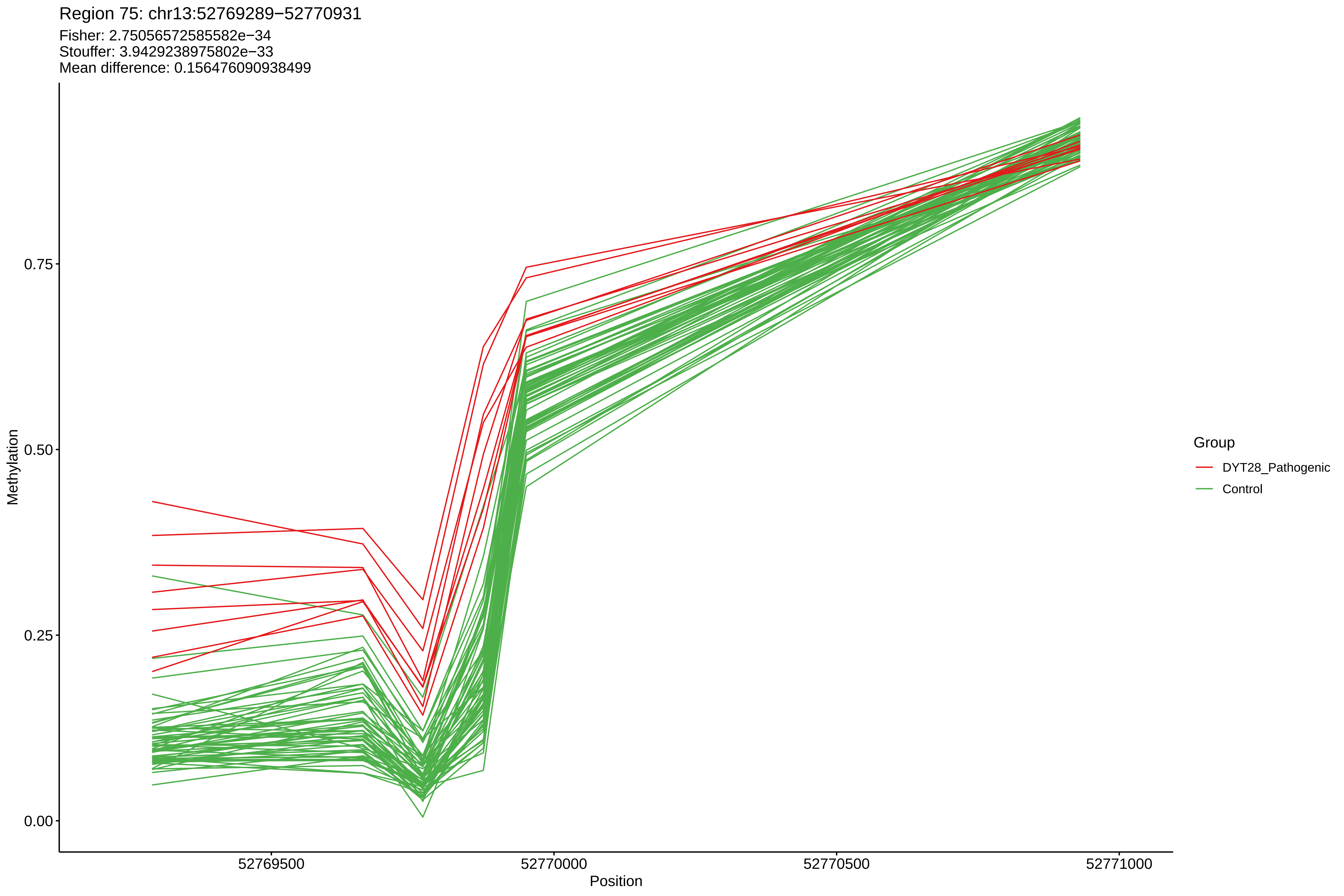

Region 75: chr13:52769289−52770931
Fisher: 2.75056572585582e−34
Stouffer: 3.9429238975802e−33
Mean difference: 0.156476090938499
Methylation
0.00
0.25
0.50
0.75
Position
52769500
52770000
52770500
52771000
Group
DYT28_Pathogenic
Control

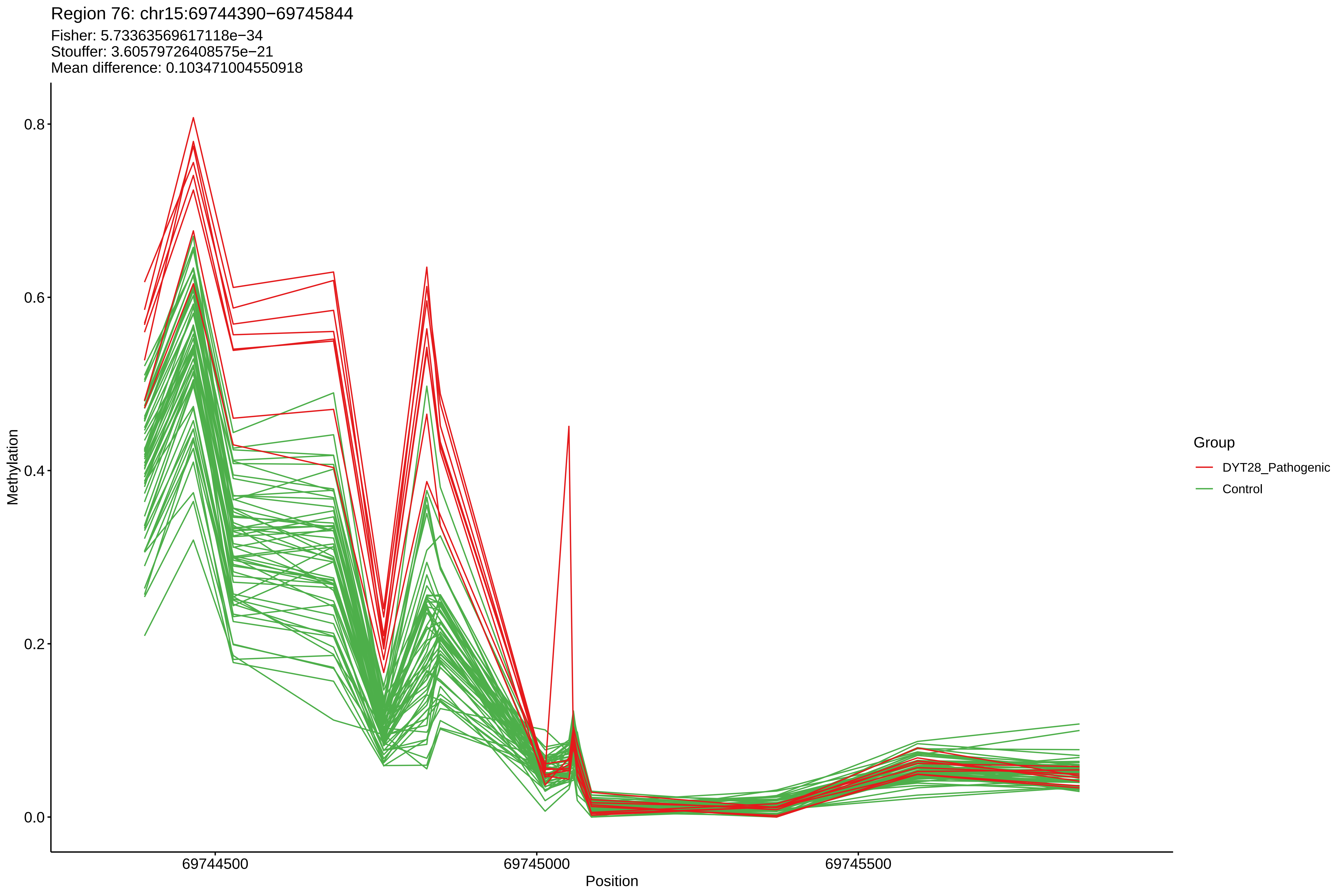

Region 76: chr15:69744390−69745844
Fisher: 5.73363569617118e−34
Stouffer: 3.60579726408575e−21
Mean difference: 0.103471004550918
Methylation
0.0
0.2
0.4
0.6
0.8
69744500
69745000
69745500
Position
Group
DYT28_Pathogenic
Control

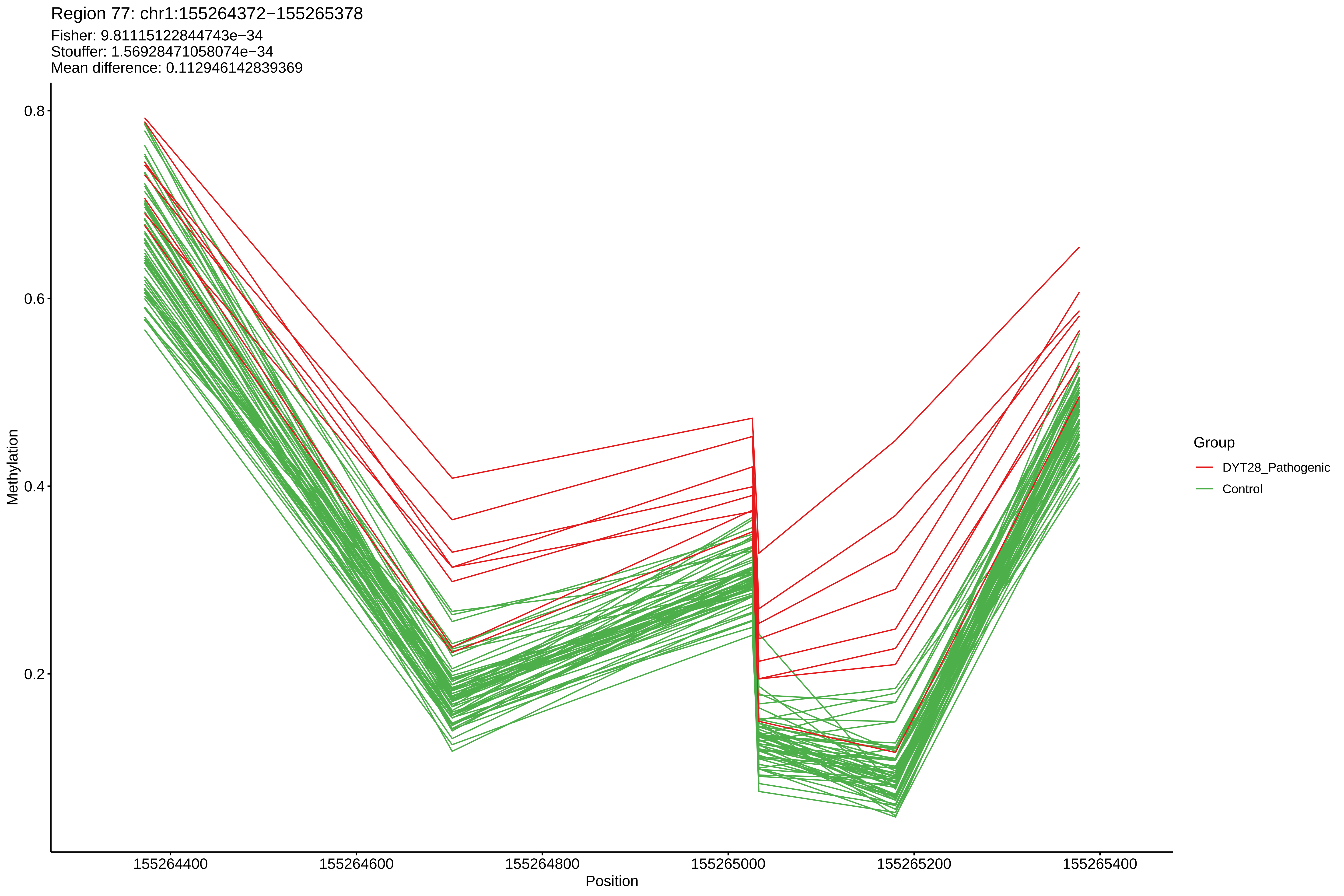
Region 77: chr1:155264372−155265378
Fisher: 9.81115122844743e−34
Stouffer: 1.56928471058074e−34
Mean difference: 0.112946142839369
0.8
0.6
0.4
0.2
Methylation
155264400
155264600
155264800
155265000
155265200
155265400
Position
Group
DYT28_Pathogenic
Control

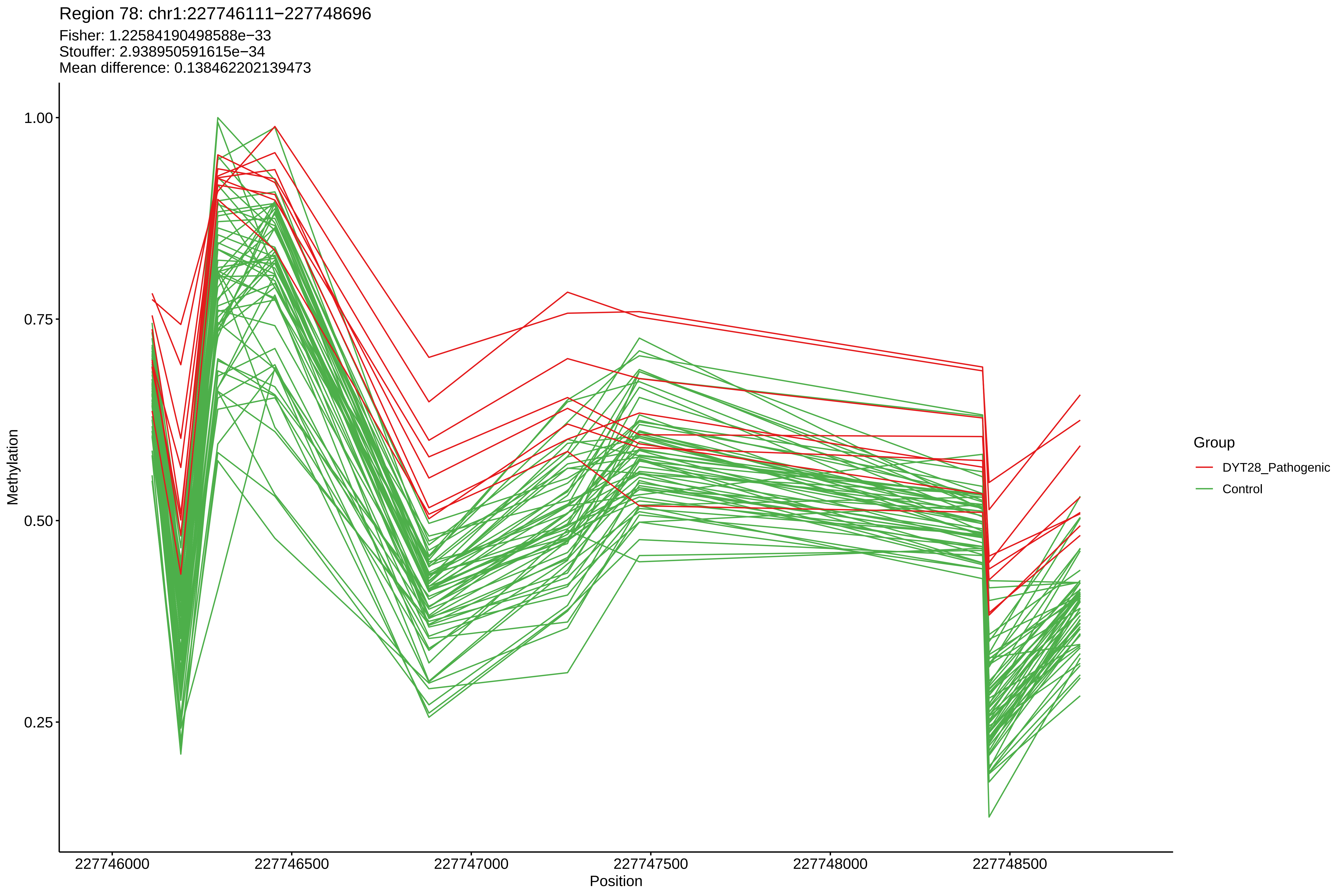
Region 78: chr1:227746111−227748696
Fisher: 1.22584190498588e−33
Stouffer: 2.938950591615e−34
Mean difference: 0.138462202139473
Methylation
1.00
0.75
0.50
0.25
227746000
227746500
227747000
227747500
227748000
227748500
Position
Group
DYT28_Pathogenic
Control

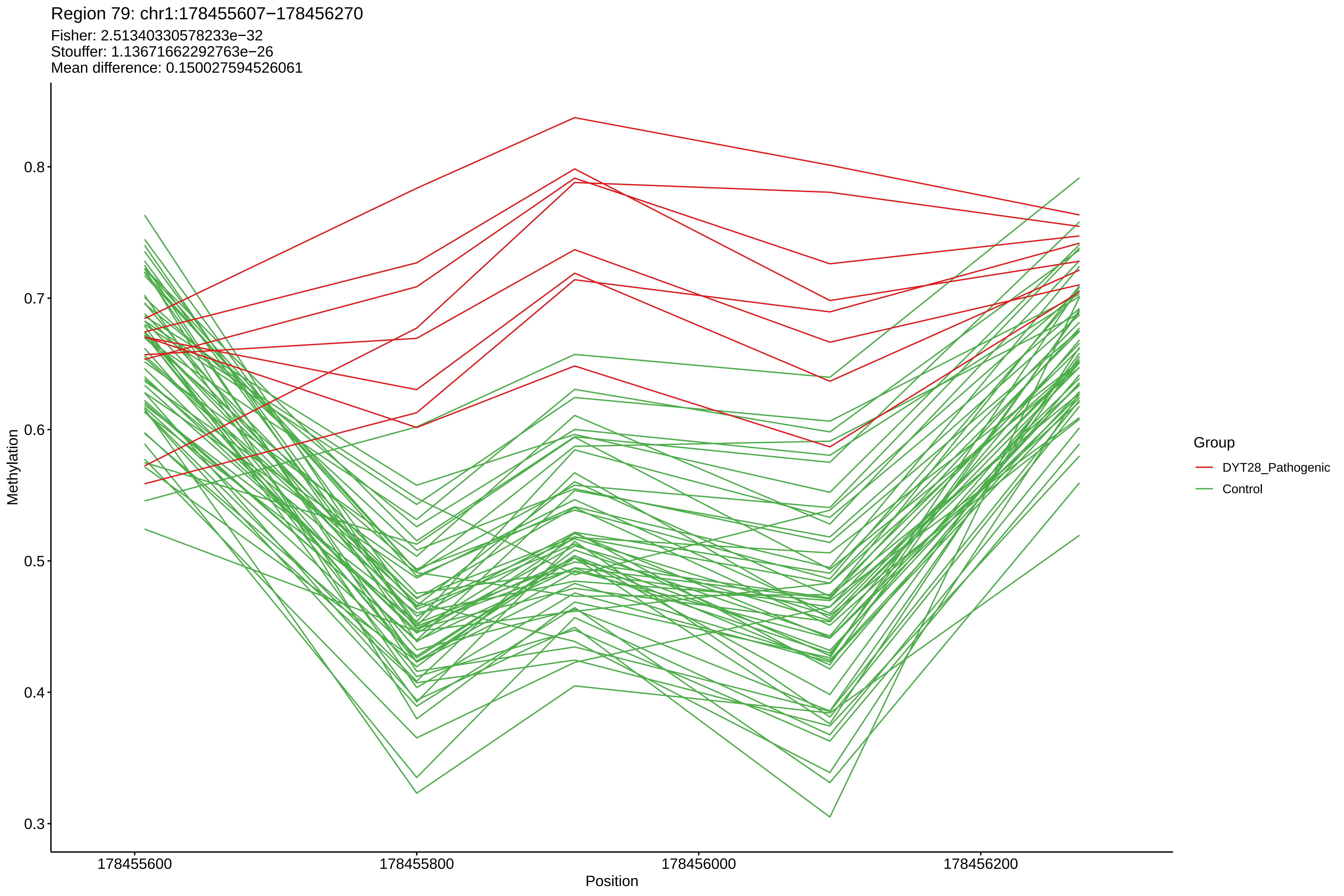
Region 79: chr1:178455607−178456270
Fisher: 2.51340330578233e−32
Stouffer: 1.13671662292763e−26
Mean difference: 0.150027594526061
Methylation
0.8
0.7
0.6
0.5
0.4
0.3
178455600
178455800
178456000
178456200
Position
Group
DYT28_Pathogenic
Control

Region 80: chr5:126408756−126410348
Fisher: 3.65713980583759e−32
Stouffer: 1.48244664693489e−28
Mean difference: 0.110493277992197

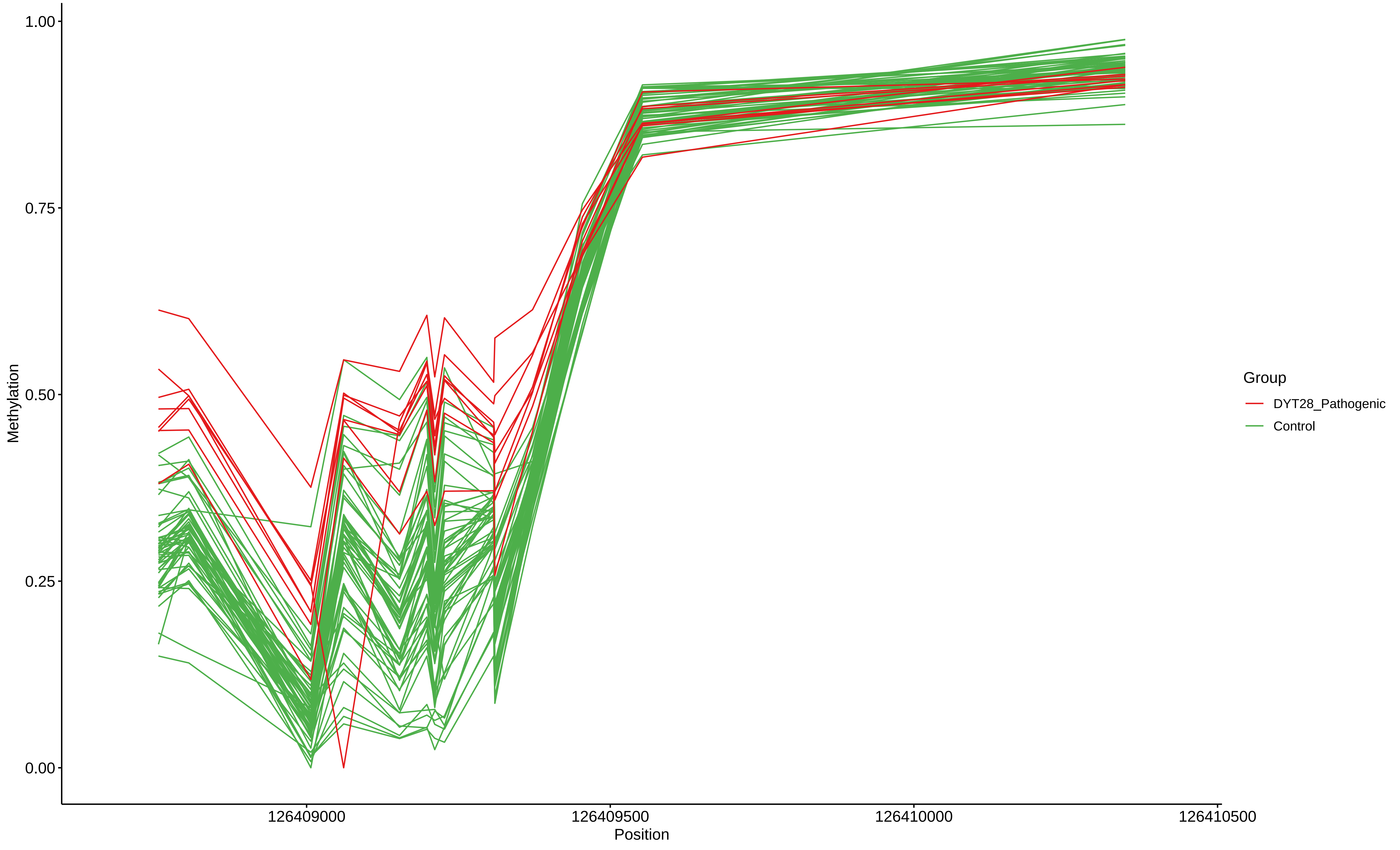

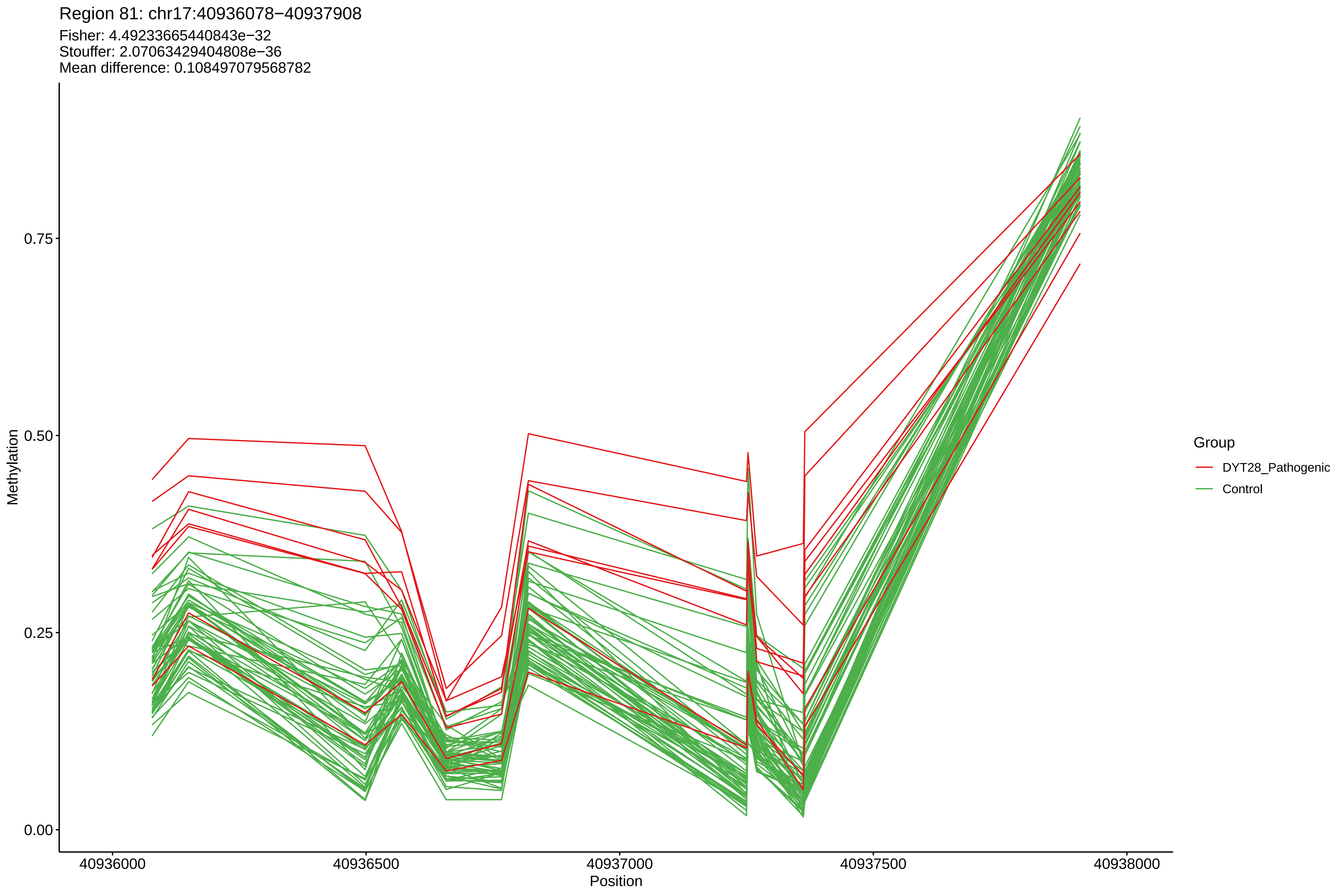
Region 81: chr17:40936078−40937908
Fisher: 4.49233665440843e−32
Stouffer: 2.07063429404808e−36
Mean difference: 0.108497079568782
Methylation
0.00
0.25
0.50
0.75
40936000
40936500
40937000
40937500
40938000
Position
Group
DYT28_Pathogenic
Control

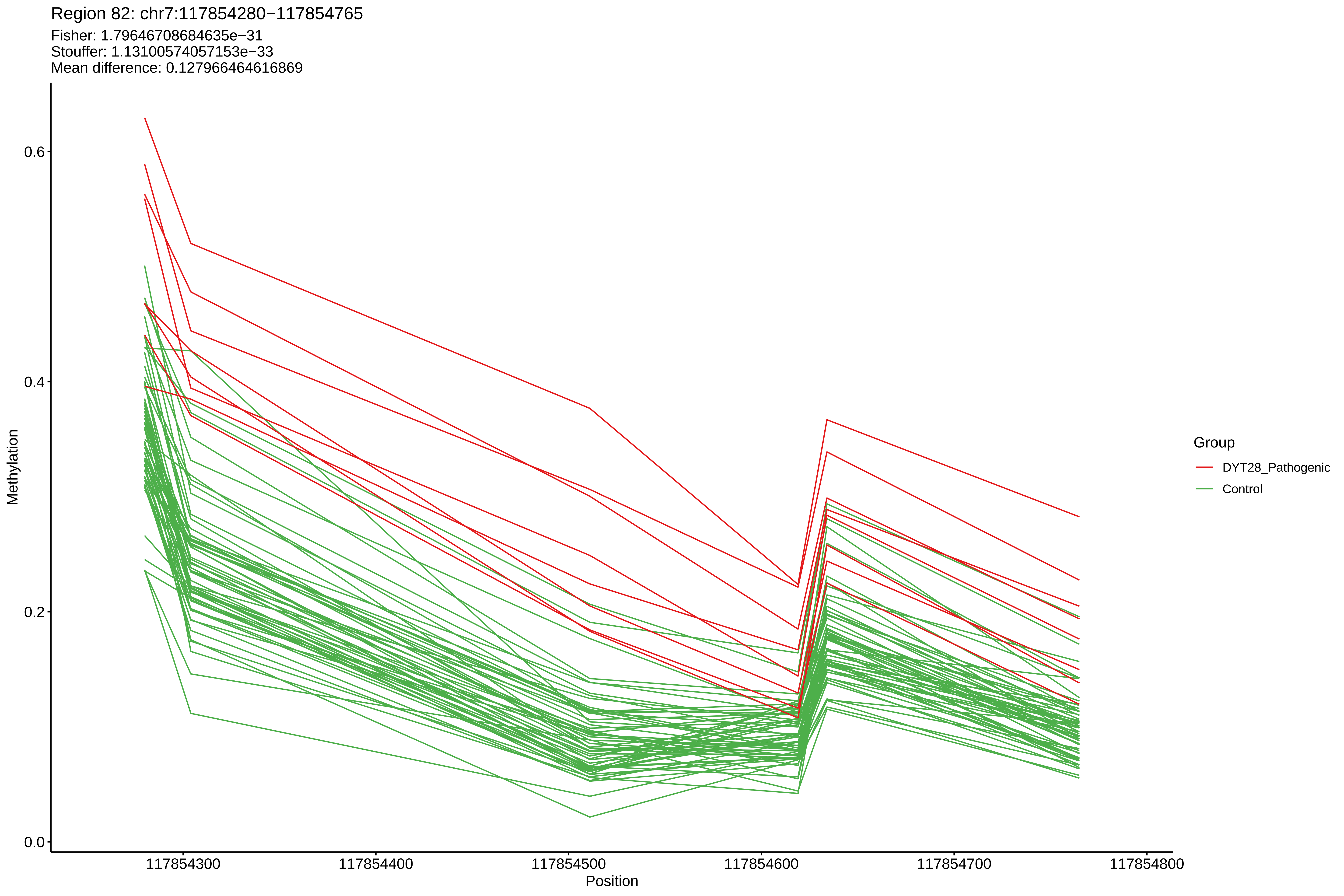
Region 82: chr7:117854280−117854765
Fisher: 1.79646708684635e−31
Stouffer: 1.13100574057153e−33
Mean difference: 0.127966464616869
Methylation
0.6
0.4
0.2
0.0
117854300
117854400
117854500
117854600
117854700
117854800
Position
Group
DYT28_Pathogenic
Control

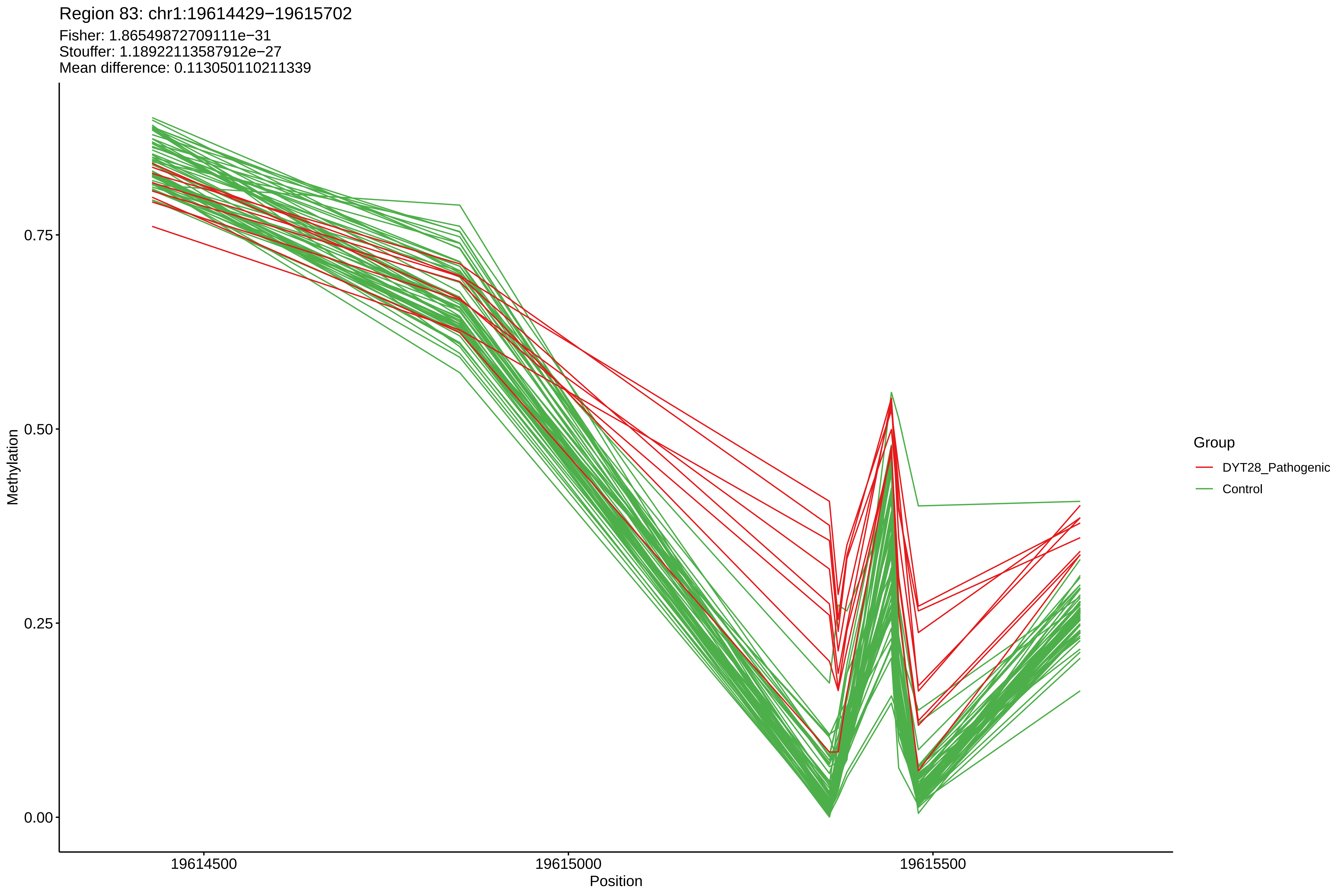

Region 83: chr1:19614429−19615702
Fisher: 1.86549872709111e−31
Stouffer: 1.18922113587912e−27
Mean difference: 0.113050110211339
Methylation
0.75
0.50
0.25
0.00
19614500
19615000
19615500
Position
Group
DYT28_Pathogenic
Control

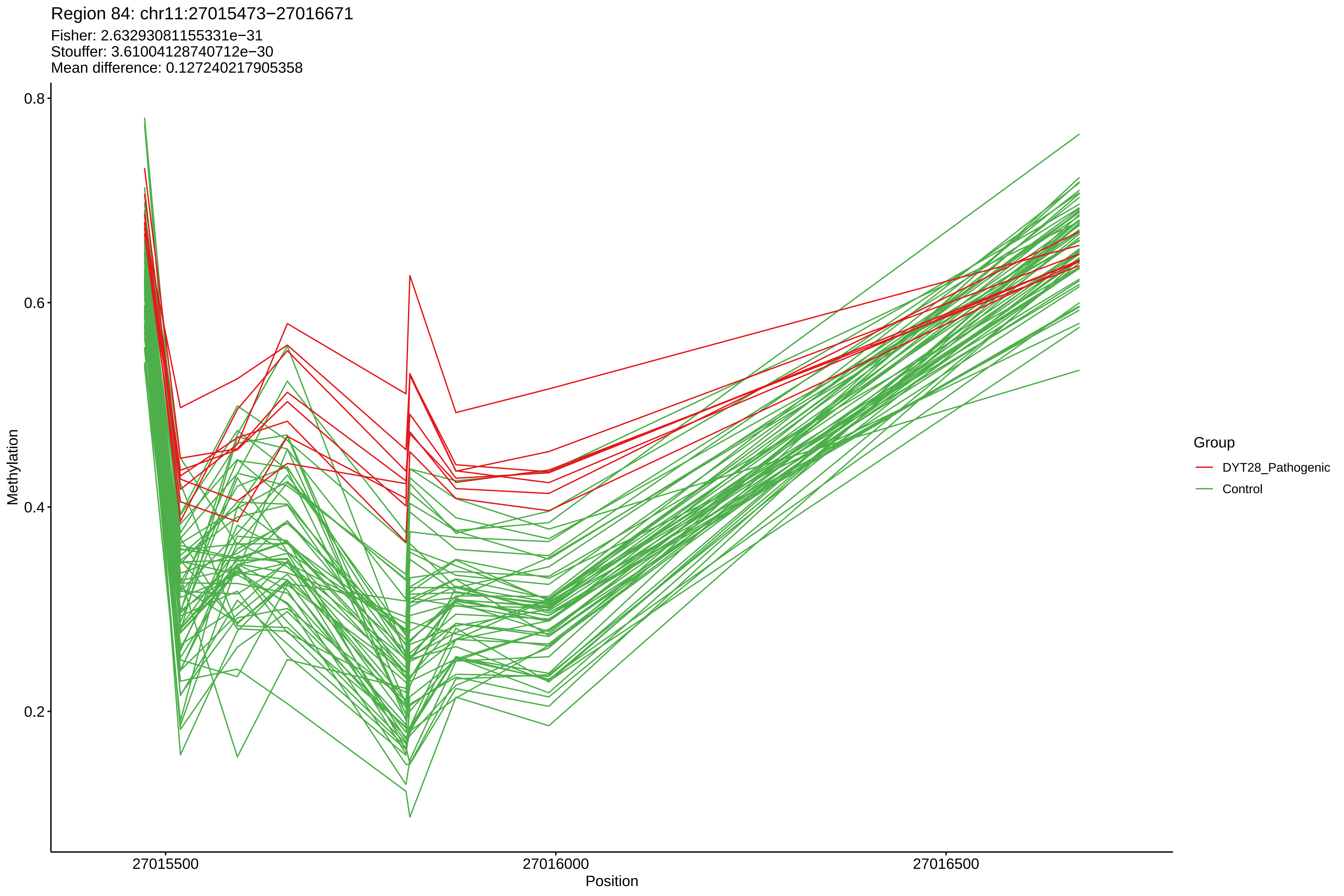

Region 84: chr11:27015473−27016671
Fisher: 2.63293081155331e−31
Stouffer: 3.61004128740712e−30
Mean difference: 0.127240217905358
Methylation
0.8
0.6
0.4
0.2
27015500
27016000
27016500
Position
Group
DYT28_Pathogenic
Control

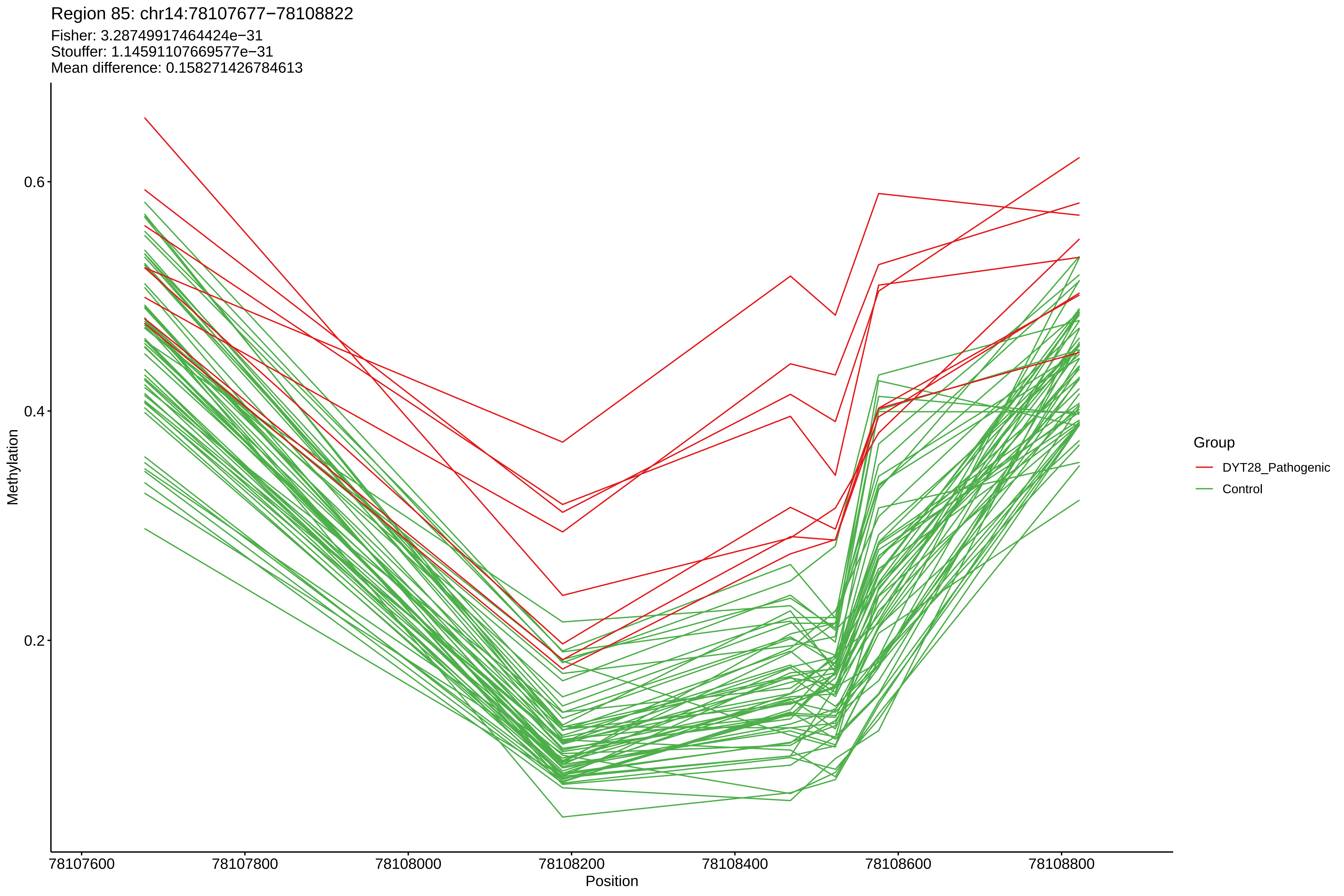
Region 85: chr14:78107677−78108822
Fisher: 3.28749917464424e−31
Stouffer: 1.14591107669577e−31
Mean difference: 0.158271426784613
Methylation
0.6
0.4
0.2
78107600
78107800
78108000
78108200
78108400
78108600
78108800
Position
Group
DYT28_Pathogenic
Control

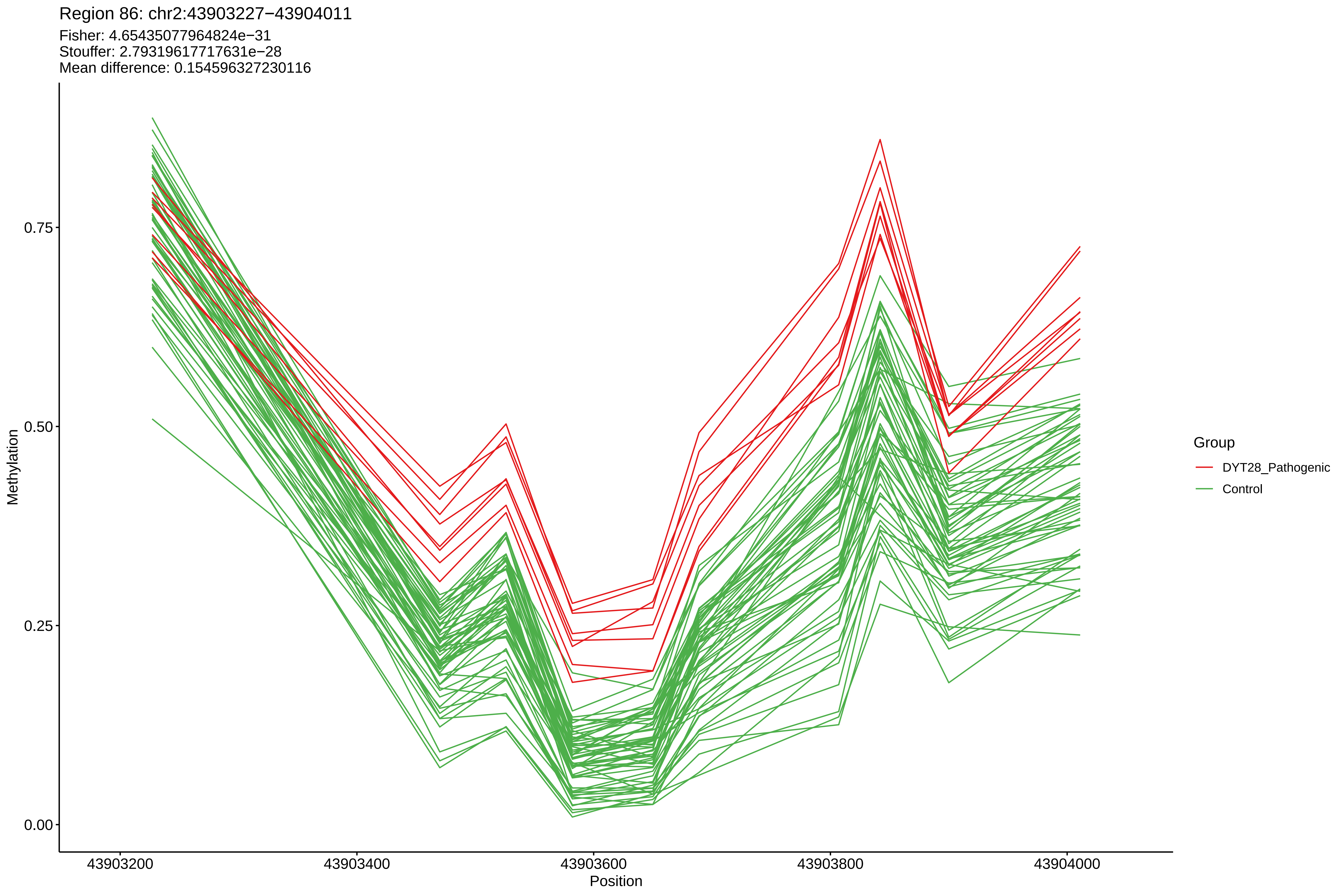
Region 86: chr2:43903227−43904011
Fisher: 4.65435077964824e−31
Stouffer: 2.79319617717631e−28
Mean difference: 0.154596327230116
Methylation
0.00
0.25
0.50
0.75
43903200
43903400
43903600
43903800
43904000
Position
Group
DYT28_Pathogenic
Control

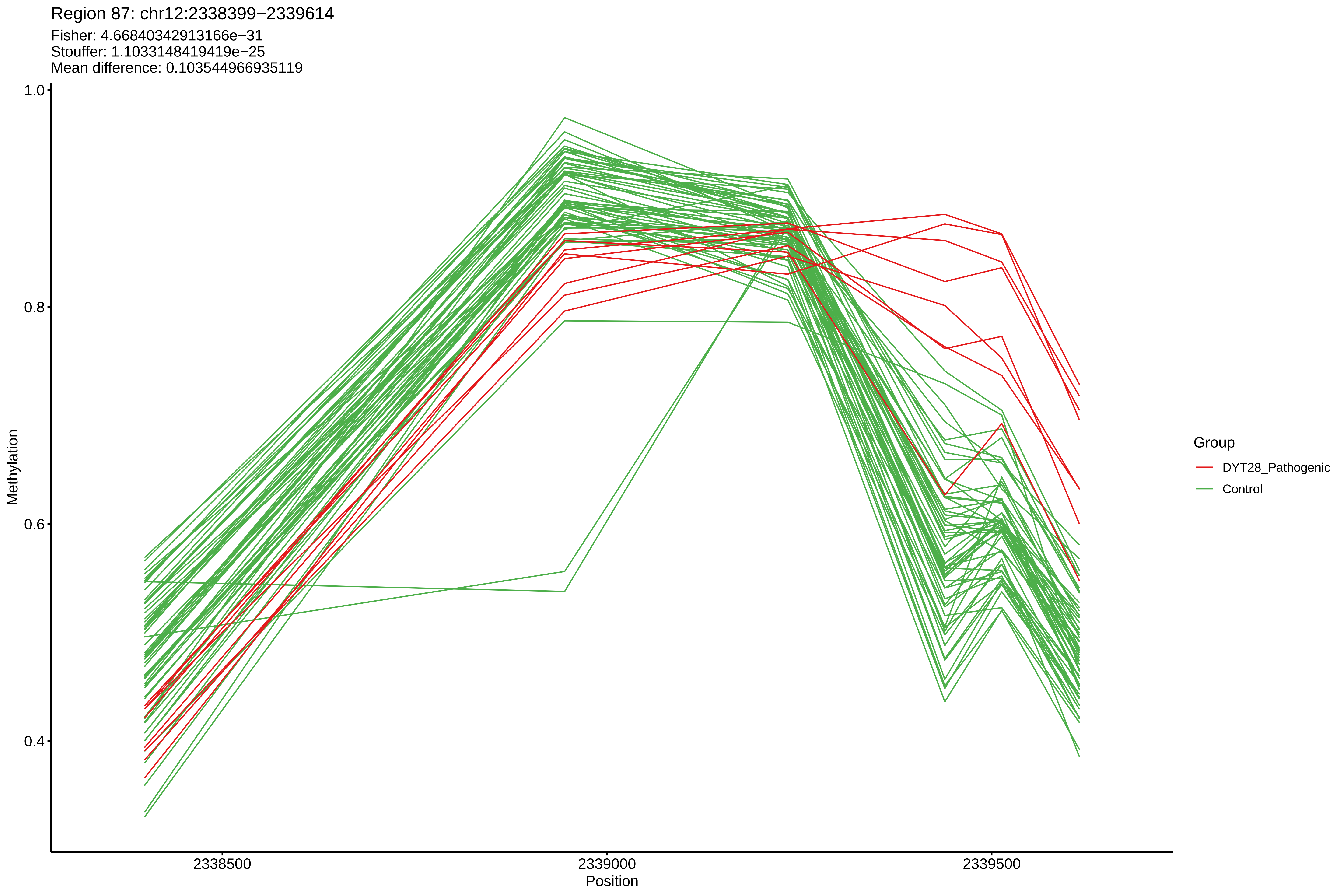

Region 87: chr12:2338399−2339614
Fisher: 4.66840342913166e−31
Stouffer: 1.1033148419419e−25
Mean difference: 0.103544966935119
Methylation
1.0
0.8
0.6
0.4
2338500
2339000
2339500
Position
Group
DYT28_Pathogenic
Control

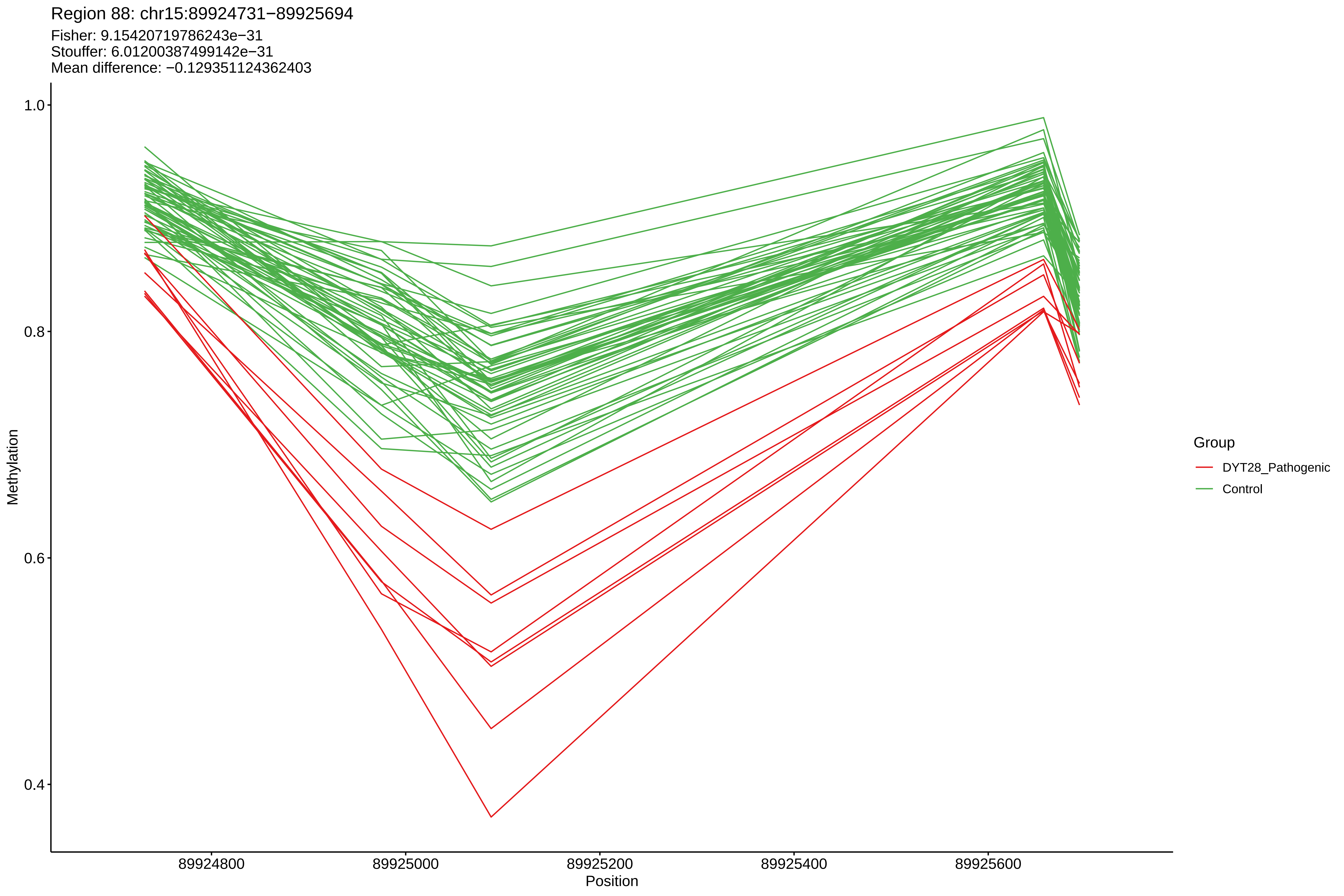

Region 88: chr15:89924731−89925694
Fisher: 9.15420719786243e−31
Stouffer: 6.01200387499142e−31
Mean difference: −0.129351124362403
Methylation
1.0
0.8
0.6
0.4
89924800
89925000
89925200
89925400
89925600
Position
Group
DYT28_Pathogenic
Control

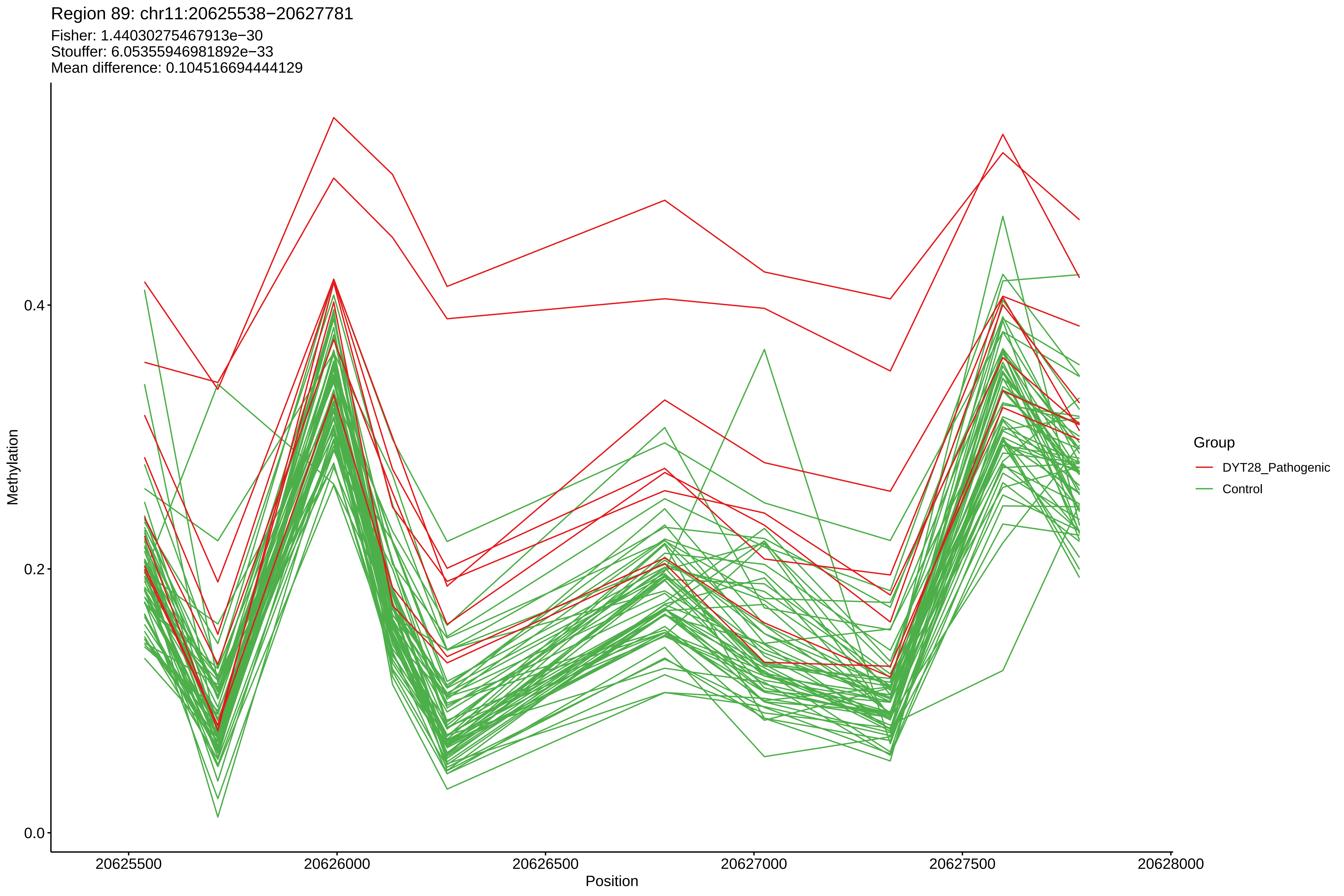

Region 89: chr11:20625538−20627781
Fisher: 1.44030275467913e−30
Stouffer: 6.05355946981892e−33
Mean difference: 0.104516694444129
Methylation
0.0
0.2
0.4
Position
20625500
20626000
20626500
20627000
20627500
20628000
Group
DYT28_Pathogenic
Control

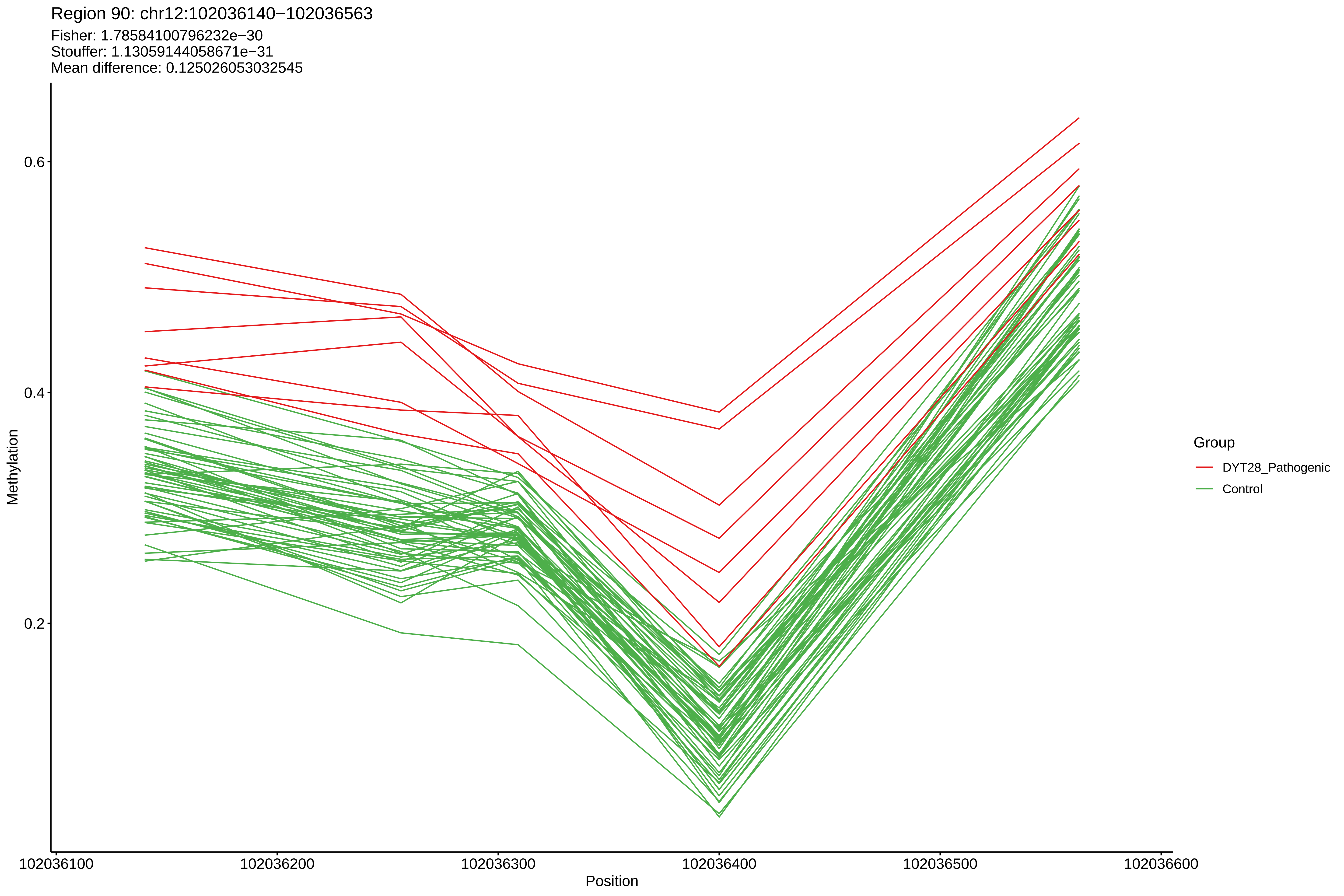

Region 90: chr12:102036140−102036563
Fisher: 1.78584100796232e−30
Stouffer: 1.13059144058671e−31
Mean difference: 0.125026053032545
Methylation
0.6
0.4
0.2
102036100
102036200
102036300
102036400
102036500
102036600
Position
Group
DYT28_Pathogenic
Control

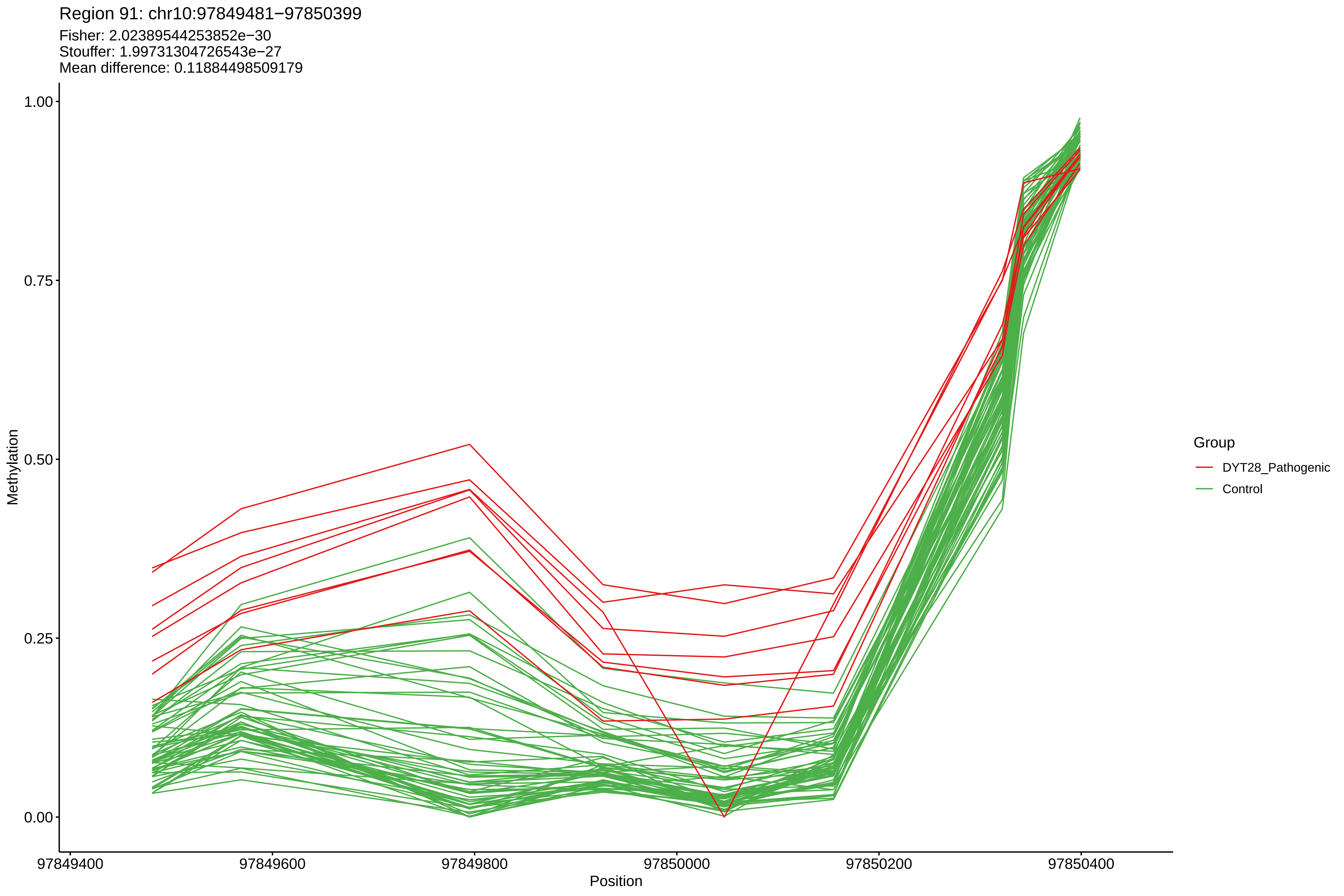
Region 91: chr10:97849481−97850399
Fisher: 2.02389544253852e−30
Stouffer: 1.99731304726543e−27
Mean difference: 0.11884498509179
Methylation
1.00
0.75
0.50
0.25
0.00
97849400
97849600
97849800
97850000
97850200
97850400
Position
Group
DYT28_Pathogenic
Control

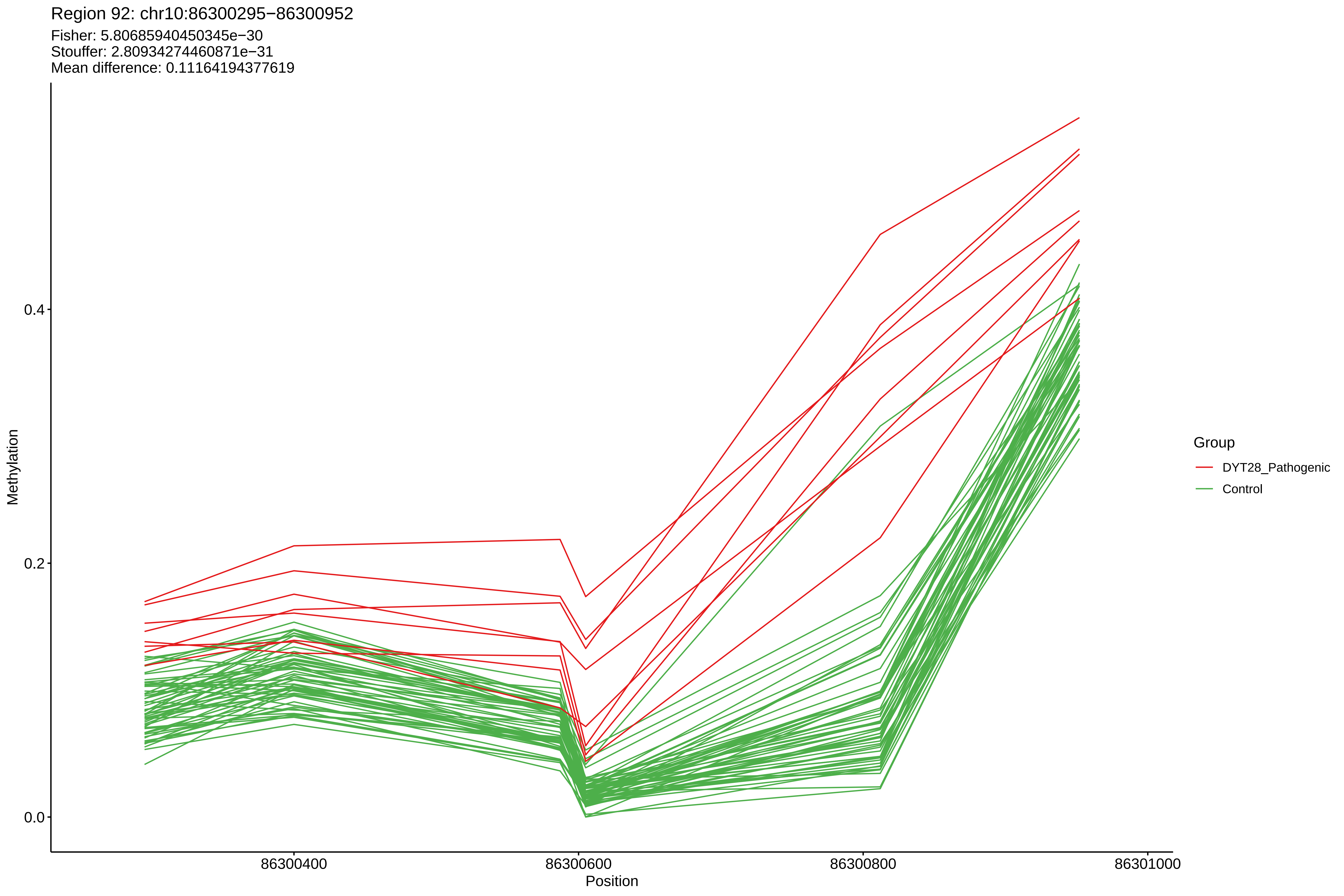
Region 92: chr10:86300295−86300952
Fisher: 5.80685940450345e−30
Stouffer: 2.80934274460871e−31
Mean difference: 0.11164194377619
Methylation
0.4
0.2
0.0
86300400
86300600
86300800
86301000
Position
Group
DYT28_Pathogenic
Control

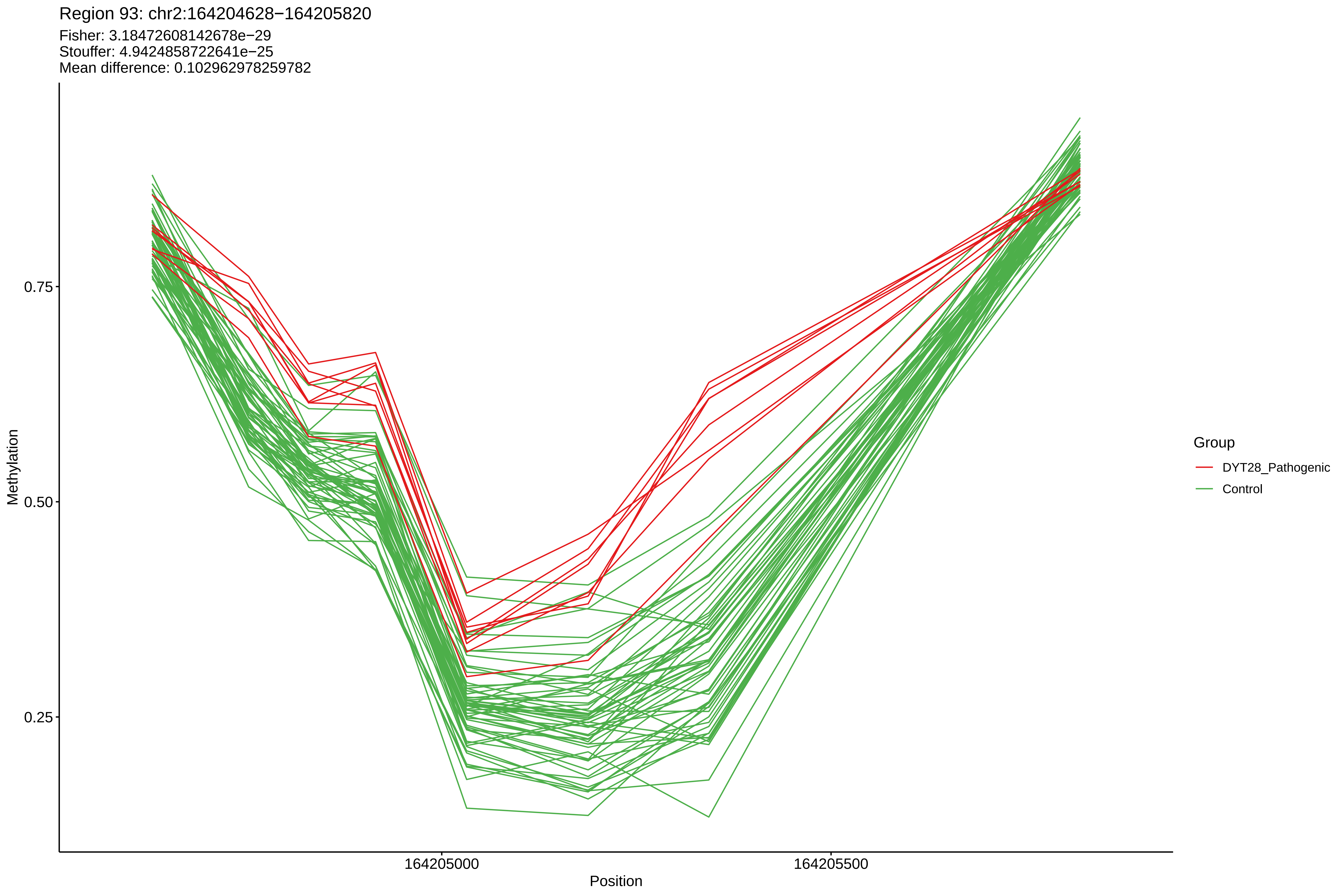
Region 93: chr2:164204628−164205820
Fisher: 3.18472608142678e−29
Stouffer: 4.9424858722641e−25
Mean difference: 0.102962978259782
Methylation
0.75
0.50
0.25
164205000
164205500
Position
Group
DYT28_Pathogenic
Control

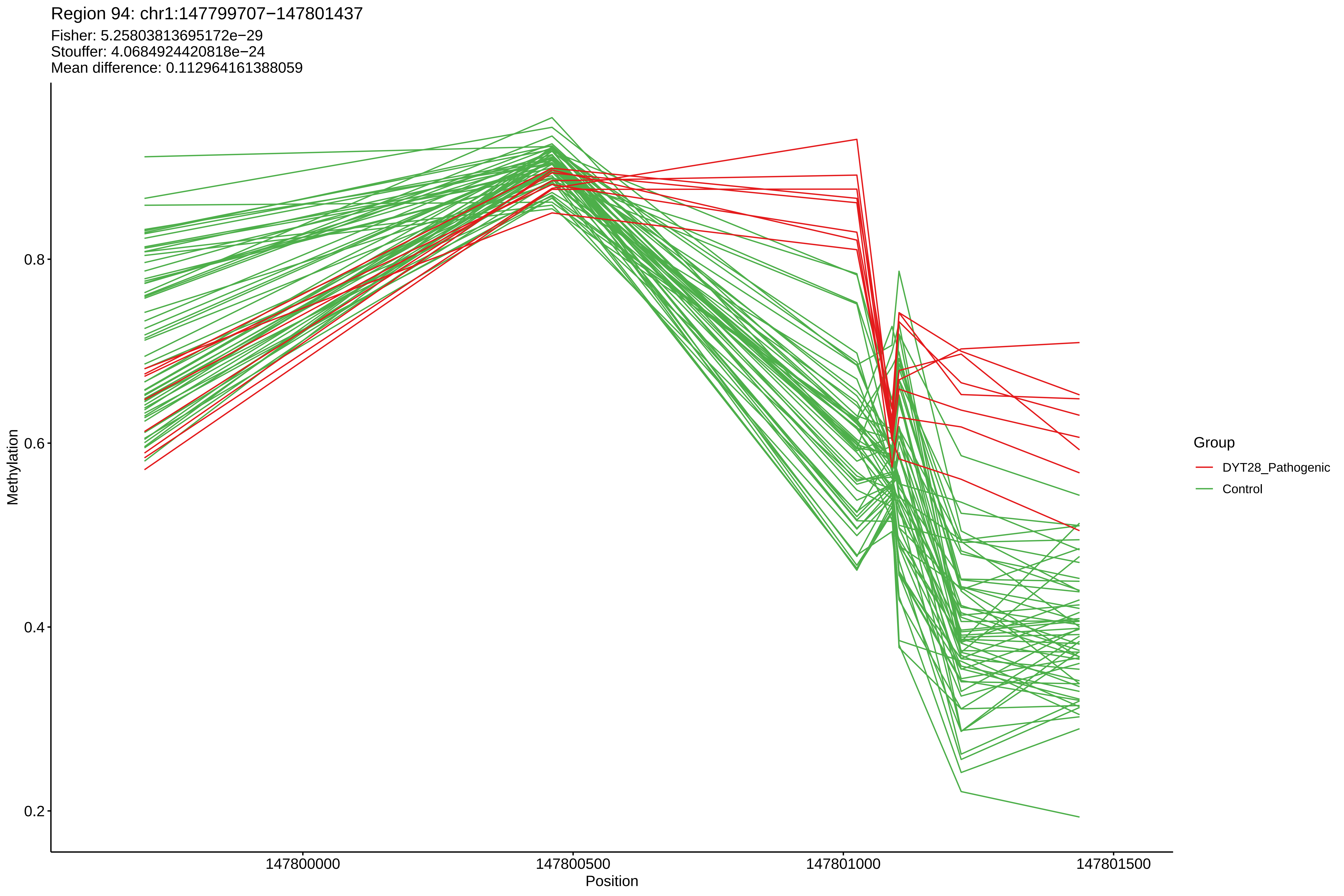

Region 94: chr1:147799707−147801437
Fisher: 5.25803813695172e−29
Stouffer: 4.0684924420818e−24
Mean difference: 0.112964161388059
Methylation
0.8
0.6
0.4
0.2
147800000
147800500
147801000
147801500
Position
Group
DYT28_Pathogenic
Control

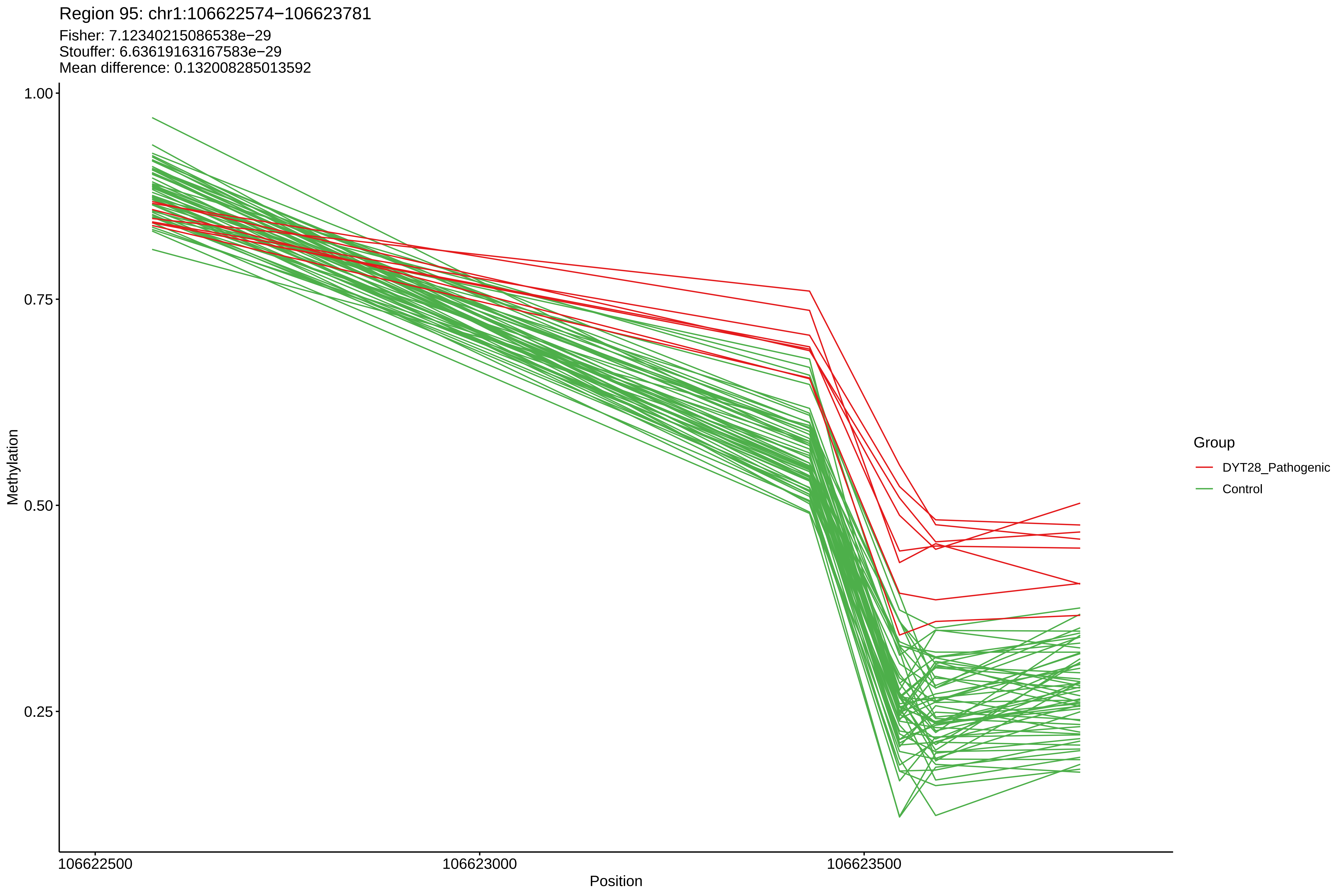
Region 95: chr1:106622574−106623781
Fisher: 7.12340215086538e−29
Stouffer: 6.63619163167583e−29
Mean difference: 0.132008285013592
Methylation
1.00
0.75
0.50
0.25
106622500
106623000
106623500
Position
Group
DYT28_Pathogenic
Control

Region 96: chr7:89975159−89976216

Fisher: 2.49672664842777e−28
Stouffer: 2.63090500678596e−21
Mean difference: 0.102532570612731

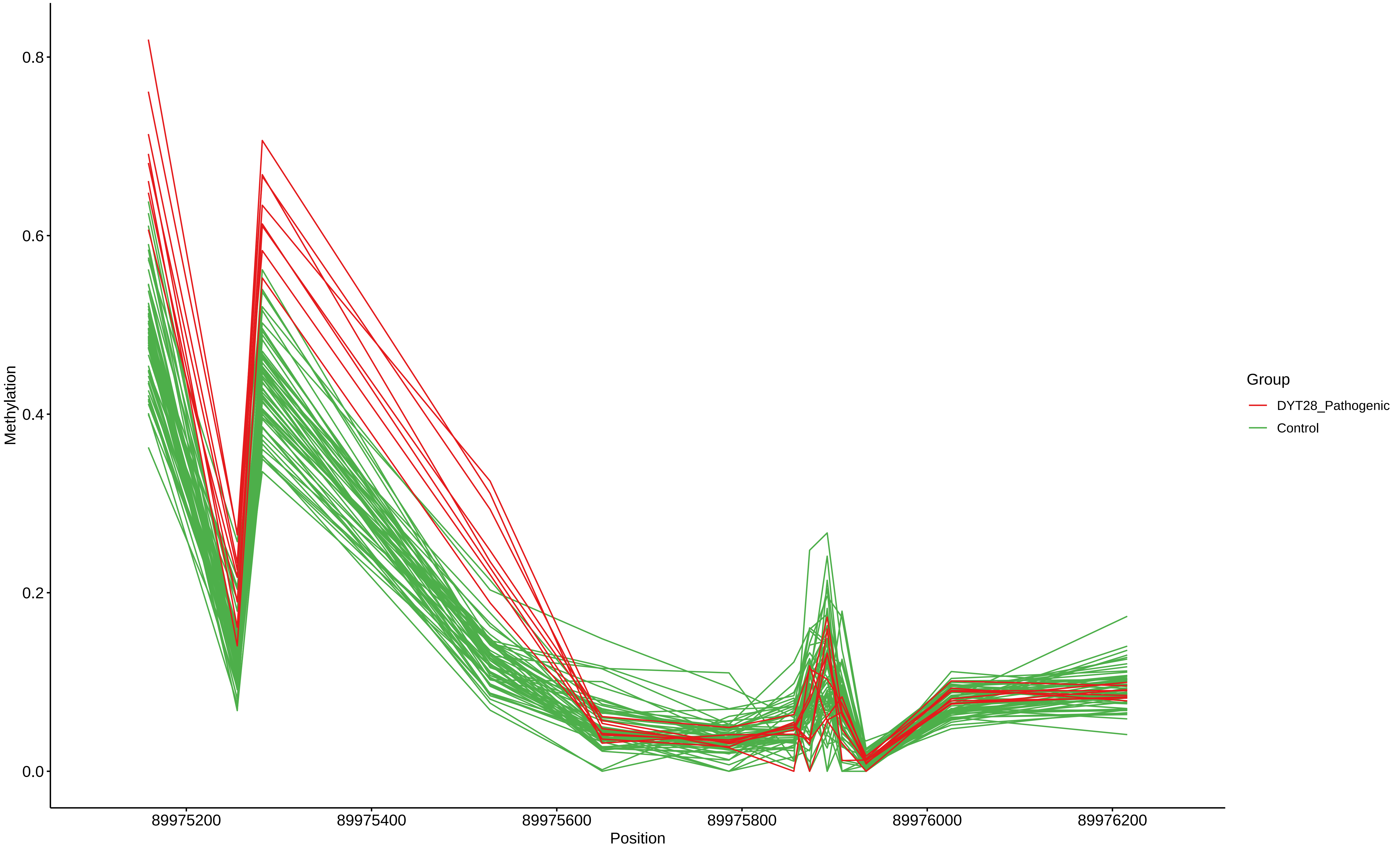

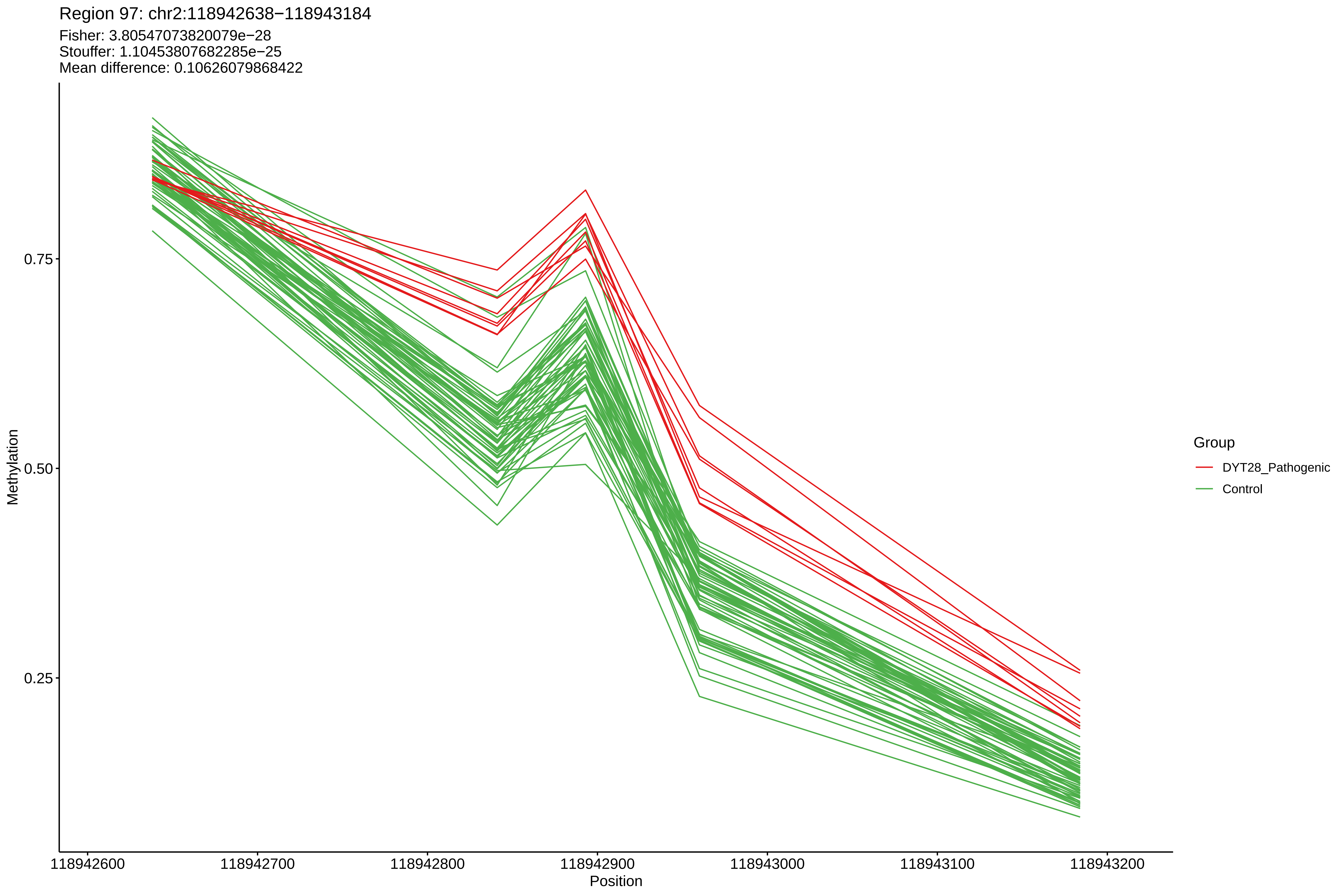
Region 97: chr2:118942638−118943184
Fisher: 3.80547073820079e−28
Stouffer: 1.10453807682285e−25
Mean difference: 0.10626079868422
Methylation
0.75
0.50
0.25
Position
118942600
118942700
118942800
118942900
118943000
118943100
118943200
Group
DYT28_Pathogenic
Control

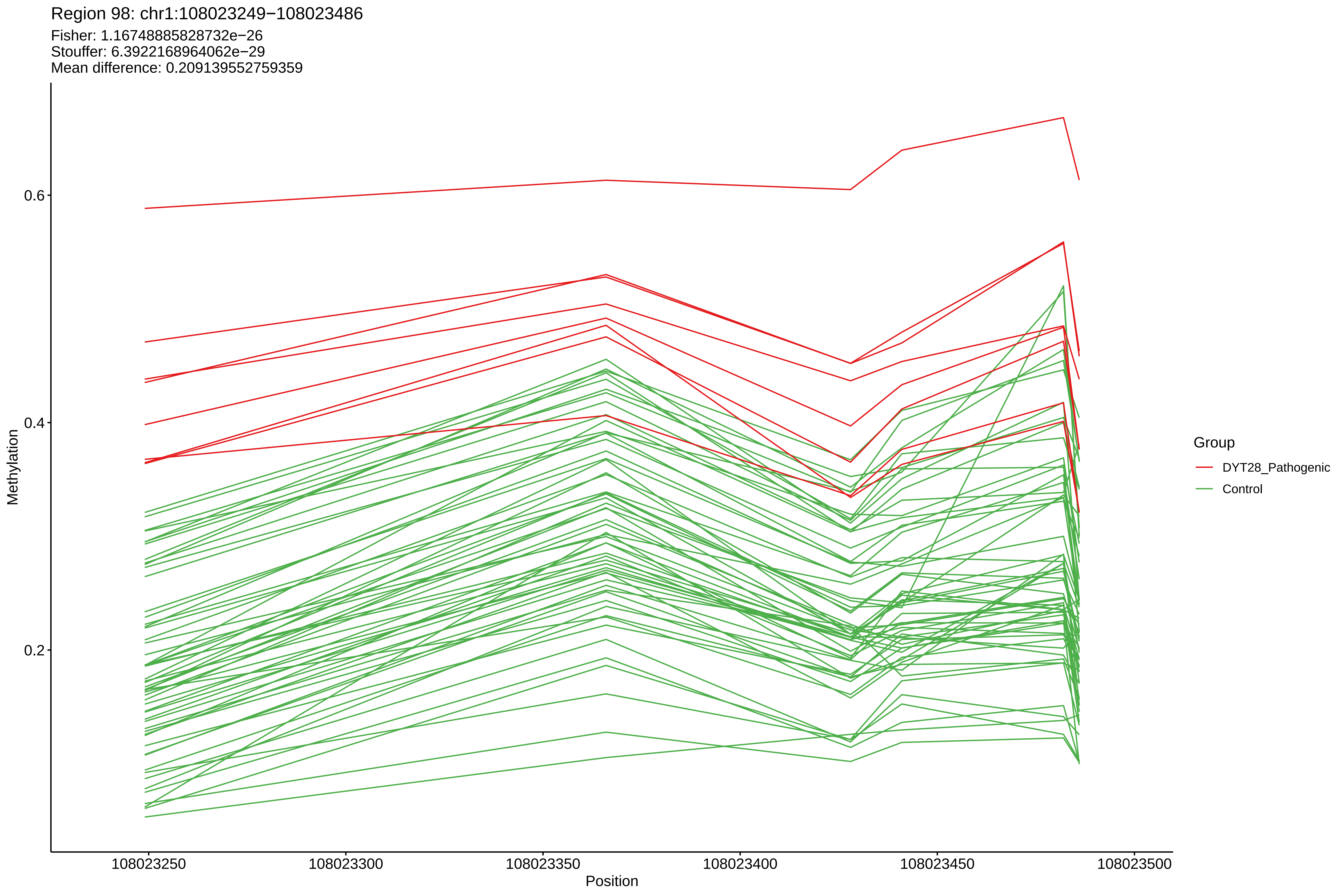

Region 98: chr1:108023249−108023486
Fisher: 1.16748885828732e−26
Stouffer: 6.3922168964062e−29
Mean difference: 0.209139552759359
0.6
0.4
0.2
Methylation
108023250
108023300
108023350
108023400
108023450
108023500
Position
Group
DYT28_Pathogenic
Control

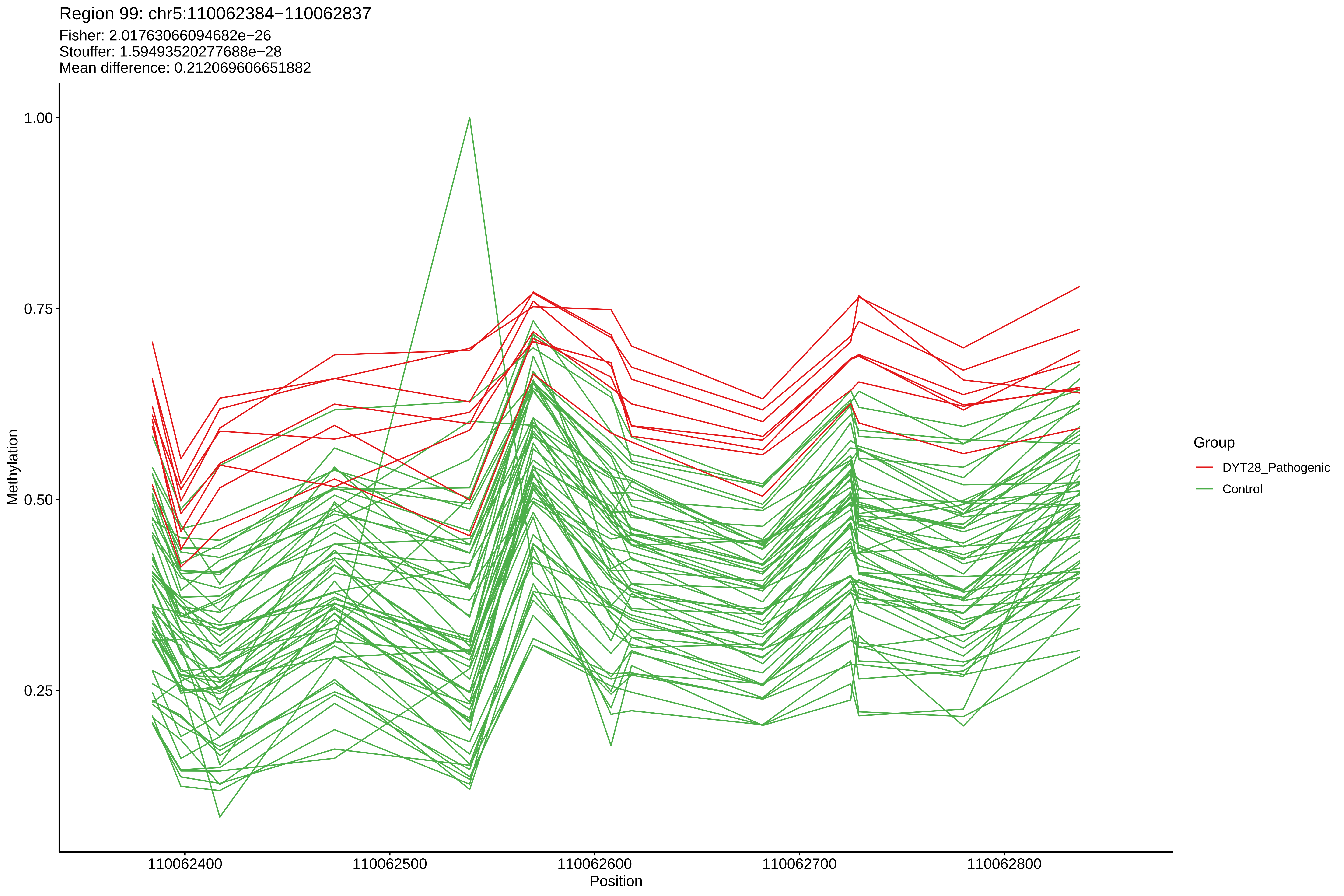
Region 99: chr5:110062384−110062837
Fisher: 2.01763066094682e−26
Stouffer: 1.59493520277688e−28
Mean difference: 0.212069606651882
Methylation
1.00
0.75
0.50
0.25
110062400
110062500
110062600
110062700
110062800
Position
Group
DYT28_Pathogenic
Control

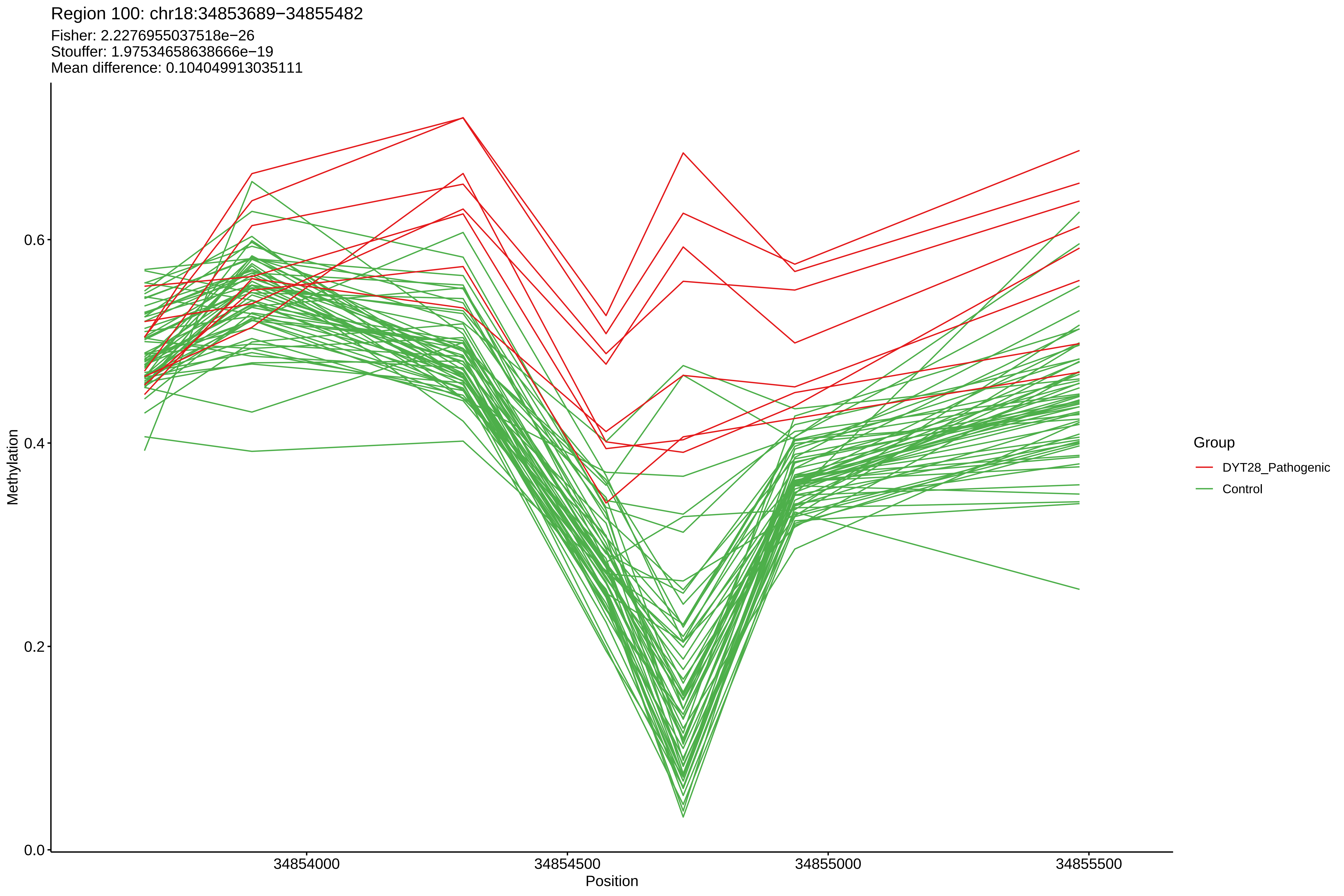
Region 100: chr18:34853689−34855482
Fisher: 2.2276955037518e−26
Stouffer: 1.97534658638666e−19
Mean difference: 0.104049913035111
Methylation
0.0
0.2
0.4
0.6
34854000
34854500
34855000
34855500
Position
Group
DYT28_Pathogenic
Control

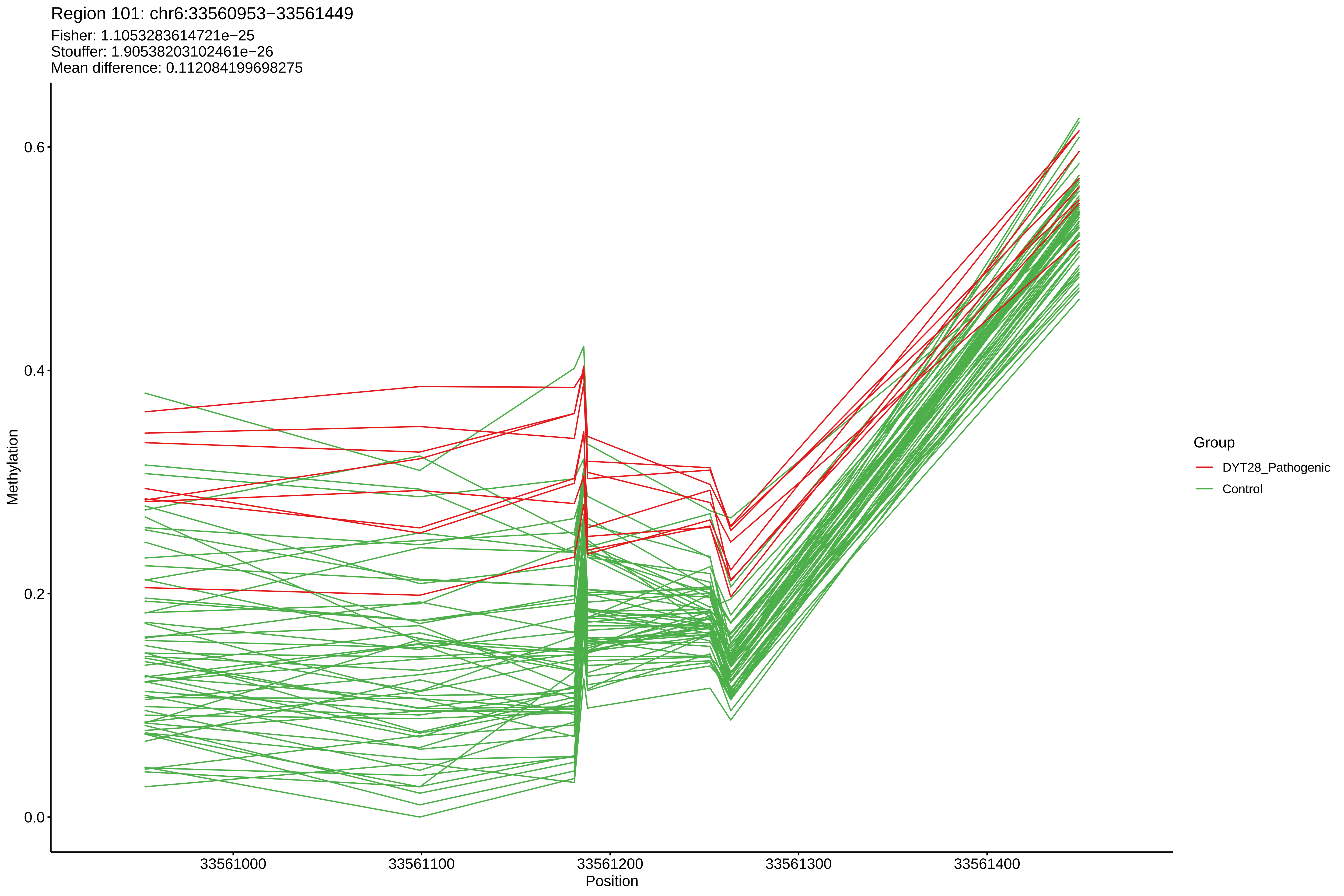

Region 101: chr6:33560953−33561449
Fisher: 1.1053283614721e−25
Stouffer: 1.90538203102461e−26
Mean difference: 0.112084199698275
Methylation
0.0
0.2
0.4
0.6
Position
33561000
33561100
33561200
33561300
33561400
Group
DYT28_Pathogenic
Control

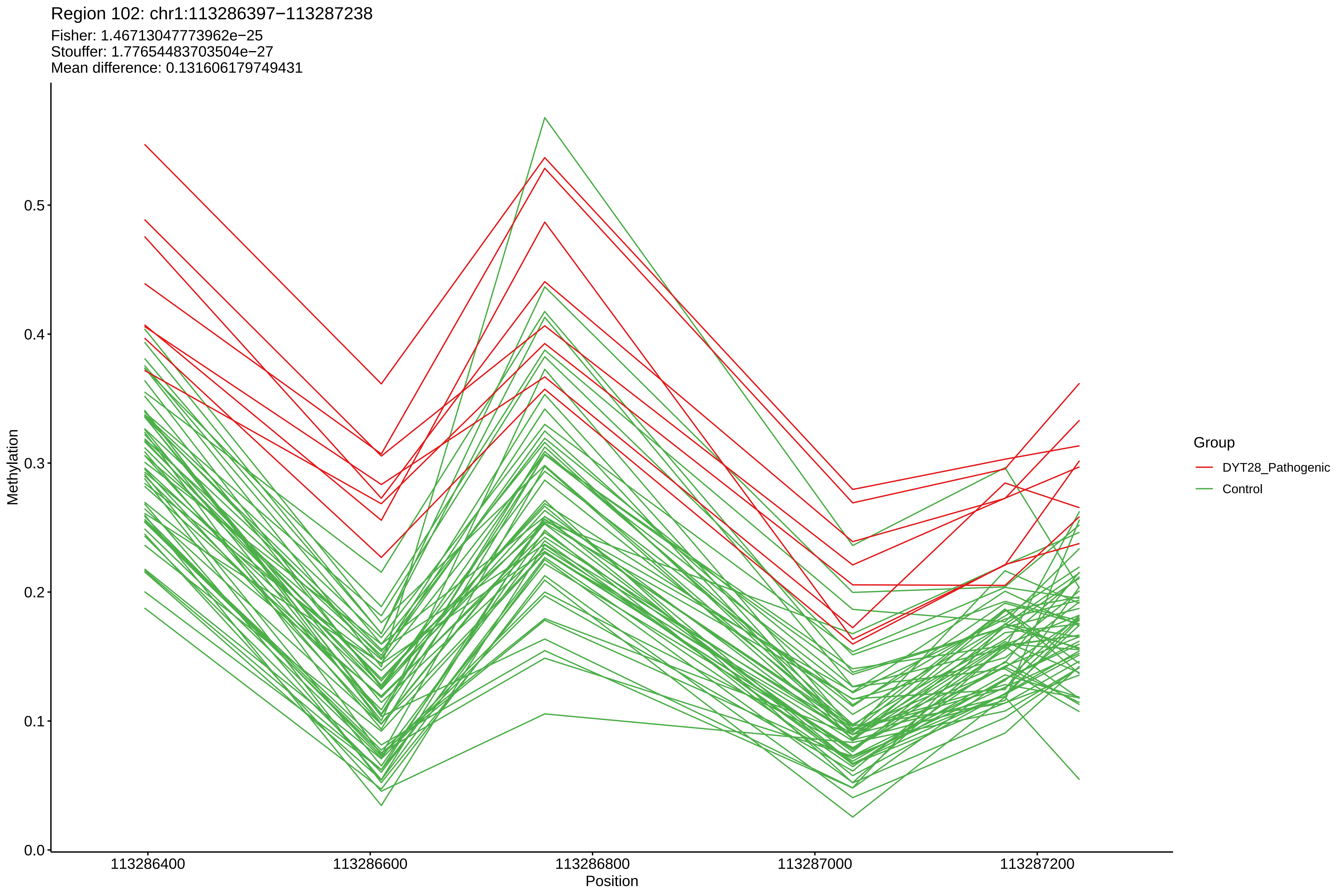
Region 102: chr1:113286397−113287238
Fisher: 1.46713047773962e−25
Stouffer: 1.77654483703504e−27
Mean difference: 0.131606179749431
Methylation
0.0
0.1
0.2
0.3
0.4
0.5
113286400
113286600
113286800
113287000
113287200
Position
Group
DYT28_Pathogenic
Control

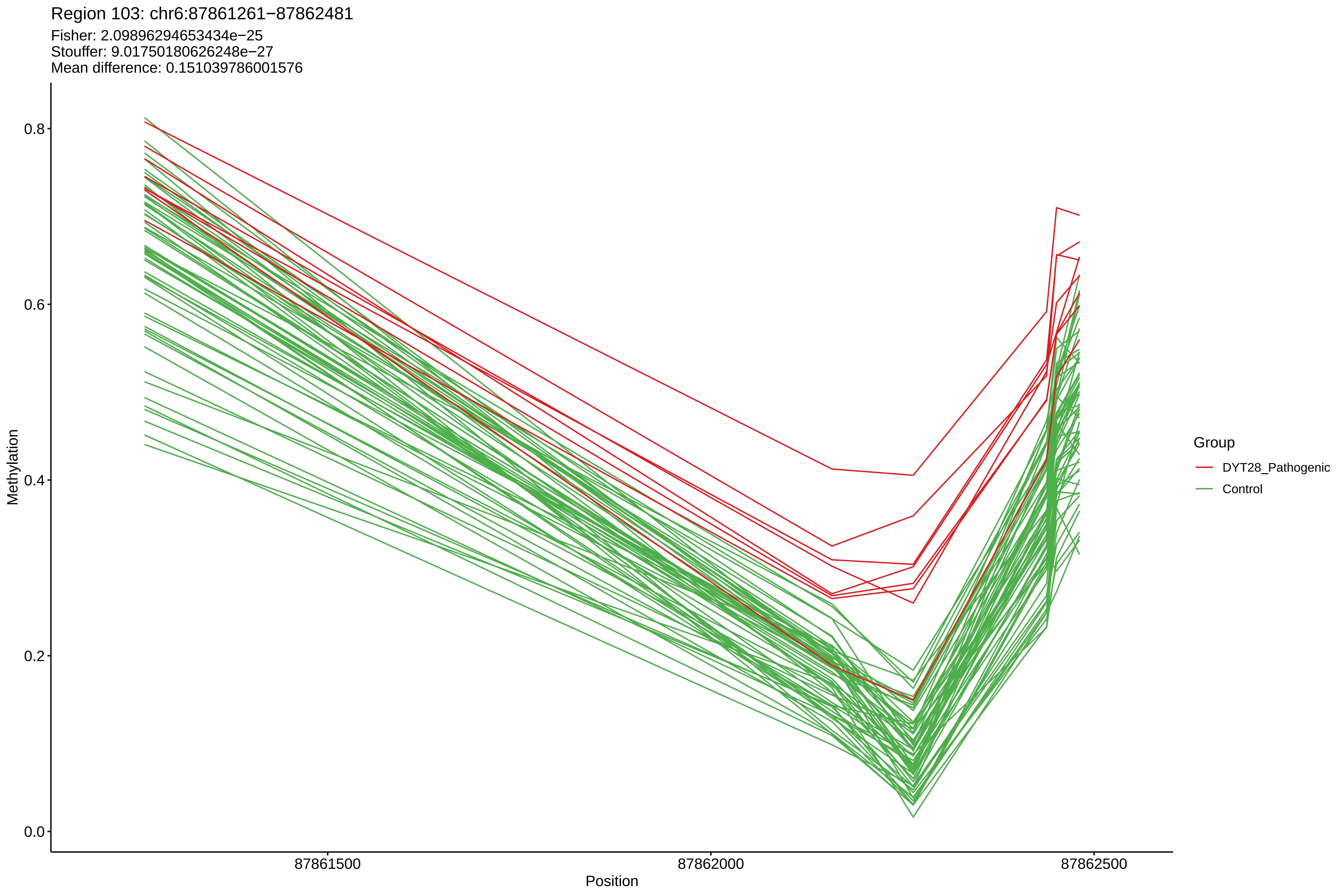
Region 103: chr6:87861261−87862481
Fisher: 2.09896294653434e−25
Stouffer: 9.01750180626248e−27
Mean difference: 0.151039786001576
Methylation
0.0
0.2
0.4
0.6
0.8
87861500
87862000
87862500
Position
Group
DYT28_Pathogenic
Control

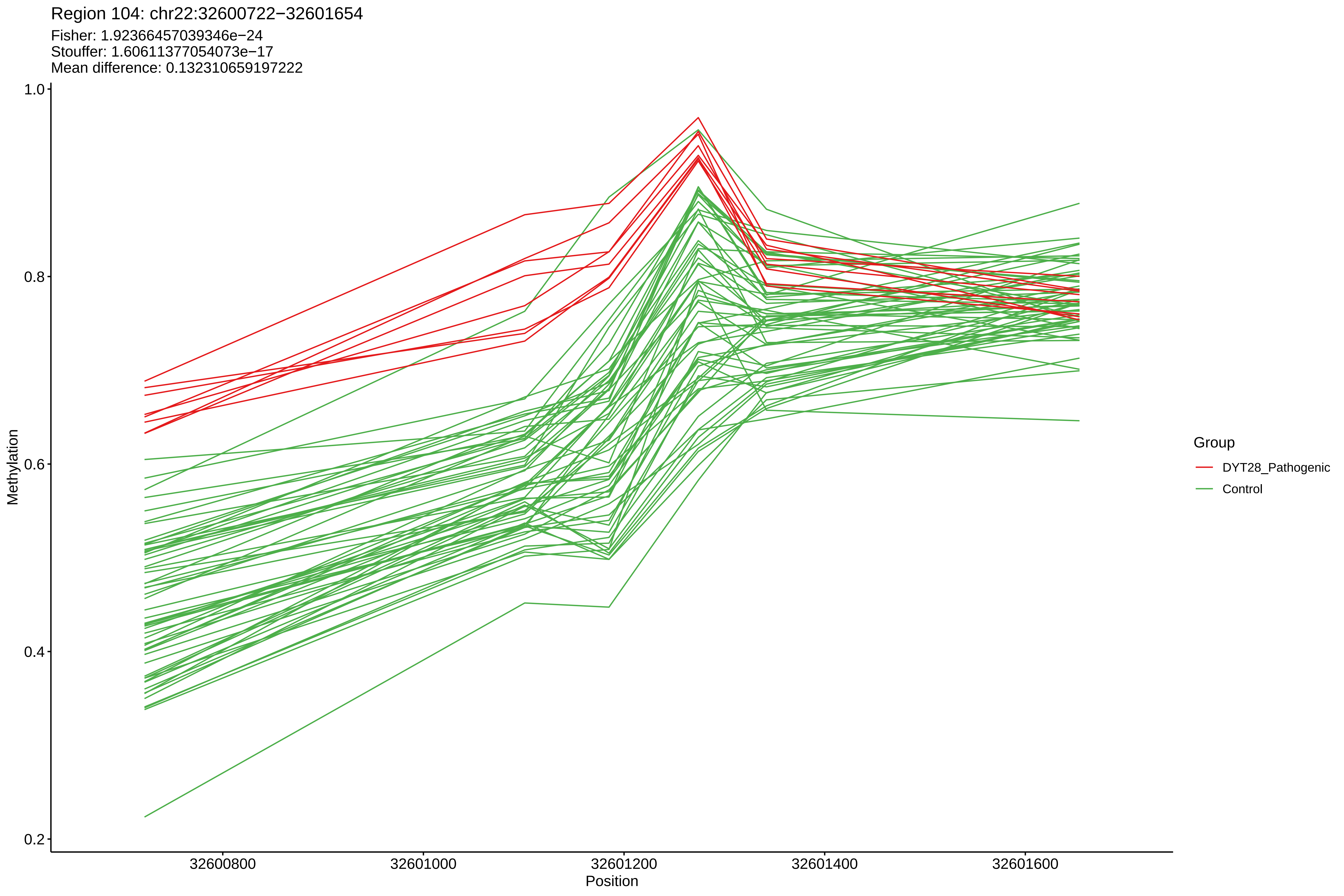
Region 104: chr22:32600722−32601654
Fisher: 1.92366457039346e−24
Stouffer: 1.60611377054073e−17
Mean difference: 0.132310659197222
Methylation
1.0
0.8
0.6
0.4
0.2
32600800
32601000
32601200
32601400
32601600
Position
Group
DYT28_Pathogenic
Control

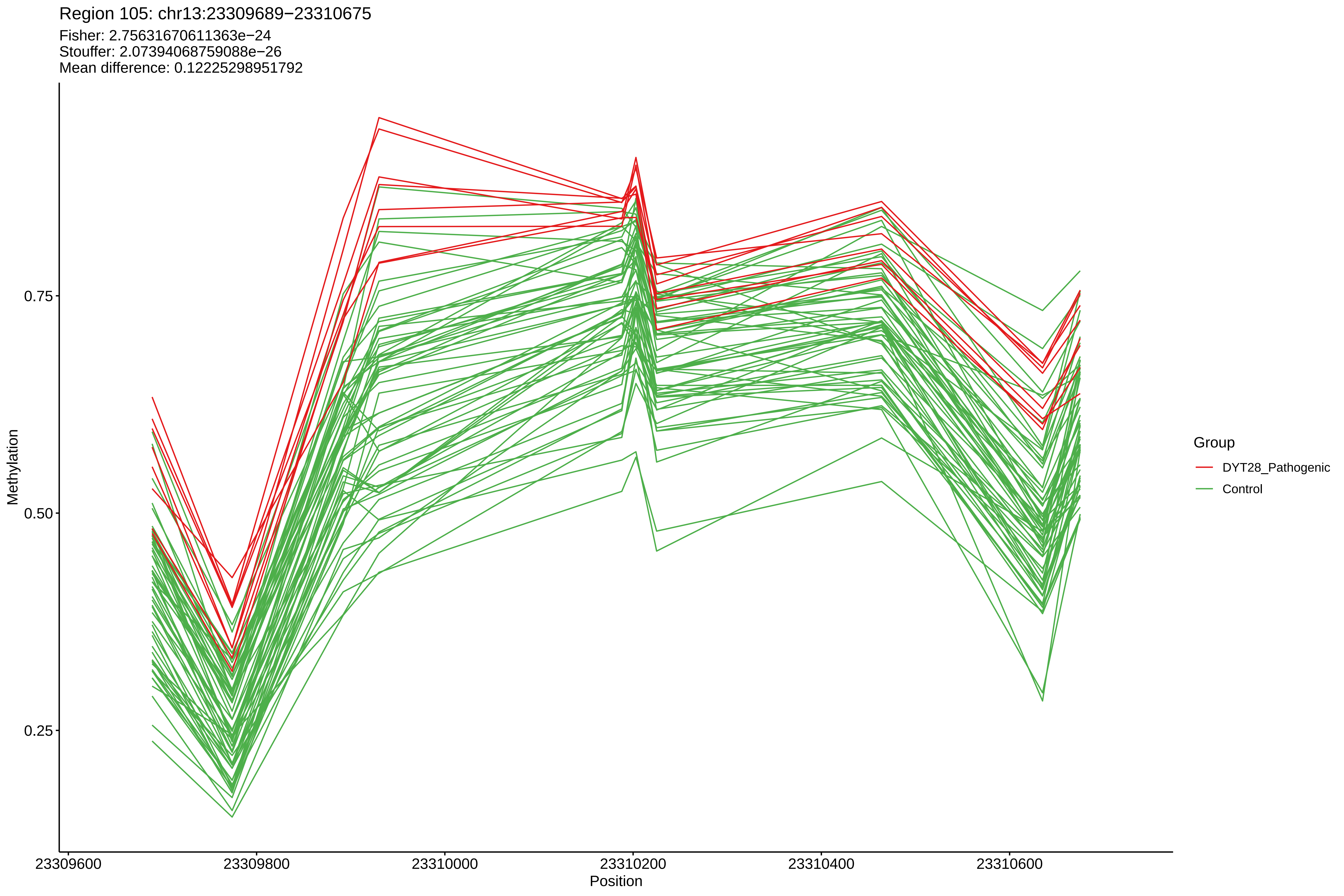
Region 105: chr13:23309689−23310675
Fisher: 2.75631670611363e−24
Stouffer: 2.07394068759088e−26
Mean difference: 0.12225298951792
Methylation
0.75
0.50
0.25
23309600
23309800
23310000
23310200
23310400
23310600
Position
Group
DYT28_Pathogenic
Control

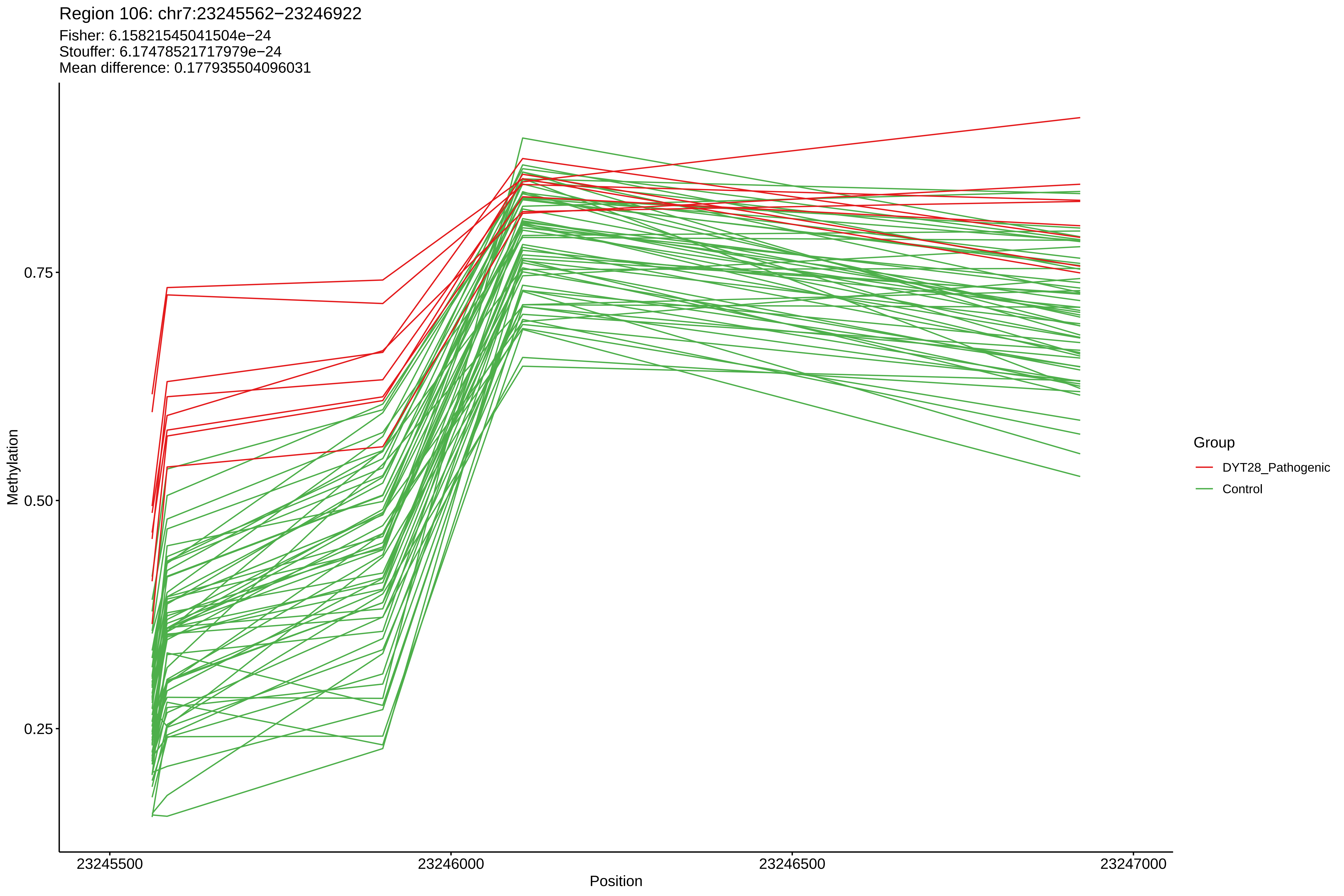
Region 106: chr7:23245562−23246922
Fisher: 6.15821545041504e−24
Stouffer: 6.17478521717979e−24
Mean difference: 0.177935504096031
Methylation
0.75
0.50
0.25
Position
23245500
23246000
23246500
23247000
Group
DYT28_Pathogenic
Control

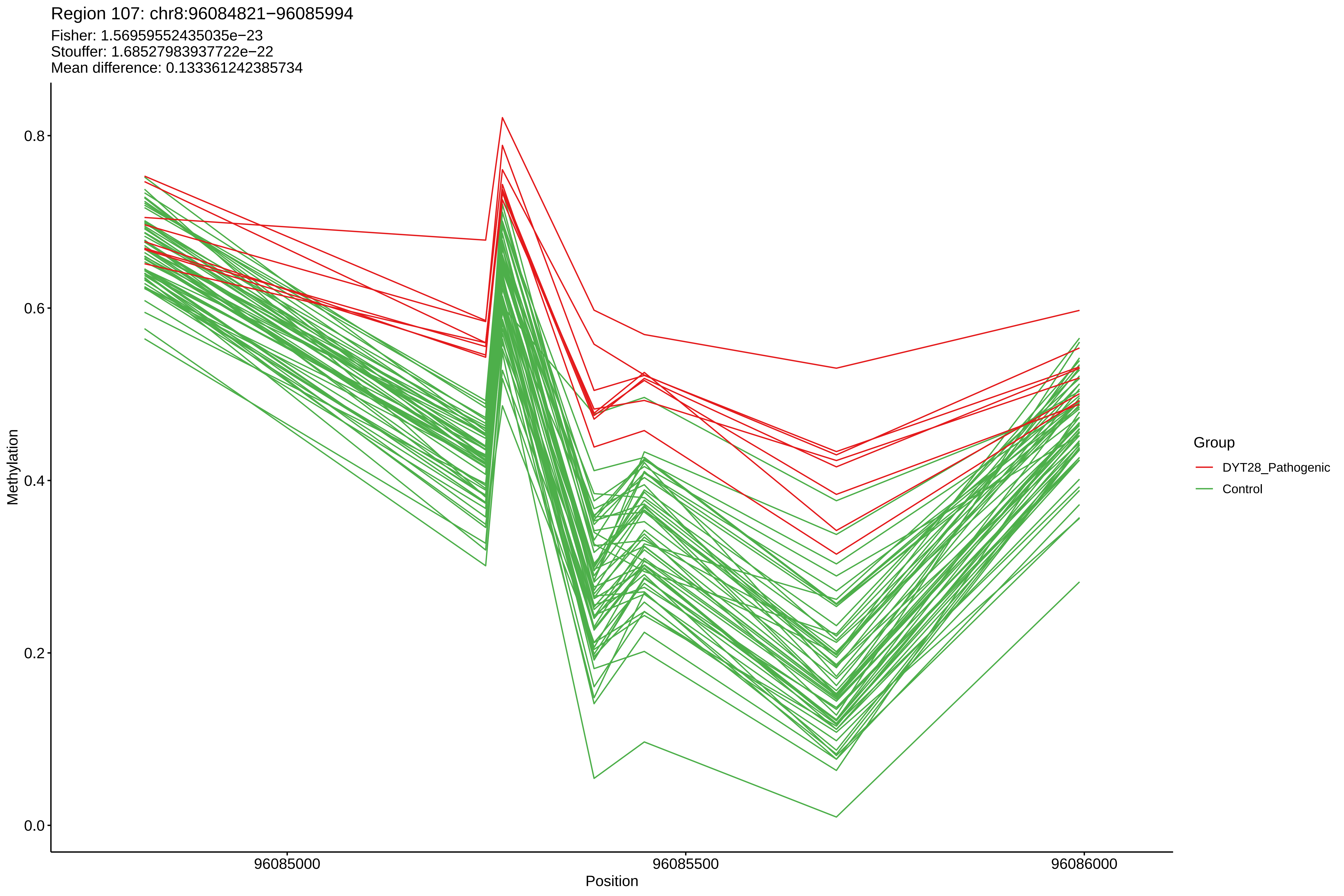
Region 107: chr8:96084821−96085994
Fisher: 1.56959552435035e−23
Stouffer: 1.68527983937722e−22
Mean difference: 0.133361242385734
Methylation
0.0
0.2
0.4
0.6
0.8
96085000
96085500
96086000
Position
Group
DYT28_Pathogenic
Control

Region 108: chr7:152622022−152623304
Fisher: 1.83807346540876e−23
Stouffer: 1.56641654606548e−24
Mean difference: 0.129229752880561

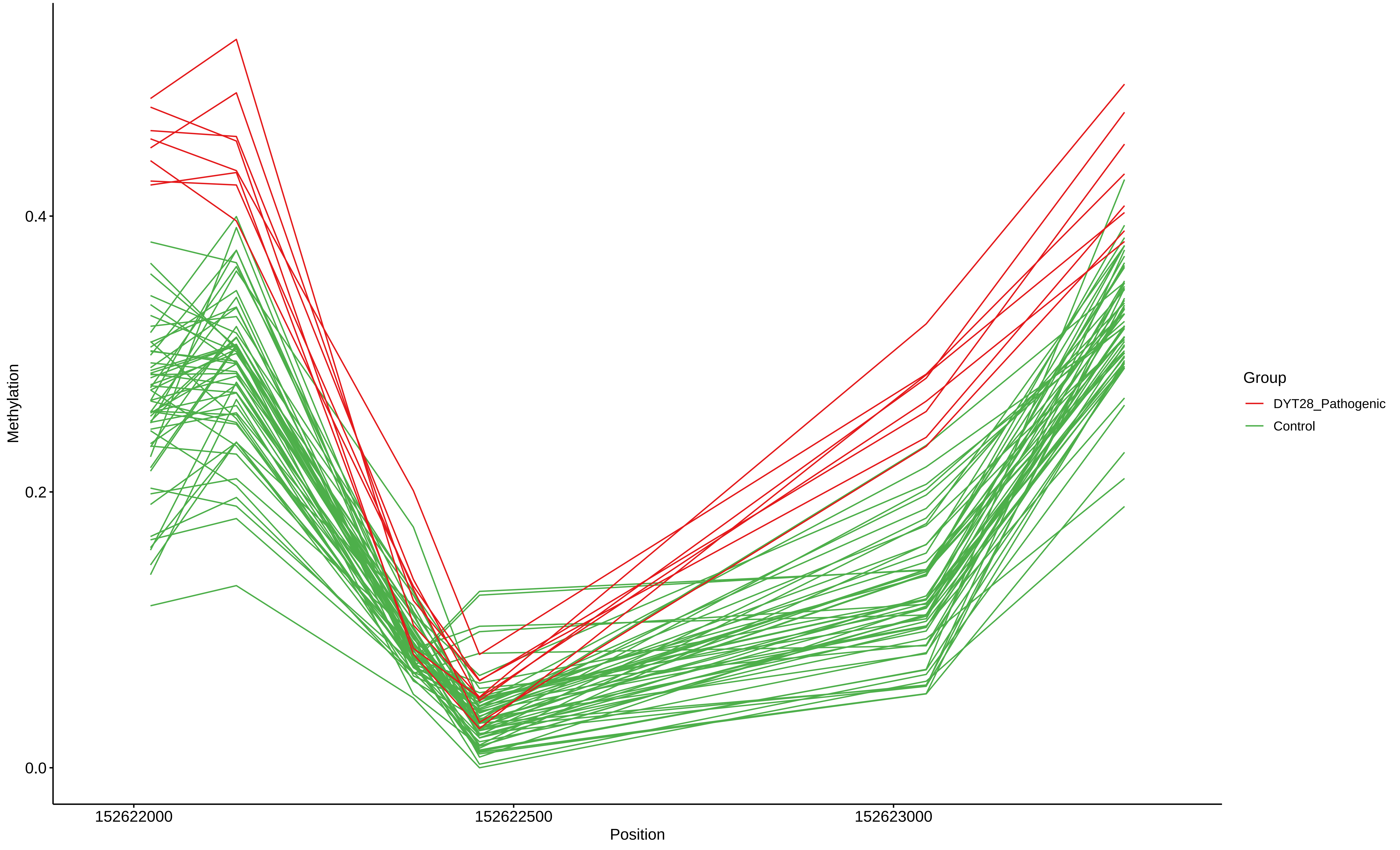

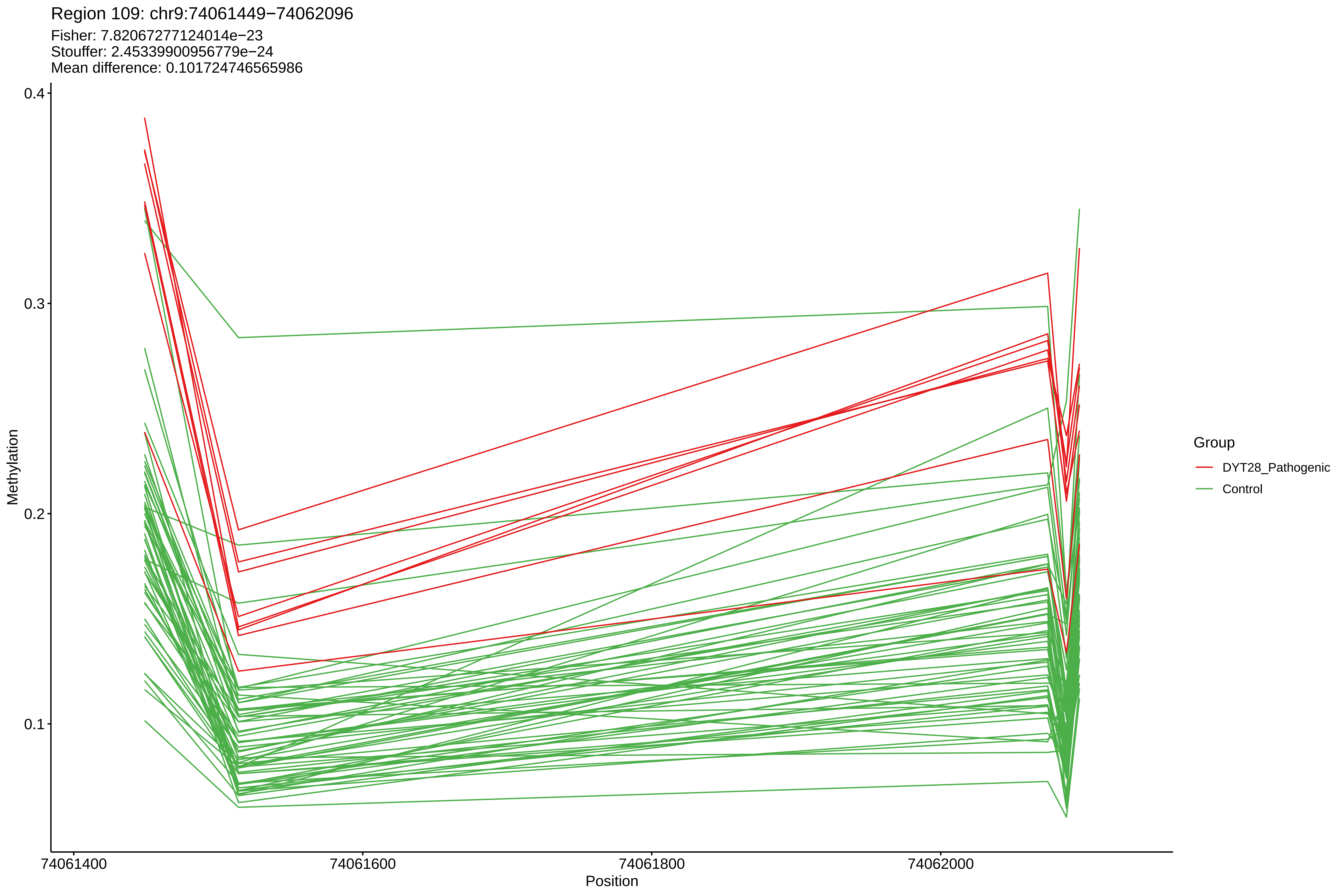
Region 109: chr9:74061449−74062096
Fisher: 7.82067277124014e−23
Stouffer: 2.45339900956779e−24
Mean difference: 0.101724746565986
Methylation
0.4
0.3
0.2
0.1
74061400
74061600
74061800
74062000
Position
Group
DYT28_Pathogenic
Control

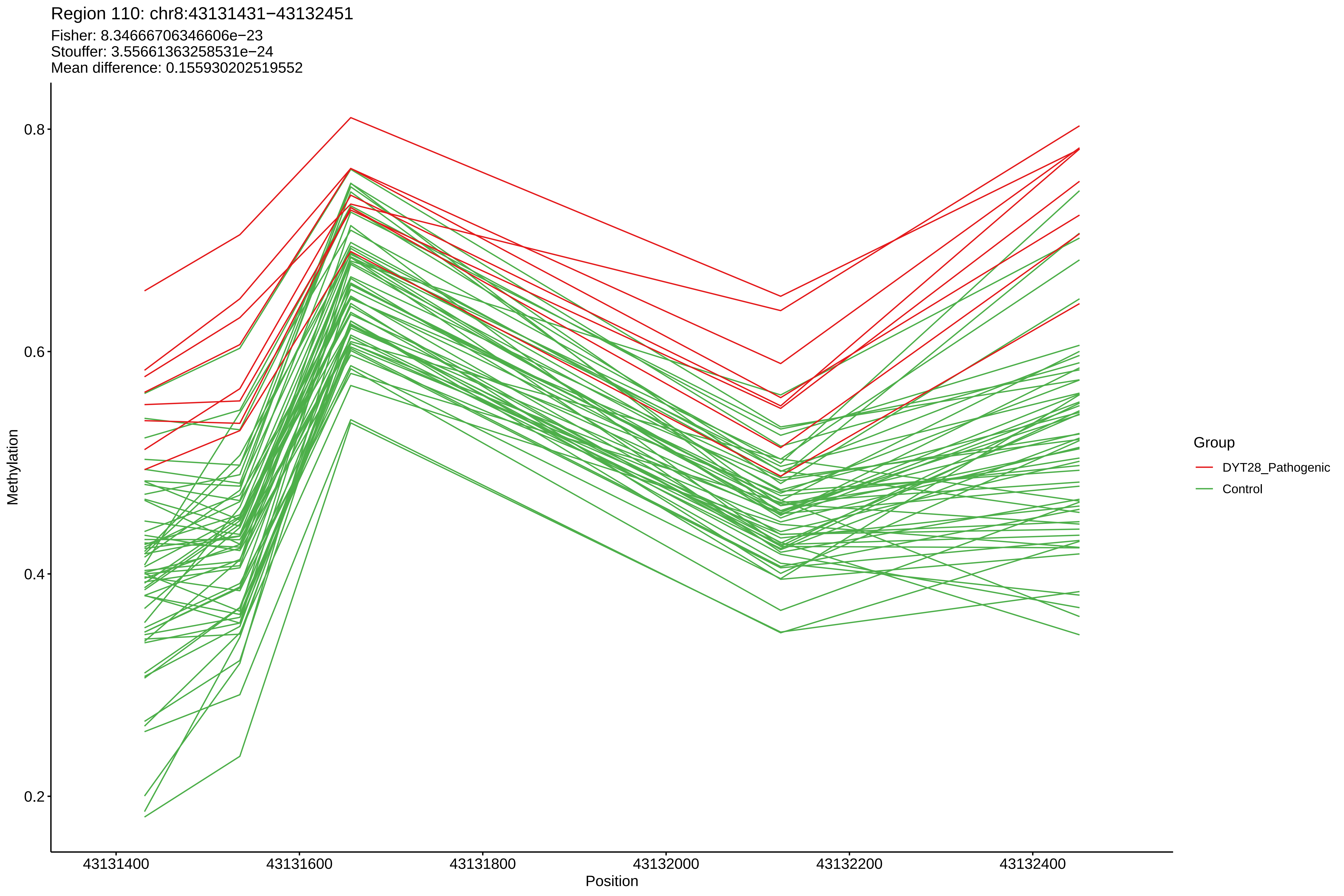
Region 110: chr8:43131431−43132451
Fisher: 8.34666706346606e−23
Stouffer: 3.55661363258531e−24
Mean difference: 0.155930202519552
Methylation
0.8
0.6
0.4
0.2
43131400
43131600
43131800
43132000
43132200
43132400
Position
Group
DYT28_Pathogenic
Control

Region 111: chr2:112895559−112896214
Fisher: 1.83763229511784e−22
Stouffer: 2.98207247582058e−21
Mean difference: 0.103063111380722

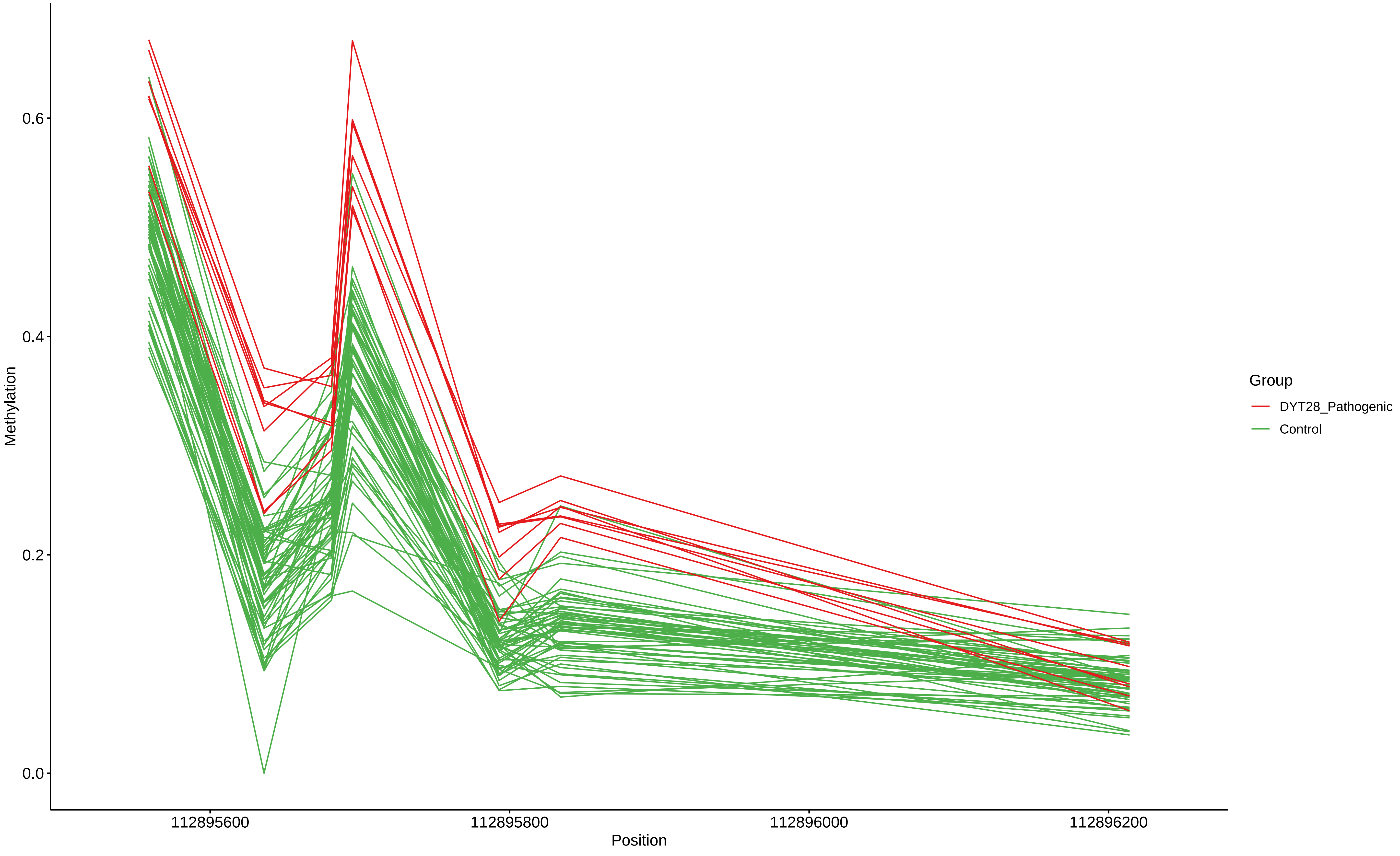

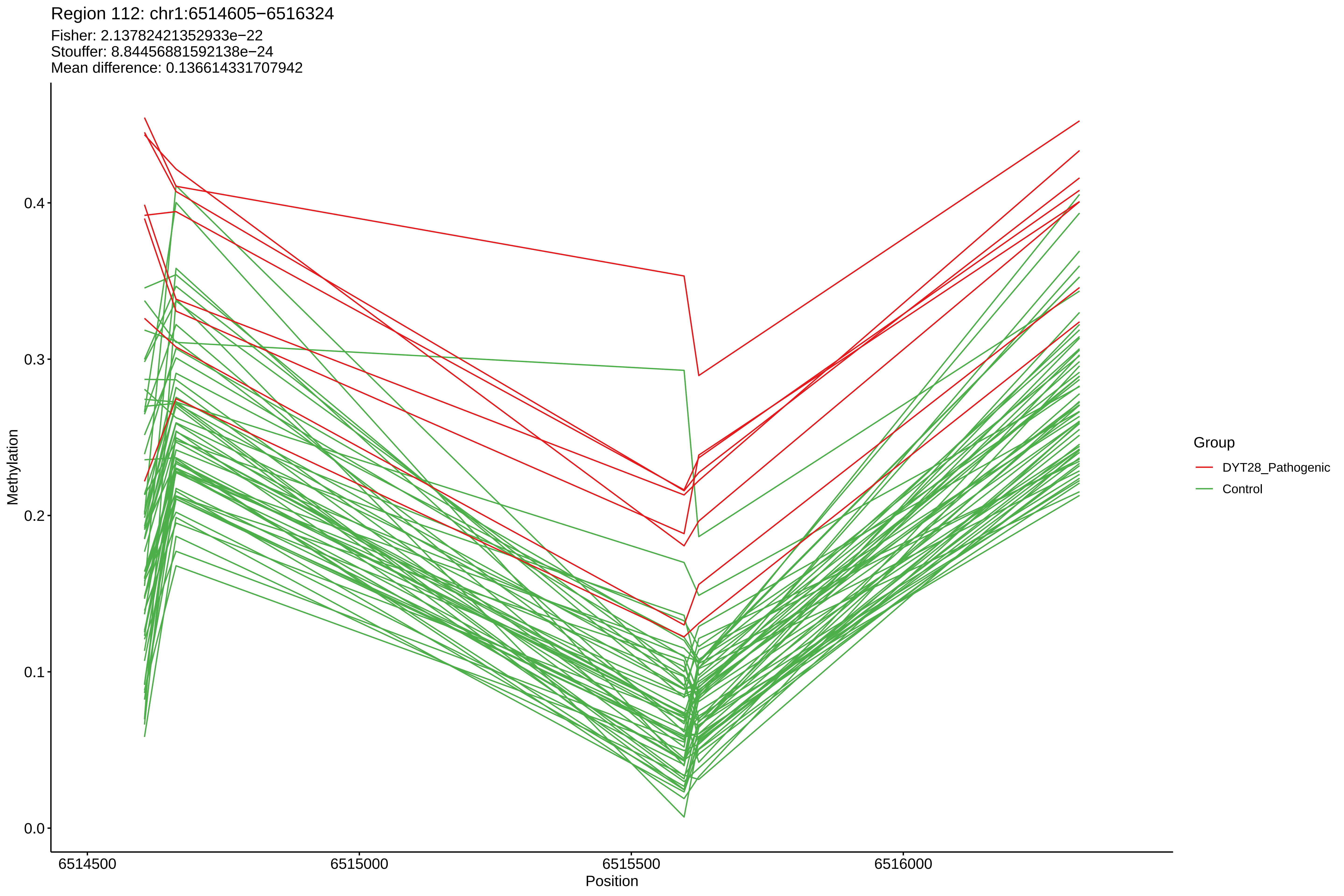
Region 112: chr1:6514605−6516324
Fisher: 2.13782421352933e−22
Stouffer: 8.84456881592138e−24
Mean difference: 0.136614331707942
Methylation
0.0
0.1
0.2
0.3
0.4
6514500
6515000
6515500
6516000
Position
Group
DYT28_Pathogenic
Control

Region 113: chr17:17109640−17110641

Fisher: 2.1656010989696e−22

Stouffer: 5.75452334157866e−16

Mean difference: 0.106976011011533

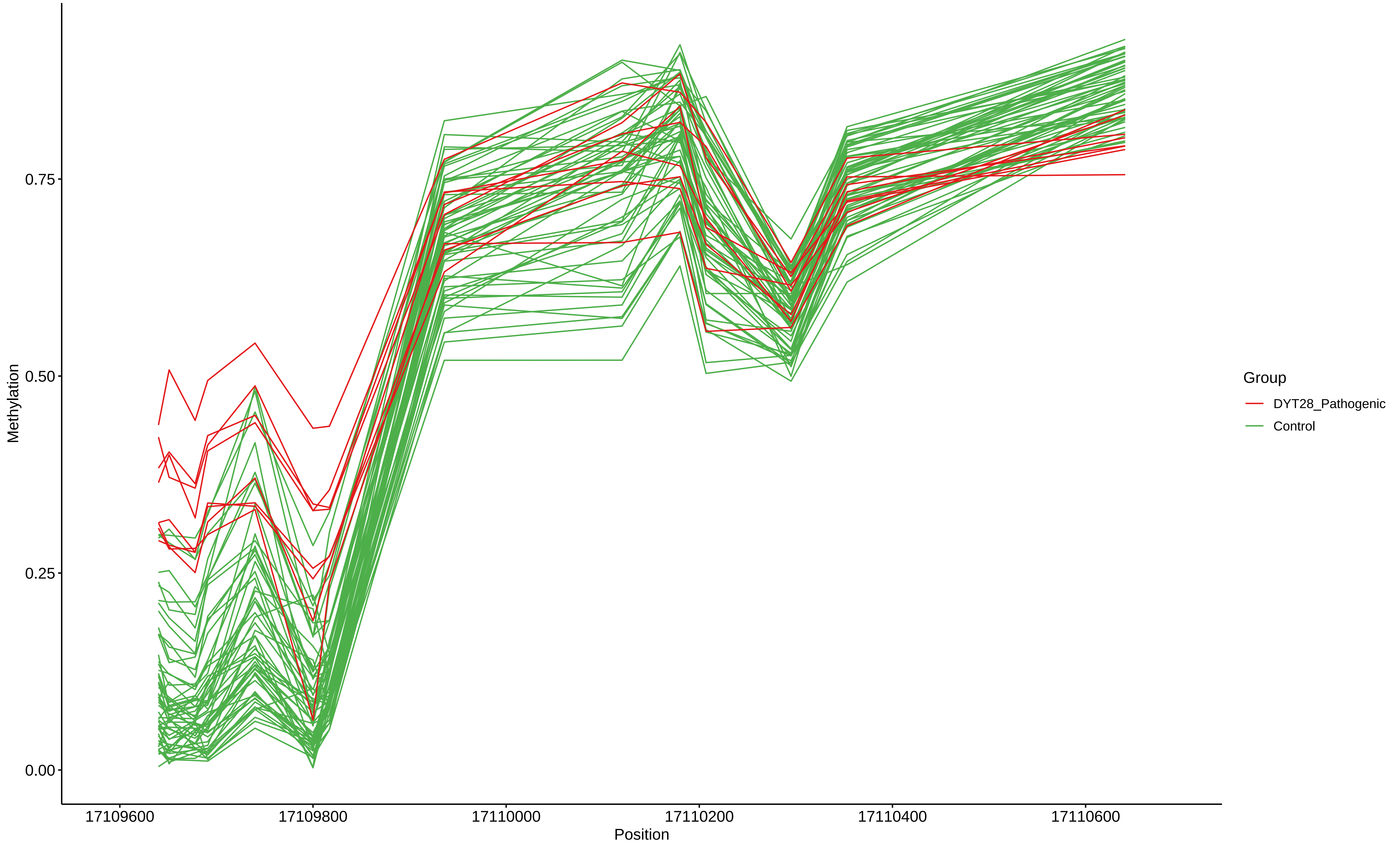

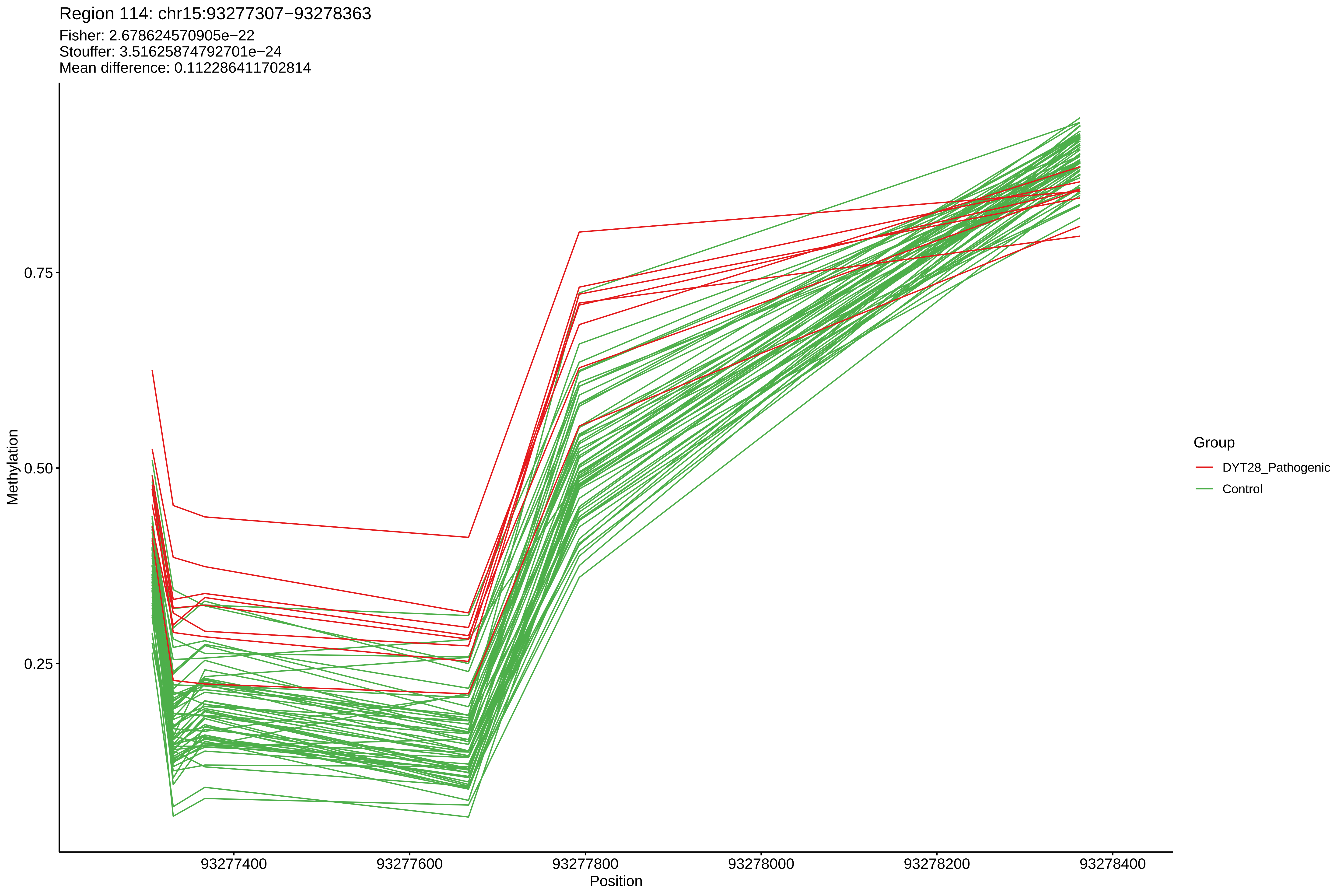
Region 114: chr15:93277307−93278363
Fisher: 2.678624570905e−22
Stouffer: 3.51625874792701e−24
Mean difference: 0.112286411702814
Methylation
0.75
0.50
0.25
93277400
93277600
93277800
93278000
93278200
93278400
Position
Group
DYT28_Pathogenic
Control

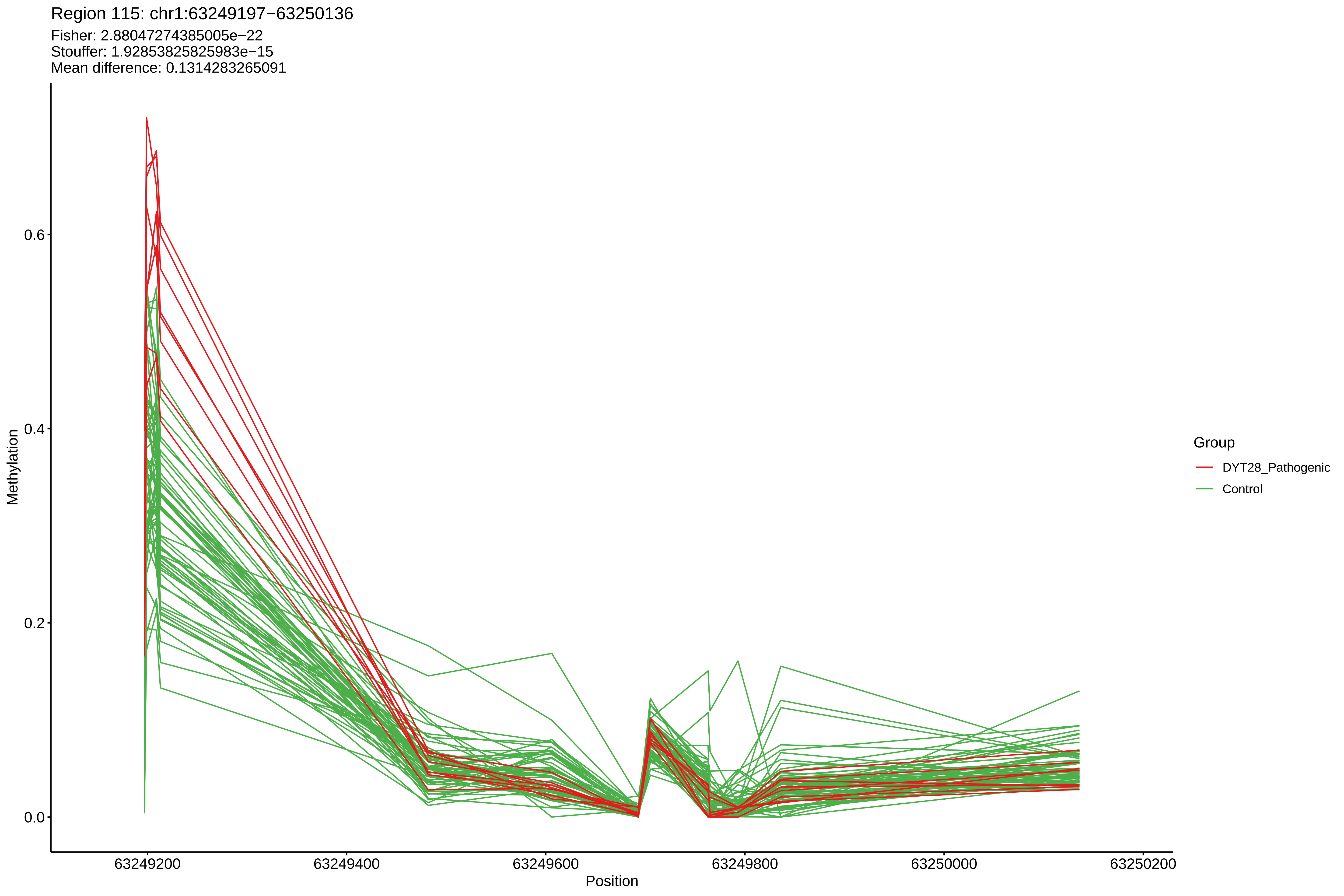

Region 115: chr1:63249197−63250136
Fisher: 2.88047274385005e−22
Stouffer: 1.92853825825983e−15
Mean difference: 0.1314283265091
Methylation
0.0
0.2
0.4
0.6
63249200
63249400
63249600
63249800
63250000
63250200
Position
Group
DYT28_Pathogenic
Control

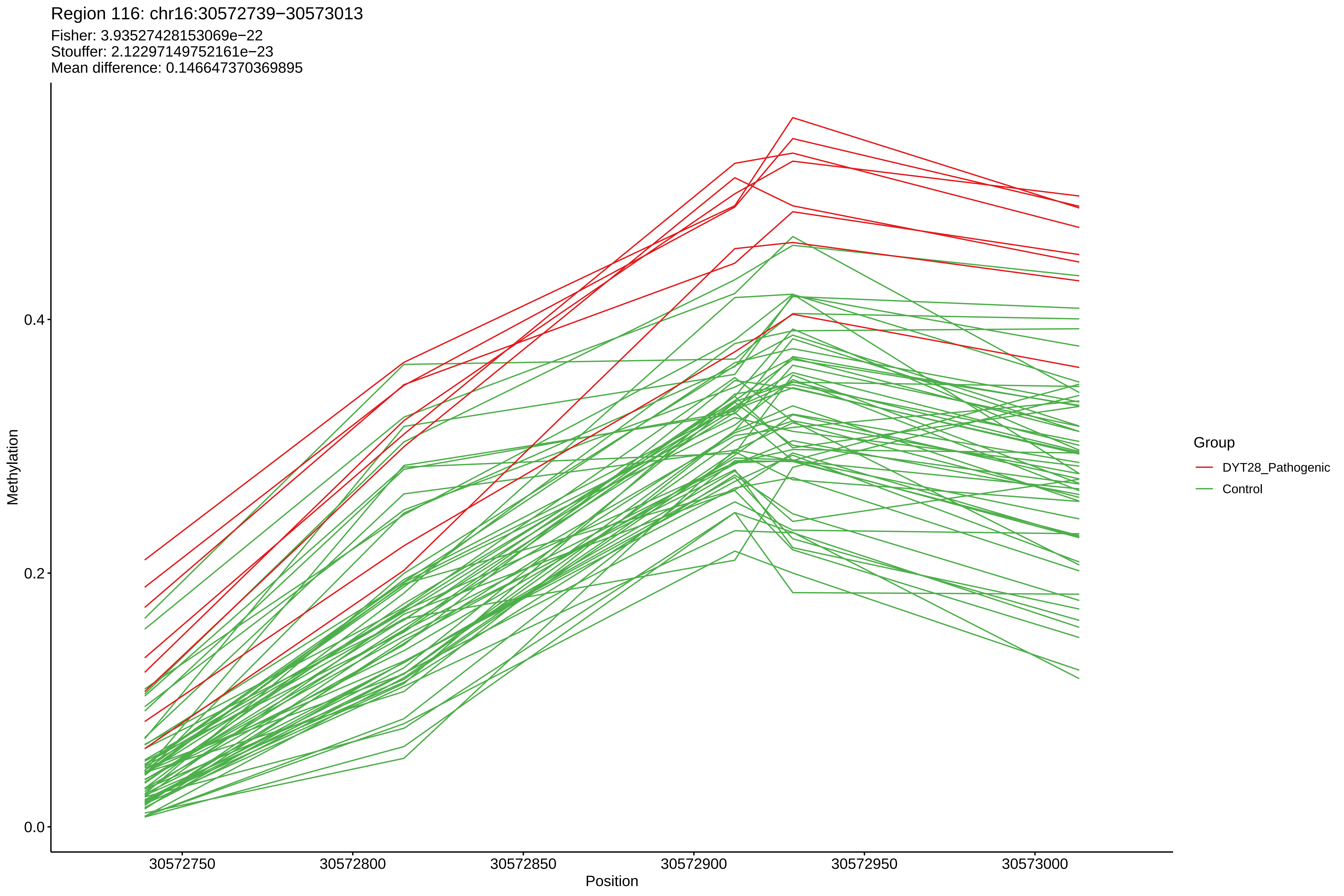
Region 116: chr16:30572739−30573013
Fisher: 3.93527428153069e−22
Stouffer: 2.12297149752161e−23
Mean difference: 0.146647370369895
Methylation
0.0
0.2
0.4
30572750
30572800
30572850
30572900
30572950
30573000
Position
Group
DYT28_Pathogenic
Control

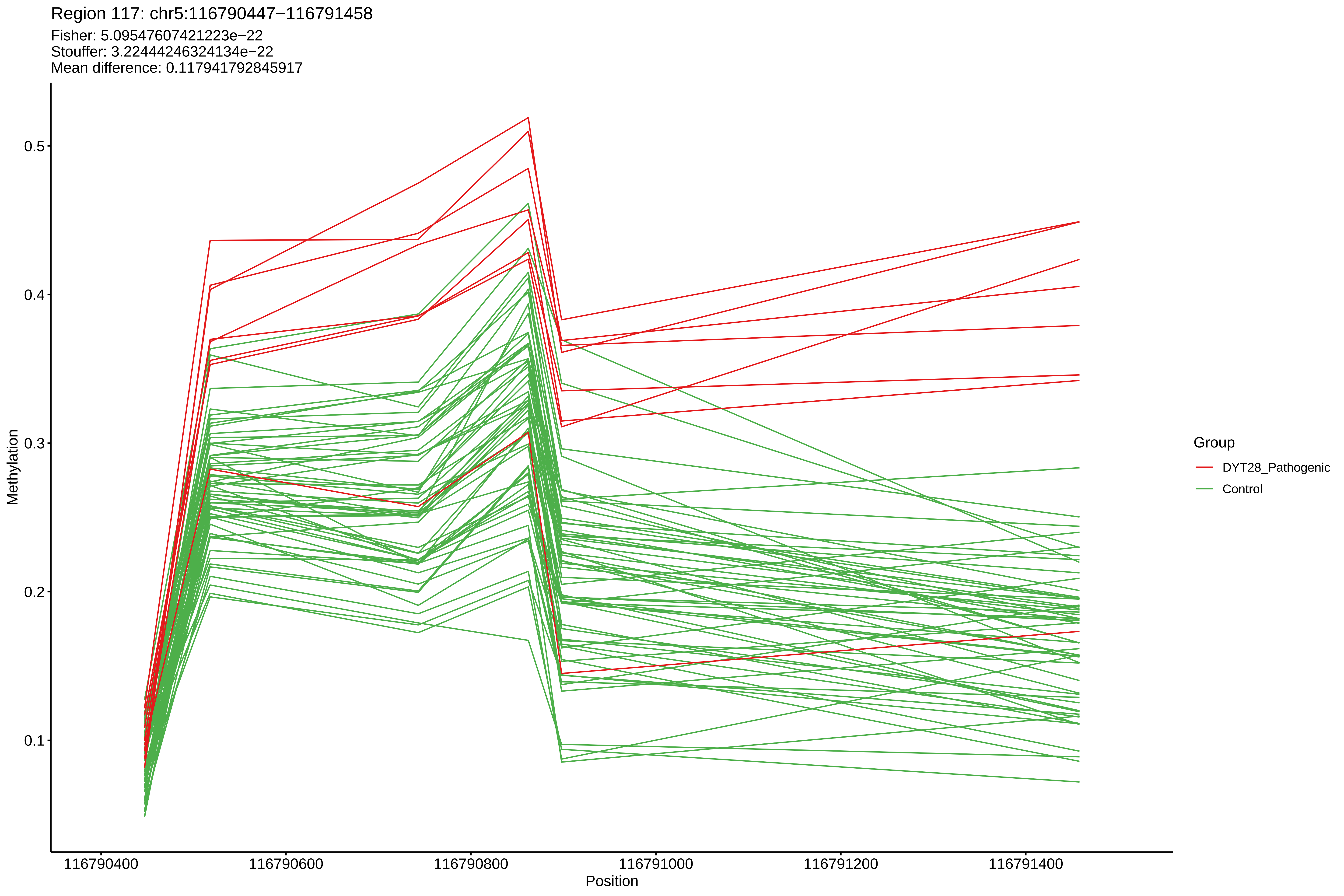

Region 117: chr5:116790447−116791458
Fisher: 5.09547607421223e−22
Stouffer: 3.22444246324134e−22
Mean difference: 0.117941792845917
Methylation
0.5
0.4
0.3
0.2
0.1
116790400
116790600
116790800
116791000
116791200
116791400
Position
Group
DYT28_Pathogenic
Control

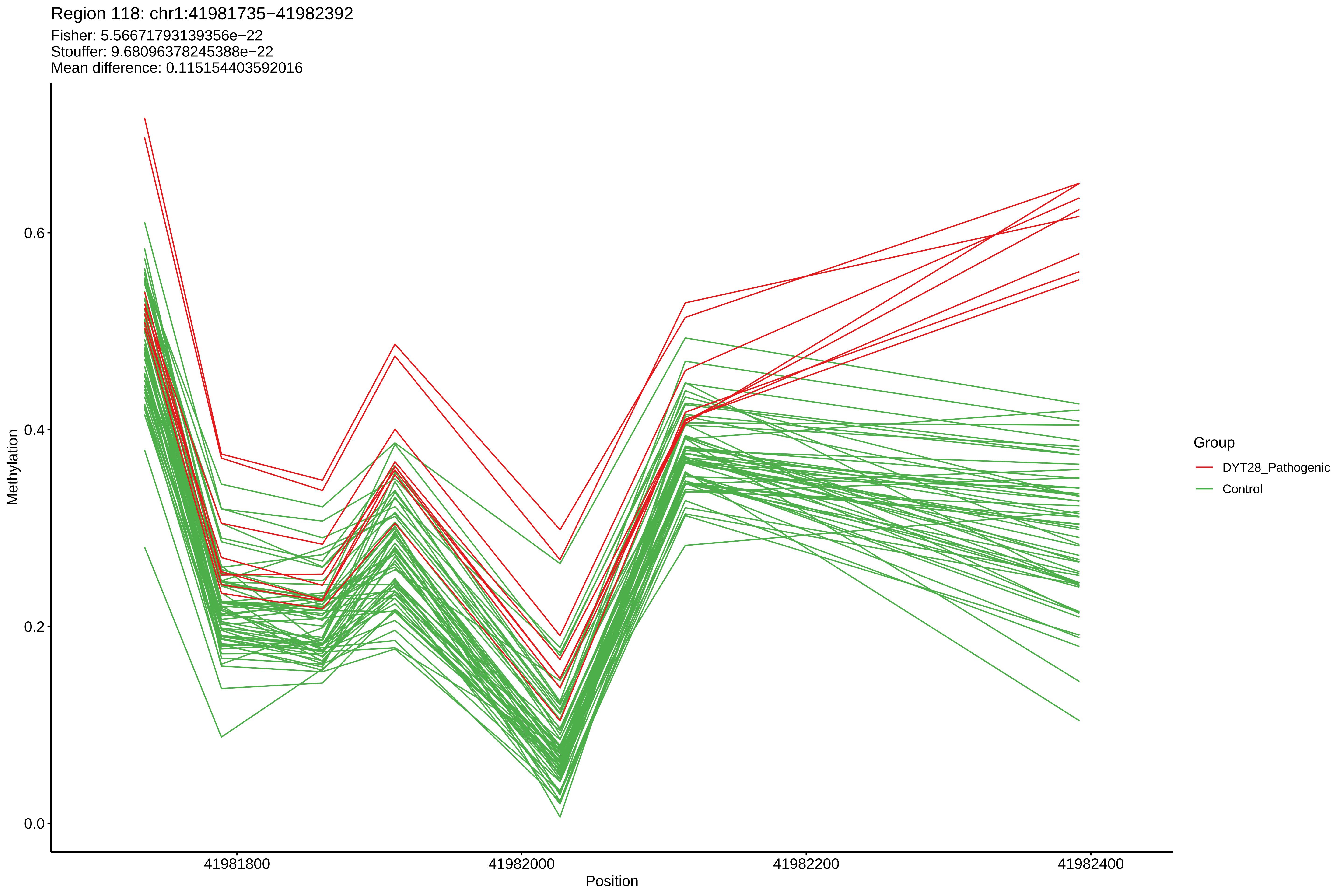
Region 118: chr1:41981735−41982392
Fisher: 5.56671793139356e−22
Stouffer: 9.68096378245388e−22
Mean difference: 0.115154403592016
Methylation
0.0
0.2
0.4
0.6
41981800
41982000
41982200
41982400
Position
Group
DYT28_Pathogenic
Control

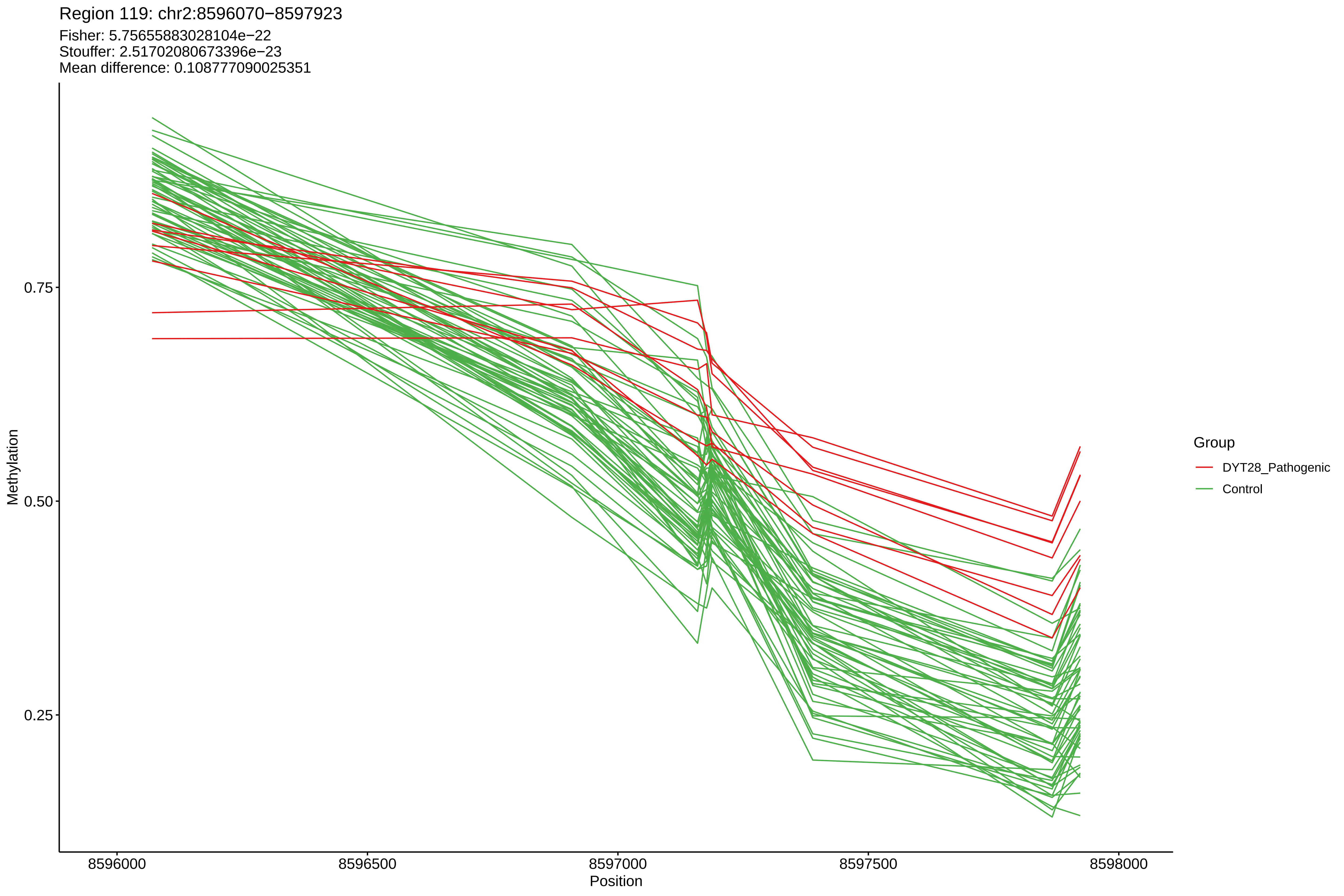
Region 119: chr2:8596070−8597923
Fisher: 5.75655883028104e−22
Stouffer: 2.51702080673396e−23
Mean difference: 0.108777090025351
Methylation
0.75
0.50
0.25
8596000
8596500
8597000
8597500
8598000
Position
Group
DYT28_Pathogenic
Control

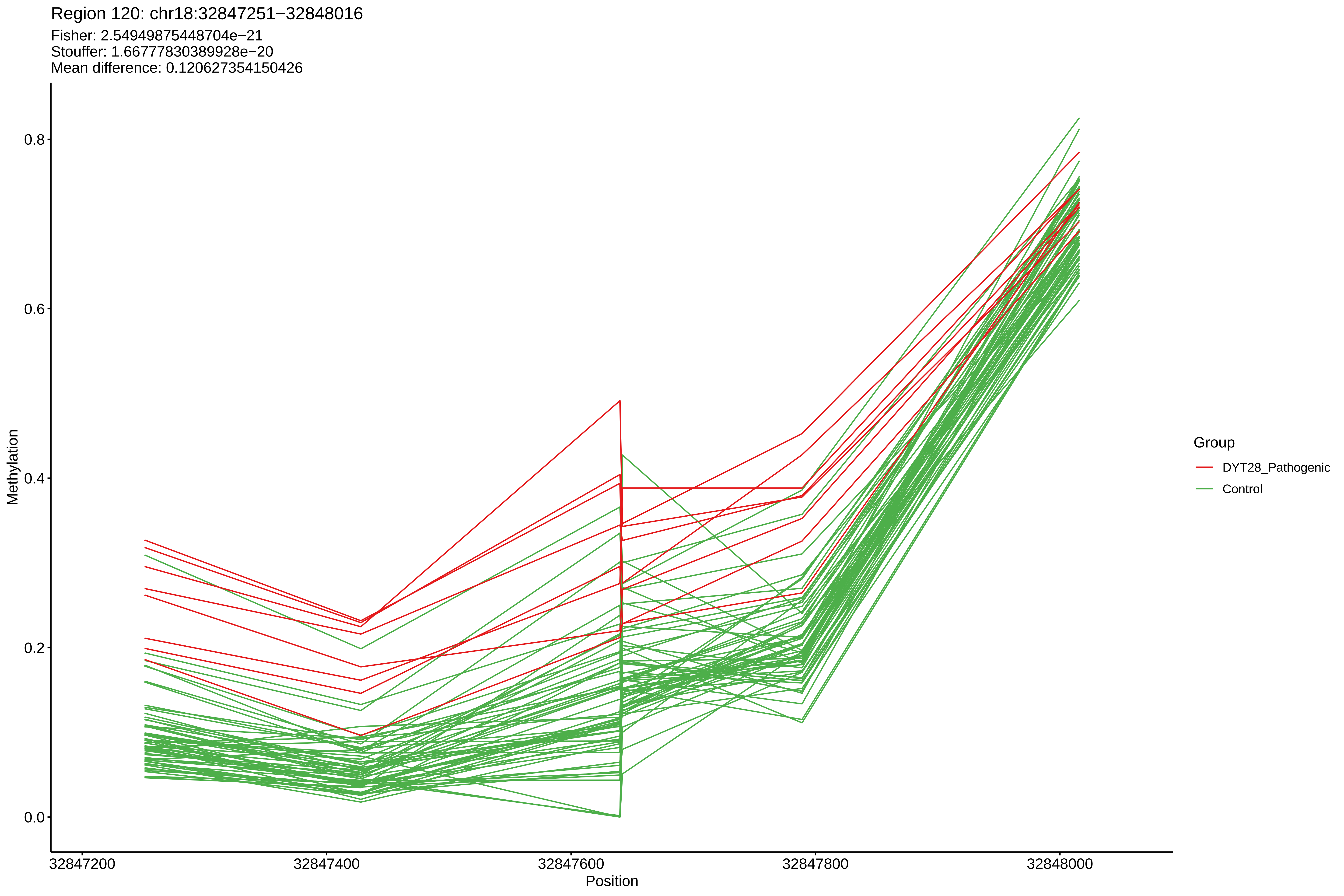
Region 120: chr18:32847251−32848016
Fisher: 2.54949875448704e−21
Stouffer: 1.66777830389928e−20
Mean difference: 0.120627354150426
Methylation
0.0
0.2
0.4
0.6
0.8
32847200
32847400
32847600
32847800
32848000
Position
Group
DYT28_Pathogenic
Control

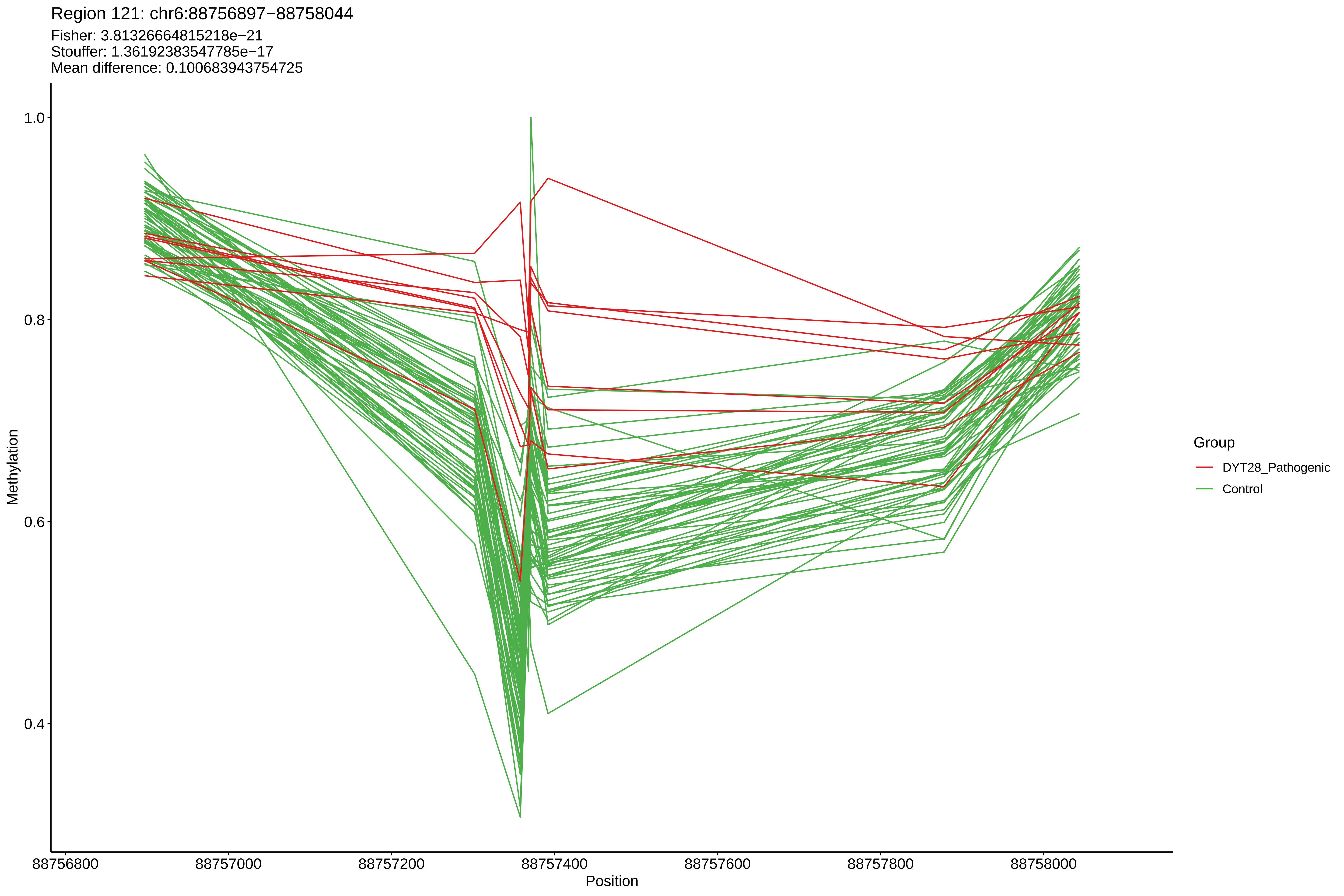

Region 121: chr6:88756897−88758044
Fisher: 3.81326664815218e−21
Stouffer: 1.36192383547785e−17
Mean difference: 0.100683943754725
Methylation
1.0
0.8
0.6
0.4
Position
88756800
88757000
88757200
88757400
88757600
88757800
88758000
Group
DYT28_Pathogenic
Control

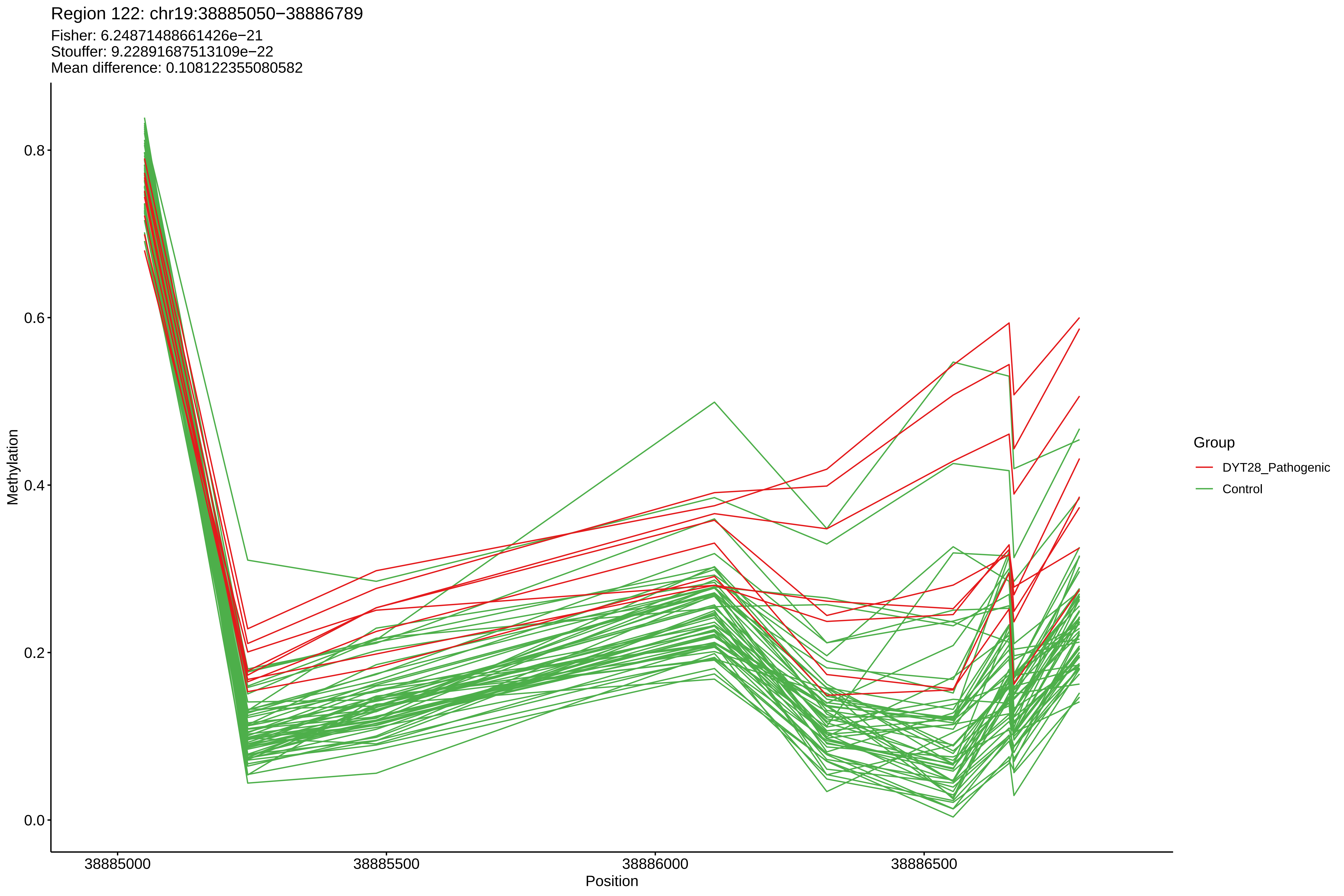

Region 122: chr19:38885050−38886789
Fisher: 6.24871488661426e−21
Stouffer: 9.22891687513109e−22
Mean difference: 0.108122355080582
Methylation
0.0
0.2
0.4
0.6
0.8
Position
38885000
38885500
38886000
38886500
Group
DYT28_Pathogenic
Control

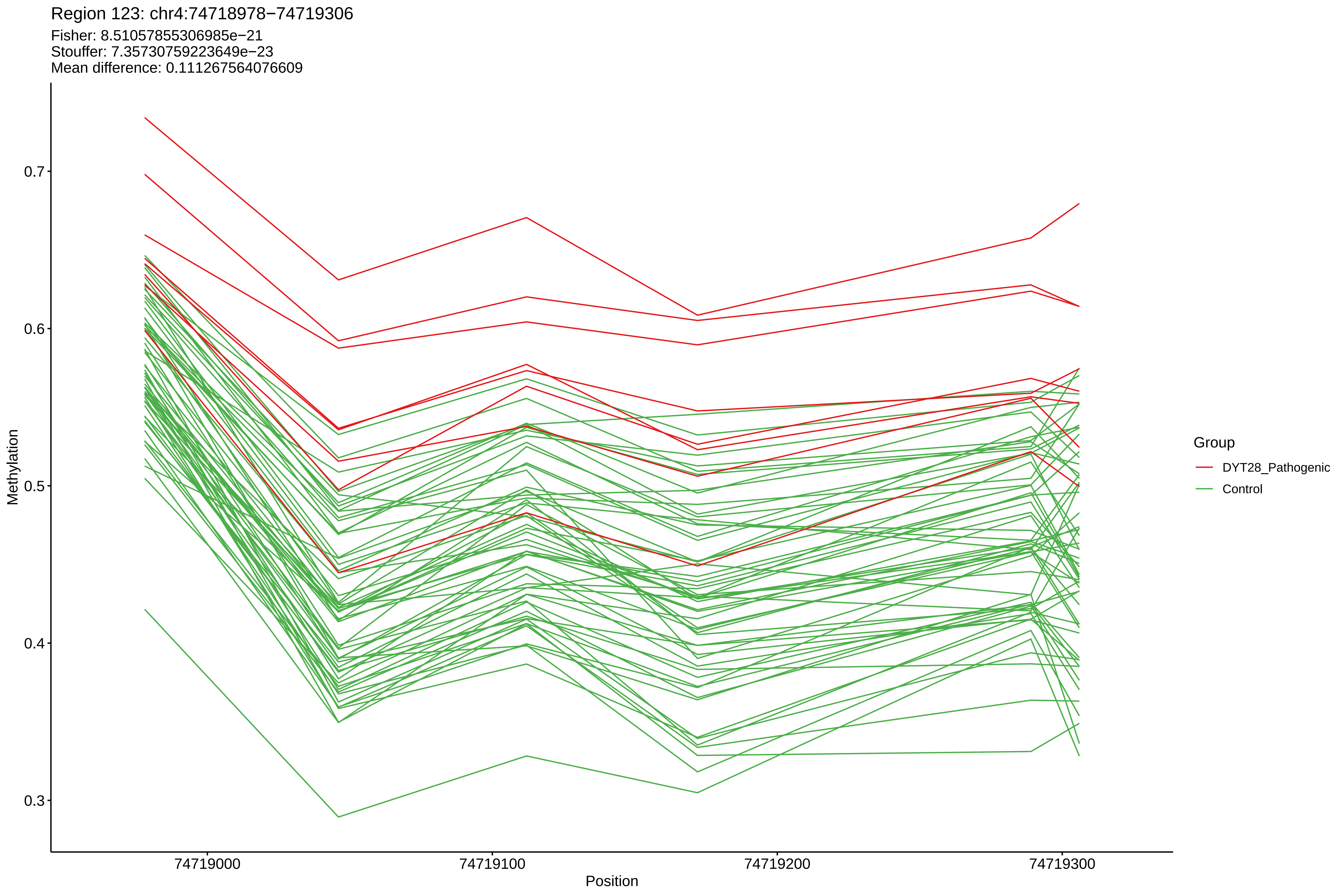

Region 123: chr4:74718978−74719306
Fisher: 8.51057855306985e−21
Stouffer: 7.35730759223649e−23
Mean difference: 0.111267564076609
0.7
0.6
0.5
0.4
0.3
Methylation
74719000
74719100
74719200
74719300
Position
Group
DYT28_Pathogenic
Control

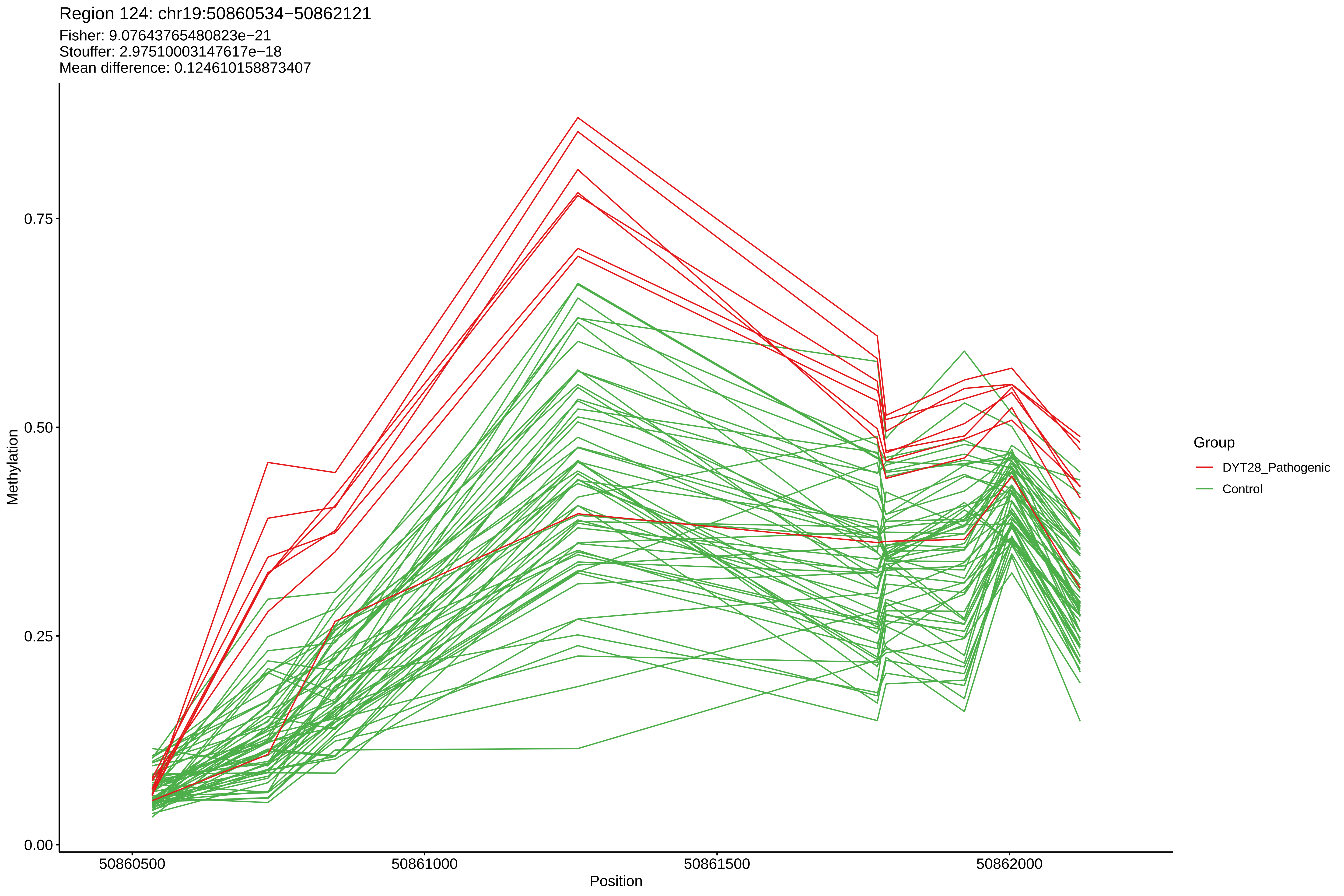
Region 124: chr19:50860534−50862121
Fisher: 9.07643765480823e−21
Stouffer: 2.97510003147617e−18
Mean difference: 0.124610158873407
Methylation
0.75
0.50
0.25
0.00
50860500
50861000
50861500
50862000
Position
Group
DYT28_Pathogenic
Control

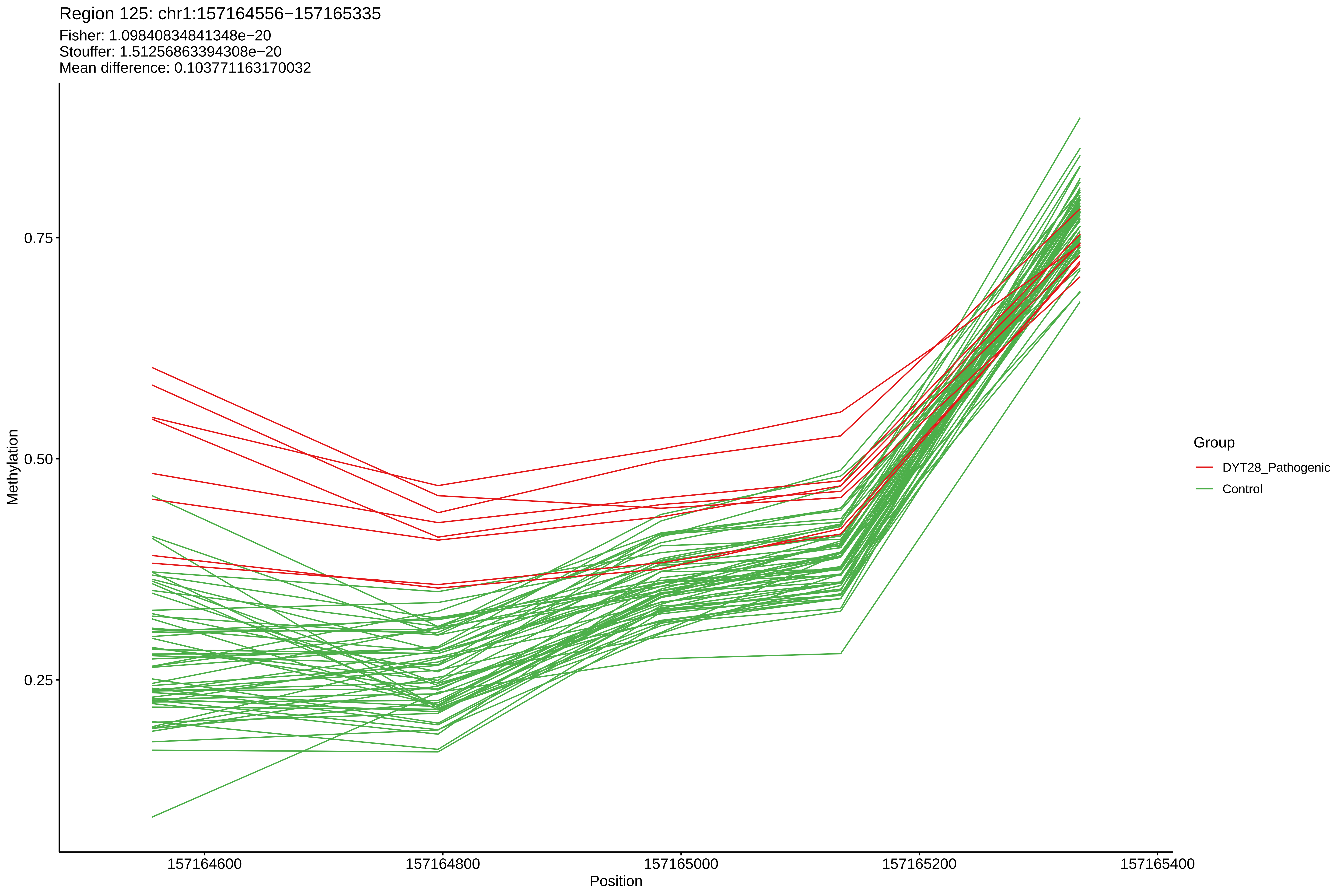

Region 125: chr1:157164556−157165335
Fisher: 1.09840834841348e−20
Stouffer: 1.51256863394308e−20
Mean difference: 0.103771163170032
Methylation
0.75
0.50
0.25
Position
157164600
157164800
157165000
157165200
157165400
Group
DYT28_Pathogenic
Control

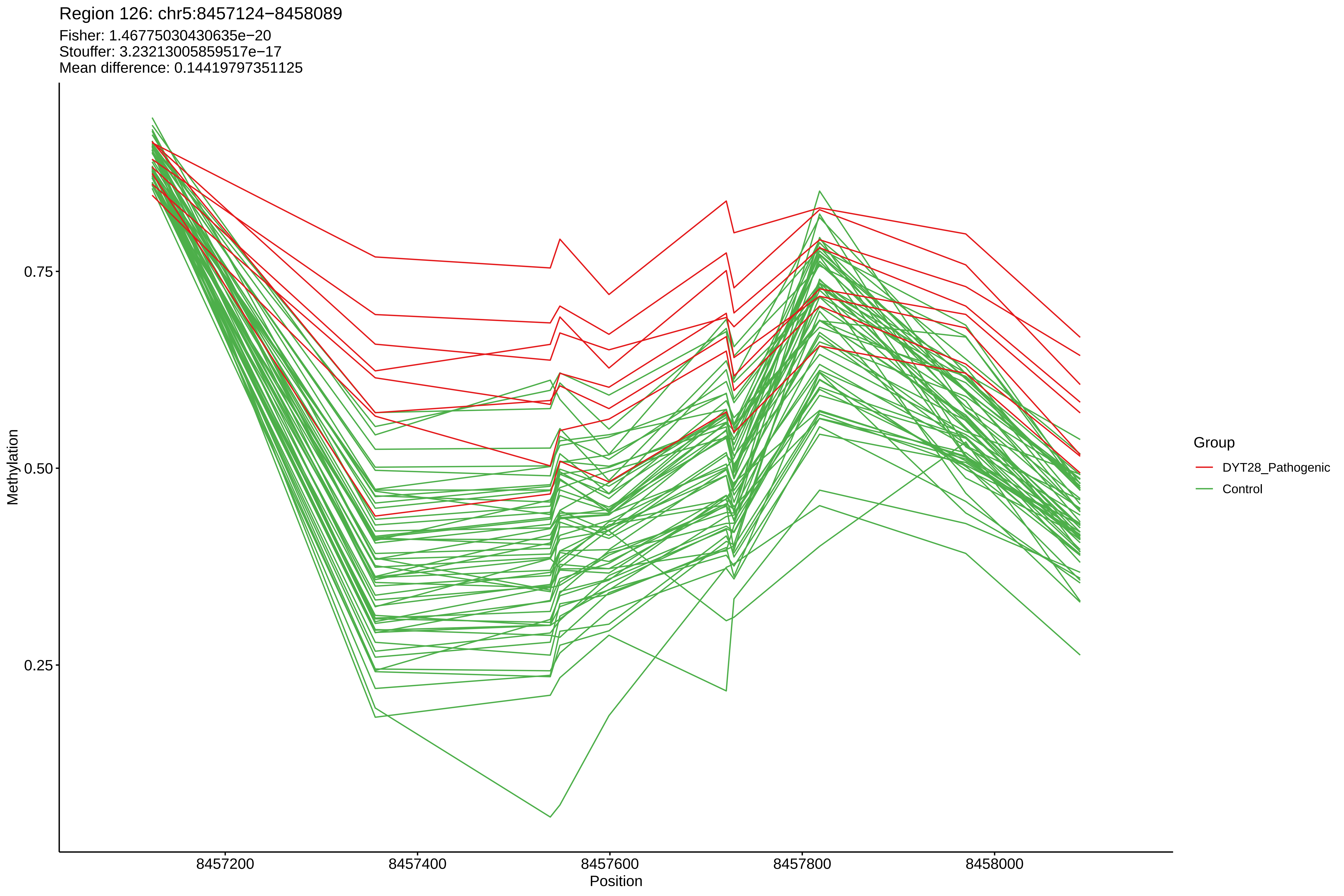

Region 126: chr5:8457124−8458089
Fisher: 1.46775030430635e−20
Stouffer: 3.23213005859517e−17
Mean difference: 0.14419797351125
Methylation
0.75
0.50
0.25
8457200
8457400
8457600
8457800
8458000
Position
Group
DYT28_Pathogenic
Control

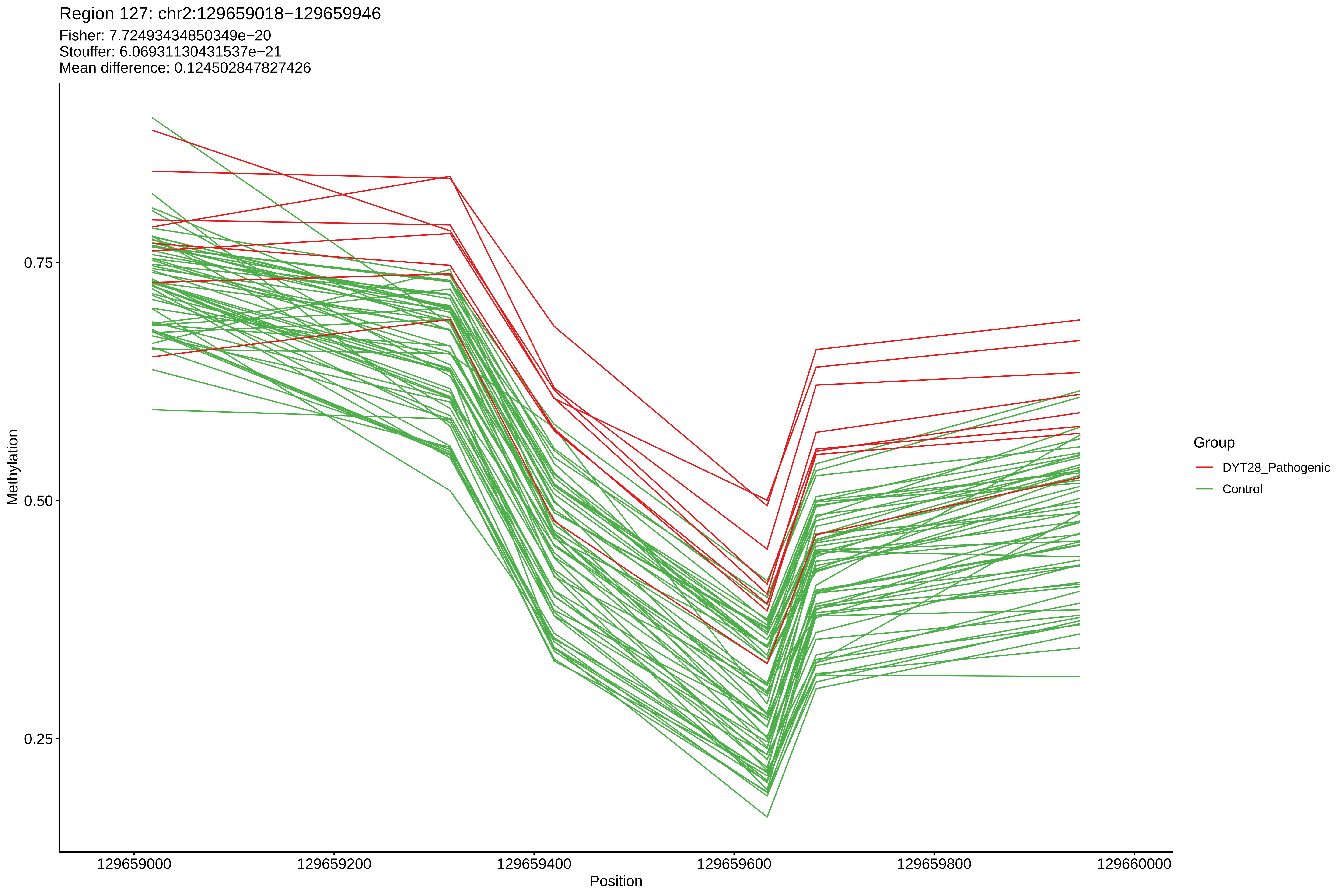

Region 127: chr2:129659018−129659946
Fisher: 7.72493434850349e−20
Stouffer: 6.06931130431537e−21
Mean difference: 0.124502847827426
Methylation
0.75
0.50
0.25
Position
129659000
129659200
129659400
129659600
129659800
129660000
Group
DYT28_Pathogenic
Control

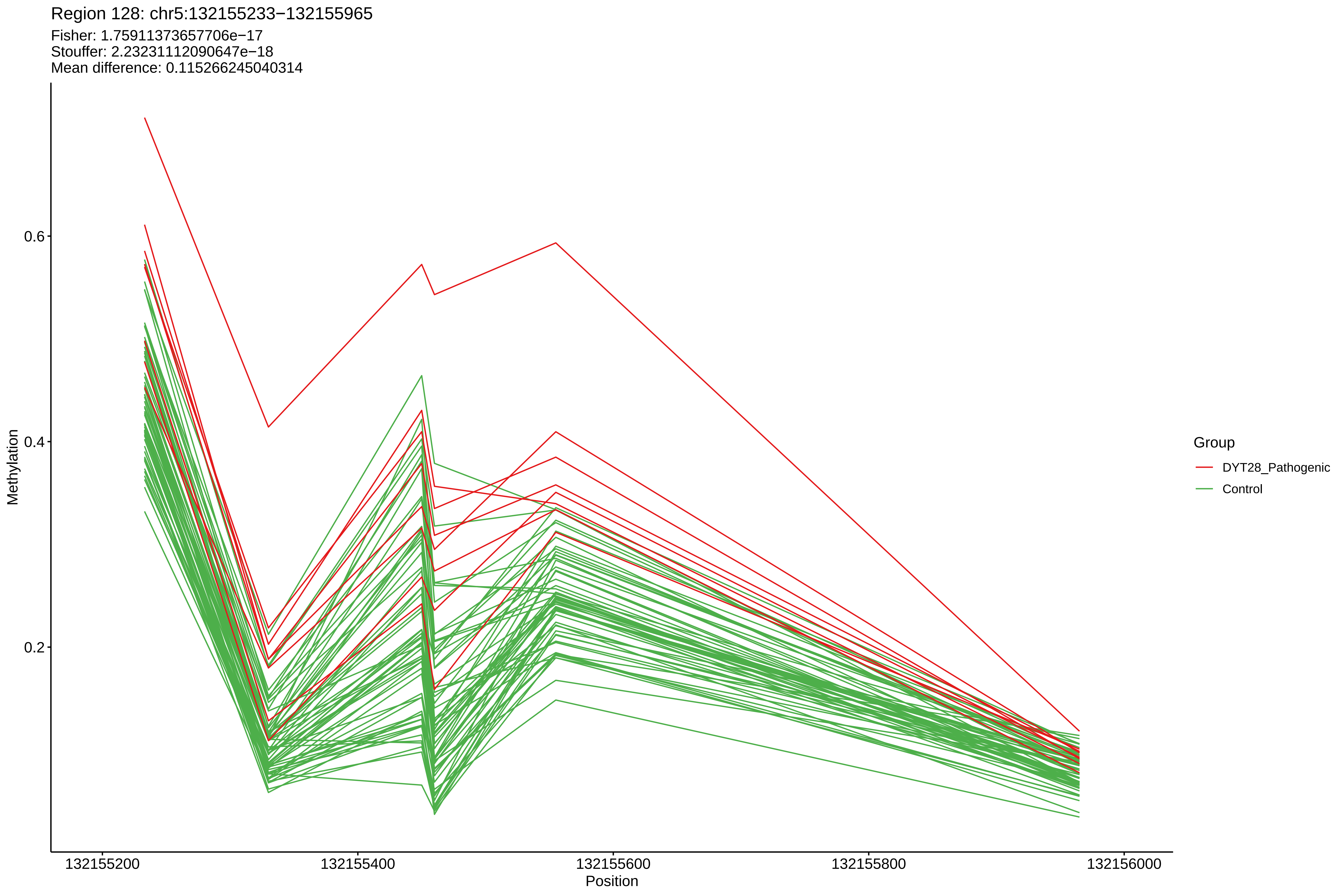

Region 128: chr5:132155233−132155965
Fisher: 1.75911373657706e−17
Stouffer: 2.23231112090647e−18
Mean difference: 0.115266245040314
Methylation
0.6
0.4
0.2
132155200
132155400
132155600
132155800
132156000
Position
Group
DYT28_Pathogenic
Control

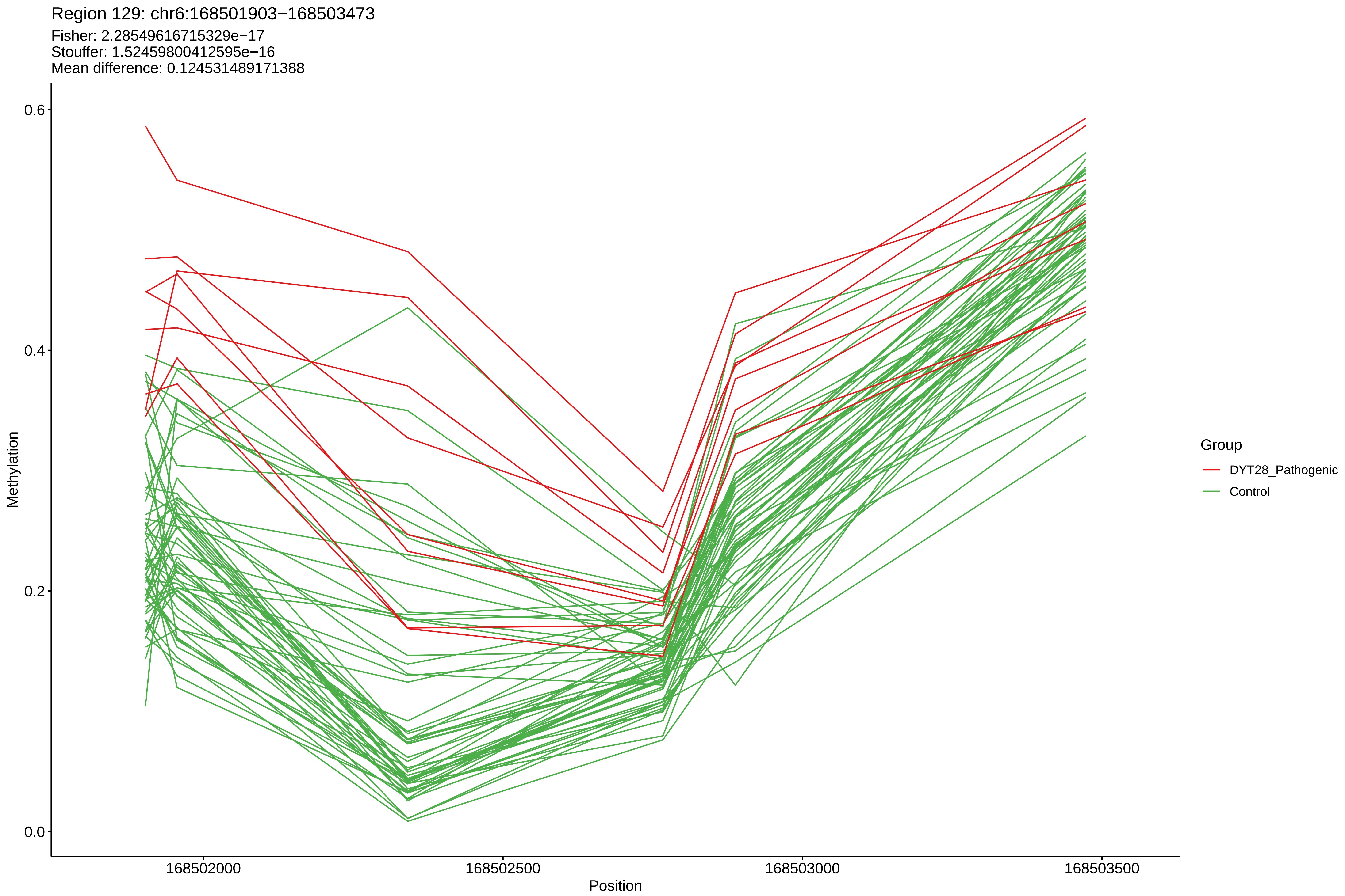
Region 129: chr6:168501903−168503473
Fisher: 2.28549616715329e−17
Stouffer: 1.52459800412595e−16
Mean difference: 0.124531489171388
Methylation
0.6
0.4
0.2
0.0
168502000
168502500
168503000
168503500
Position
Group
DYT28_Pathogenic
Control

Region 130: chr7:149389444−149389941

Fisher: 4.31991368609496e−17
Stouffer: 2.46565906869148e−17
Mean difference: 0.102694014764301

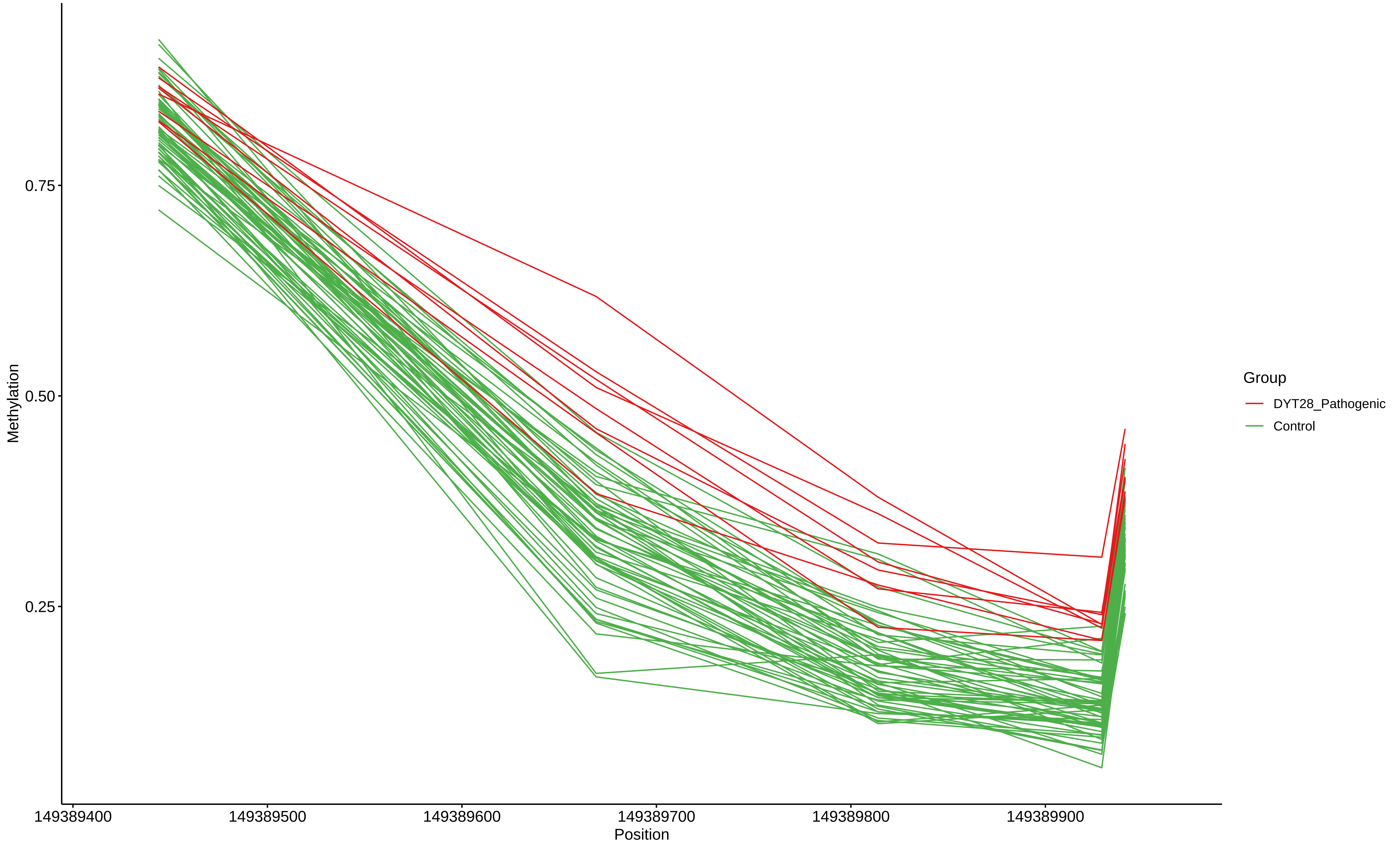

Region 131: chr6:166422182−166422741
Fisher: 5.61012226647902e−17
Stouffer: 4.08809585299748e−18
Mean difference: 0.101948780904776

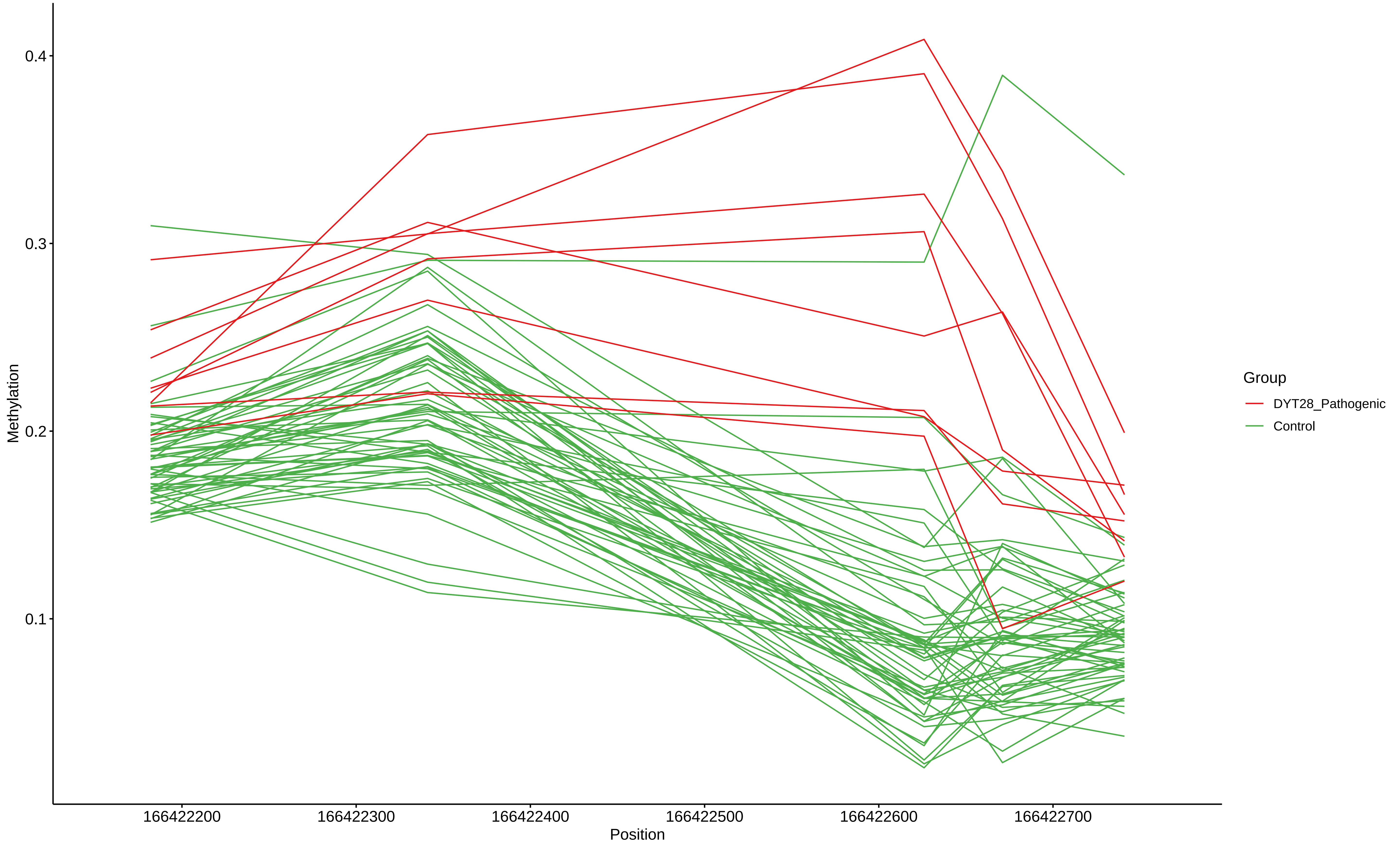

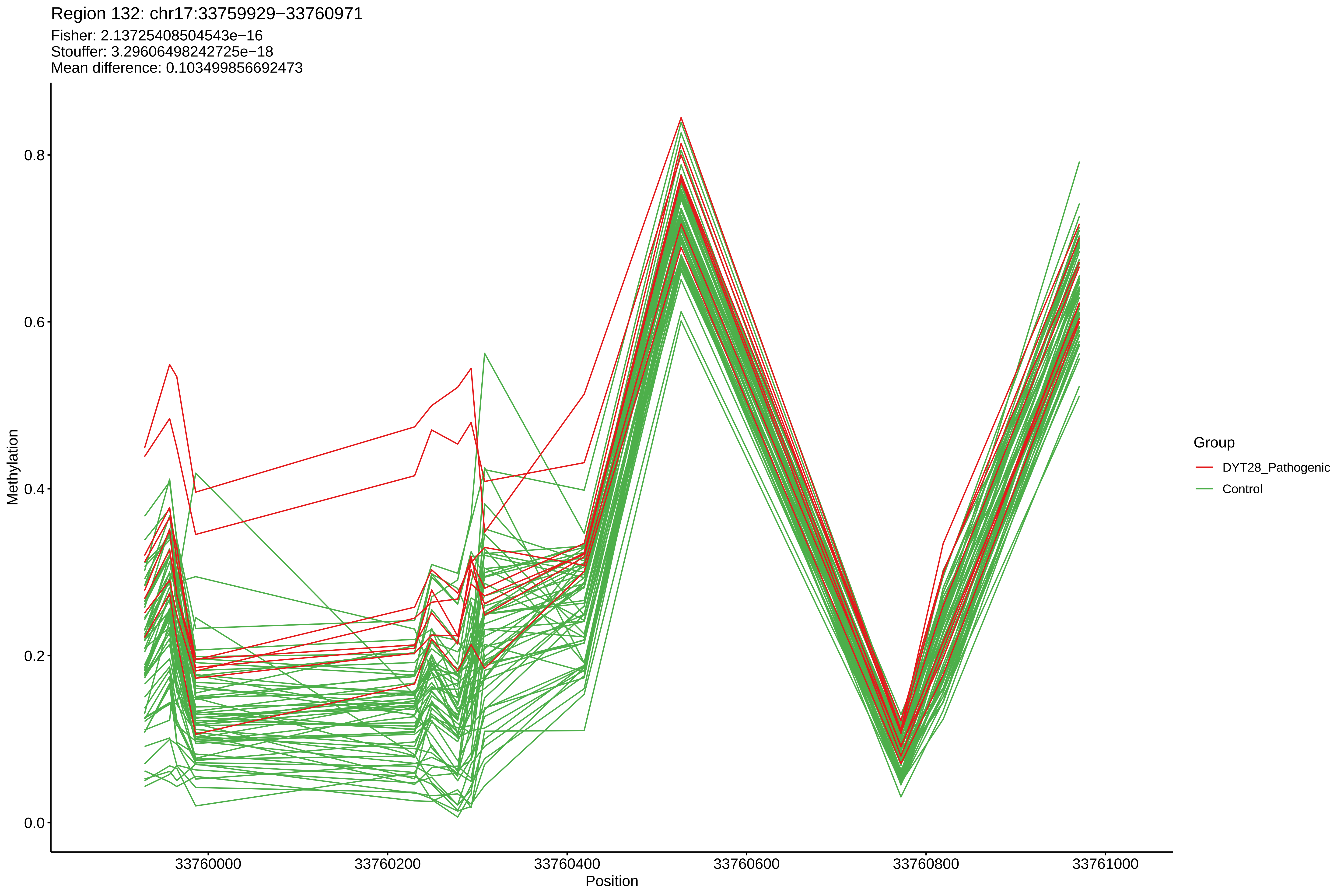
Region 132: chr17:33759929−33760971
Fisher: 2.13725408504543e−16
Stouffer: 3.29606498242725e−18
Mean difference: 0.103499856692473
Methylation
0.0
0.2
0.4
0.6
0.8
33760000
33760200
33760400
33760600
33760800
33761000
Position
Group
DYT28_Pathogenic
Control

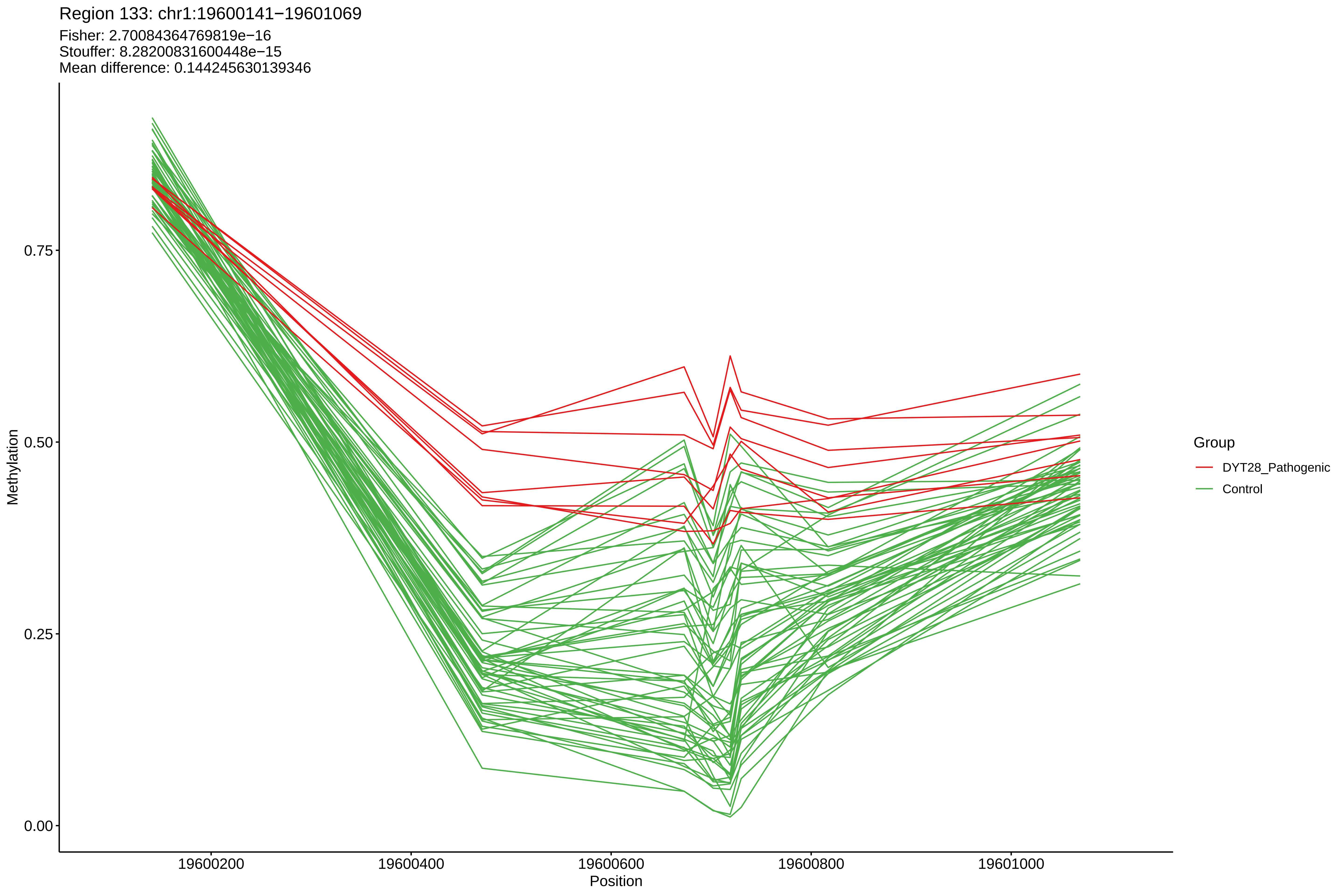
Region 133: chr1:19600141−19601069
Fisher: 2.70084364769819e−16
Stouffer: 8.28200831600448e−15
Mean difference: 0.144245630139346
Methylation
0.00
0.25
0.50
0.75
19600200
19600400
19600600
19600800
19601000
Position
Group
DYT28_Pathogenic
Control

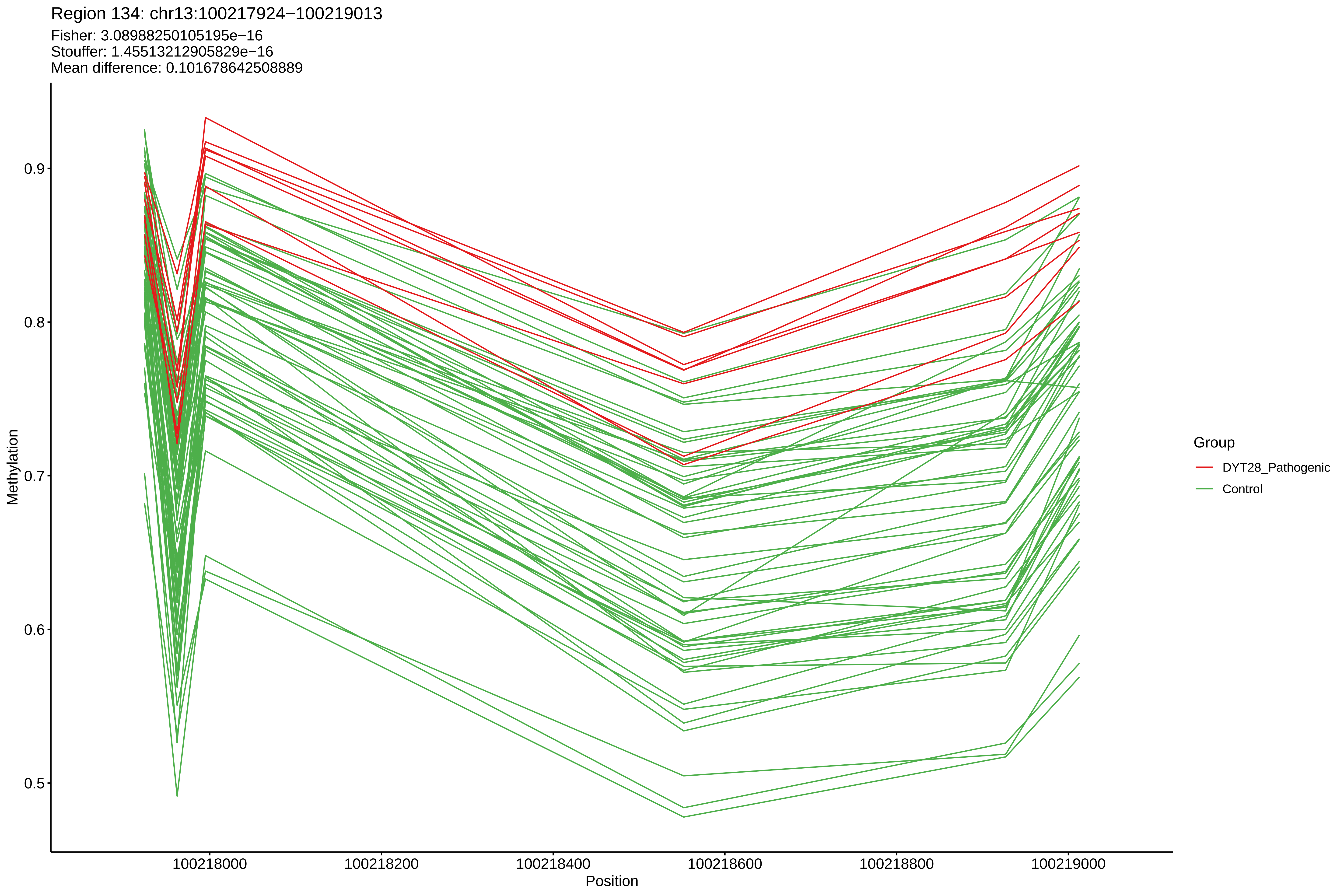

Region 134: chr13:100217924−100219013
Fisher: 3.08988250105195e−16
Stouffer: 1.45513212905829e−16
Mean difference: 0.101678642508889
Methylation
0.9
0.8
0.7
0.6
0.5
100218000
100218200
100218400
100218600
100218800
100219000
Position
Group
DYT28_Pathogenic
Control

Region 135: chr2:118616377−118618593

Fisher: 8.34568931459336e−16
Stouffer: 1.67563354241717e−14
Mean difference: 0.10087465198642

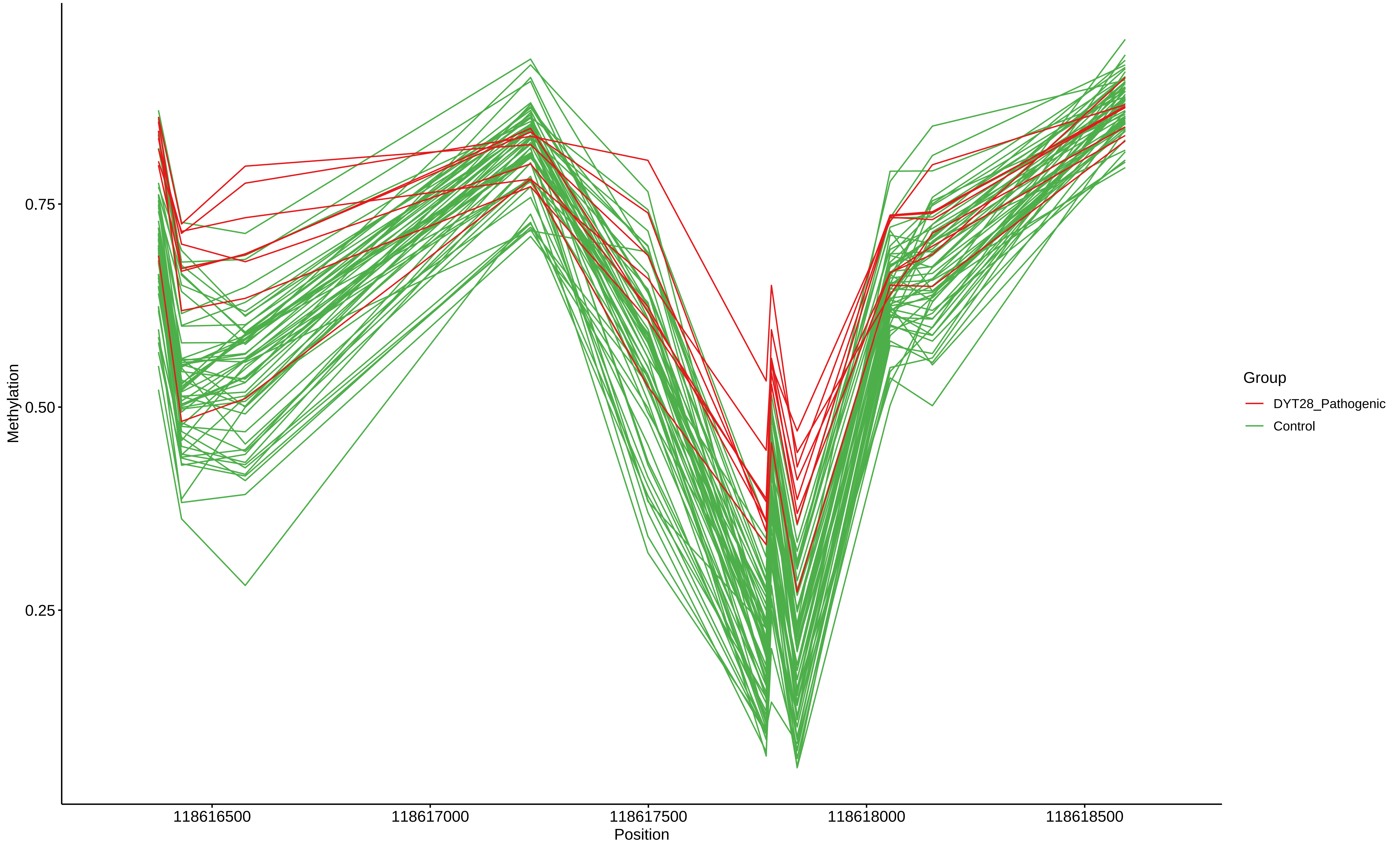

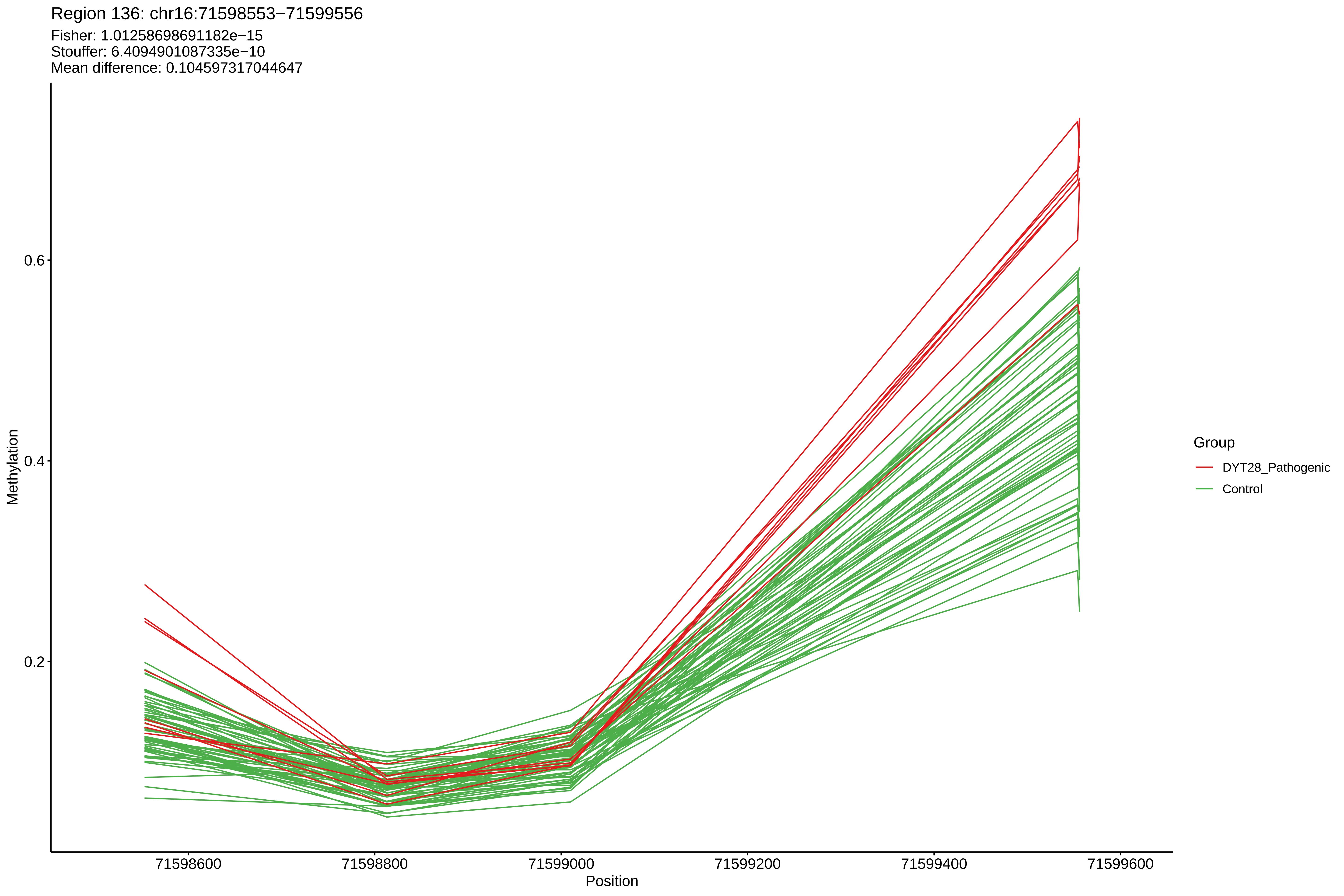
Region 136: chr16:71598553−71599556
Fisher: 1.01258698691182e−15
Stouffer: 6.4094901087335e−10
Mean difference: 0.104597317044647
Methylation
0.6
0.4
0.2
71598600
71598800
71599000
71599200
71599400
71599600
Position
Group
DYT28_Pathogenic
Control

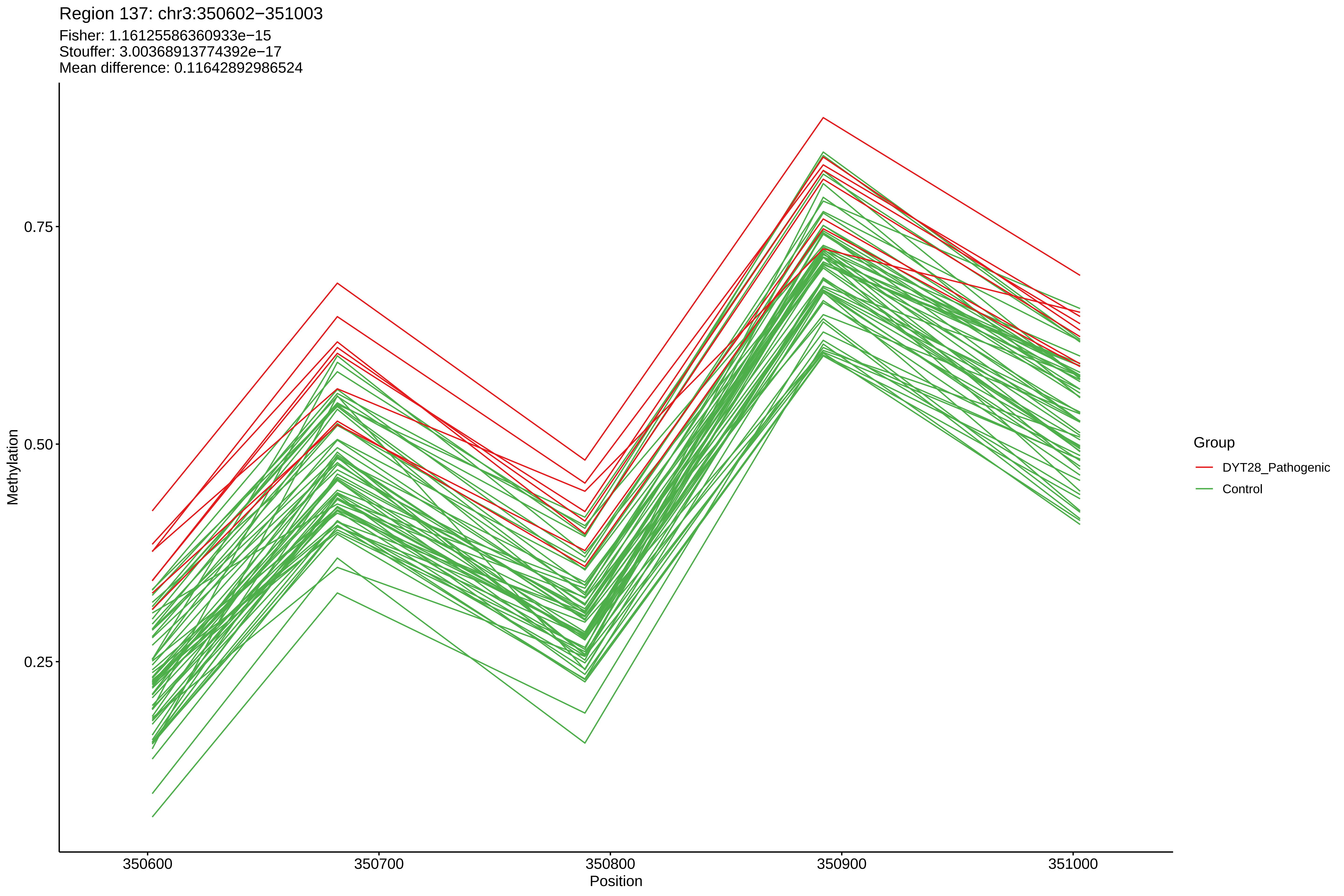
Region 137: chr3:350602−351003
Fisher: 1.16125586360933e−15
Stouffer: 3.00368913774392e−17
Mean difference: 0.11642892986524
Methylation
0.75
0.50
0.25
350600
350700
350800
350900
351000
Position
Group
DYT28_Pathogenic
Control

Region 138: chr17:42430376−42432488

Fisher: 1.52381661770405e−15
Stouffer: 3.99779651972259e−14
Mean difference: 0.127490073580688

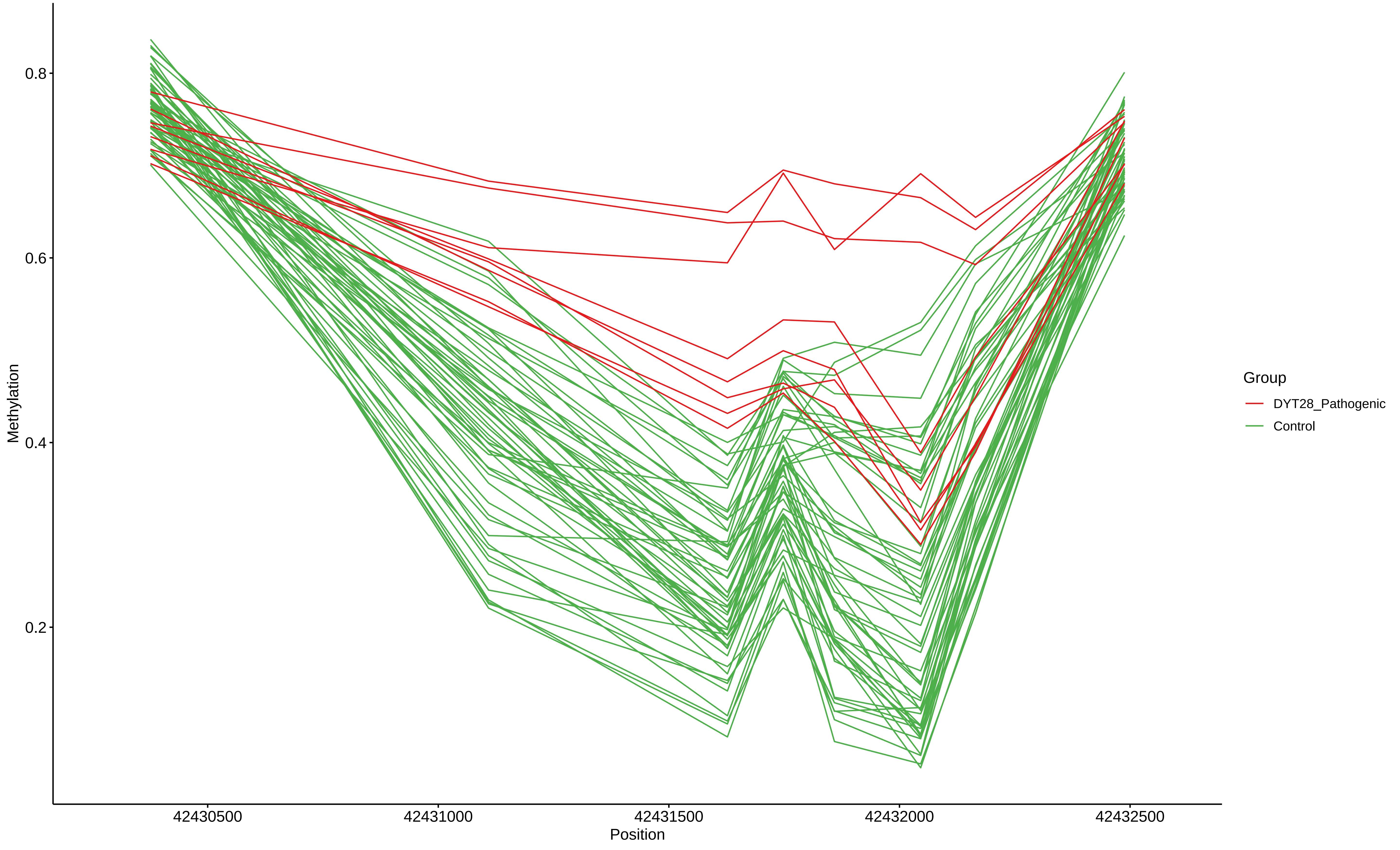

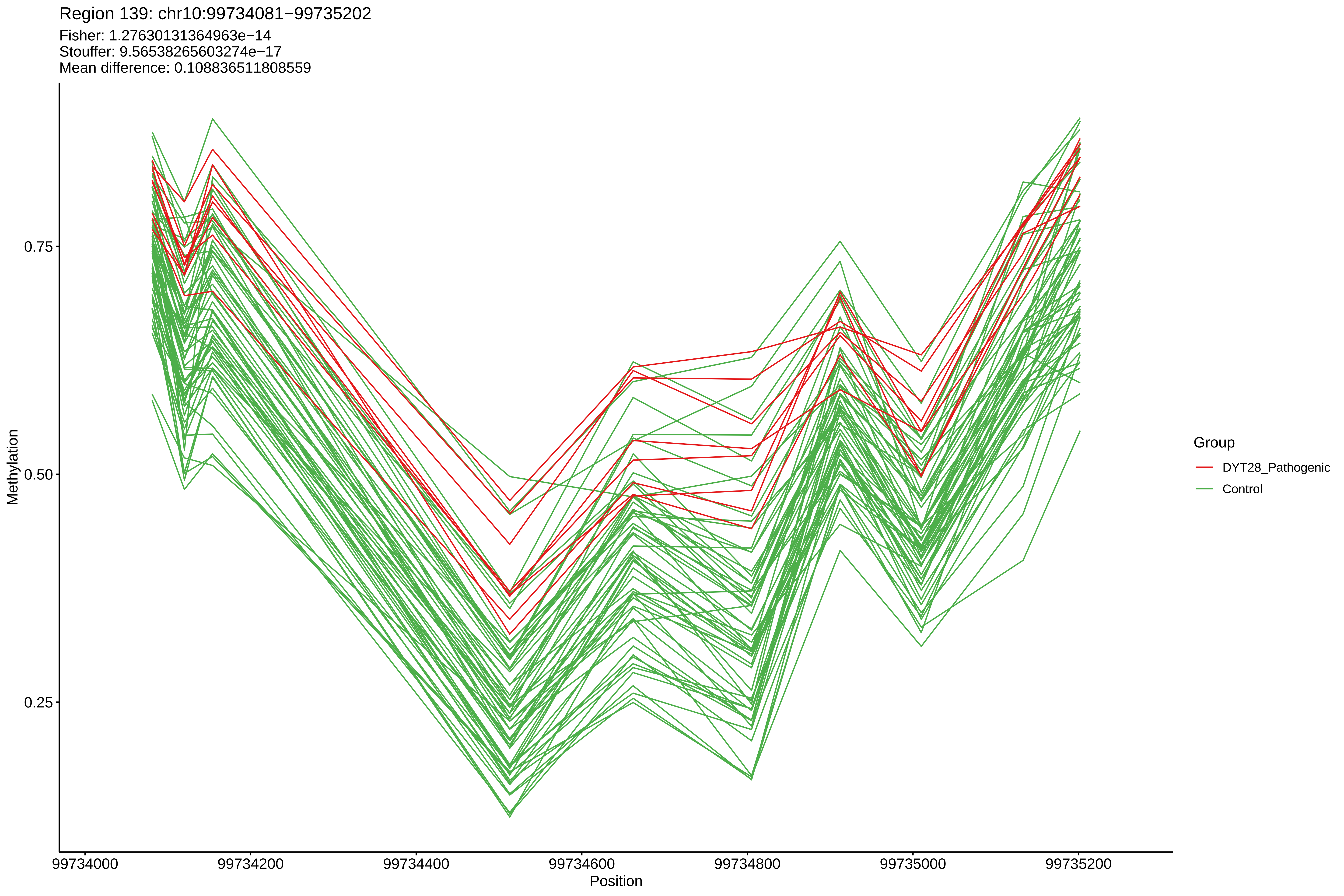

Region 139: chr10:99734081−99735202
Fisher: 1.27630131364963e−14
Stouffer: 9.56538265603274e−17
Mean difference: 0.108836511808559
Methylation
0.75
0.50
0.25
99734000
99734200
99734400
99734600
99734800
99735000
99735200
Position
Group
DYT28_Pathogenic
Control

Region 140: chr4:25090198−25090665

Fisher: 8.82471296798307e−14
Stouffer: 1.73596203246899e−15
Mean difference: 0.159078554080391

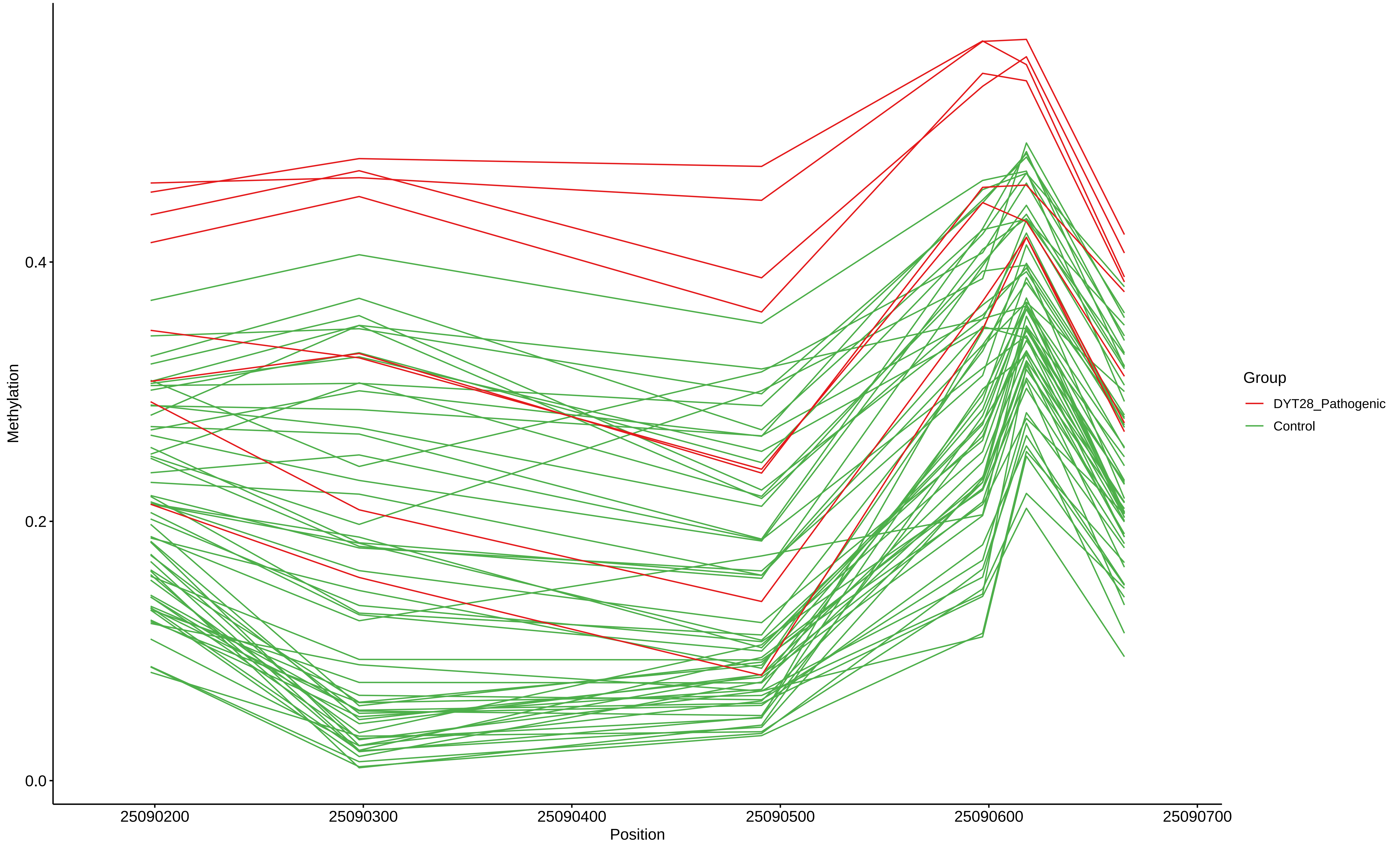

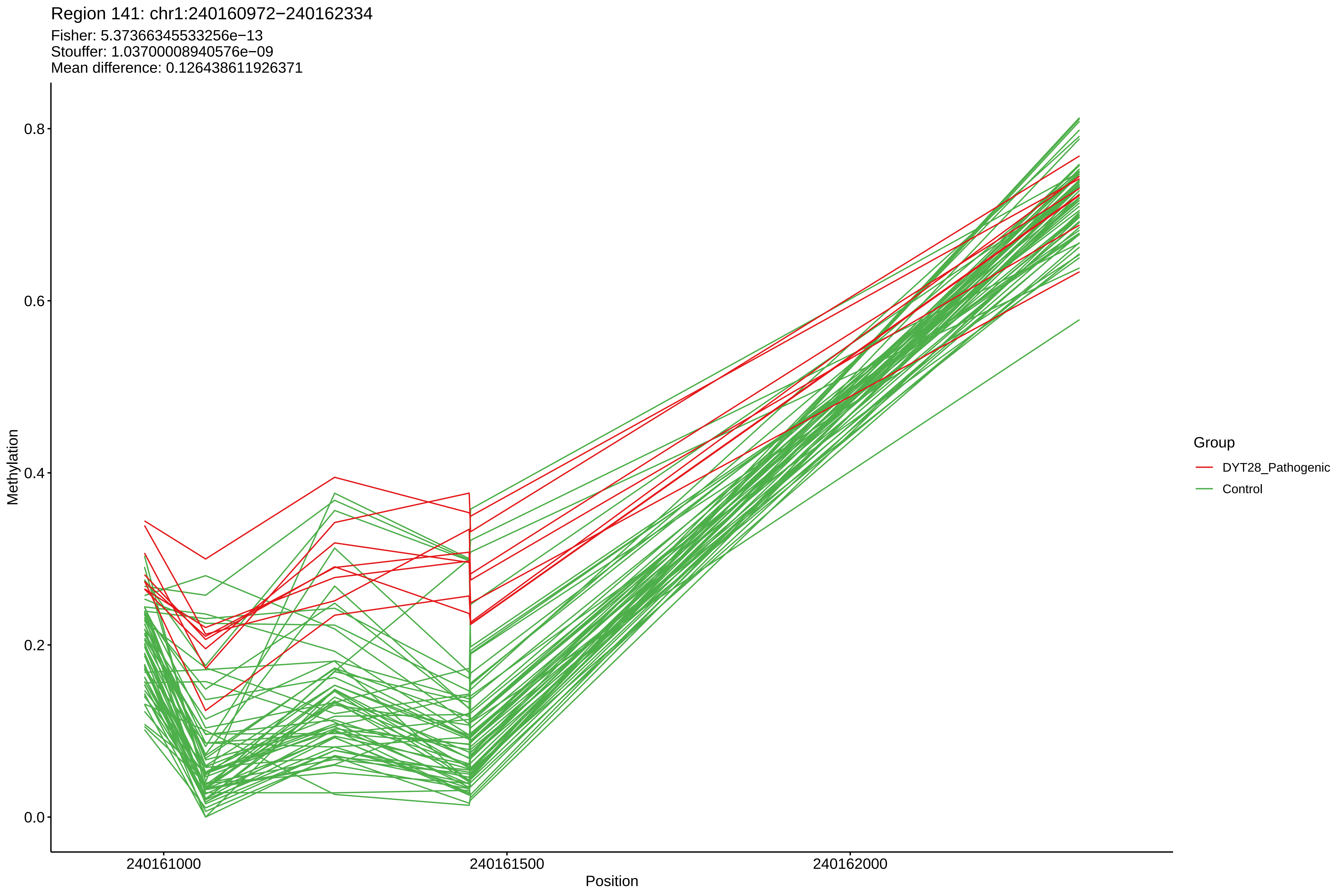
Region 141: chr1:240160972−240162334
Fisher: 5.37366345533256e−13
Stouffer: 1.03700008940576e−09
Mean difference: 0.126438611926371
Methylation
0.0
0.2
0.4
0.6
0.8
240161000
240161500
240162000
Position
Group
DYT28_Pathogenic
Control

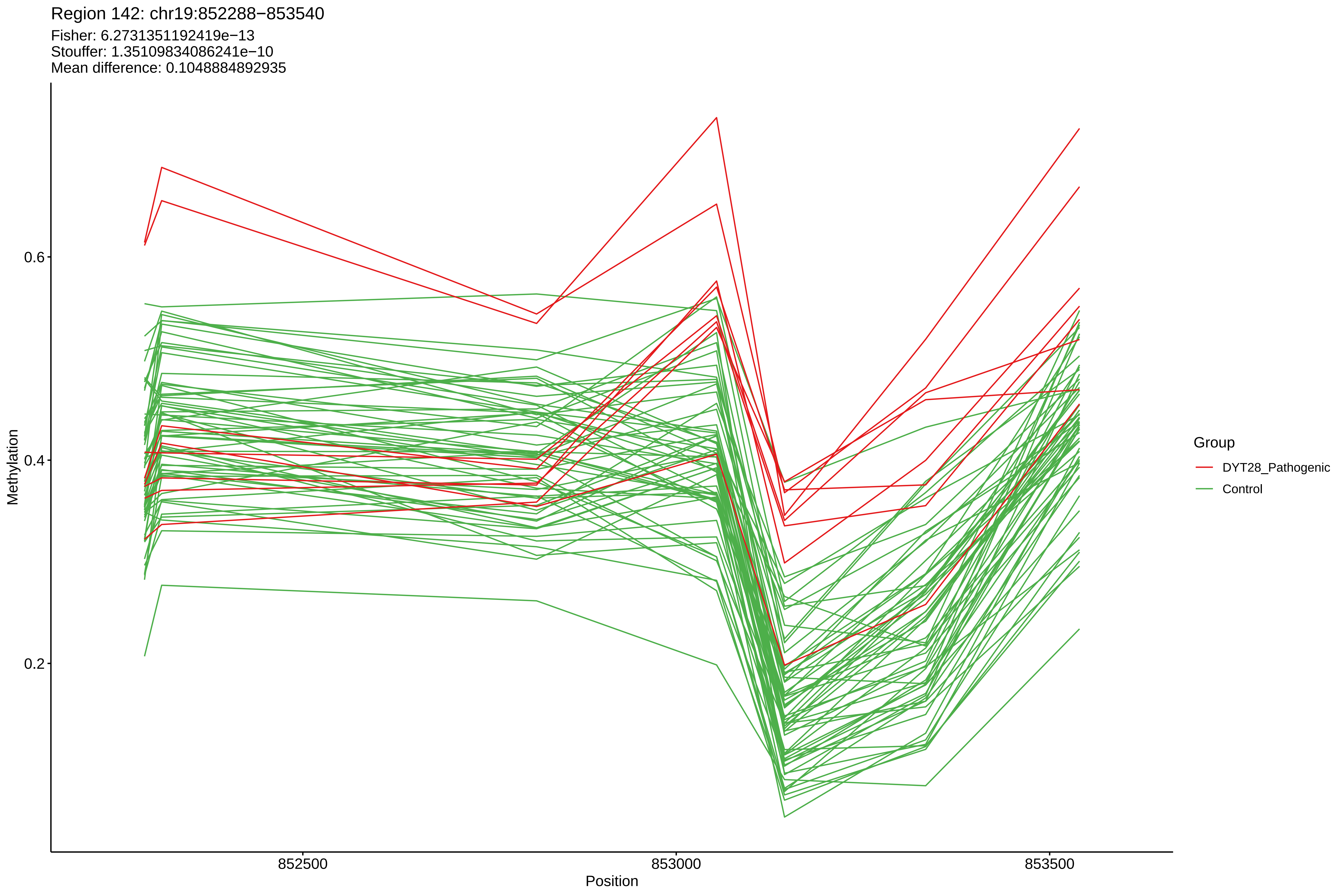
Region 142: chr19:852288−853540
Fisher: 6.2731351192419e−13
Stouffer: 1.35109834086241e−10
Mean difference: 0.1048884892935
0.6
0.4
0.2
Methylation
852500
853000
853500
Position
Group
DYT28_Pathogenic
Control

Region 143: chr7:51538921−51539678
Fisher: 1.7382148099561e−12
Stouffer: 3.16704599355341e−14
Mean difference: 0.100872114885186

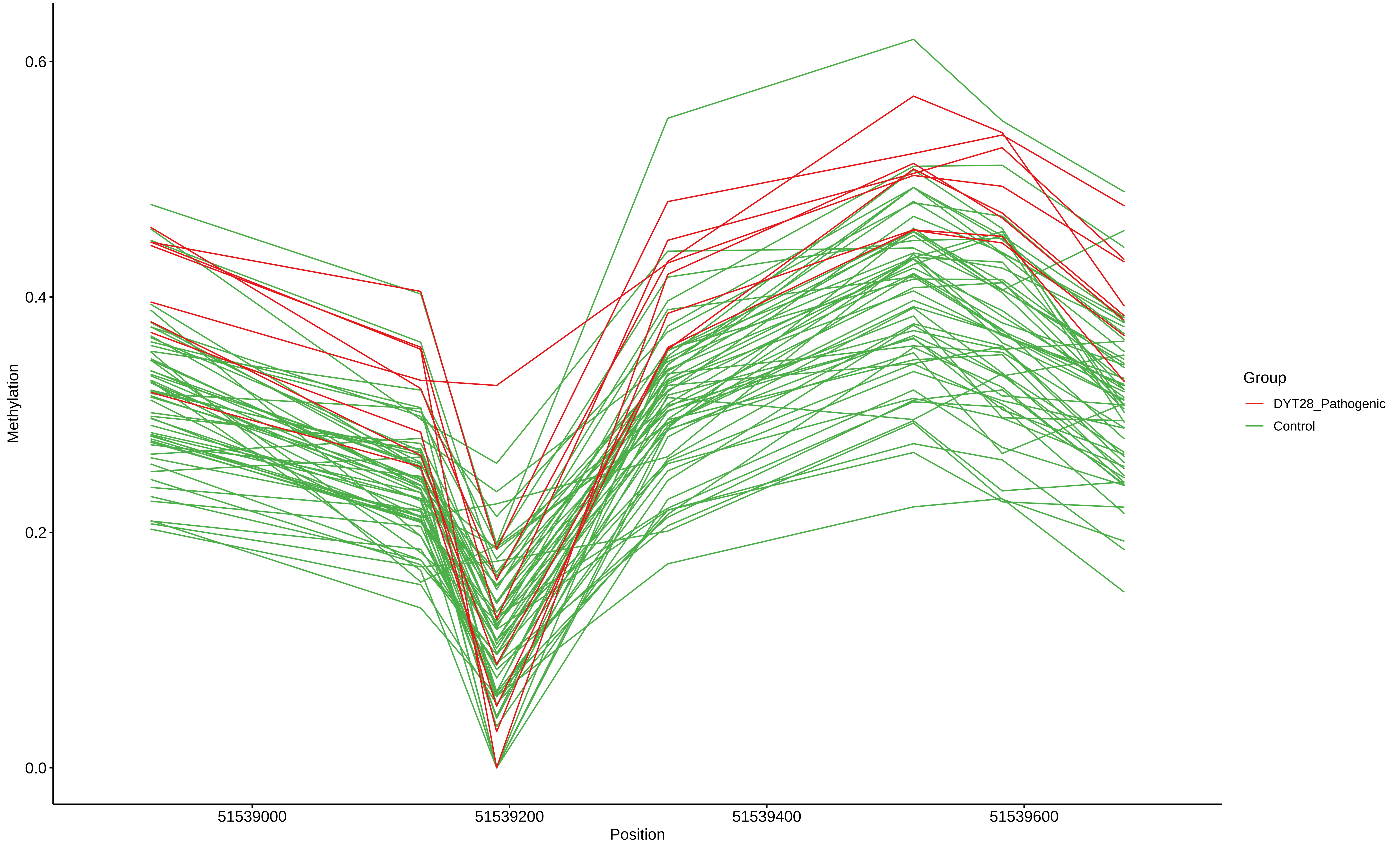

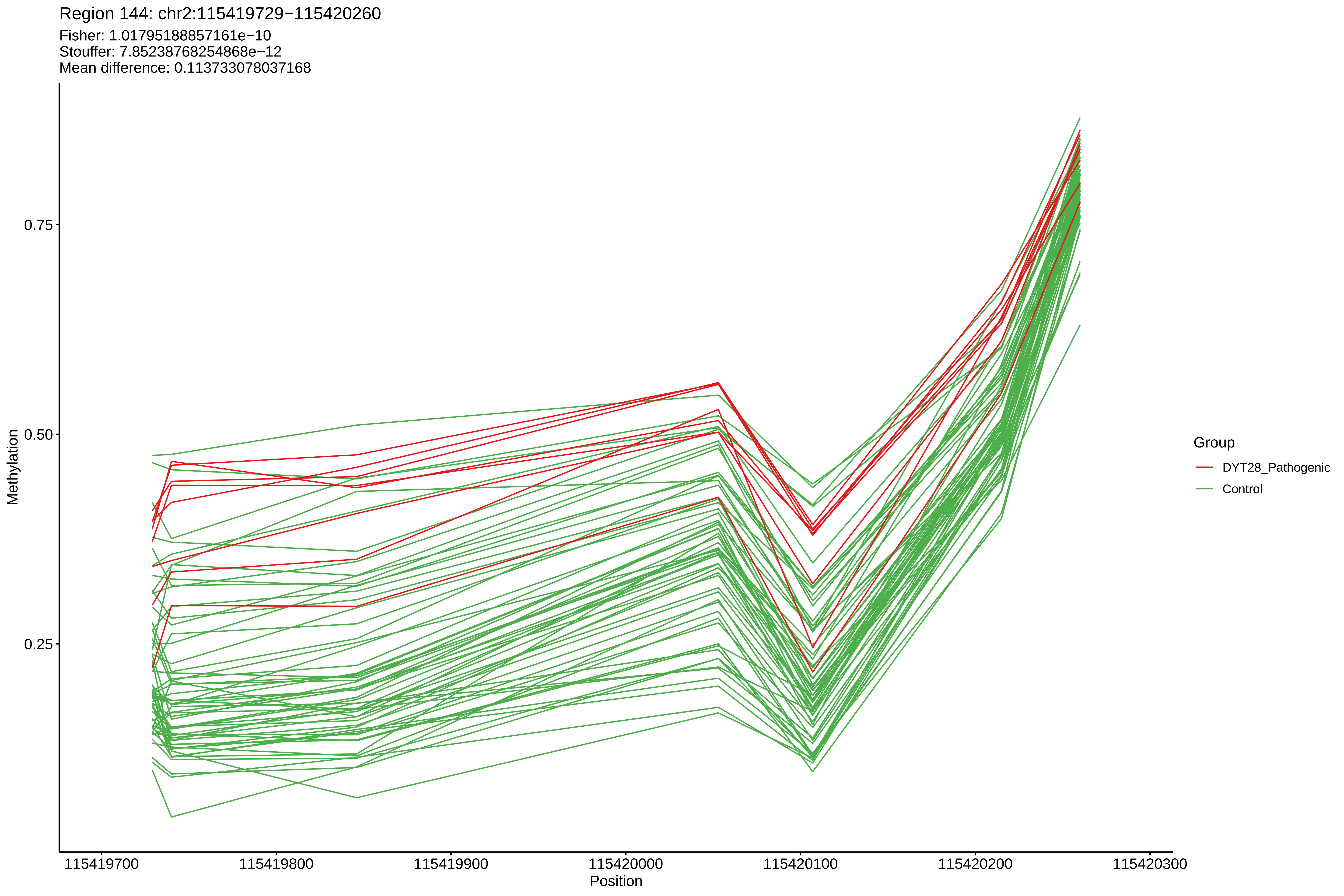
Region 144: chr2:115419729−115420260
Fisher: 1.01795188857161e−10
Stouffer: 7.85238768254868e−12
Mean difference: 0.113733078037168
Methylation
0.75
0.50
0.25
Position
115419700
115419800
115419900
115420000
115420100
115420200
115420300
Group
DYT28_Pathogenic
Control

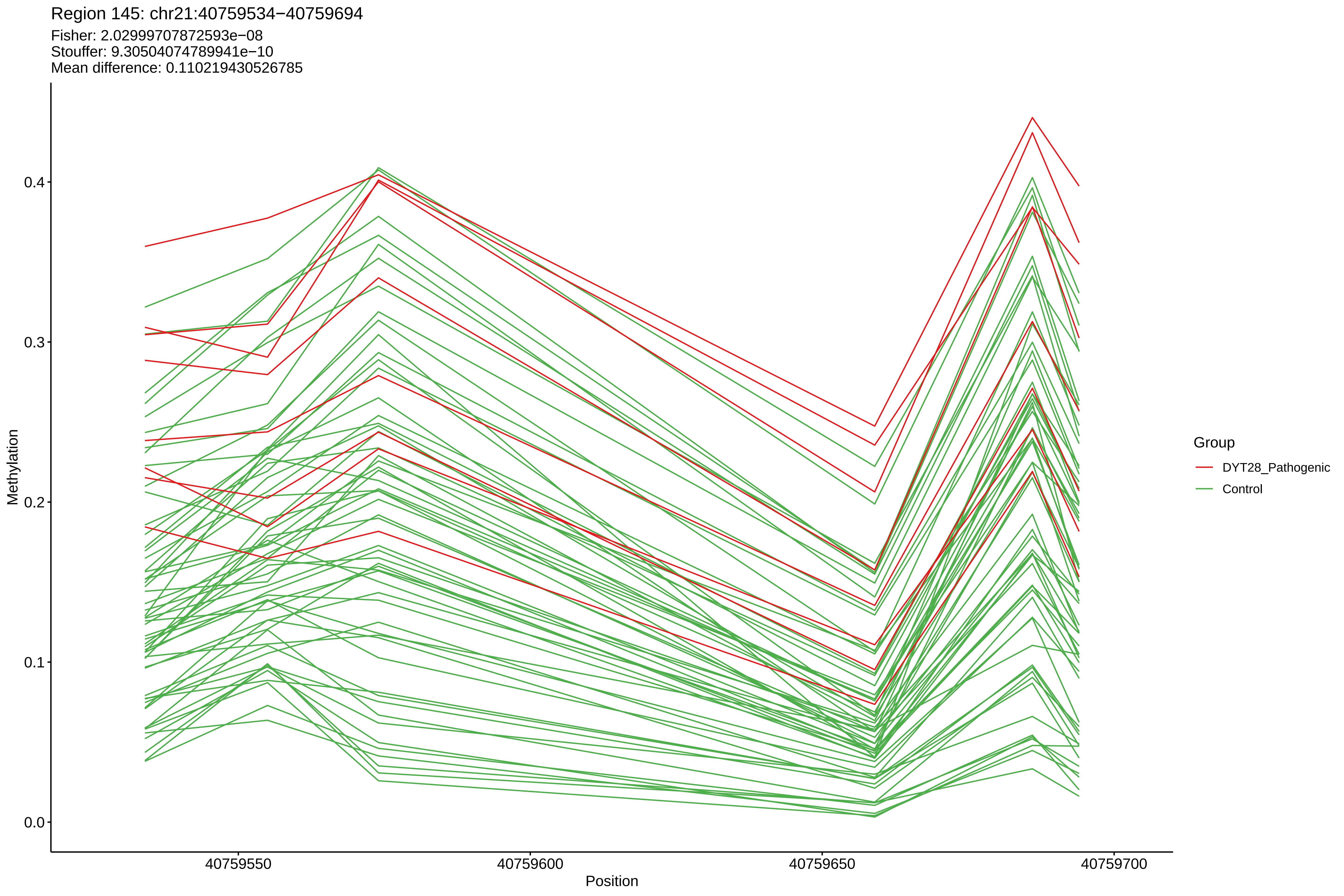
Region 145: chr21:40759534−40759694
Fisher: 2.02999707872593e−08
Stouffer: 9.30504074789941e−10
Mean difference: 0.110219430526785
Methylation
0.0
0.1
0.2
0.3
0.4
40759550
40759600
40759650
40759700
Position
Group
DYT28_Pathogenic
Control

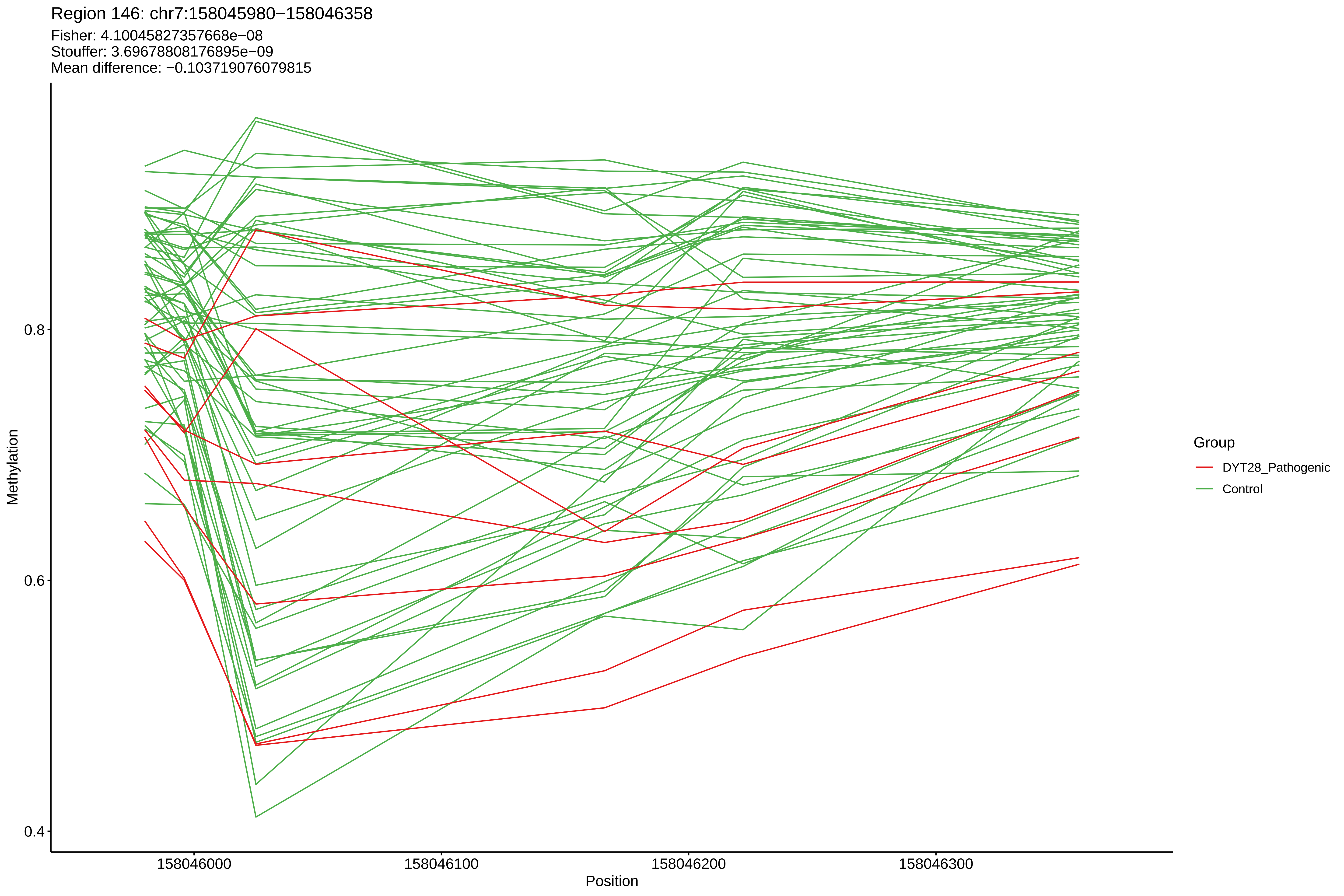
Region 146: chr7:158045980−158046358
Fisher: 4.10045827357668e−08
Stouffer: 3.69678808176895e−09
Mean difference: −0.103719076079815
Methylation
0.8
0.6
0.4
158046000
158046100
158046200
158046300
Position
Group
DYT28_Pathogenic
Control
